# IsomiR expression profiles in human lymphoblastoid cell lines exhibit population and gender dependencies

**DOI:** 10.18632/oncotarget.2405

**Published:** 2014-08-27

**Authors:** Phillipe Loher, Eric R. Londin, Isidore Rigoutsos

**Affiliations:** ^1^ Computational Medicine Center, Sidney Kimmel Medical College at Thomas Jefferson University, Philadelphia, PA

**Keywords:** microRNAs, miRNAs, isomiRs, 1000 Genomes

## Abstract

For many years it was believed that each mature microRNA (miRNA) existed as a single entity with fixed endpoints and a ‘static’ and unchangeable primary sequence. However, recent evidence suggests that mature miRNAs are more ‘dynamic’ and that each miRNA precursor arm gives rise to multiple isoforms, the isomiRs. Here we report on our identification of numerous and abundant isomiRs in the lymphoblastoid cell lines (LCLs) of 452 men and women from five different population groups. Unexpectedly, we find that these isomiRs exhibit an expression profile that is population-dependent and gender-dependent. This is important as it indicates that the LCLs of each gender/population combination have their own unique collection of mature miRNA transcripts. Moreover, each identified isomiR has its own characteristic abundance that remains consistent across biological replicates indicating that these are not degradation products. The primary sequences of identified isomiRs differ from the known miRBase miRNA either at their 5´-endpoint (leads to a different ‘seed’ sequence and suggests a different targetome), their 3´-endpoint, or both simultaneously. Our analysis of Argonaute PAR-CLIP data from LCLs supports the association of many of these newly identified isomiRs with the Argonaute silencing complex and thus their functional roles through participation in the RNA interference pathway.

## INTRODUCTION

MicroRNAs (miRNAs) are small single-stranded noncoding RNAs of roughly 22 nucleotides (nts) in length that regulate their targets in a sequence-dependent manner and affect their roles through either degradation or translational repression [1, 2]. MiRNAs are ubiquitously expressed across cell types and found to regulate a diverse array of cellular processes in health and disease [3-6].

MiRNAs are transcribed as large primary transcripts (pri-mRNA) of RNA polymerase II, then processed into shorter ~70 nt hairpin loop precursors (pre-miRNAs) by the nuclear RNase III protein Drosha and DGCR8. Pre-miRNAs are exported by Exportin 5 to the cytoplasm where they are further processed by another RNase III, Dicer, to generate the ~22 nt mature miRNA products. It has long been thought that each arm of the hairpin precursor miRNA gives rise to a single mature product that is then loaded onto the Argonaute (Ago) silencing complex [7]. However, recent advances in NGS have revealed that multiple distinct mature miRNA species can arise from the same hairpin arm, termed isomiRs. These sequence variants typically differ from the mature miRNA sequences currently in public databases such as miRBase [8] at either their 5´ or 3´ ends thereby increasing the diversity and complexity of the miRNA-ome. While the biological relevance of isomiRs is not fully understood, they have been shown to associate with Ago [9], which in turn suggests a functional role.

Recent studies of isomiR expression have either focused on isomiRs of a single miRNA or on the isomiR expression patterns within a specific tissue. For example, a 5´-isomiR of miR-101 was observed to be ubiquitously expressed in several human tissues and cell lines [10]. Similarly, examination of isomiRs in peripheral blood mononuclear cells (PBMC) identified tissue specific isomiRs [11]. The use of next-generation sequencing in these studies enabled a preliminary isomiR characterization but the limited number of samples (four) prevented a systematic analysis. In an effort to better characterize isomiR space, we used the recently reported short RNA profiles from lymphoblastoid cell lines (LCL) of 452 healthy men and women belonging to five different populations: Utah Residents with Northern and Western European ancestry (CEU), Finnish from Finland (FIN), British from England and Scotland (GBR), Toscani Italians (TSI), and Yoruban Africans from the city of Ibadan (YRI) [12]. Summarily, our results show that LCLs exhibit a considerable diversity in the isomiRs that arise from any given miRNA arm and that there are isomiR expression differences across population and gender boundaries.

## RESULTS

In what follows, we will use the term isomiR to refer to the multitude of mature miRNA products that are produced from a given arm (e.g. the left arm or ‘5p’) of a given miRNA precursor (e.g. miR-155). Any two isomiRs will differ from one another at either their 5´ only, their 3´ end only, or at both ends simultaneously. Clearly, if the miRBase reference miRNA arising from a given precursor arm is expressed it will be part of the isomiR collection that is associated with the arm.

### Multiple isomiRs arise from miRNA precursor arms in LCLs

We analyzed all 452 unique datasets corresponding to 452 individuals (i.e. neither technical replicates nor population-dependent groups were considered in this step – see Materials and Methods) and identified 194 miRNA precursor arms each of which gave rise to one or more significantly expressed mature miRNAs (Supp. File 2). These 194 arms correspond to 153 miRNA precursors. For each precursor arm, we only consider those isomiRs that contribute to the 95^th^-quantile of the reads that arise from the arm: doing so, we find a total of 445 unique isomiRs (i.e. an average of 2.29 isomiRs per identified arm).

Could it be that the mature miRNA product variability that we observe within the span of a precursor arm is due to technical artifacts or random degradation? We assessed this possibility by examining isomiR expression in the sequenced technical replicates where five samples (one from each population) were sequenced a total of seven times each [12]. We used these replicates to determine the extent at which the expression of isomiRs remained consistent across datasets generated by the different sequencing centers. In particular, we calculated the Spearman and Pearson correlations for each pair of replicates following normalization and found the isomiR profiles to be remarkably consistent across the replicates (Supp. File 3 and Supp. File 4).

### The reference miRBase miRNA often differs from the most abundant isomiR in LCLs

**Figure 1:**
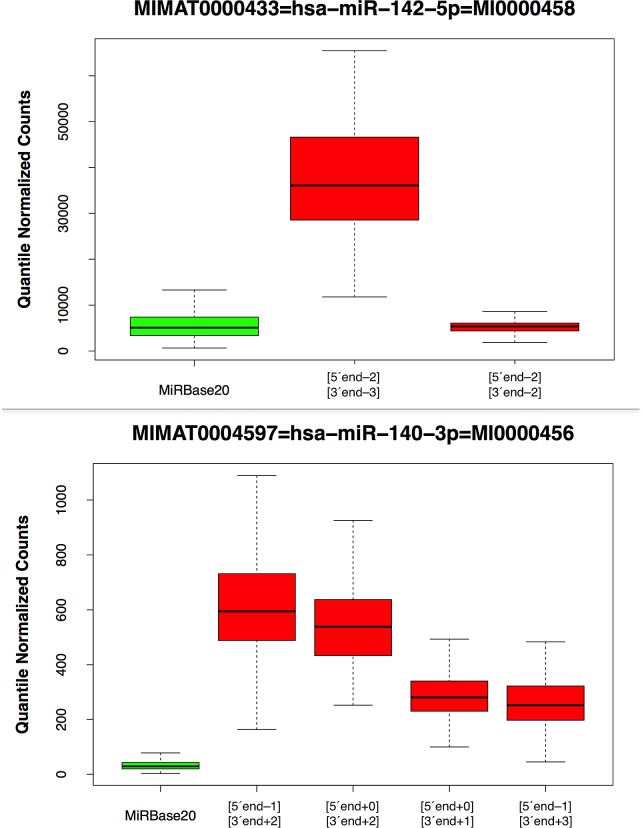
MiRBase and isomiR boxplots This Fig. shows boxplots for the miR-142-5p and miR-140-3p arms. The data was generated using the group of 452 unique samples (no technical replicates were included). The miRBase reference entry is shown in green whereas other detected isomiRs are shown in red. In the miR-142-5p case, the 5´ terminus of the first shown isomiR begins 2 positions before that of the miRBase reference whereas its 3´ terminus ends 3 positions before that of the miRBase reference. Analogously, in the miR-140-3p case, we identified 4 isomiRs whose 5´ and 3´ termini differ in various ways from those of the miRBase reference and whose abundance is consistently higher than the miRBase reference's.

Looking across all 452 analyzed datasets, we found that for 91 of the 194 precursor arms (46.9%) the most abundant isomiR at that arm differs from the corresponding miRBase reference miRNA (Table 1 and Supp. File 2). This most abundant isomiR differed from the miRBase entry at either the 3´ end only (79 of 91 cases), or the 5´ end only (3 of 91 cases), or both ends (9 of 91 cases). Fig. 1 shows two characteristic examples for the miR-142-5p and miR-140-3p arms respectively: in each case the miRBase reference variant is shown in green. For both arms, some of the abundant isomiRs differ in both the 5´ and 3´ ends from the miRBase reference. In Supp. Fig. 1, we show box-plots for all 194 miRNA precursor arms. The corresponding expression values are listed in Supp. File 2.

**Table 1:** Statistics across all populations for the single most abundant isomiR produced from each precursor arm The most abundant isomiR's identity was determined by analyzing the entire collection of all unique datasets that passed quality control (452 datasets, one for each of 452 individuals; no technical replicates were included). At each locus, we also generated the “*conservative*” estimate (4th row) by requiring that the most abundant isomiR have higher expression than the miRBase reference by at least 0.585 log2 units.

	Number	Percentage
MiRNA precursor arms considered	194	
MiRBase miRNA sequence not among the 95-th percentile	60	31%
Top isomiR not in agreement with miRBase	91	47%
Top isomiR (conservative) not in agreement with miRBase	84	43%
Top isomiR disagrees with miRBase at 5' end	12	6%
Top isomiR disagrees with miRBase at 5' end ***only***	3	2%
Top isomiR disagrees with miRBase at 3' end	88	45%
Top isomiR disagrees with miRBase at 3' end ***only***	79	41%
Top isomiR disagrees with miRBase at ***both ends***	9	5%

### IsomiRs from LCLs are loaded on Argonaute

As mentioned above, we found 445 isomiRs that are produced from the 194 identified miRNA precursor arms. To generate independent corroborating support for these isomiRs, we sought evidence of their loading in Argonaute silencing complexes. We used a publicly available deep-sequencing collection (GEO reference: GSE41437) that was generated from LCL samples [13] after performing photoactivatable ribonucleoside-enhanced crosslinking and Argonaute (Ago) immunoprecipitation (Ago PAR-CLIP). For this step, we worked with only the subset of sequenced reads that a) we could map on the genome unambiguously and exactly (i.e. no matching errors permitted), and b) whose endpoints from the Ago PAR-CLIP data matched perfectly the endpoints of the 445 identified isomiRs. Furthermore, we only consider an isomiR to be significantly loaded on Ago if the read support reached statistical significance (pVal ≤ 0.05; truncated negative binomial distribution fitting) for that isomiR in at least one of the Ago PAR-CLIP datasets. The truncated negative binomial distributions were generated individually for each of the Ago PAR-CLIP datasets because the required number of reads needed to reach statistical significance is dependent on the number of sequenced reads for the dataset and consequently differs across datasets. Of the 134 miRBase reference miRNAs that are present in the 452 analyzed datasets, 73 (54.5%) are supported by the Ago PAR-CLIP data at the chosen threshold of statistical significance. Repeating the calculation for the remaining 311 isomiRs that differ from the miRBase miRNA reference, we find PAR-CLIP support for 139 (44.7%) of them (Supp. File 10). In other words, the isomiRs that we have identified, whether they match the miRBase miRNA reference or not, receive similar levels of corroborating support by an independently generated Ago PAR-CLIP dataset as the known miRBase reference entries.

### Some isomiRs exhibit population-dependent expression profiles

**Figure 2:**
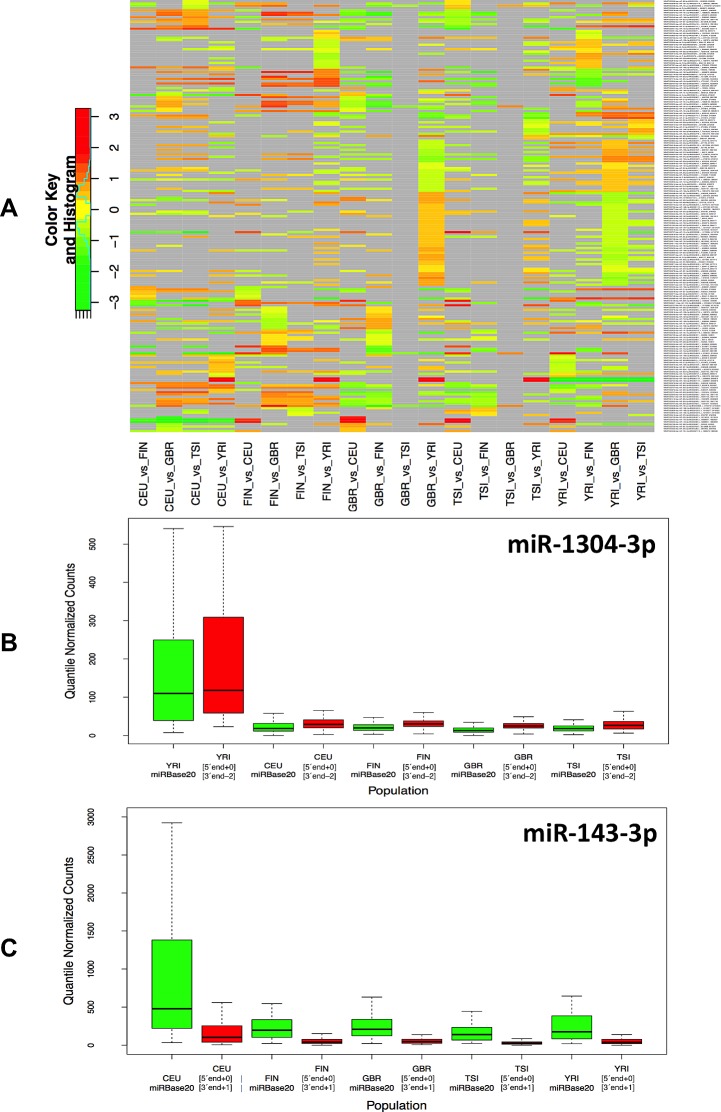
Differential expression of isomiRs across populations A: Using samples from both genders, we identified differentially expressed isomiRs separately for each pair of populations. This figure shows the resulting heat-map for the identified isomiRs. Cells shown in light gray correspond to cases that did not reach statistical significance for the corresponding isomiR/population combination. All other cells correspond to statistically significant combinations with an associated FDR value ≤ 0.05. The color intensity scale is logarithmic (log2 base). Note that the Fig. depicts redundant information in that all 5x4 population pairs are shown. B: This panel shows a boxplot for the miR-1304-3p arm within each of the five population groups (both genders): miR-1304-3p is more abundant in the YRI population. In particular, an isomiR variant that is 2 nts shorter on the 3´ end compared to the miRBase reference is also present in the YRI population and more abundant than the other variants. C: This panel shows a boxplot for the miR-143-3p arm within each of the five population groups (both genders): the reference miRBase entry and an isomiR variant that is longer on the 3´ end by 1 nt compared to the miRBase reference is more abundant in the CEU compared to the other four populations.

Not surprisingly, when considering the samples within and across population groups, we find that the five population groups show a very similar profile in terms of the numbers and endpoint characteristics (i.e. 5´ and/or 3´ differences) of isomiRs that are present in their respective samples (Table 2 and Supp. File 5). Moreover, and not surprisingly, the five populations show extensive overlap in the *identity* of isomiRs that are present in each group (Table 3 and Supp. File 5). However, when we examine the *expression* of the statistically significant isomiRs we find that many are differentially expressed across different population boundaries. As can be seen in Fig. 2A and Supp. File 6, the statistically significant isomiRs exhibit expression that in some instances changes by as much as 8-fold (3 log2 units) between the two examined populations. Several isomiRs stand out in these comparisons with the ones from the right arm of miR-1304 and the right arm of miR-143 being the most notable. The two isomiRs from the miR-1304-3p arm are between 5.6 and 9.5 times more abundant in the YRI group compared to the other four populations with associated false discovery rate (FDR) values for the pair-wise comparisons ranging between 2.06 e-08 and 3.02 e-10 (Fig. 2B and Supp. File 6). Similarly, the two isomiRs produced from the miR-143-3p arm are much more abundant in the CEU group compared to the other four populations (between 2.8 and 4.1 times) with associated FDR values for the four comparisons ranging between 1.66 e-02 and 5.18 e-03 (Fig. 2C and Supp. File 6A). Supp. Fig. 2 shows in detail the results of the pair-wise population comparisons. The heat-map of Fig. 2 makes it apparent that a fair number of isomiRs are differentially expressed between any two of the five populations considered here; generally, the “distinguishing” isomiRs are different for each of the 10 possible population pairs.

**Table 2:** Statistics for each population separately for the single most abundant isomiR produced from a given precursor arm The most abundant isomiR's identity was determined by analyzing the collection of all unique datasets that passed quality control and belonged to the population under consideration (a total of 452 datasets; no technical replicates were included). At each locus, we also generated the “*conservative*” estimate (4^th^ row) by requiring that the most abundant isomiR have higher expression than the miRBase reference by at least 0.585 log2 units.

	CEU		FIN		GBR		TSI		YRI	
	Number	Percentage	Number	Percentage	Number	Percentage	Number	Percentage	Number	Percentage
MiRNA precursor arms considered	196		185		188		185		197	
MiRBase miRNA sequence not among the 95-th percentile	61	31%	54	29%	57	30%	55	30%	59	30%
Top isomiR not in agreement with miRBase	95	48%	87	47%	92	49%	90	49%	93	47%
Top isomiR (conservative) not in agreement with miRBase	85	43%	79	43%	84	45%	81	44%	83	42%
Top isomiR disagrees with miRBase at 5' end	13	7%	11	6%	10	5%	10	5%	11	6%
Top isomiR disagrees with miRBase at 5' end ***only***	2	1%	3	2%	3	2%	2	1%	2	1%
Top isomiR disagrees with miRBase at 3' end	93	47%	84	45%	89	47%	88	48%	91	46%
Top isomiR disagrees with miRBase at 3' end ***only***	82	42%	76	41%	82	44%	80	43%	82	42%
Top isomiR disagrees with miRBase at ***both ends***	11	6%	8	4%	7	4%	8	4%	9	5%

### Some isomiRs exhibit gender-dependent expression profiles

**Figure 3:**
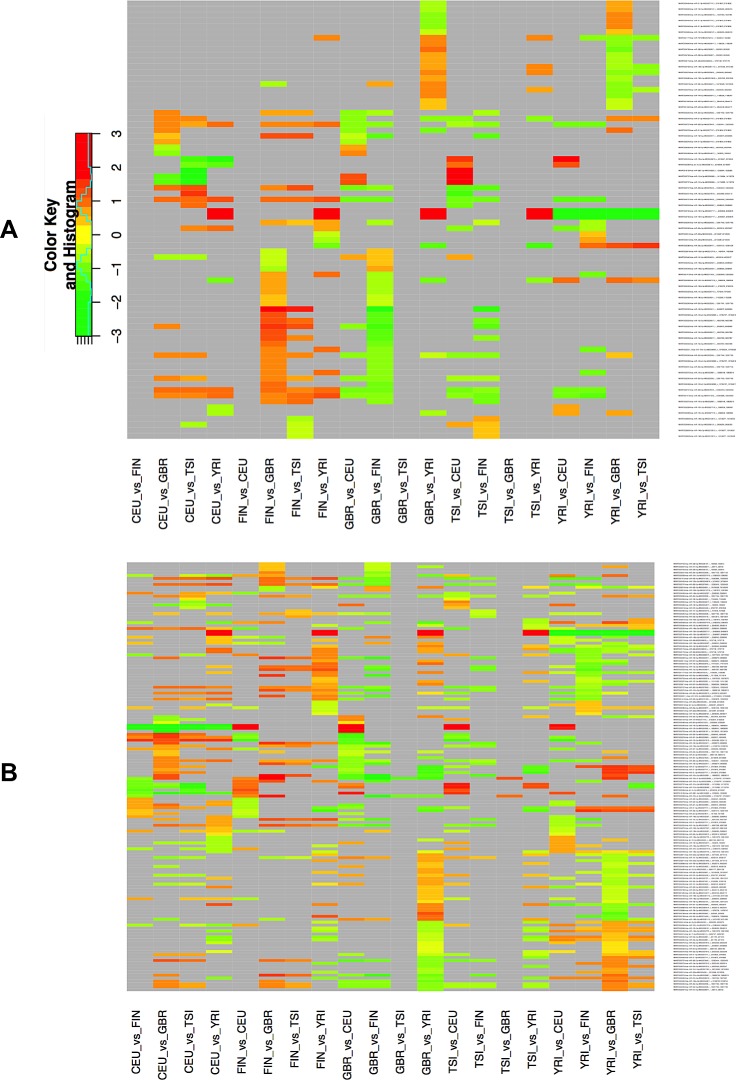
Differential expression of isomiRs across populations by gender Using samples of the same gender, we identified differentially expressed isomiRs separately for each pair of populations. This figure shows the resulting heat-map for the identified isomiRs. Cells shown in light gray correspond to cases that did not reach statistical significance for the corresponding isomiR/population combination. All other cells correspond to statistically significant combinations with an associated FDR value ≤ 0.05. The color intensity scale is logarithmic (log2 base). A: Result from processing only the male samples. B: Result from processing only the female samples. Note that each panel depicts redundant information in that all 5x4 population pairs are shown.

Given that some isomiRs exhibit expression profiles that depend on the population, we hypothesized that some isomiRs may exhibit gender-dependent differences. To address this question, we examined single gender isomiR expression across populations. Fig. 3A shows a heat-map of the results when only the expression profiles among the male members of each population are analyzed: there are 76 unique isomiRs whose expression levels show population-dependent differences that are statistically significant (FDR ≤ 0.05). Of these 76 isomiRs, 68 (of which only 22 match a miRBase reference miRNA) exhibit a log2 fold change of 0.585 or higher. The complete list of isomiRs together with expression changes among populations and the associated FDR values is shown in Supp. File 6B-C. Fig. 3B shows an analogous heat-map this time analyzing the expression profiles of only the female members of each population: the expression levels for 146 unique isomiRs show population-dependent differences that are statistically significant (FDR ≤ 0.05). Of these 146 isomiRs, 120 (of which only 34 match a miRBase reference miRNA) exhibit a log2 fold change of 0.585 or higher. As can be seen from Fig. 3B and Supp. File 6C, the miR-143-3p precursor arm (see above) is more abundant in CEU females (log2 fold change of 1.75 or higher with an associated FDR value ranging from 1.89 e-03 to 9.00 e-03) compared to the other four populations. However, and somewhat surprisingly, no isomiRs from the miR-143-3p arm show differential expression in any males-vs.-males population comparisons. On the other hand, the isomiRs from the miR-1304-3p arm are more abundant in YRI compared to the other four populations in both males-vs.-males and females-vs.-females comparisons (log2 fold change of 2.20 or higher with an associated FDR value ranging from 8.10 e-03 to 2.70 e-07). Supp. Fig. 3 (males) and Supp. Fig. 4 (females) show the heat-maps for these population pairs in more detail. Details regarding the isomiRs within a population are listed in Supp. File 7 (males) and Supp. File 8 (females). Additional examples of precursor arms that give rise to differentially expressed isomiRs include: miR-34a-5p (isomiRs are less abundant in YRI compared to the other four populations in only females-vs.-females comparisons; miR-221-3p and miR-222-3p (isomiRs from both loci are less abundant in CEU compared to the other four populations in only females-vs.-females comparisons), and other. Fig. 3 and Supp. File 6 make it evident that when comparing any two population groups there are many more isomiRs that are differentially expressed (and statistically significant) among females than are among males, an unexpected finding. Interestingly, and for all five populations, when we compared males vs. females *within a population* we did not find any isomiRs whose differential expression reached statistical significance.

### IsomiRs exhibit more variation in their 3´ ends

**Figure 4:**
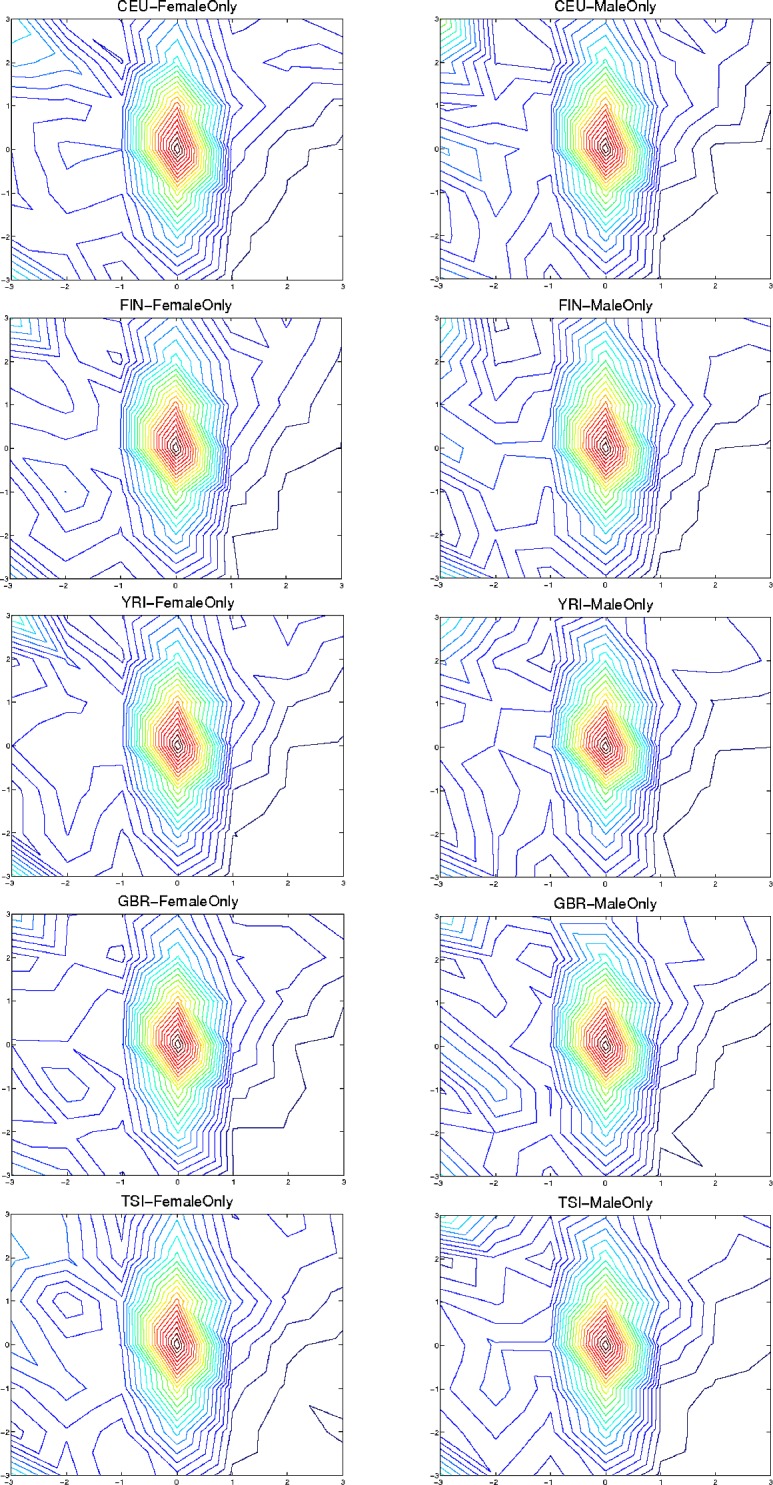
Distribution of termini combination separately for each population and gender combination Each isomiR is mapped to an (X, Y) point based on how its termini differ from those of the miRBase reference: negative (respectively positive) values indicate that the isomiR terminus is positioned to the left (respectively to the right) in the 5´→3´ direction of the corresponding terminus of the miRBase reference. The X-axis shows differences between the 5´ terminus of the isomiR compared to 5´ terminus of the corresponding miRBase entry. Analogously, the Y-axis shows differences between the 3´ terminus of the isomiR compared to the 3´ terminus of the corresponding miRBase entry. Within an arm, each isomiR's (X, Y) point has an associated height Z, whose value is between 0 and 1 and captures the isomiR's portion of the contribution to the reads that arise from the arm. The Figure shows the contours of the mesh formed by these points and separately for each of the 10 gender/population pairs: most of the isomiRs differ by +/- 1 nt at either the 5´ terminus or 3´ terminus, or both (see also Supp. Figure 5).

In light of the fact that frequently the miRBase entry does not correspond to the most abundant variant from the corresponding arm, we sought to determine whether the observed isomiR variants exhibit specific preferences in their 5´ or 3´ termini. To this end, we examined how the isomiRs' termini are distributed relative to those of the corresponding miRBase entries (Fig. 4 and Supp. Figure 5). Of the 445 unique isomiRs that we identified (Supp. File 2) 296 (67%) show 3´ changes whereas only 39 (9%) show 5´ changes. Fig. 4 shows the contours of the resulting distributions while Supp. Figure 5 provides count and distribution information separately for the 5´ and 3´ terminus. Generally, the 5´ ends show a narrower range (+/-1 nt) of modifications compared to the 3´ ends (+/-3 nts) – see Supp. Figure 5. As can also be seen from Fig. 4, the distribution of isomiR types (5´ differences with respect to the miRBase reference, 3´ differences with respect to the miRBase reference, etc.) remains consistent across the five population groups. The findings indicate that the majority of the diversity in the observed isomiRs is the result of a concomitant diversity in the 3´ termini of the isomiRs.

### GO term analysis for miR-1304-3p and miR-143-3p

We took a closer look at the two miRNA precursor arms, miR-1304-3p (YRI vs. each of the other four groups) and miR-143-3p (CEU vs. each of the other four groups) that the above analysis highlighted as being most highly differentially expressed across population pairs. For the isomiRs arising from these two arms we used rna22 (see Materials and Methods) to predict targets among the 8,501 mRNAs that are expressed across the 452 analyzed datasets and whose RPKM value is ≥ 1/1024 of the RPKM for ACTB, i.e. 10 PCR cycles away from ACTB (data obtained from the Geuvadis project website http://www.ebi.ac.uk/arrayexpress/files/E-GEUV-1/analysis_results/). There are 2,355 predicted targets for miR-1304-3p isomiRs and 2,358 predicted targets for miR-143-3p isomiRs. The complete list of results is shown in Supp. File 9. We generated GO terms for these targets using DAVID [14, 15] and clustered them using REVIGO [16]. We only considered terms that per DAVID analysis had an associated FDR value ≤ 0.05 and a fold enrichment ≥ 2.0. Summarily, among the predicted targets for miR-143-3p we find a strong enrichment for genes that participate in ribonucleoprotein complex biogenesis and mRNA splicing. Among molecular functions, enriched GO terms include helicase activity and nuclear hormone receptor binding. Among the pathways whose component genes were enriched among the predicted targets we find the one for ‘ubiquitin-mediated proteolysis.’ Analogously, for miR-1304-3p we find a strong enrichment for genes that participate in DNA replication, ribonucleoprotein complex biogenesis, mRNA metabolism and catabolism, RNA splicing, response to DNA damage etc. Among molecular functions, enriched GO terms include chromatic binding and helicase activity. Finally among the pathways whose component genes were enriched among the predicted targets we find the one for ‘spliceosome.’ As with miR-143-3p, we only considered terms that per DAVID analysis had an associated FDR value ≤ 0.05 and a fold enrichment ≥ 2.0.

## DISCUSSION

For a long time, miRNA research proceeded under the assumption that each arm of a miRNA precursor gives rise to a single mature miRNA [1, 7]. In recent years, the advent of next generation sequencing permitted the in-depth exploration of the transcriptomes from many cell types. Some of the findings that emerged from these early studies began to challenge the notion of “one mature miRNA per precursor arm” [17]. However, the various analyses to date examined collections with relatively small numbers of samples thus making it difficult to draw general conclusions. The recent release of RNA-sequencing data from the GEUVADIS project for 452 individuals presented us with an unprecedented opportunity to study the isomiR question in more depth. What makes this sample collection particularly appealing is that it comprises samples from a single cell type with both genders and five different human populations represented evenly.

The studies we were able to carry out add further strength to the emerging view that the concept of “mature miRNA” cannot be associated with a single sequence. Instead, as we find, multiple distinct mature miRNA products can arise from the same arm of a miRNA precursor (Fig. 1 and Supp. Figure 1). These ‘variants’ are not random degradation products or artifacts of the RNA sequencing step, but rather the result of apparently marshaled processing. This is evidenced by several observations: a) the isomiRs from a given precursor arm are the same, in terms of both composition and relative abundance across technical replicates from the same sample (Supp. File 3 and Supp. File 4); b) the isomiRs from a given precursor arm remain consistent across *different* population groups (Table 3) and across same-gender individuals that belong to different populations (Supp. File 7 and Supp. File 8); c) the isomiRs from a given precursor arm also remain consistent between males and females that belong to the *same* population group (Supp. File 7 and Supp. File 8); lastly, d) many of the isomiRs we identified are also found to be part of the Ago silencing complex in Ago PAR-CLIP data that were independently generated from LCLs by a different group (Supp. File 10).

**Table 3:** IsomiRs are shared across populations even when we examine each gender separately On the left table we show the actual raw numbers of identified isomiRs that are present within a population group and shared across population groups. On the right side, we show the corresponding Jaccard coefficient for the various pair-wise population group comparisons.

**Both Genders - isomiR sharing across populations**					
	**YRI**	**CEU**	**FIN**	**GBR**	**TSI**
**YRI**	449	422	407	407	406
**CEU**	422	450	415	407	410
**FIN**	407	415	427	406	413
**GBR**	407	407	406	436	421
**TSI**	406	410	413	421	431
**Males Only - isomiR sharing across populations**					
	**YRI**	**CEU**	**FIN**	**GBR**	**TSI**
**YRI**	452	422	411	409	397
**CEU**	422	453	417	421	399
**FIN**	411	417	436	415	406
**GBR**	409	421	415	444	411
**TSI**	397	399	406	411	424
**Females Only - isomiR sharing across populations**					
	**YRI**	**CEU**	**FIN**	**GBR**	**TSI**
**YRI**	446	410	399	398	408
**CEU**	410	438	408	399	405
**FIN**	399	408	424	402	410
**GBR**	398	399	402	431	415
**TSI**	408	405	410	415	437

**Both Genders – Jaccard coefficient for isomiR groups across populations**					
	**YRI**	**CEU**	**FIN**	**GBR**	**TSI**
**YRI**	1.00	0.88	0.87	0.85	0.86
**CEU**	0.88	1.00	0.90	0.85	0.87
**FIN**	0.87	0.90	1.00	0.89	0.93
**GBR**	0.85	0.85	0.89	1.00	0.94
**TSI**	0.86	0.87	0.93	0.94	1.00
**Males Only – Jaccard coefficient for isomiR groups across populations**					
	**YRI**	**CEU**	**FIN**	**GBR**	**TSI**
**YRI**	1.00	0.87	0.86	0.84	0.83
**CEU**	0.87	1.00	0.88	0.88	0.83
**FIN**	0.86	0.88	1.00	0.89	0.89
**GBR**	0.84	0.88	0.89	1.00	0.90
**TSI**	0.83	0.83	0.89	0.90	1.00
**Females Only – Jaccard coefficient for isomiR groups across populations**					
	**YRI**	**CEU**	**FIN**	**GBR**	**TSI**
**YRI**	1.00	0.86	0.85	0.83	0.86
**CEU**	0.86	1.00	0.90	0.85	0.86
**FIN**	0.85	0.90	1.00	0.89	0.91
**GBR**	0.83	0.85	0.89	1.00	0.92
**TSI**	0.86	0.86	0.91	0.92	1.00
Color Code:	0.00	0.25	0.50	0.75	1.00

Another and perhaps somewhat surprising observation relates to our finding that the miRBase reference entry is the most abundant variant among the mature products from a given precursor arm only ~53% of the time (Table 1). Additionally, we find that the majority of the produced variants result from differences in the 3´ terminus of the isomiR: in some isomiRs the terminus occurs before the terminus of the miRBase reference entry (in the 5´➔3´ direction) whereas in other it occurs after (Supp. Figure 1). In fact, the majority of the isomiR termini occur within a 5-nt window centered at the 3´ terminus of the miRBase entry. By comparison the 5´ termini of the isomiRs show less variability with the majority of them occurring within a 3-nt window centered at the 5´ terminus of the miRBase entry (Fig. 4 and Supp. Figure 5). It is however possible that these observations are specific to the LCLs, and may not be mirrored in future studies involving other tissues and cell types.

The samples we analyzed represent five distinct population groups; in turn, this allowed us to also examine the possibility of differences in isomiR expression across populations. Indeed, we found a few miRNAs to be statistically up-regulated in a single population compared to the remaining four populations (Fig. 2 and Fig. 3). In particular, isomiRs from the miR-1304-3p precursor arm, a locus previously associated with regulating enamel formation in Neanderthals [18], were significantly up-regulated in the YRI population (in both males and females) whereas isomiRs from the miR-143-3p precursor arm were significantly up-regulated in the CEU population (but only in females). We generated target predictions separately for each isomiR from each of these arms and analyzed them with DAVID and REVIGO (Supp. File 9) and found that the predicted targets were enriched in several GO categories that among other included: helicase activity, nuclear hormone receptor binding, ribonucleoprotein complex biogenesis, mRNA splicing, DNA replication, response to DNA damage, etc. Even though the associated FDR values strongly argue for the findings' statistical significance, the biological implications of the categories and pathways that emerge from this analysis is not currently understood. As these two miRNAs were population-specific and gender-specific respectively, there is the possibility that they may underlie various aspects of disease (e.g. differences in disease onset triggers, differences in disease progression, differences in response to specific therapies, etc.). For example, while the incidence of chronic lymphocytic leukemia is lower in African Americans than Caucasians, African Americans are more than twice as likely to die from the disease [19].

When we compared the levels of expression across population groups separately for each gender, we found many more isomiRs among females that were differentially expressed between population groups than among males. This result holds true for all pair-wise population group comparisons. The observed difference suggests that more of the contribution to the differential expression signal that is observed between populations is contributed by the female members of the population.

A particular hallmark of miRNAs is their association with the Ago silencing complex that mediates the effect on their targets. Even though multiple mature products are expressed from a given miRNA precursor arm it is not clear how many of them are actually loaded onto the silencing complex. To investigate this we searched Ago PAR-CLIP datasets for the presence of the isomiRs we identified by analyzing these 452 datasets. As the Ago PAR-CLIP datasets were also from LCLs, it is reasonable to assume that they would have a similar isomiR expression profile, but that they may not necessarily express all of the isomiRs that we identified here. Our findings show that miRBase reference entries as well as the newly identified isomiRs are represented at roughly equal levels in the Ago PAR-CLIP data (Supp. File 10), which in turn suggests that in addition to the miRBase reference the isomiR variants can also be functionally active. One possible implication of this observation could be that the different isomiRs from the same precursor arm can work cooperatively to repress target genes or that they have slightly different targeting profiles that permit an increased diversity of the targeted transcripts [10, 20-22].

The samples we analyzed were sequenced in the context of the GEUVADIS project [12] and were originally collected as part of the HapMap [23] and 1000 genomes projects [24]. Many of the samples have been in culture for many years (CEU and YRI are the oldest followed by TSI; FIN and GBR are most recent – see http://geuvadiswiki.crg.es/index.php). With that in mind one might argue that the findings regarding the isomiRs from the miR-1304-3p (YRI-vs.-others) and miR-143-3p (CEU-vs.-others) precursor arms are artifacts due to cell line age. We explain next why we believe this to not be the case.

For argument's sake let us assume for the moment that the miRNA profile was affected in a tangible manner by the age of the CEU and YRI samples. It is reasonable to assume that such an effect would be systemic in nature impacting uniformly both the males and females of the CEU and YRI populations and many, if not all, of the expressed (protein-coding and non-coding) transcripts. This would mean that any would-be-systemically-affected miRNAs should be identically present in both the male-vs.-male and female-vs.-female population group comparisons involving the CEU and YRI. However, the pair-wise comparisons for males-vs.-males (Supp. Figure 3 and Supp. File 7) and females-vs.-females (Supp. Fig. 4 and Supp. File 8) demonstrate that with regard to the miRNAs and isomiRs such a system-level impact is not supported by the data. Indeed, the isomiRs from the miR-143-3p (CEU) and miR-1304-3p (YRI) arms are the only miRNAs distinguishing the older CEU and YRI samples from the remaining three younger FIN, GBR and TSI samples. Moreover, these two miRNAs exhibit differential expression when we compare the (older) CEU samples with the (older) YRI samples: had these two miRNAs been the result of cell line age they would not have been differentially expressed in the (older) samples of these two populations. Lastly, we stress that miR-143-3p is differentially expressed only in the females-vs.-females comparisons across population groups: had this been the result of sample age it should have been exhibited by both genders in CEU, but this is not the case. On a related note, it is worth noting that the original 492 samples were randomly distributed, cultured and processed at seven different European laboratories, a procedure that has quenched any signal that might have been contributed by random processes introduced during RNA extraction, RNA library preparation and sequencing. Taken together, the above observations argue against the possibility that these two differentially expressed miRNAs are the result of either the older age of the CEU and YRI samples, or of a processed or deep-sequencing artifact.

In summary, our analyses indicate that the concept of a mature miRNA product needs to be expanded to accommodate the findings that a single precursor arm gives rise to more products than the single reference sequence that is currently listed in miRBase. These isomiR products are frequently more abundant than the miRBase reference entry. In terms of their composition, the isomiRs are produced in a consistent manner within and across genders, and within and across population groups. However, their relative expression levels are found to differ across gender and population boundaries. Naturally, in the absence of additional systematic analyses it is unclear how many and which ones of the similarities and differences we observed among the LCLs' isomiRs will carry over to other tissues and cell types. In fact, it is conceivable that additional factors define the exact isomiR population from a given precursor arm and it could well be the case that different expression rules govern a given arm across different cell types.

## METHODS

### Samples analyzed

From the Geuvadis RNA sequencing project (http://www.geuvadis.org/web/geuvadis/RNAseq-project) [12] we obtained the deep sequencing data for the 492 short RNA LCL samples. Among the 492 samples were 10 that did not pass quality control checks [12] and were removed from further consideration. Of the 482 samples, 452 are *unique* samples and represent five populations as follows: Utah Residents with Northern and Western European ancestry (CEU, 87 subjects), Finnish from Finland (FIN, 93 subjects), British in England and Scotland (GBR, 94 subjects), Toscani Italians (TSI, 89 subjects), and Yoruban Africans from the city of Ibadan (YRI, 89 subjects). The remaining 30 samples correspond to technical sequencing replicates comprising six *additional* sequencing runs for one sample from each of the five population groups (1 × 5 × 6 = 30).

### Sequence read mapping

Prior to mapping, adapters were removed and quality trimming was performed using the cutadapt tool [25] as we previously described [26, 27]. The sequenced reads were mapped, using Shrimp2 [28], to the GRCh37 (hg19) reference genome allowing a mismatch rate of no more than 4% per the reads length, and no insertion or deletions. Only reads that were at least 16 nucleotides (nts) in length and mapped unambiguously to the genome were kept and used in our analyses: by doing so we ensure that we can unambiguously assign read counts to those genomic loci to which reads can be mapped. Details on the mapping statistics can be found in Supp. File 1.

### Identification of isomiRs

We extracted the genomic location of each reference mature miRNA contained in Rel. 20 of miRBase [29] and flanked it by six nts on each side. Only reads that were wholly contained within this wider window were treated as belonging to a miRNA precursor arm and considered further. Distinct reads that had identical 5´ and 3´ endpoints within a miRNA arm were combined into a single isomiR: the union of distinct categories that we formed at each miRNA arm comprised the collection of isomiRs arising from the arm.

### Description of our isomiR notation

To facilitate our labeling of the various isomiRs from a given precursor arm, e.g. miR-142-5p, we use a notation that uses the endpoints of the corresponding miRBase entry as a reference. If the 5´ or the 3´ terminus of the isomiR is to the left (i.e. upstream in 5´➔3´ direction) of the miRBase entry's corresponding terminus, then we use a negative sign (–) to indicate this. On the other hand, we use a positive sign (+) to indicate that the 5´ or the 3´ terminus of the isomiR is to the right (in the the 5´➔3´ direction) of the corresponding miRBase terminus. A number following the sign shows how many nucleotides away the isomiR's terminus is with regard to the miRBase entry's terminus. For example: [5´end–1][3´end+2] denotes an isomiR whose 5´ terminus begins one nucleotide to the left of the miRBase entry's 5´ terminus and ends two nucleotides to the right of the miRBase entry's 5´ terminus.

### Quantification of isomiR expression

The 482 samples were divided into the following groups: a) one subgroup comprised 452 samples and excluded all technical replicates; b) five subgroups each comprising a single sample from each of the five populations (YRI, CEU, FIN, GBR, and TSI) and its respective six technical replicates (35 samples in total); c) five subgroups each comprising all the samples (excluding the technical replicates) from each of the five populations at hand; and, lastly, d) ten subgroups per human population separated by gender and excluding the technical replicates (5 subgroups for male-only and 5 subgroups for female-only representing a grand total of 452 sets).

The number of uniquely mapped sequence reads across the 482 LCL samples spans a wide range (~2 to ~50 million reads). In order to enable comparisons of relative isomiR frequency across samples of such varying sequencing depth, we used R to perform quantile normalization [30] across the samples after passing to the module the expression levels of each isomiR. For each miRNA precursor arm, we kept only those isomiRs that a) were in an arm where the top-isomiR had a normalized average of at least 25 reads per sample; and, b) the individual isomiR was within the 95th-quantile of reads for that arm; if no isomiRs of an arm satisfied these conditions, we discarded the arm. For each isomiR we kept we calculated a P-value using a one-sample t-test. This abundance-based thresholding is stringent and leaves us with approximately one third of the 644 miRNA arms described in [12] for this dataset.

### Differential Expression

To evaluate whether isomiRs are differentially expressed across the various subgroups we used DESeq [31]. For each comparison, we adjusted for multiple-testing using the Benjamini and Hochberg procedure and only considered an isomiR to be differentially expressed if the associated false discovery rate (FDR) was ≤ 0.05.

### Prediction and functional analysis of miRNA targets

MiRNA targets were predicted using the RNA22 algorithm [32] – see also http://cm.jefferson.edu/rna22v2/ for an interactively accessible implementation – and targets were allowed to be present in the 5´UTR, CDS, and 3´UTR of the candidate mRNA. The predicted targets were analyzed further using DAVID [14, 15] and predicted gene ontology (GO) terms were clustered with REVIGO [16] to determine possible enrichment of GO terms among each miRNA's predicted targets.

## SUPPLEMENTARY FIGURES AND METHODS






















