# Supplementary material for: IsomiR expression profiles in human lymphoblastoid cell lines exhibit population and gender dependencies
Source: Oncotarget. 2014 Aug 27;5(18):8790–802. doi: 10.18632/oncotarget.2405 (PMC4226722; doi:10.18632/oncotarget.2405)
Supplement: Supplementary file 1 [file oncotarget-05-8790-s001.pdf]

Supp. Figure 1 (pages 1-194). isomiR boxplots. Shown are boxplots for all 194 miRBase miRNAs and their associated isomiRs. For each plot, the Y-axis is the quantile normalized sequence read counts for each isomiR. The miRBase reference is green and the isomiRs are red.

Supp. Figure 2 (pages 195-199). Differentially expressed isomiRs for each population. Shown are individual heatmaps for each of the five different population groups examined. Plotted are the population comparisons (columns) and the rows are the different isomiRs. Cells shown in light gray correspond to cases that did not reach statistical significance for the corresponding isomiR/population combination. All other cells correspond to statistically significant combinations with an associated FDR value ≤ 0.05. The color intensity scale is logarithmic (log2).

Supp. Figure 3 (pages 199-204). Differentially expressed isomiRs for males only. Shown are individual heatmaps for each of the male subjects from the five different population groups examined. Plotted are the population comparisons (columns) and the rows are the different isomiRs. Cells shown in light gray correspond to cases that did not reach statistical significance for the corresponding isomiR/population combination. All other cells correspond to statistically significant combinations with an associated FDR value ≤ 0.05. The color intensity scale is logarithmic (log2).

Supp. Figure 4 (pages 205-209). Differentially expressed isomiRs for females only. Shown are individual heatmaps for each of the female subjects from the five different population groups examined. Plotted are the population comparisons (columns) and the rows are the different isomiRs. Cells shown in light gray correspond to cases that did not reach statistical significance for the corresponding isomiR/population combination. All other cells correspond to statistically significant combinations with an associated FDR value ≤ 0.05. The color intensity scale is logarithmic (log2).

Supp. Figure 5 (page 210). Distribution of isomiR termini. Shown is a schematic representation of the distribution of 5´ and 3´ terminus of the isomiRs. The 22 nts of the miRBase reference entry is shown in blue with each box and each box extending past represents an additional nucleotide position. The graphs show the distribution of bases at each nucleotide position either extending past the miRBase reference terminus or before the terminus.

Supp. Table 1 (page 211). Statistics for each population separately for the single most abundant isomiR produced from a given precursor arm. The most abundant isomiR's identity was determined by analyzing the technical replicates separately for each of the populations. At each locus, we also generated the "conservative" estimate by requiring that the most abundant isomiR have higher expression than the miRBase reference by at least 0.585 log2 units.

Supp. Table 2 (page 212). Statistics for each population separately for the single most abundant isomiR produced from a given precursor arm – using only females. The most abundant isomiR's identity was determined by analyzing the entire collection of all unique datasets that passed quality control and belonged to females. At each locus, we also generated the "conservative" estimate by requiring that the most abundant isomiR have higher expression than the miRBase reference by at least 0.585 log2 units.

Supp. Table 3 (page 213). Statistics for each population separately for the single most abundant isomiR produced from a given precursor arm – using only males. The most abundant isomiR's identity was determined by analyzing the entire collection of all unique datasets that passed quality control and belonged to males. At each locus, we also generated the "conservative" estimate by requiring that the most abundant isomiR have higher expression than the miRBase reference by at least 0.585 log2 units.

Supp. Figure 1
(pages 1 to 194)

MIMAT0000440=hsa−miR−191−5p=MI0000465

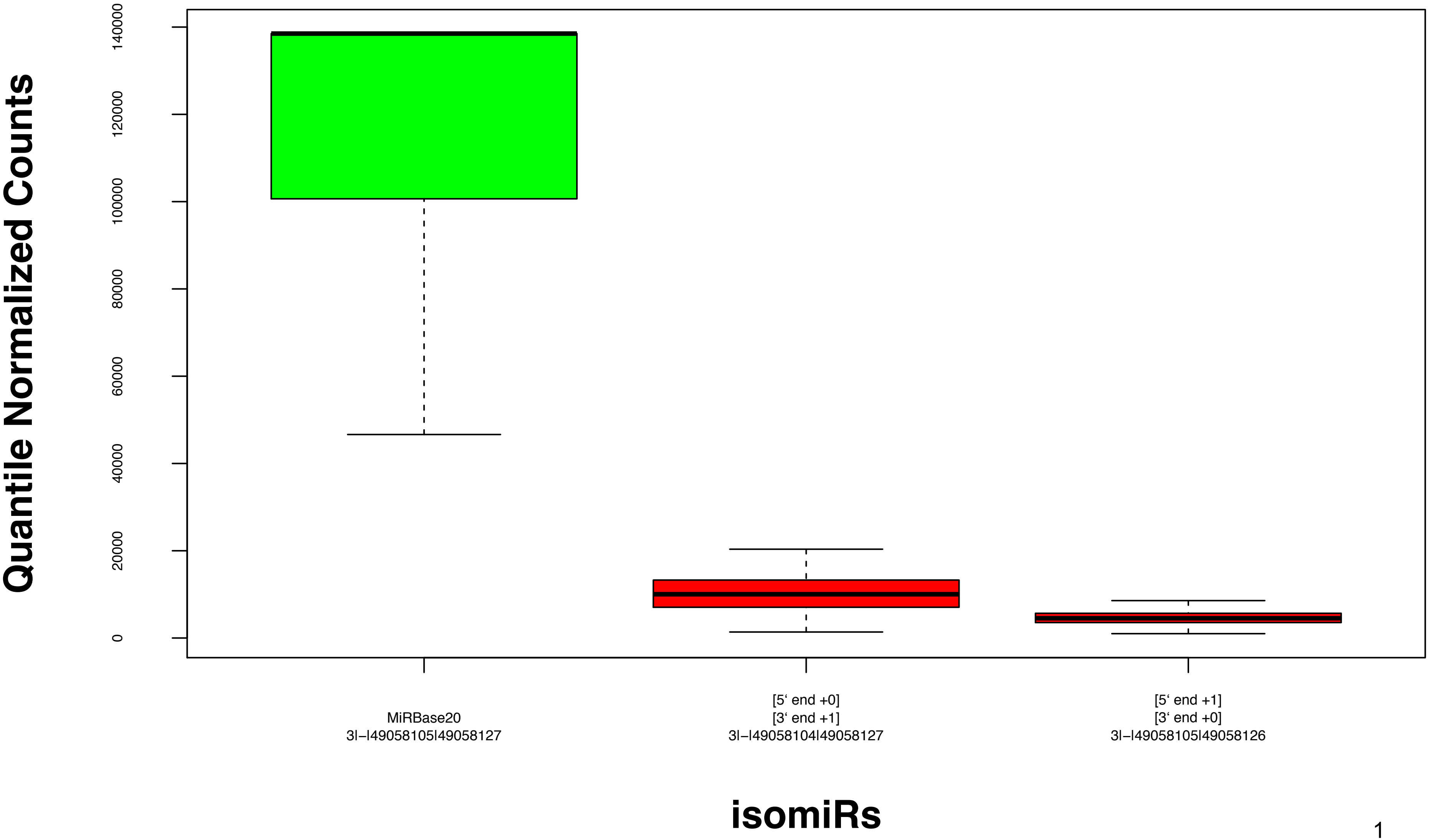

Supp. Figure 1
(pages 1 to 194)

MIMAT0000646=hsa−miR−155−5p=MI0000681

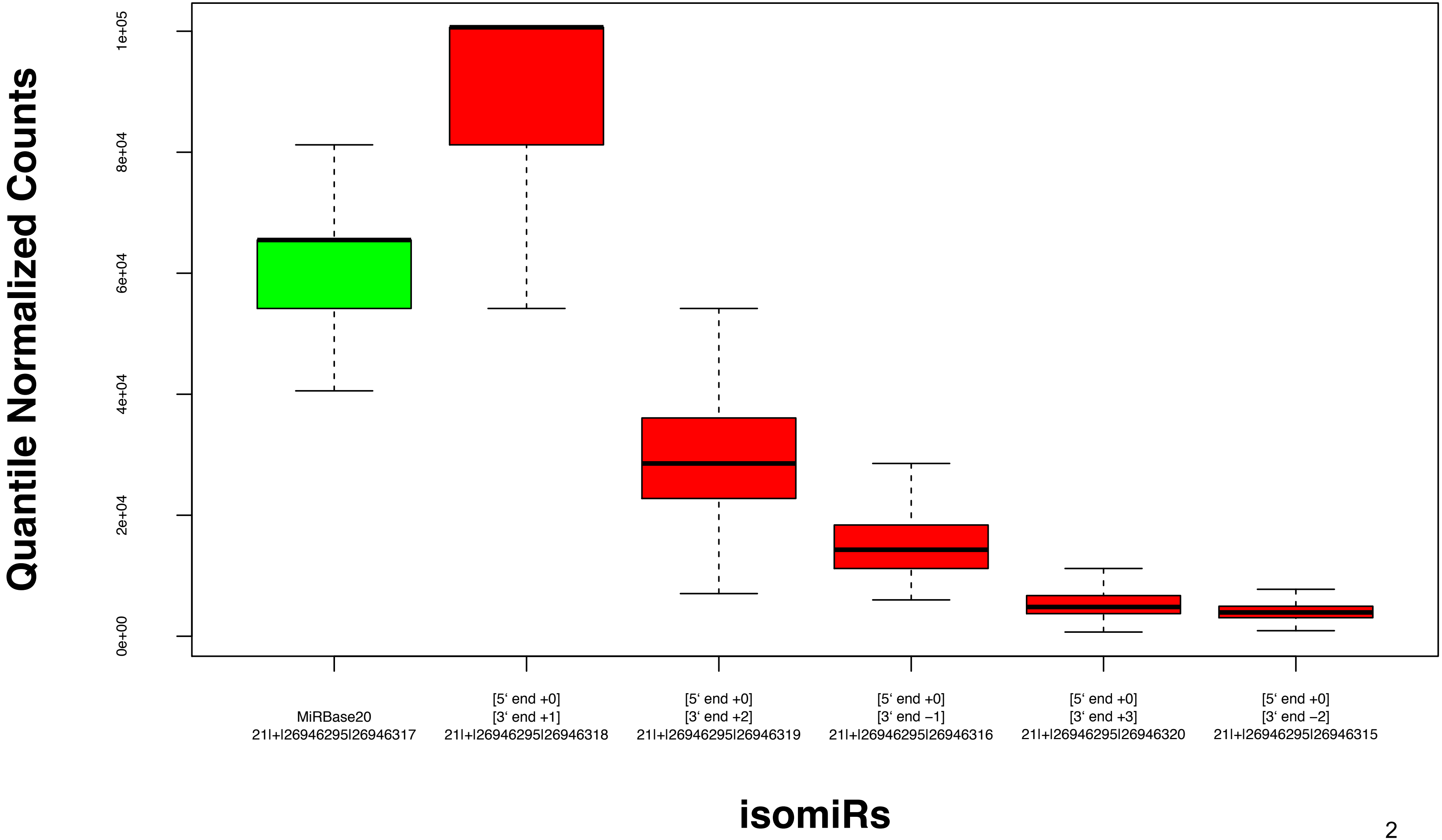

Supp. Figure 1
(pages 1 to 194)

MIMAT0000449=hsa–miR–146a–5p=MI0000477

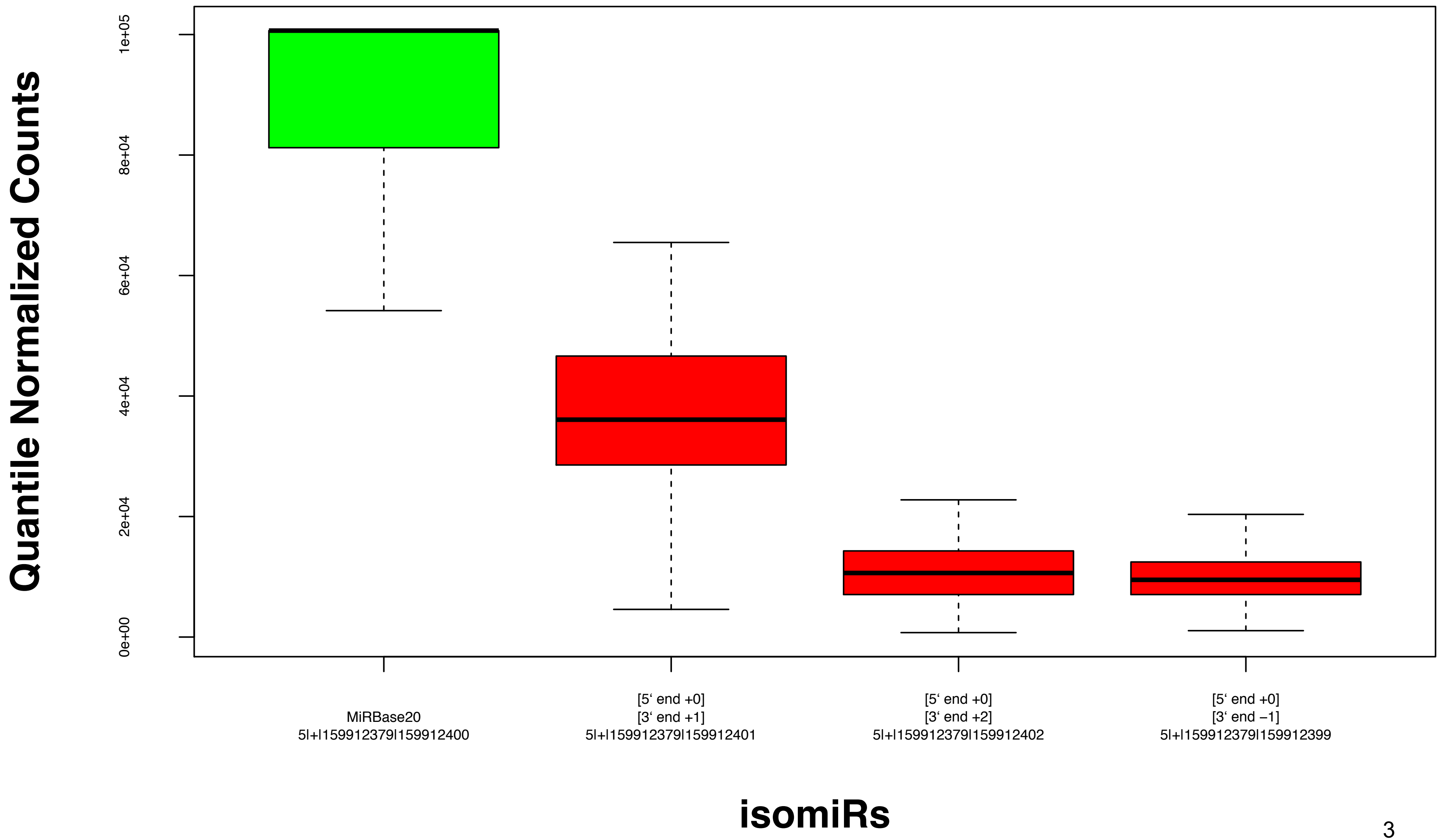

Supp. Figure 1
(pages 1 to 194)

MIMAT0000243=hsa–miR–148a–3p=MI0000253

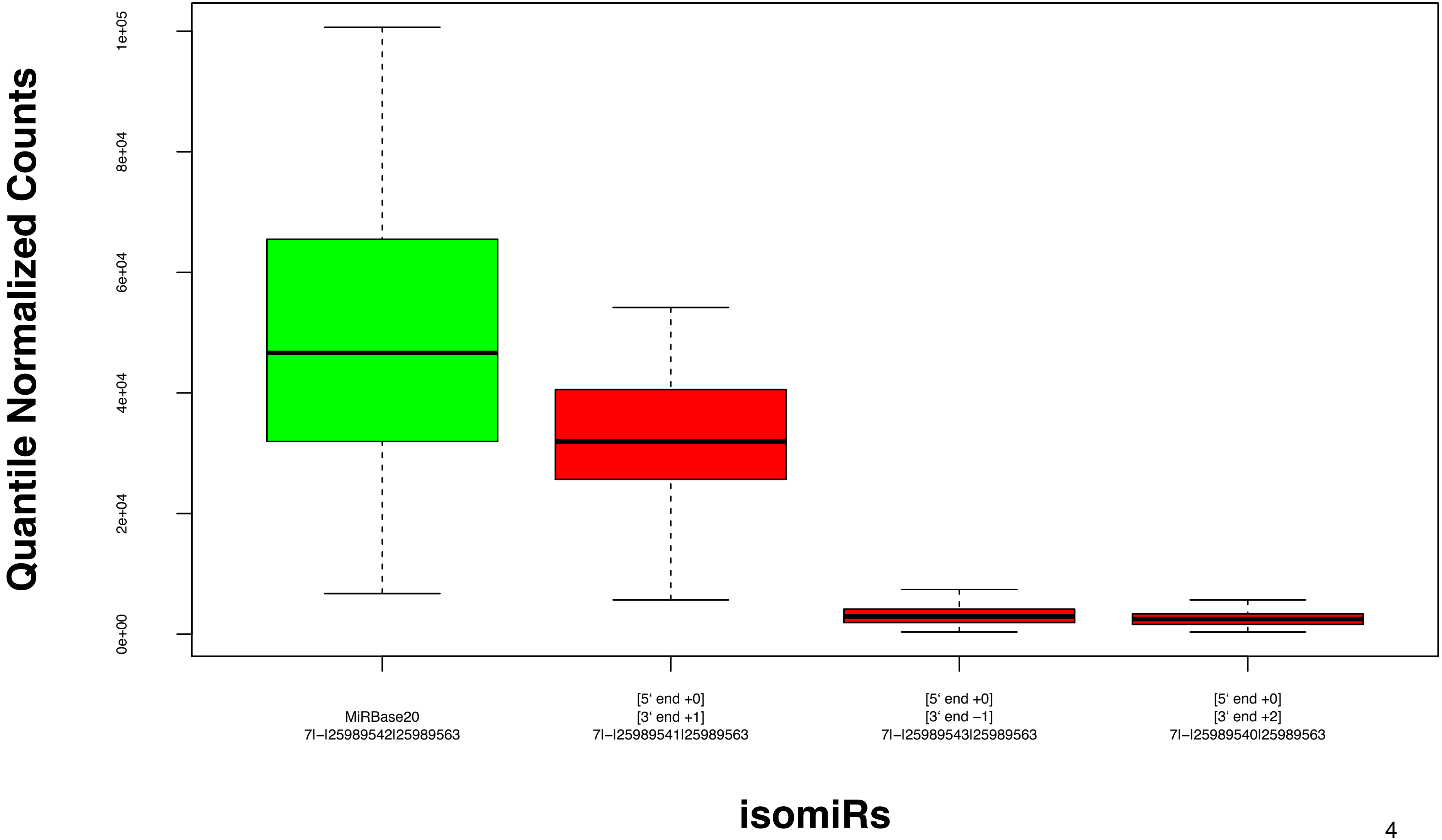

Supp. Figure 1
(pages 1 to 194)

MIMAT0002809=hsa–miR–146b–5p=MI0003129

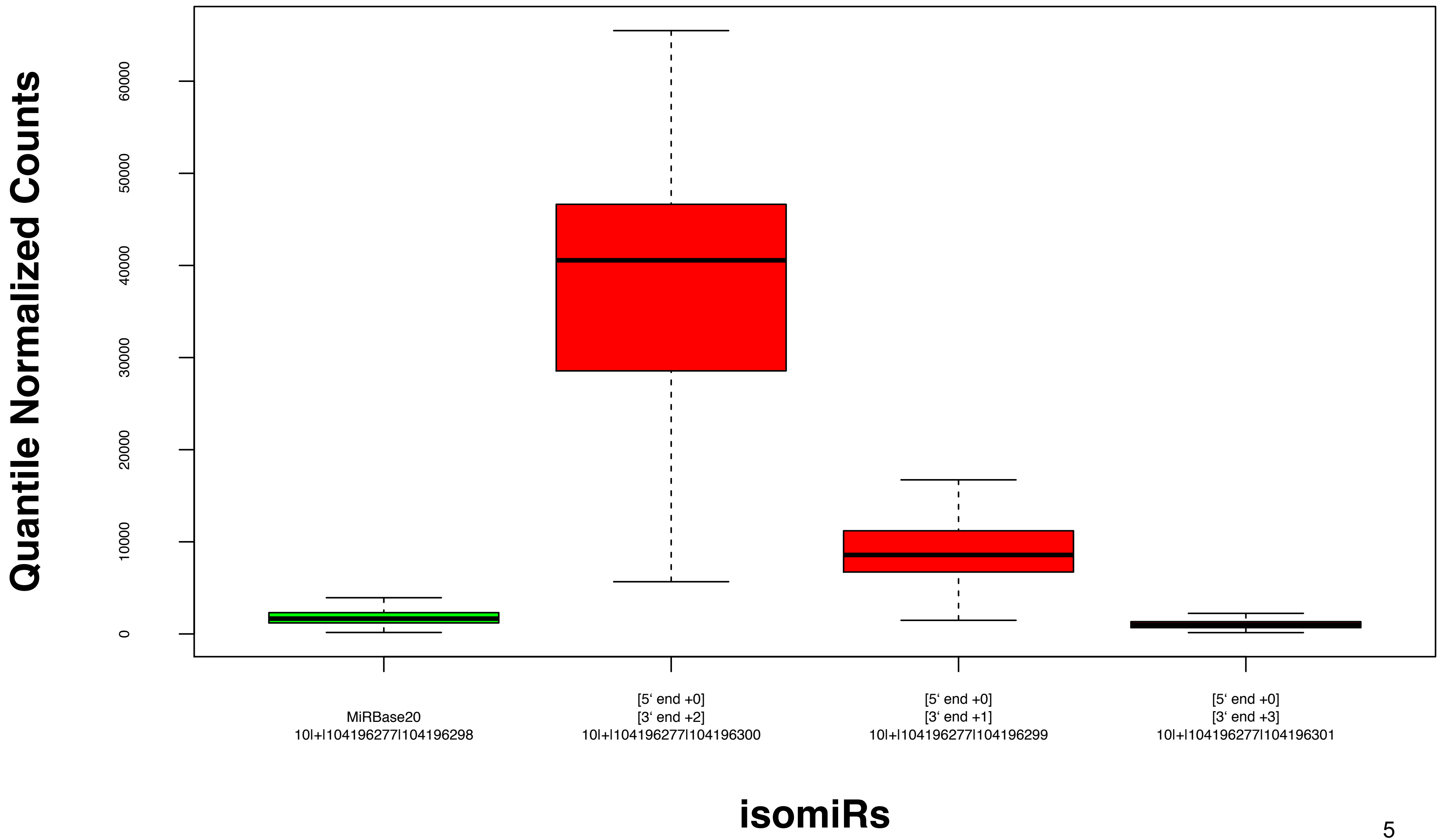

Supp. Figure 1
(pages 1 to 194)

MIMAT0000433=hsa−miR−142−5p=MI0000458

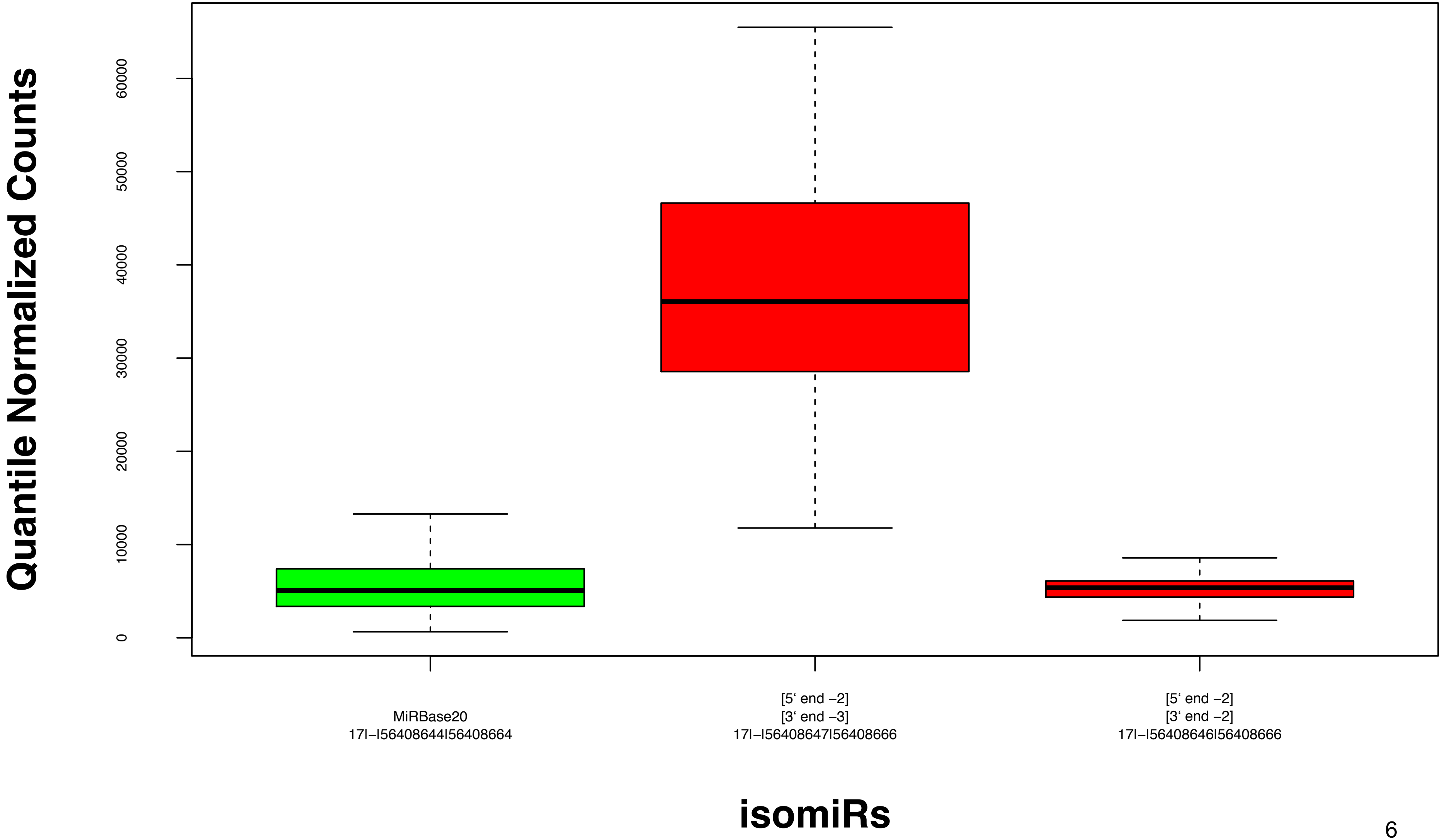

Supp. Figure 1
(pages 1 to 194)

MIMAT0000076=hsa–miR–21–5p=MI0000077

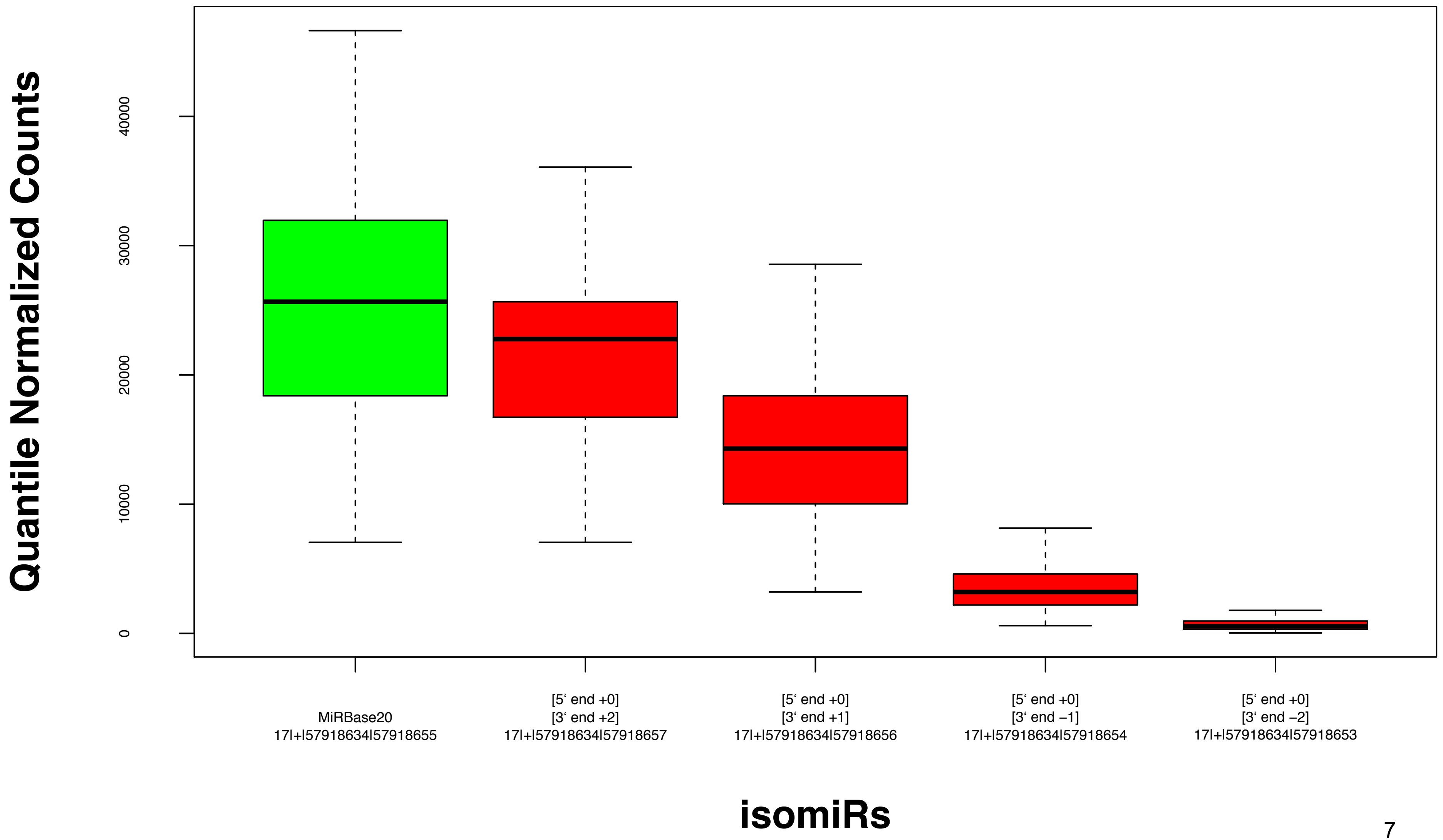

Supp. Figure 1
(pages 1 to 194)

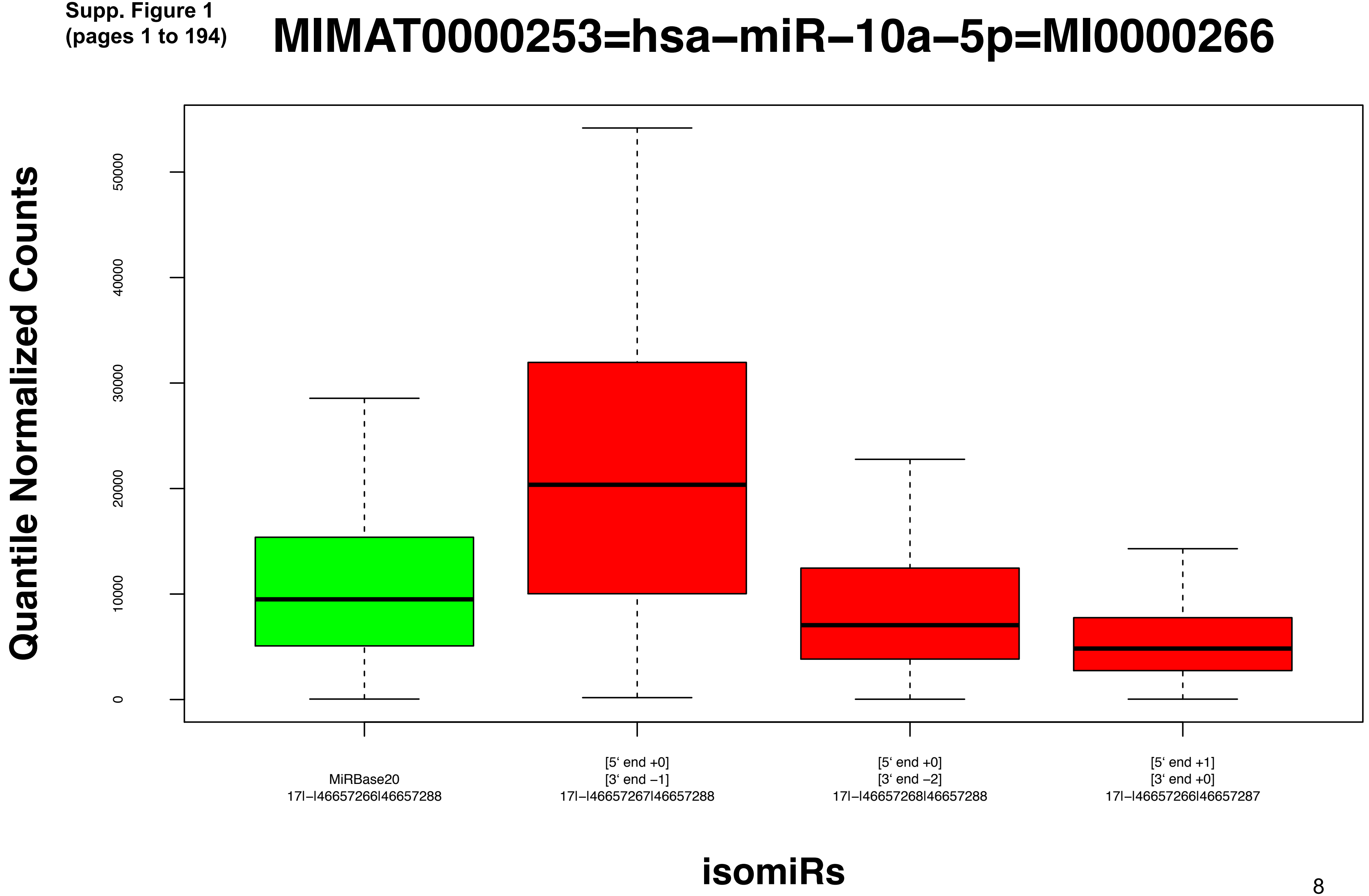

Supp. Figure 1
(pages 1 to 194)

MIMAT0000245=hsa–miR–30d–5p=MI0000255

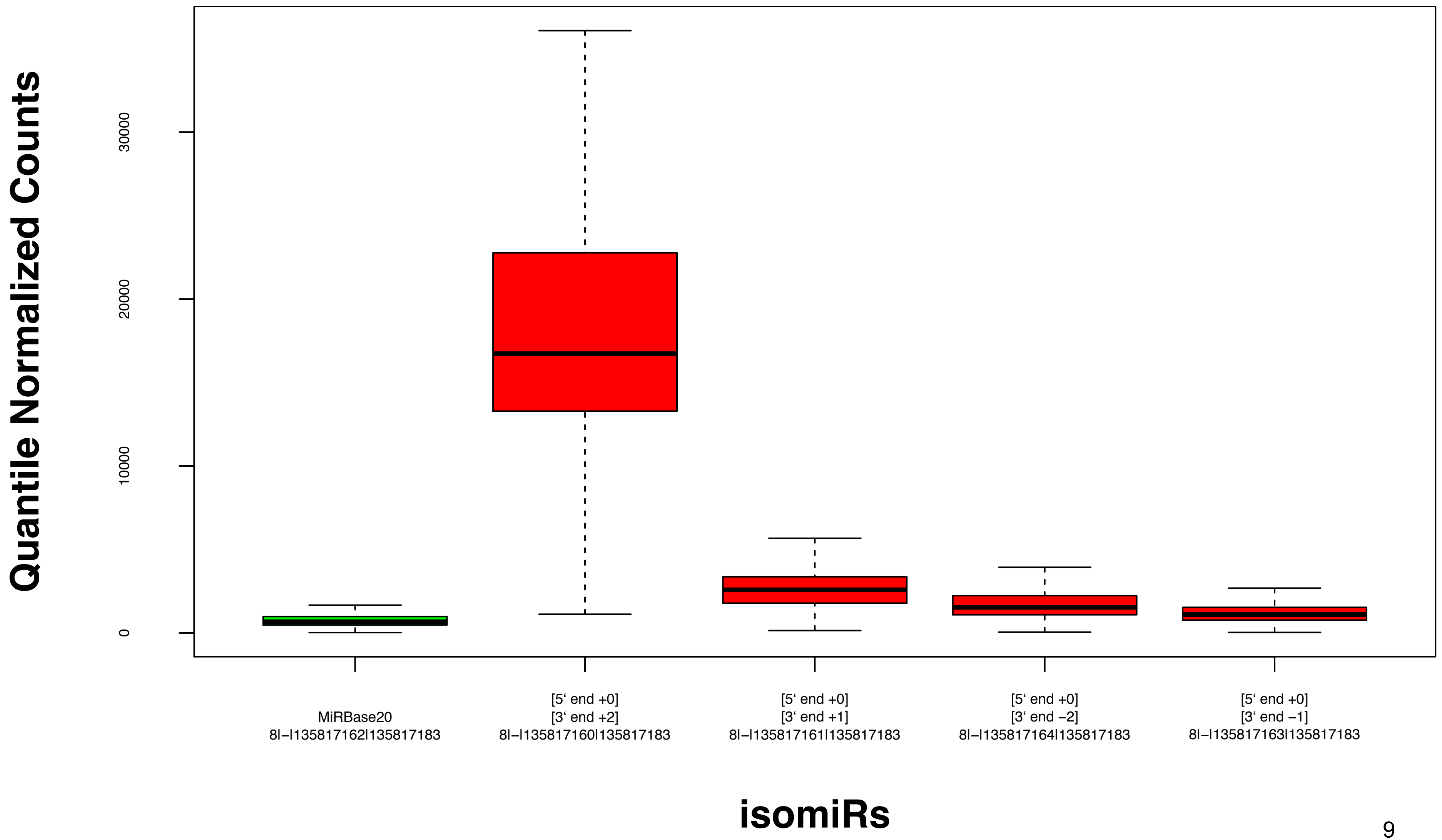

Supp. Figure 1
(pages 1 to 194)

MIMAT0000414=hsa–let–7g–5p=MI0000433

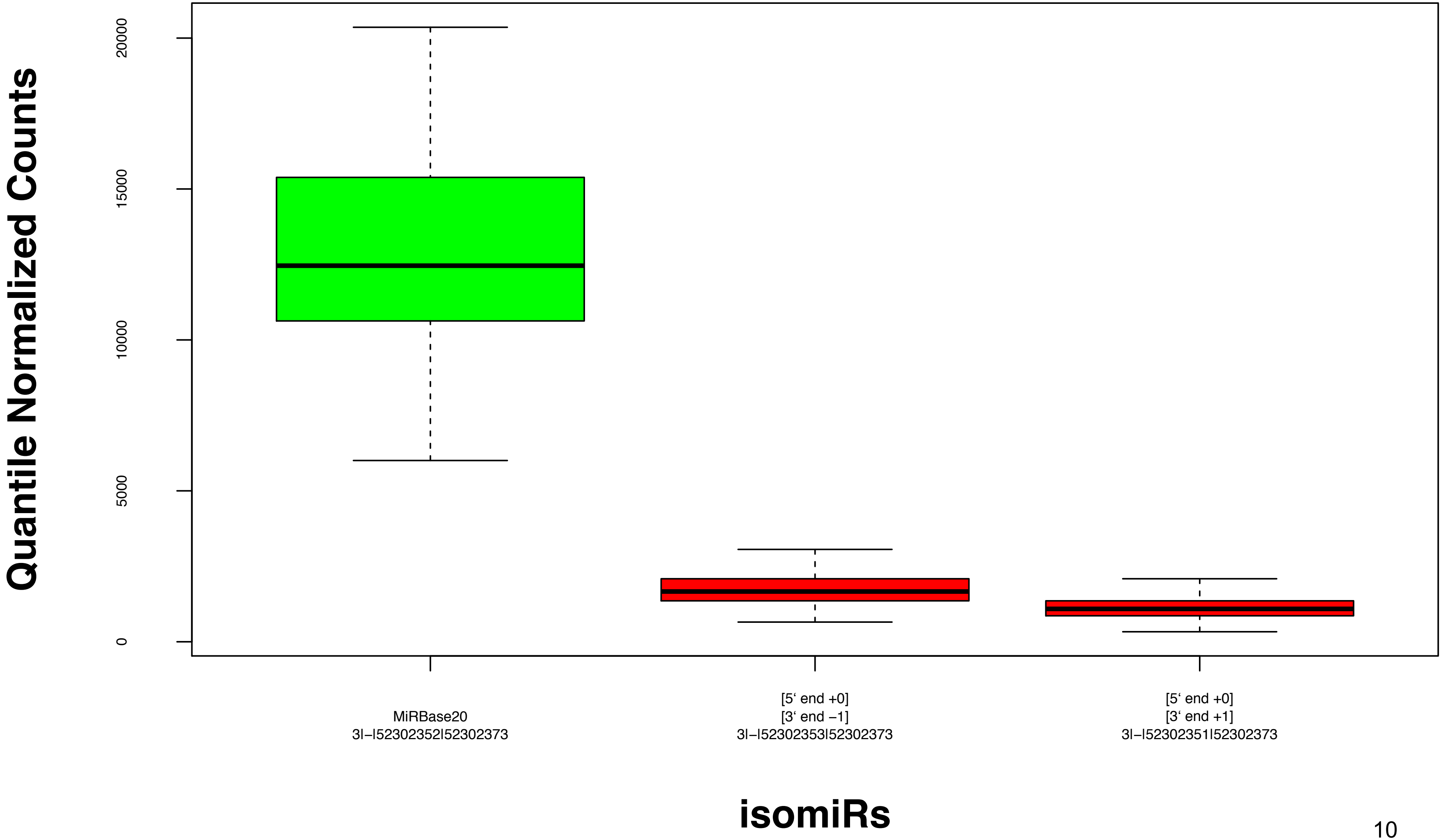

Supp. Figure 1
(pages 1 to 194)

MIMAT0004494=hsa–miR–21–3p=MI0000077

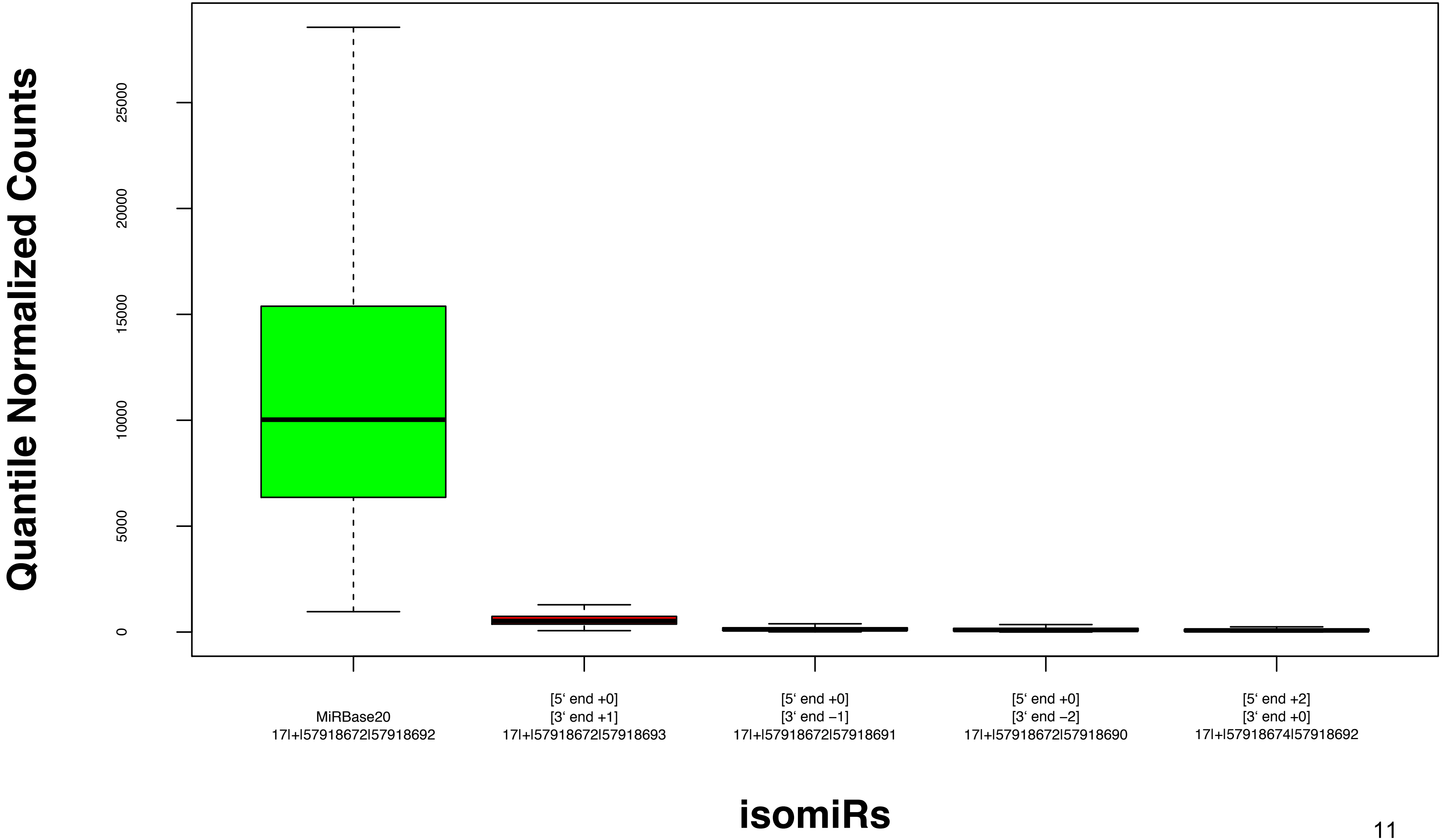

Supp. Figure 1
(pages 1 to 194)

MIMAT0000077=hsa−miR−22−3p=MI0000078

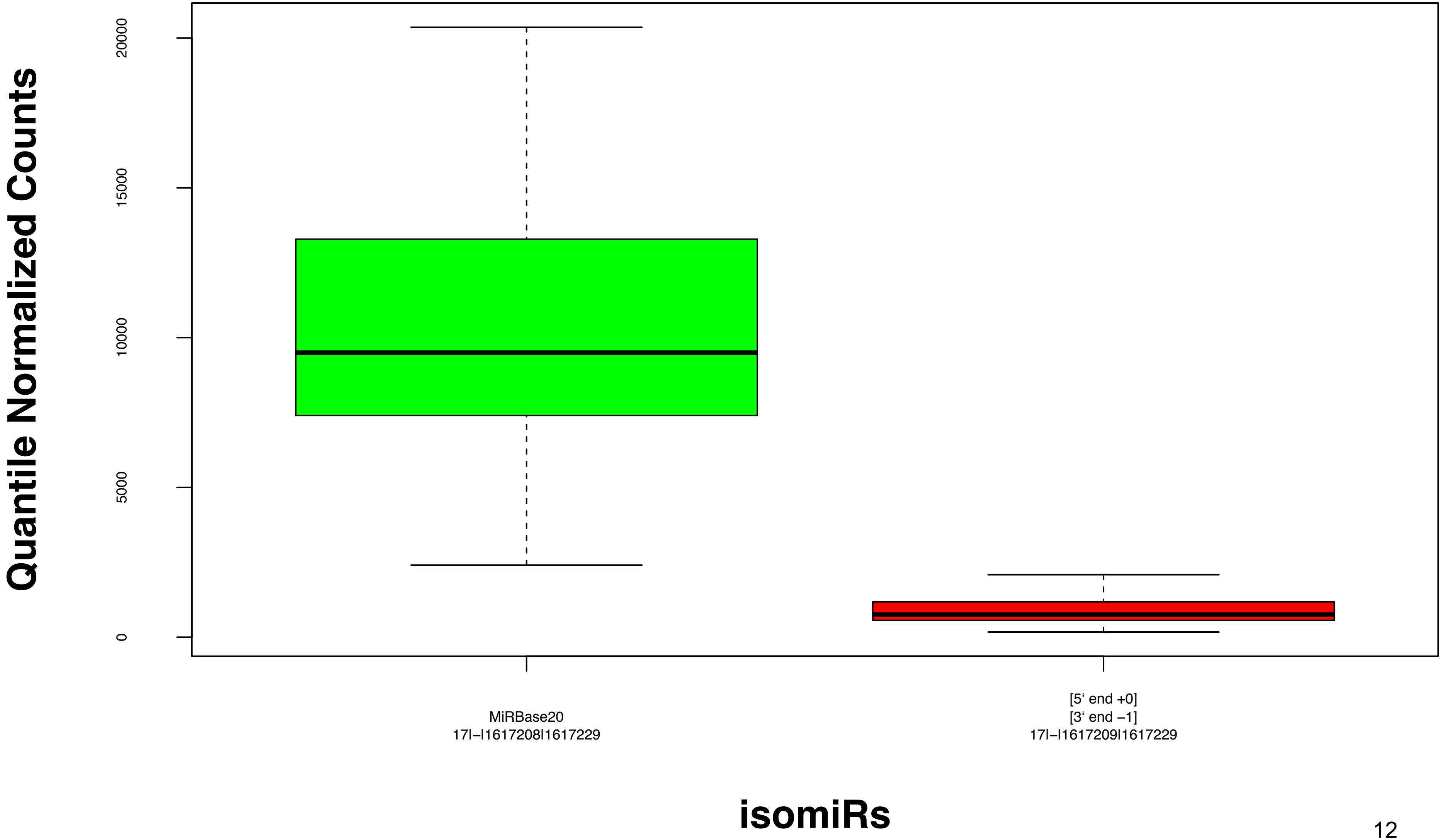

Supp. Figure 1
(pages 1 to 194)

MIMAT0000092=hsa–miR–92a–3p=MI0000093

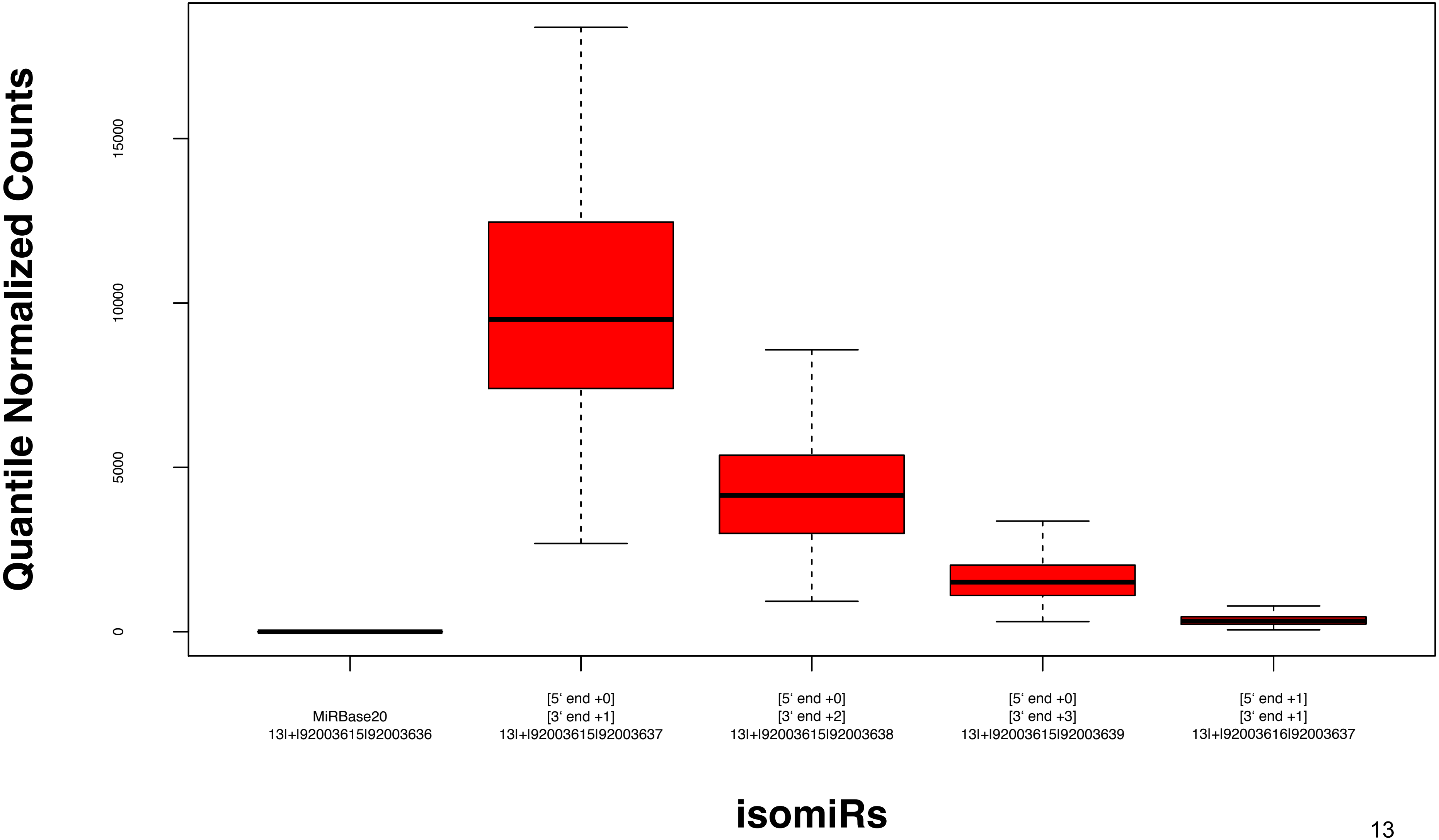

Supp. Figure 1
(pages 1 to 194)

MIMAT0000081=hsa−miR−25−3p=MI0000082

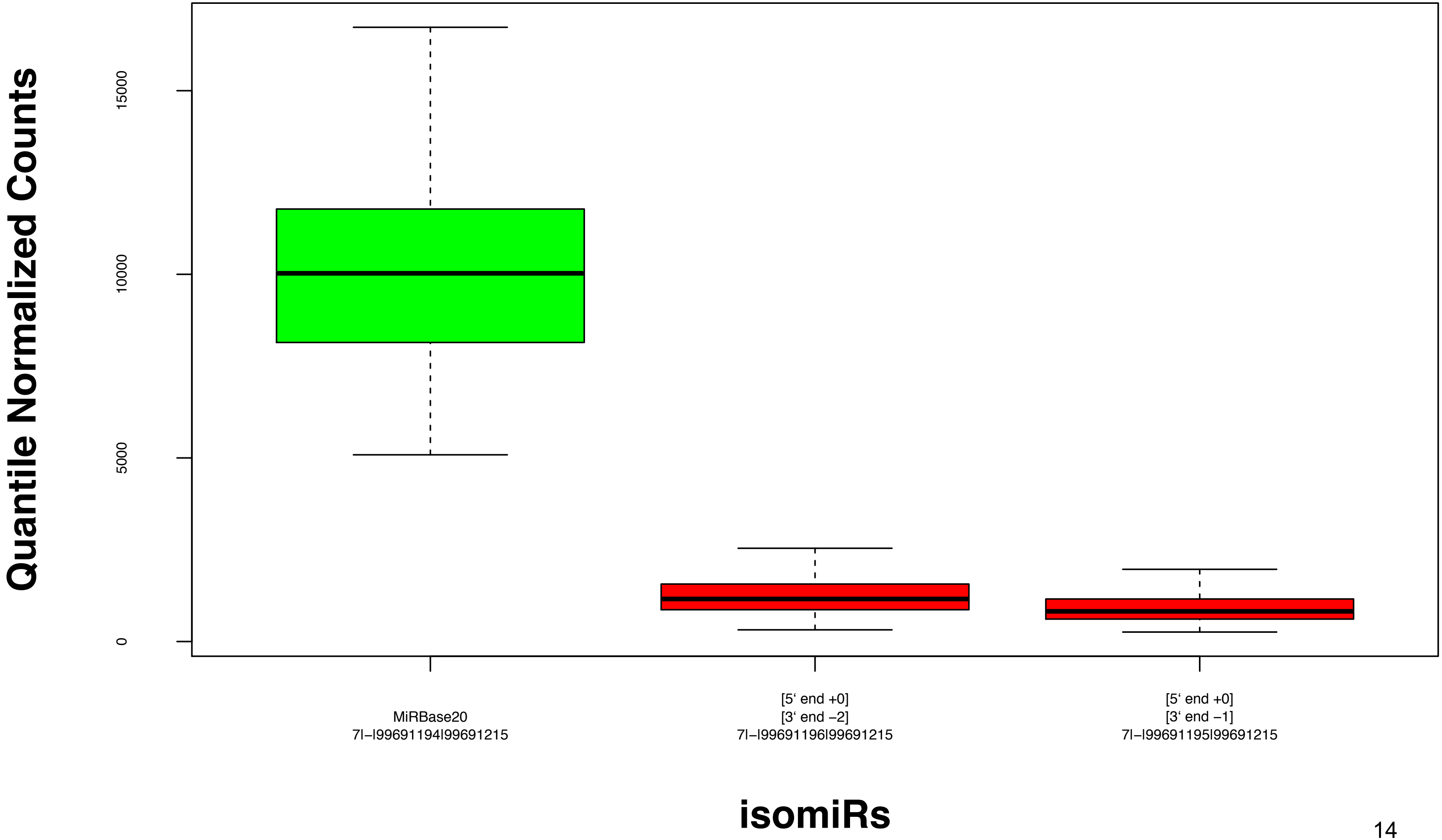

Supp. Figure 1
(pages 1 to 194)

MIMAT0000456=hsa−miR−186−5p=MI0000483

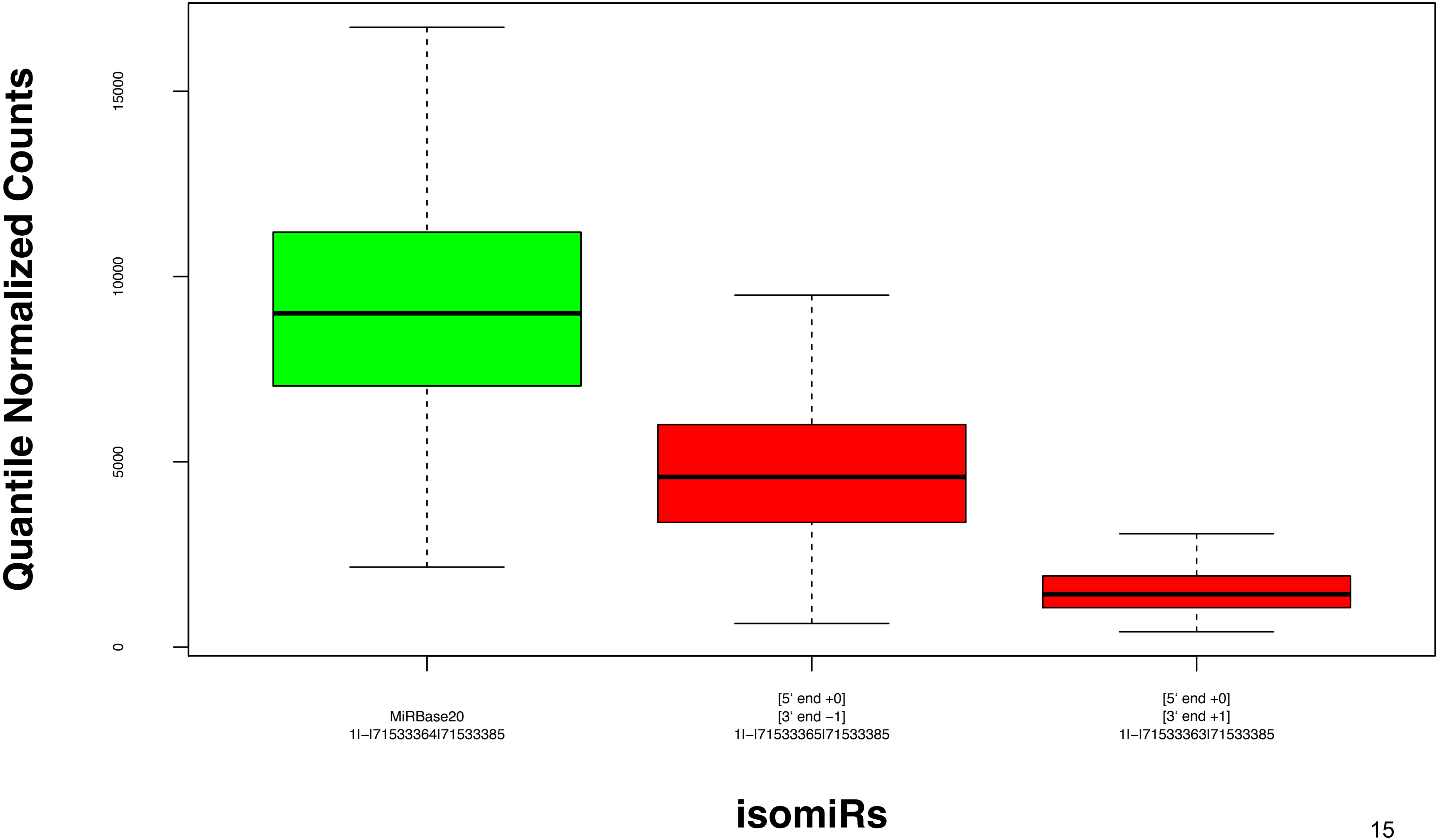

Supp. Figure 1
(pages 1 to 194)

MIMAT0000419=hsa−miR−27b−3p=MI0000440

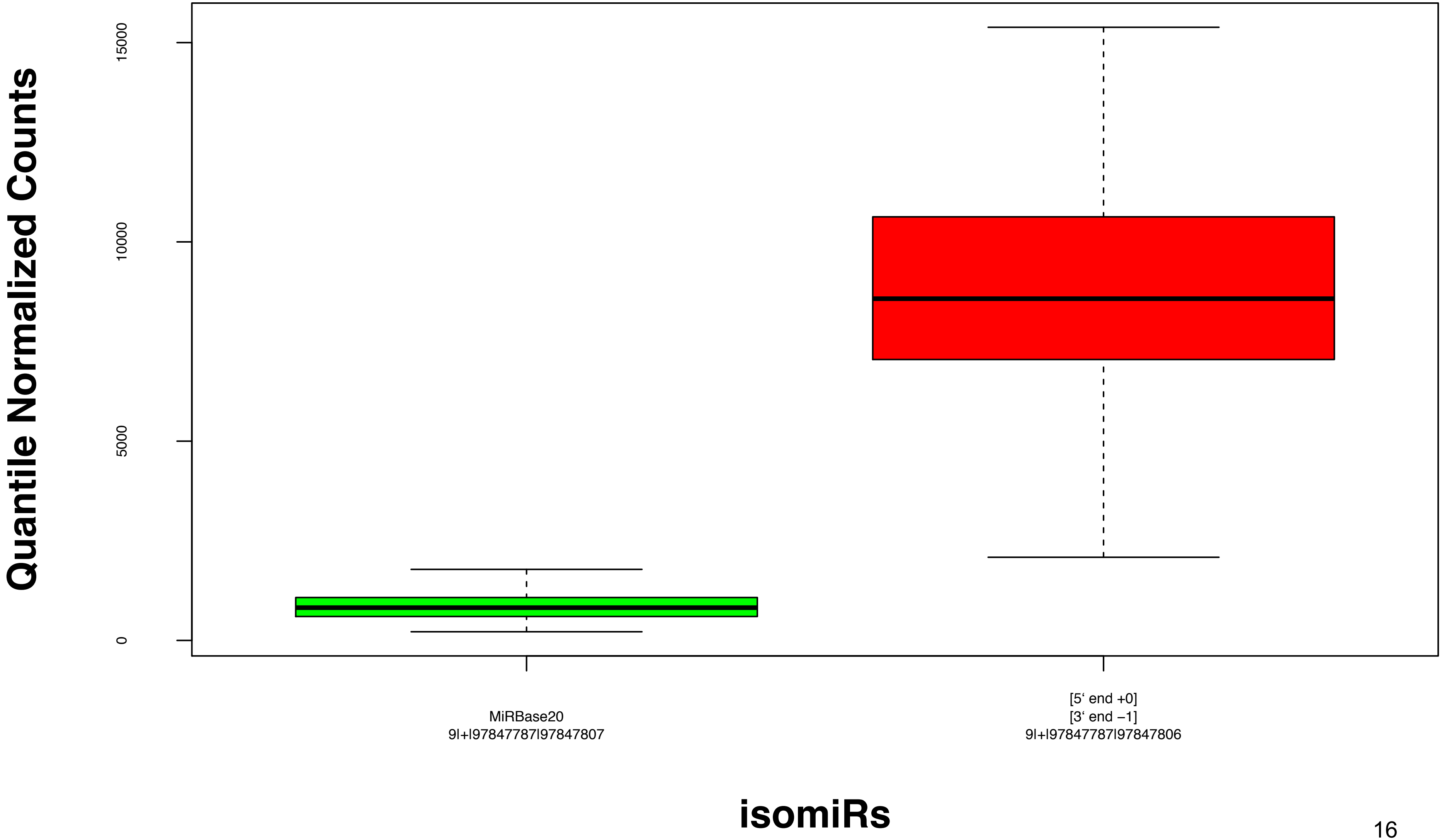

Supp. Figure 1
(pages 1 to 194)

MIMAT0000692=hsa–miR–30e–5p=MI0000749

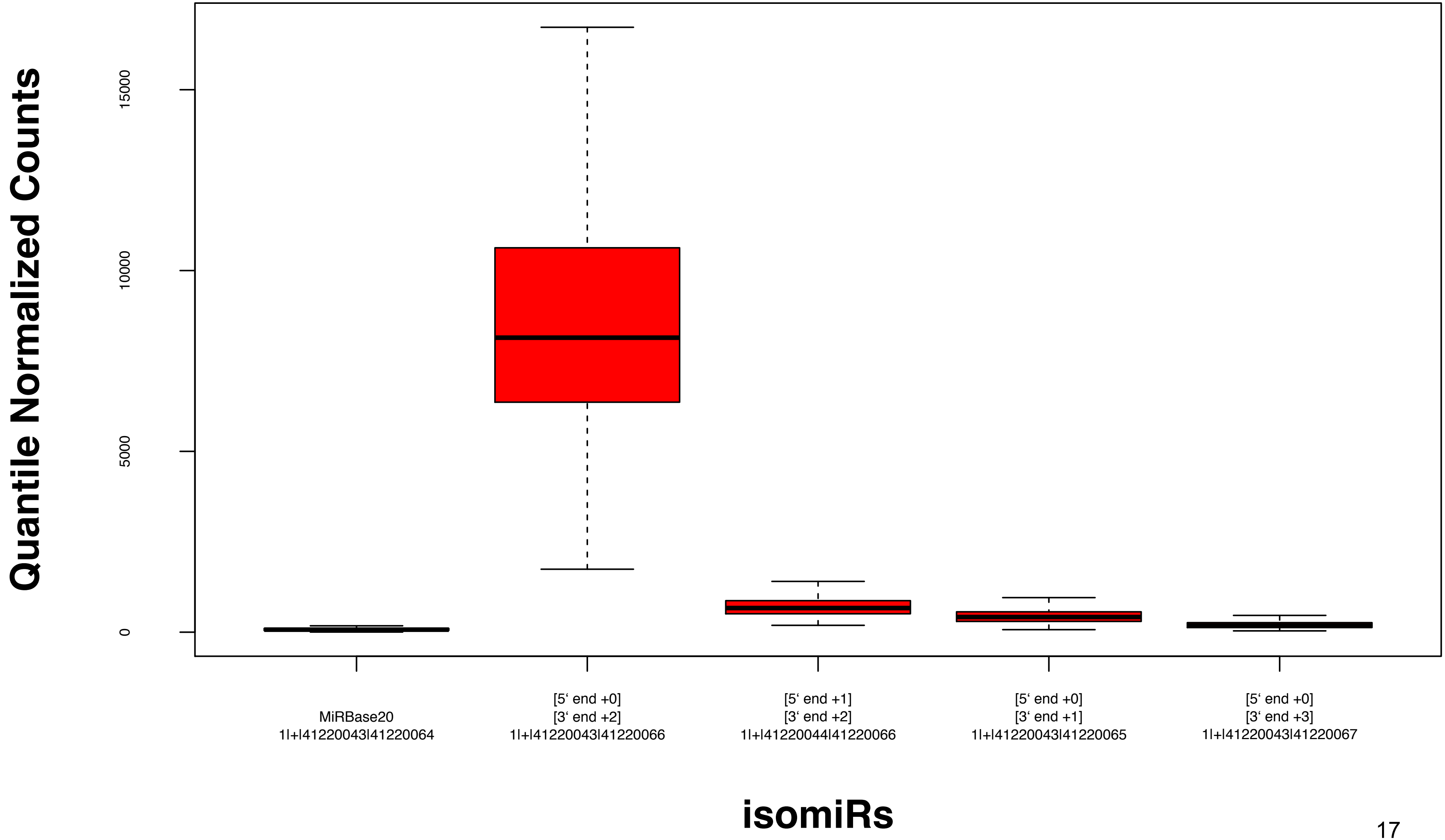

Supp. Figure 1
(pages 1 to 194)

MIMAT0000096=hsa–miR–98–5p=MI0000100

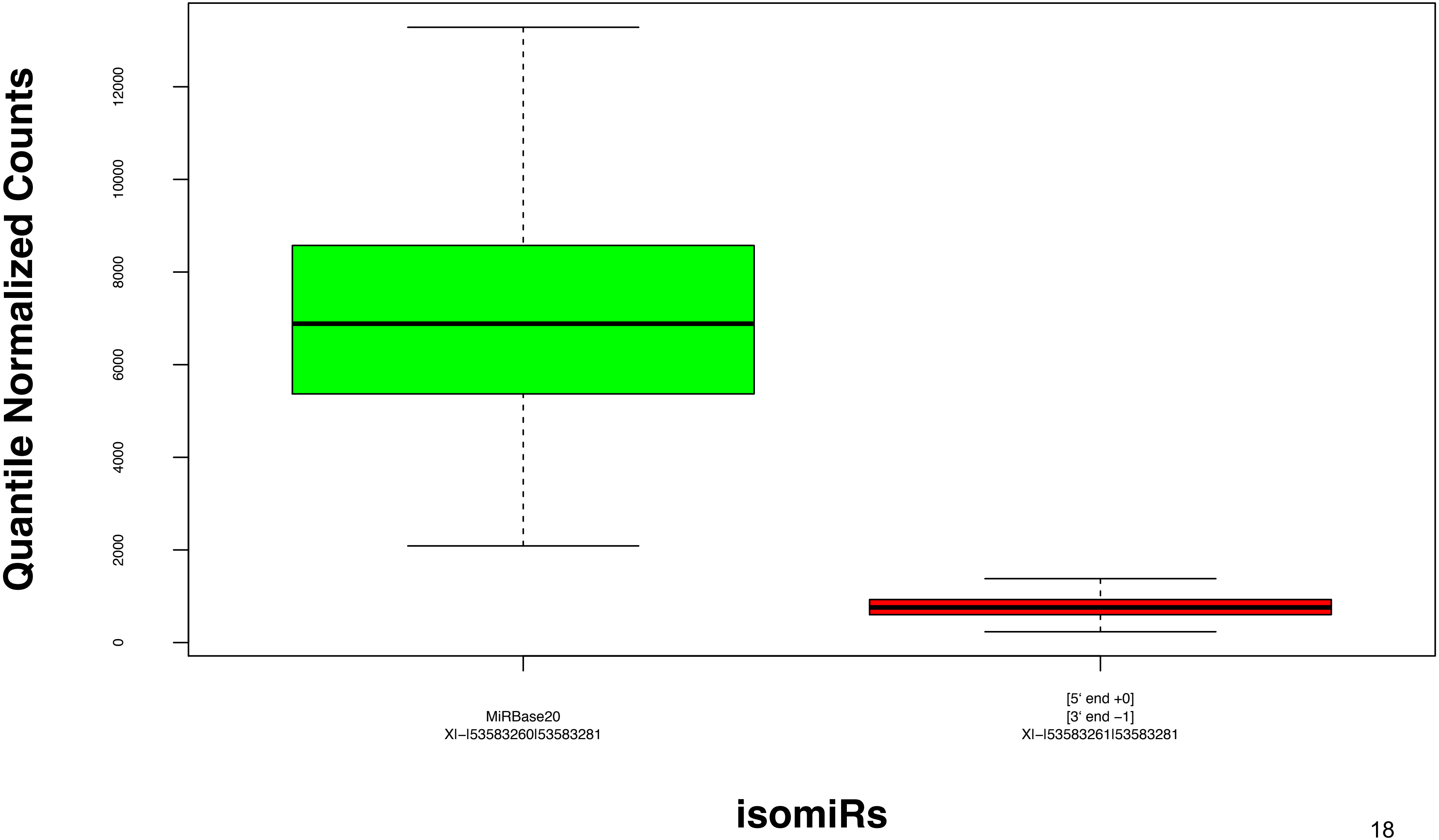

Supp. Figure 1
(pages 1 to 194)

MIMAT0000415=hsa–let–7i–5p=MI0000434

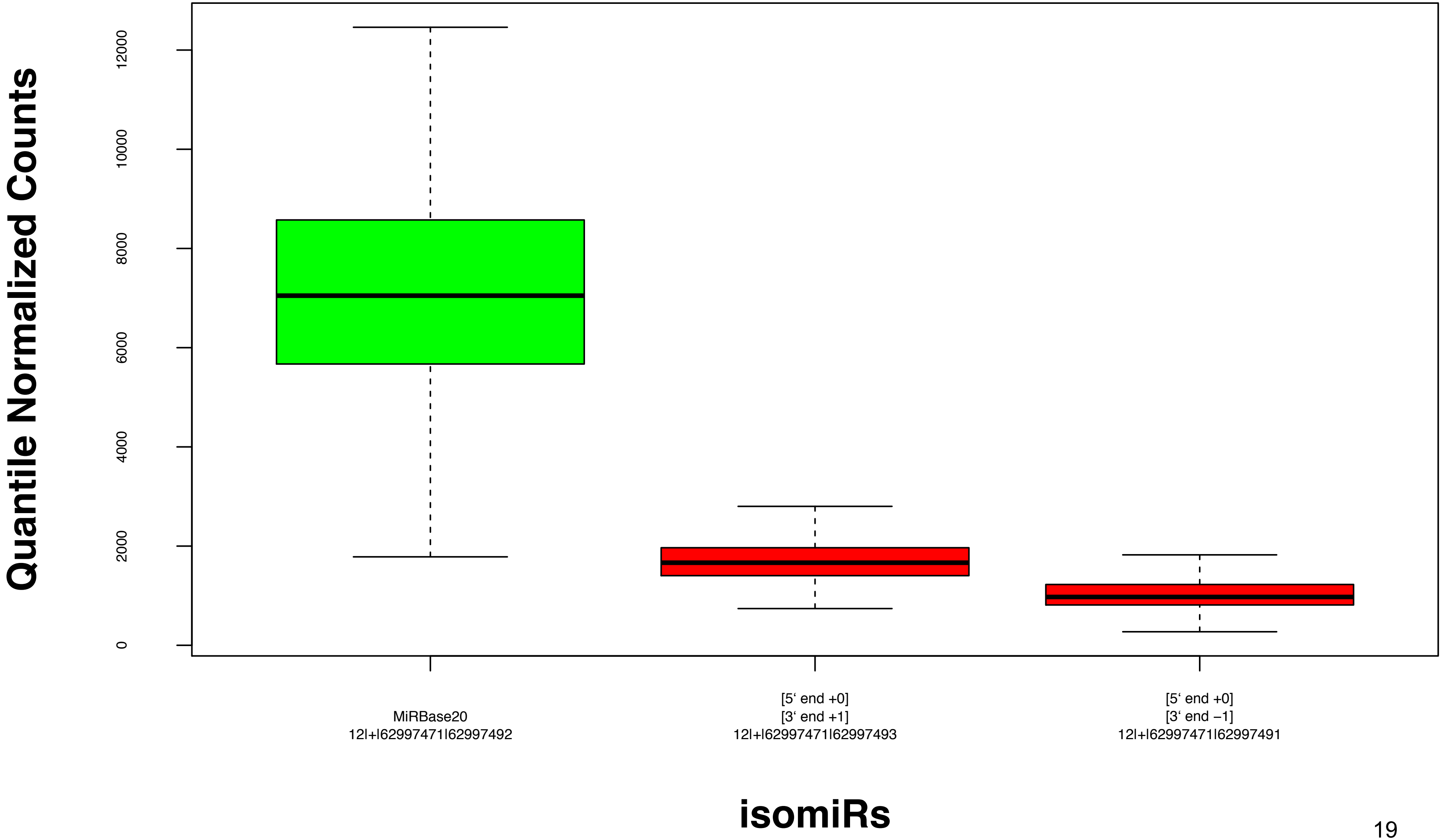

Supp. Figure 1
(pages 1 to 194)

MIMAT0000222=hsa−miR−192−5p=MI0000234

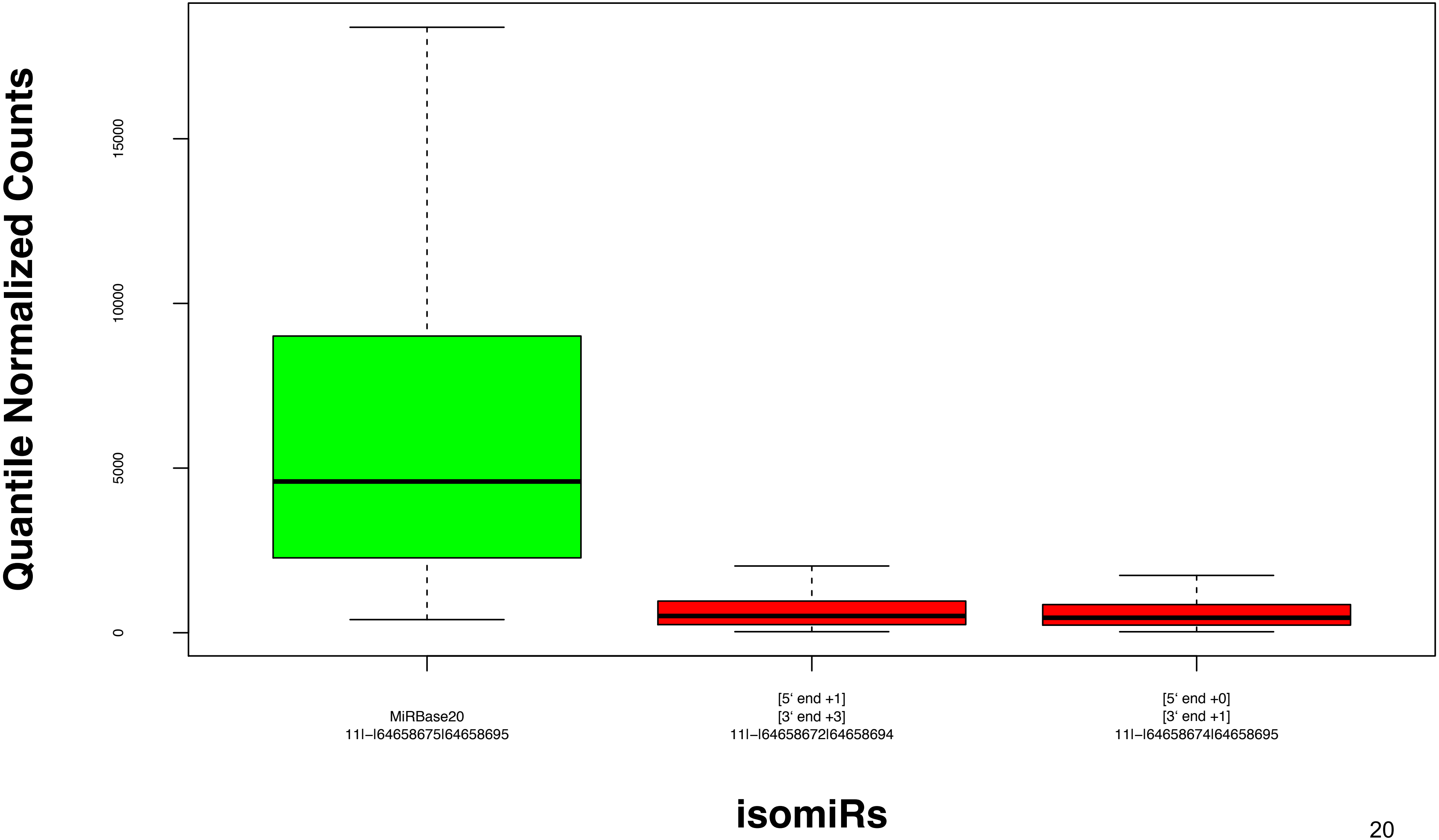

Supp. Figure 1
(pages 1 to 194)

MIMAT0000065=hsa−let−7d−5p=MI0000065

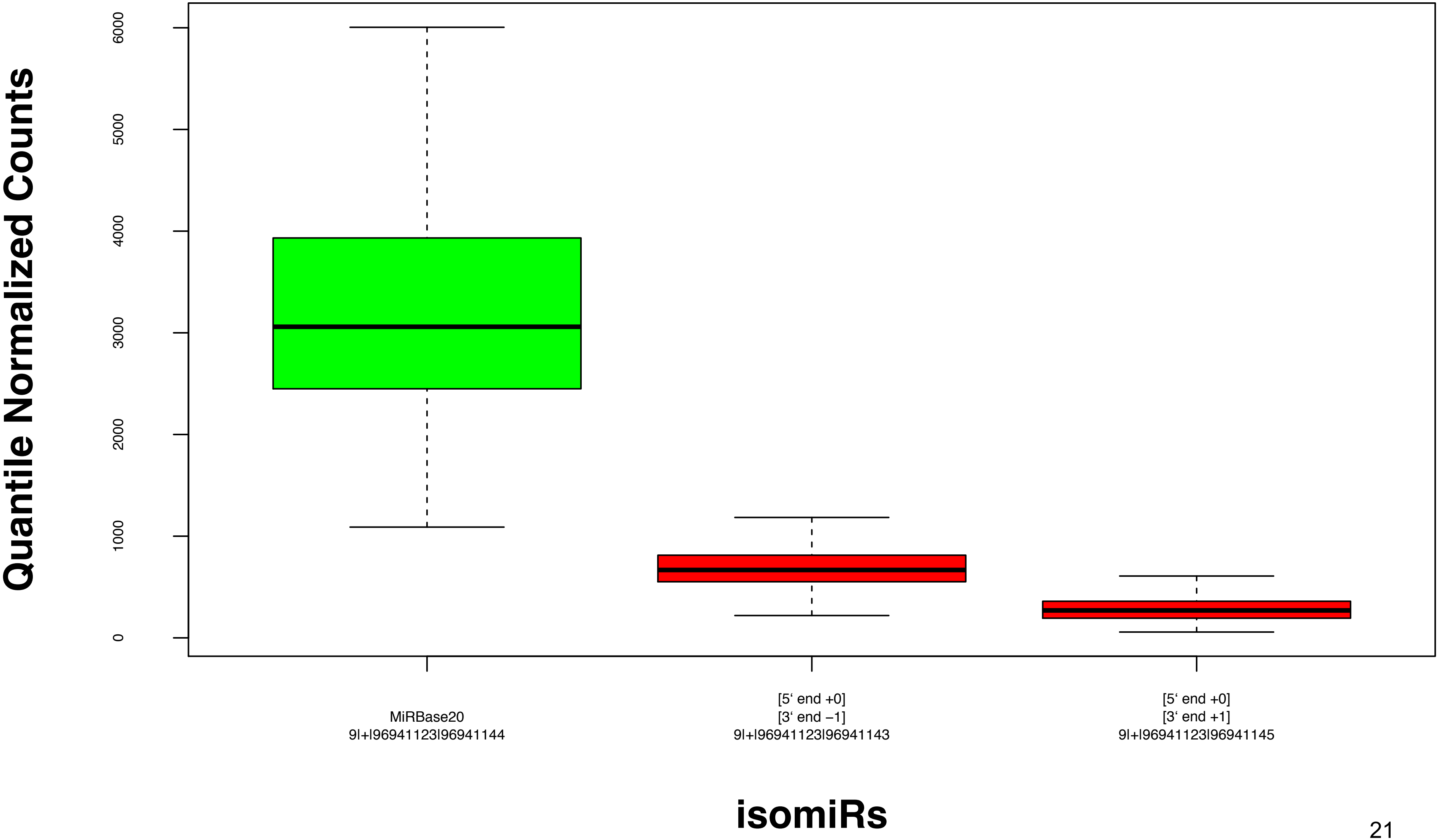

Supp. Figure 1
(pages 1 to 194)

MIMAT0001340=hsa−miR−423−3p=MI0001445

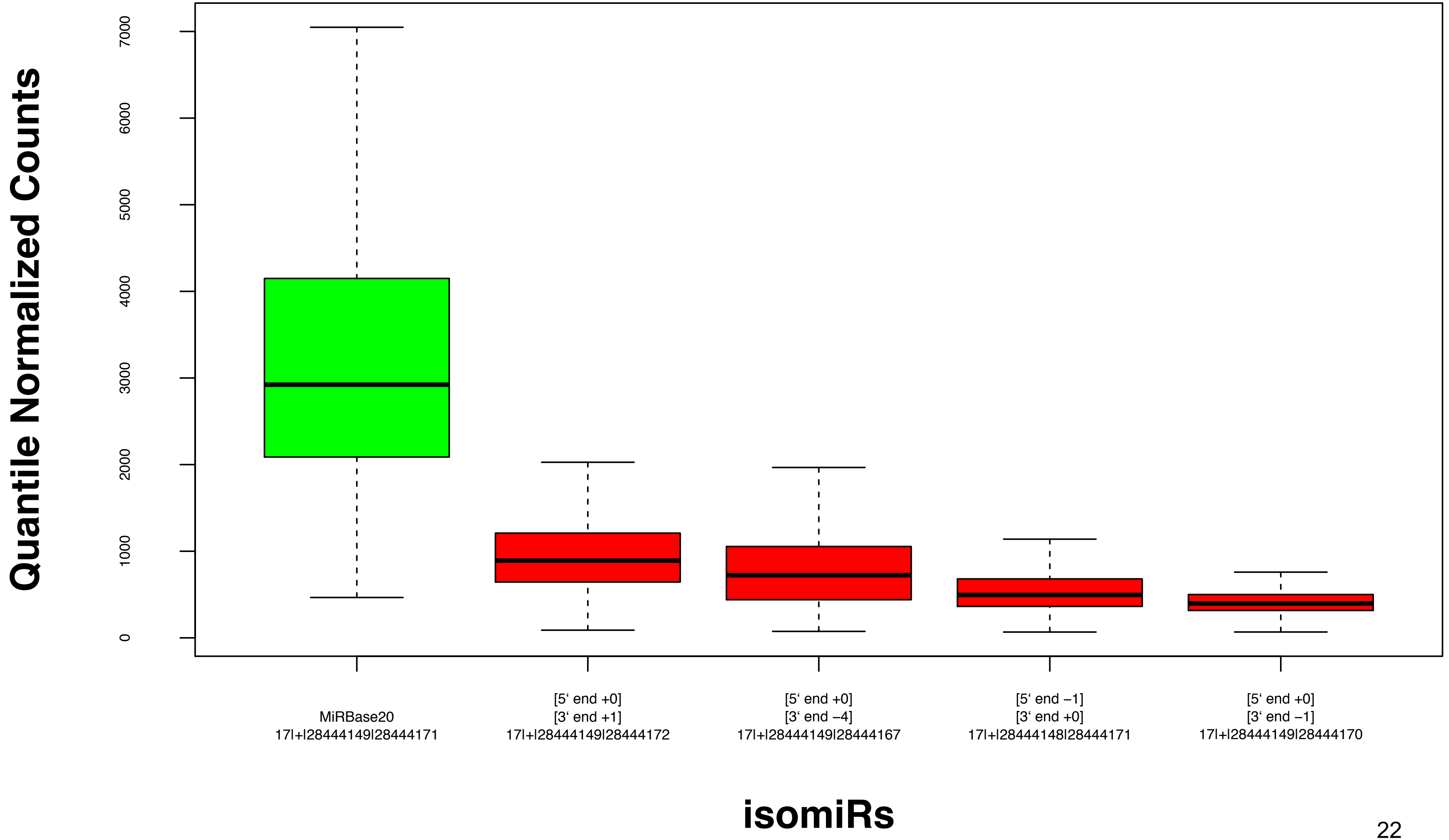

Supp. Figure 1
(pages 1 to 194)

MIMAT0004748=hsa−miR−423−5p=MI0001445

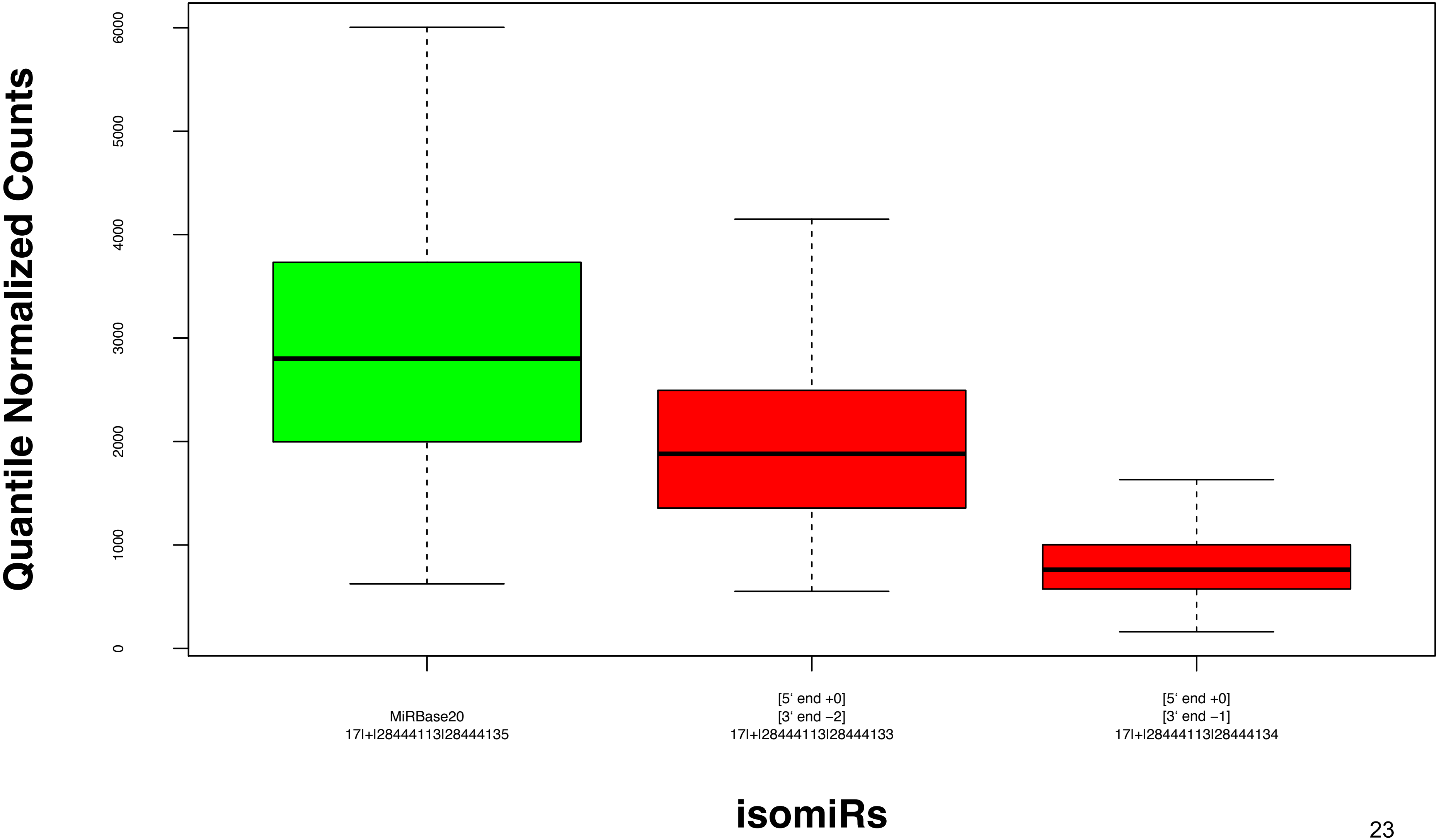

Supp. Figure 1
(pages 1 to 194)

MIMAT0004549=hsa−miR−148a−5p=MI0000253

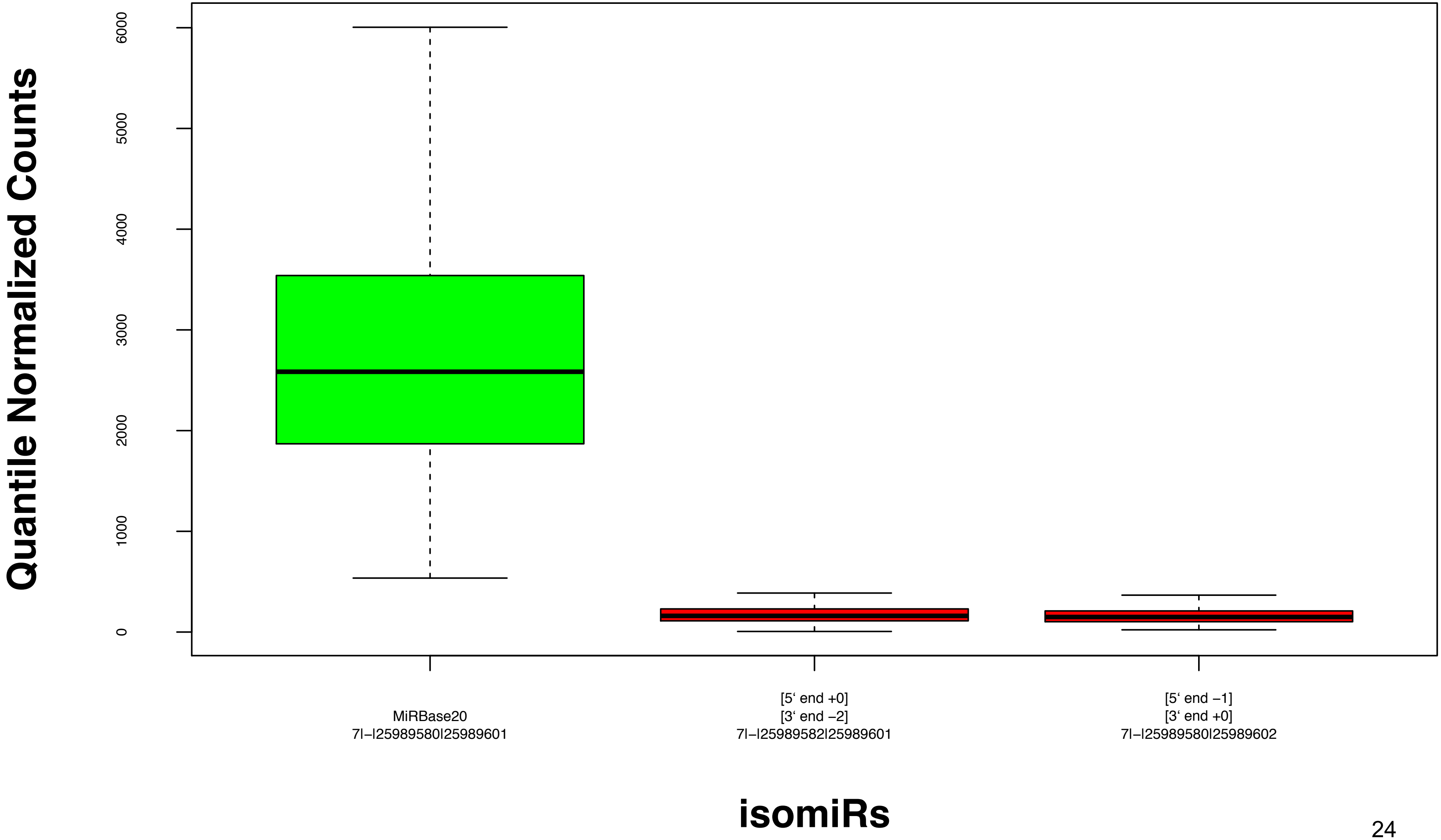

Supp. Figure 1
(pages 1 to 194)

MIMAT0000083=hsa–miR–26b–5p=MI0000084

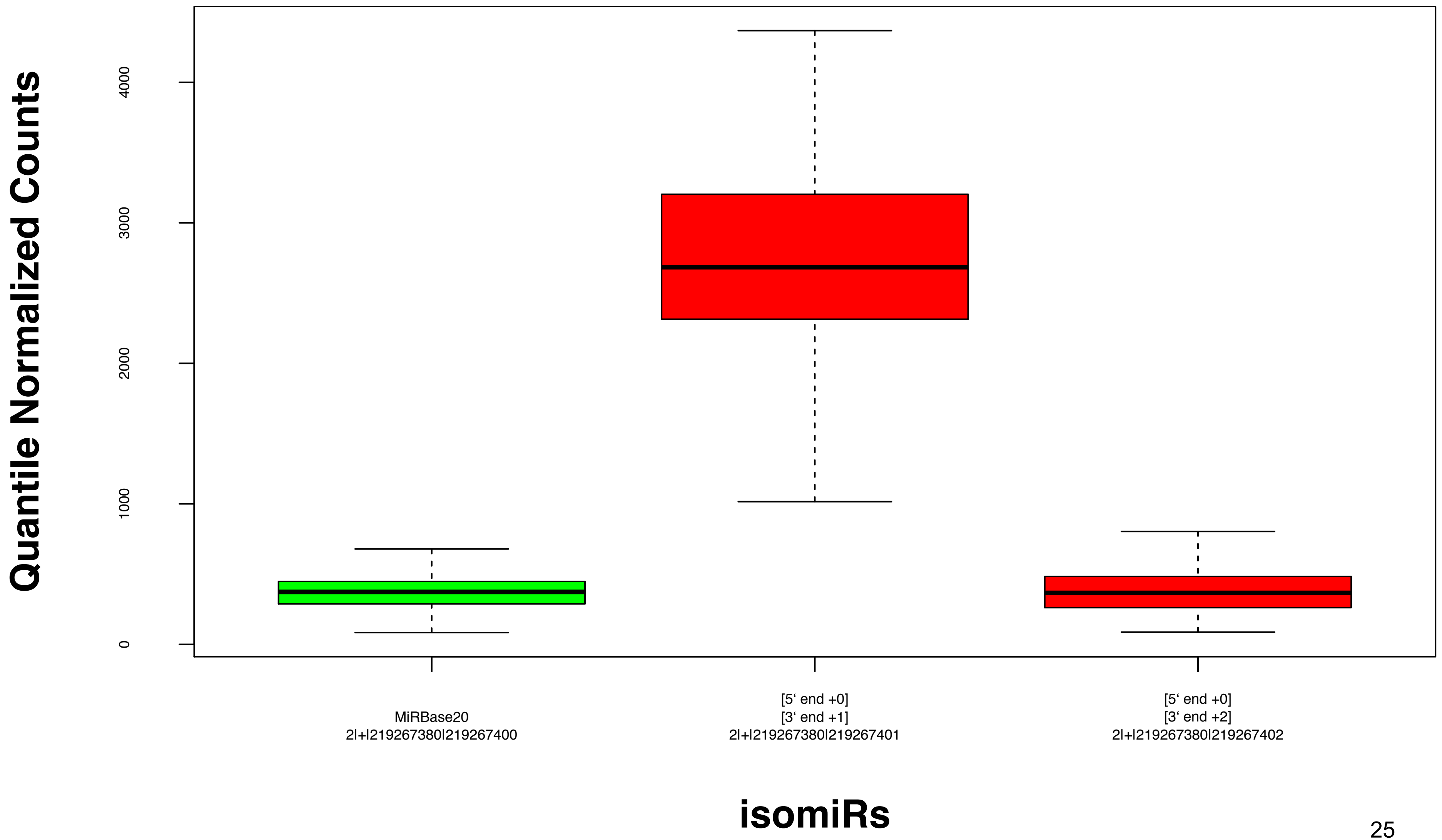

Supp. Figure 1
(pages 1 to 194)

MIMAT0002819=hsa–miR–193b–3p=MI0003137

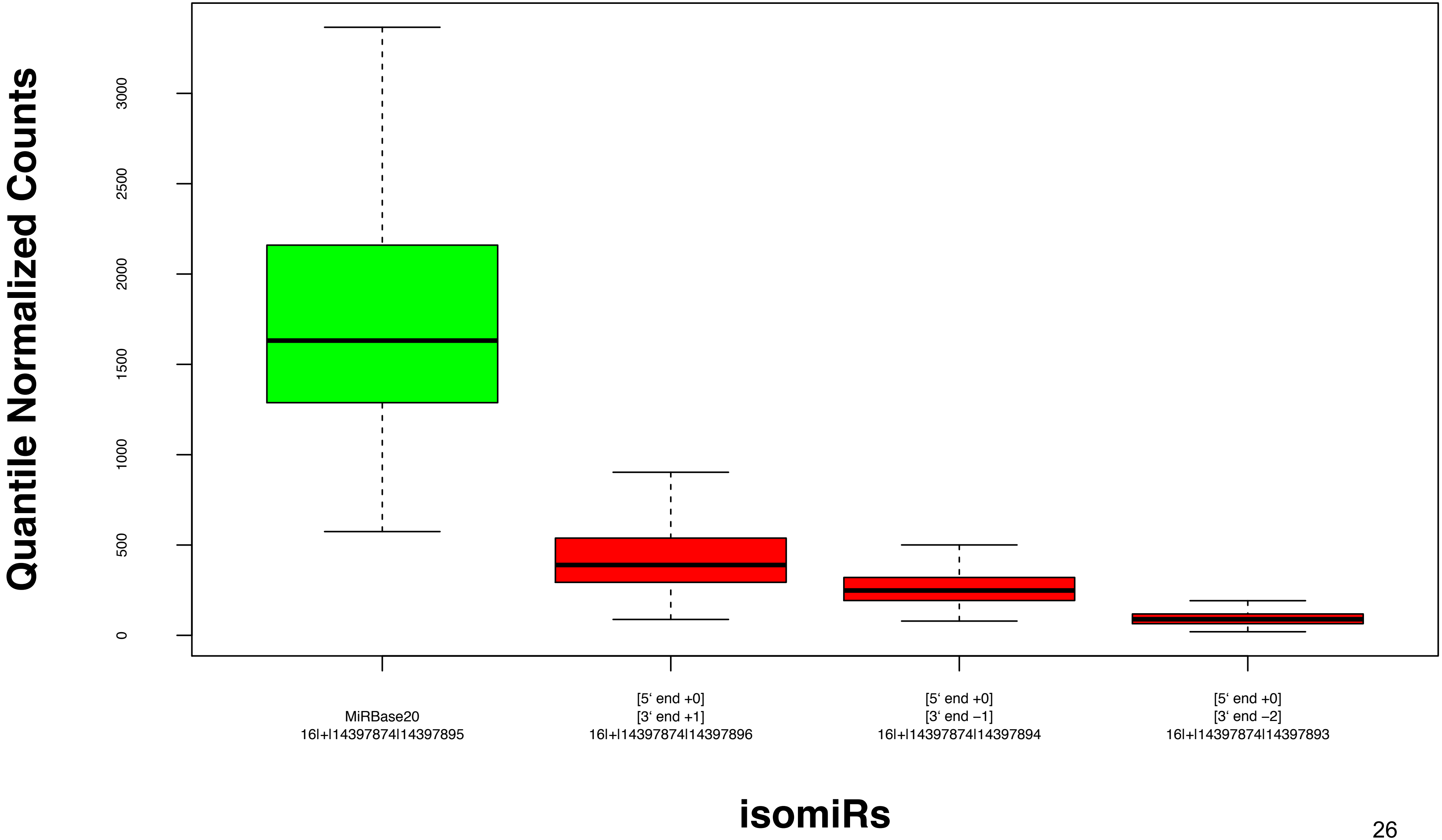

Supp. Figure 1
(pages 1 to 194)

MIMAT0000417=hsa−miR−15b−5p=MI0000438

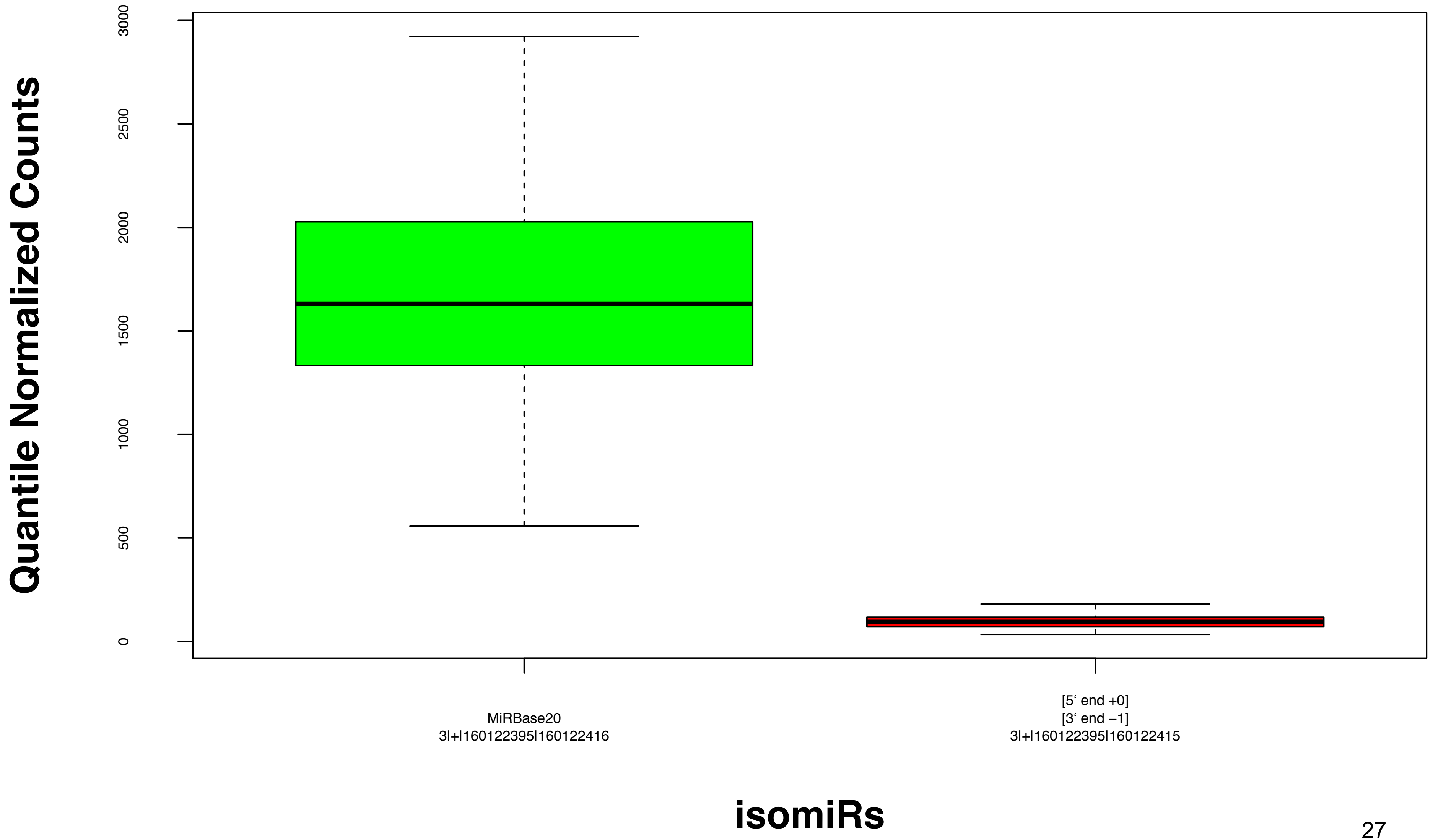

Supp. Figure 1
(pages 1 to 194)

MIMAT0000420=hsa−miR−30b−5p=MI0000441

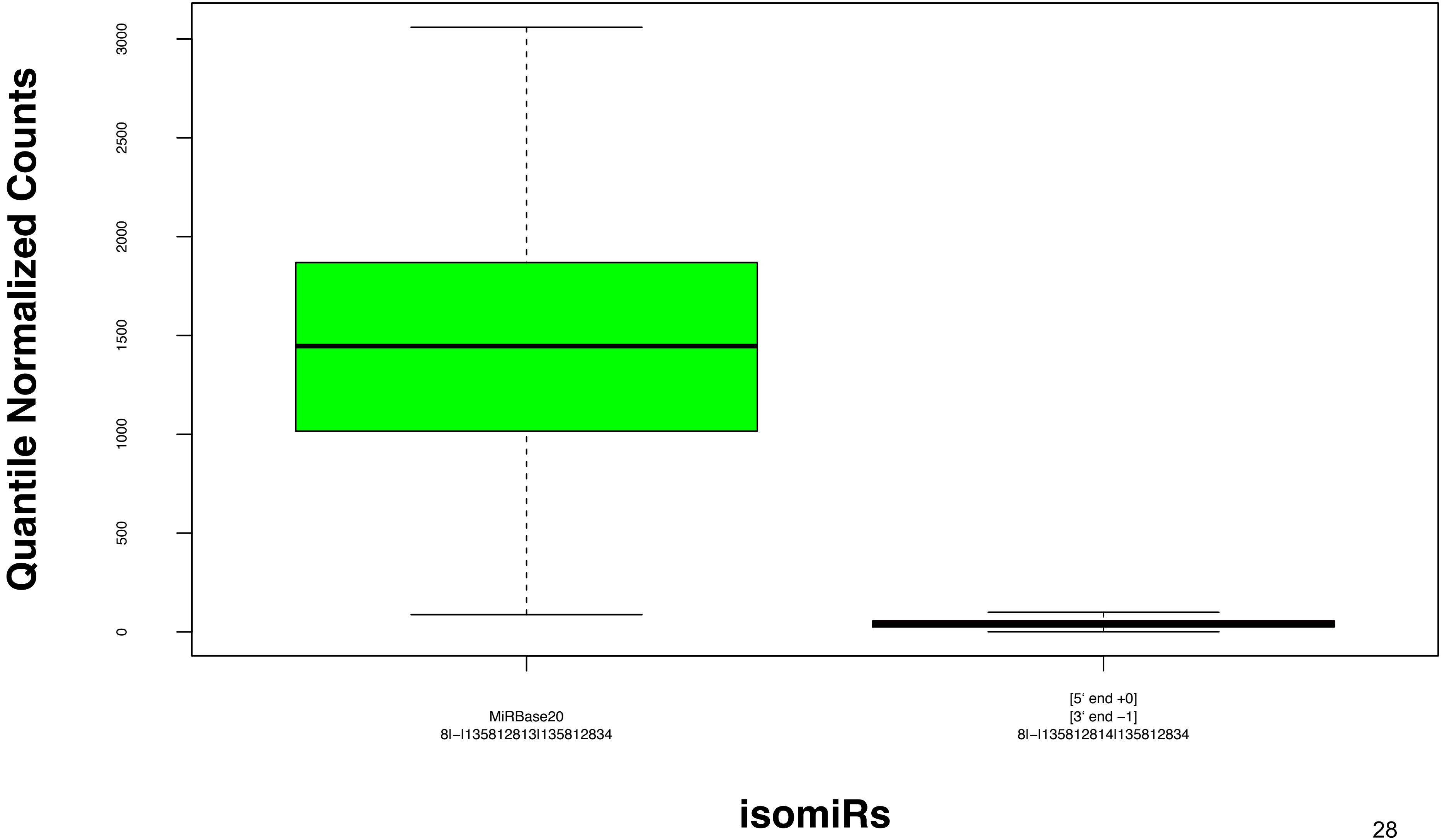

Supp. Figure 1
(pages 1 to 194)

MIMAT0000075=hsa−miR−20a−5p=MI0000076

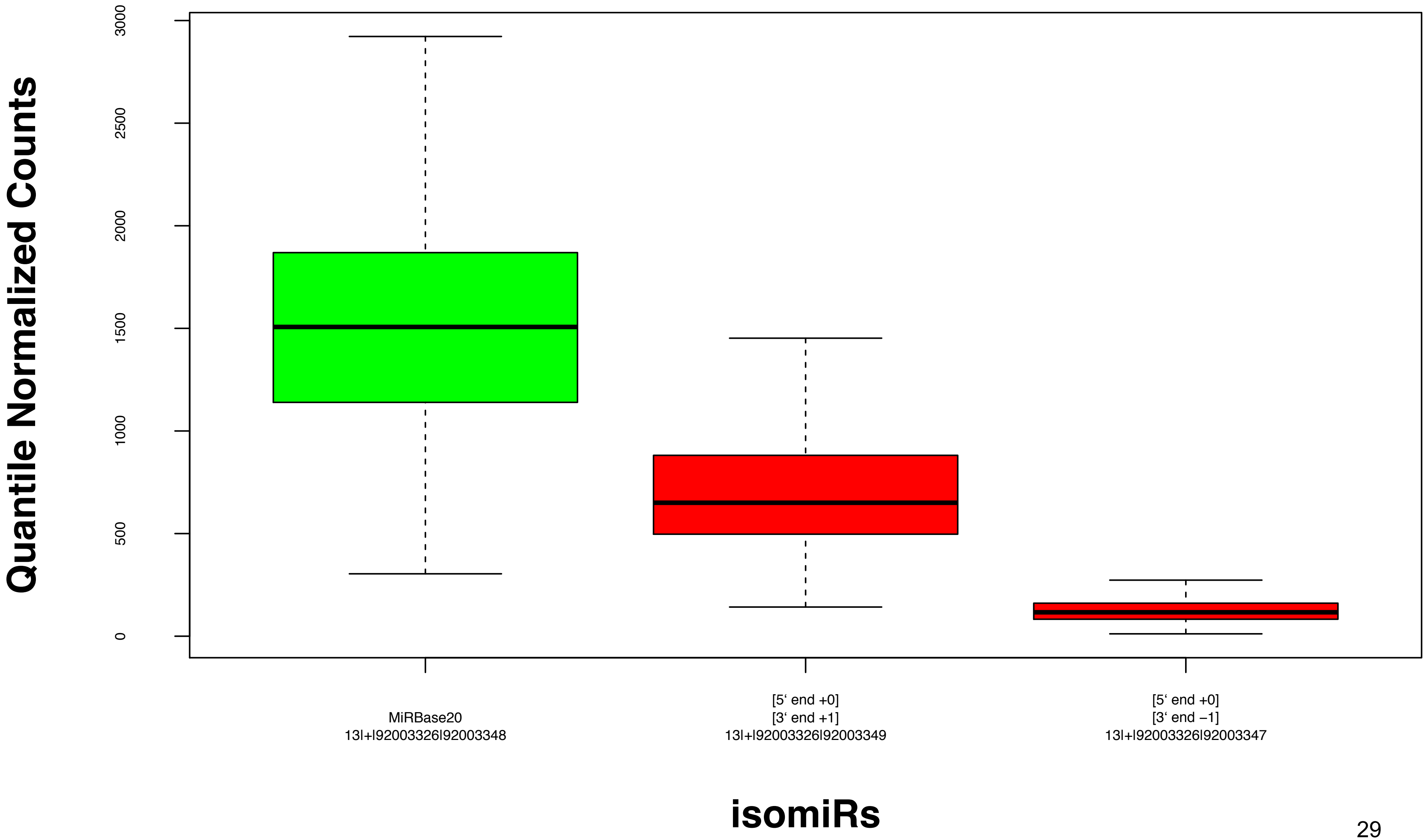

Supp. Figure 1
(pages 1 to 194)

MIMAT0000093=hsa–miR–93–5p=MI0000095

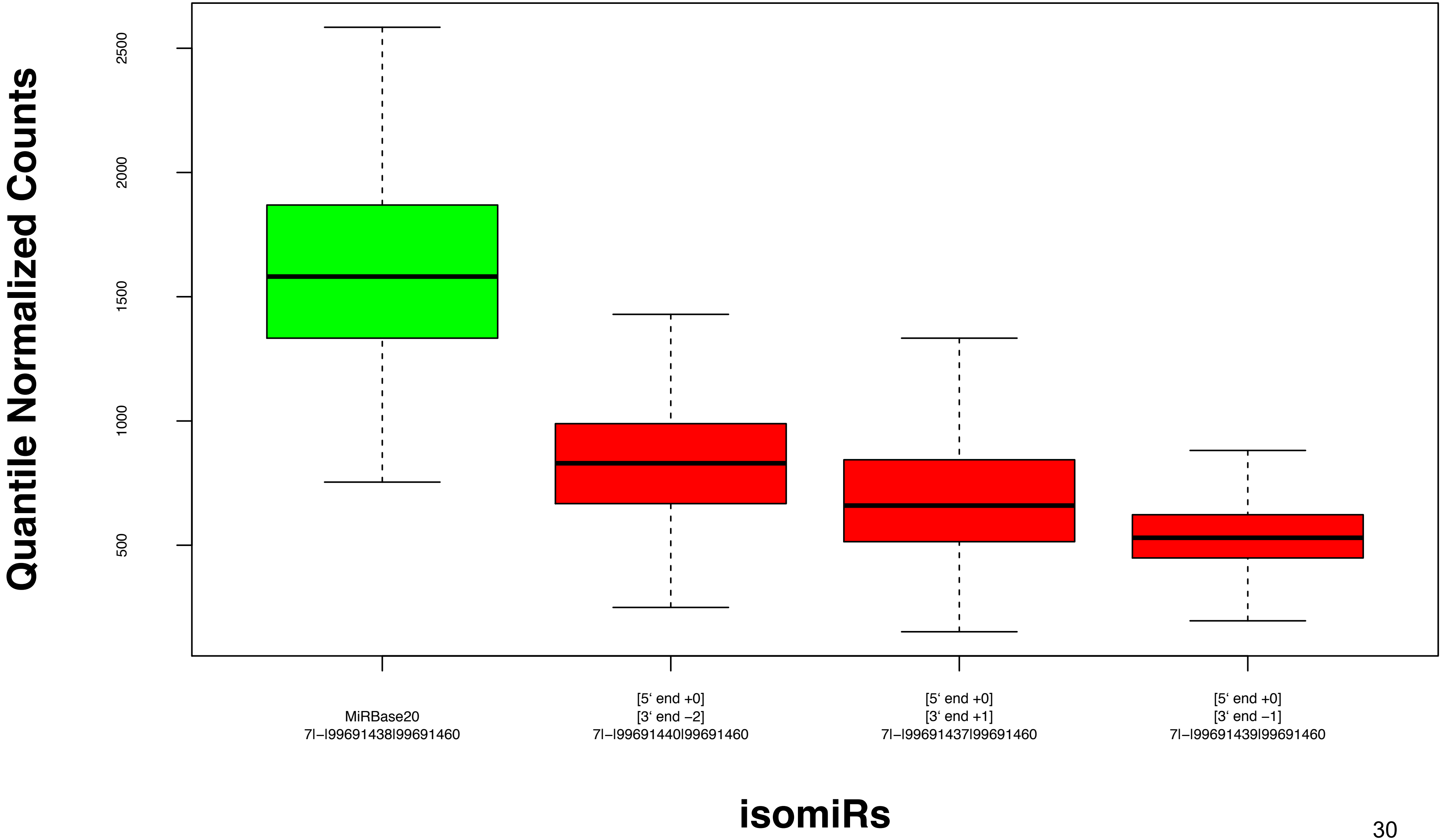

Supp. Figure 1
(pages 1 to 194)

MIMAT0000267=hsa−miR−210−3p=MI0000286

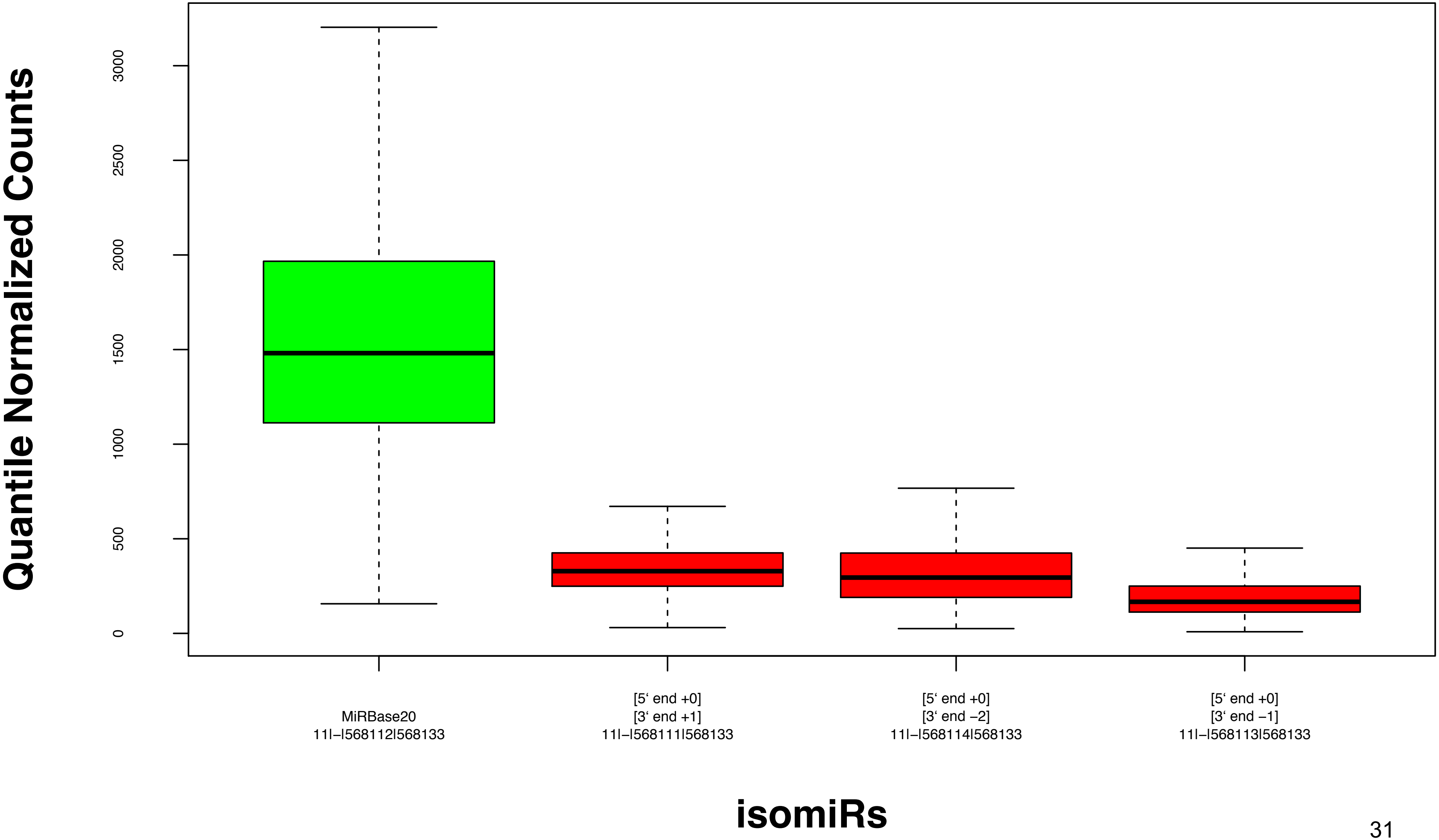

Supp. Figure 1
(pages 1 to 194)

MIMAT0000086=hsa−miR−29a−3p=MI0000087

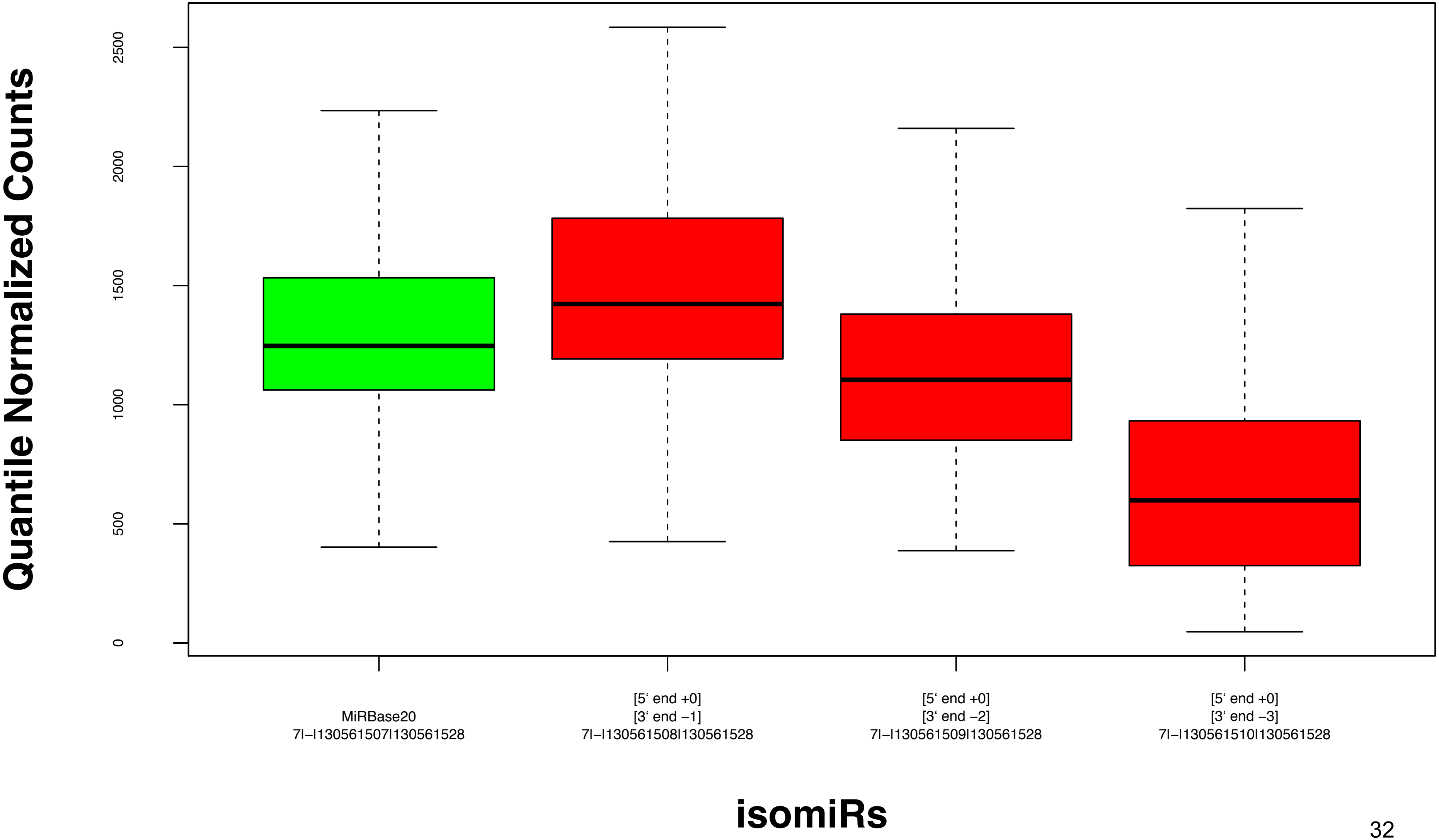

Supp. Figure 1
(pages 1 to 194)

MIMAT0000084=hsa−miR−27a−3p=MI0000085

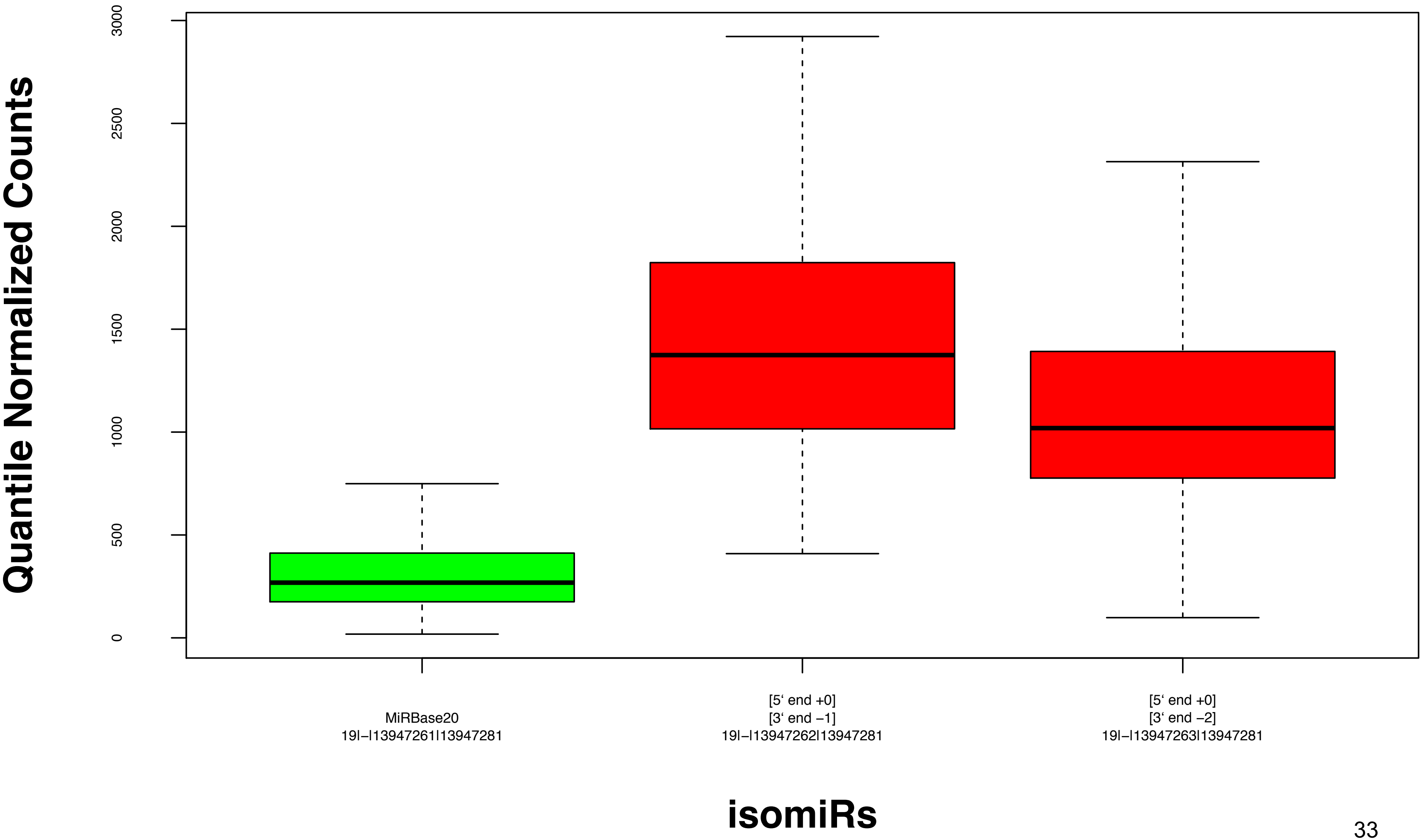

Supp. Figure 1
(pages 1 to 194)

MIMAT0000070=hsa–miR–17–5p=MI0000071

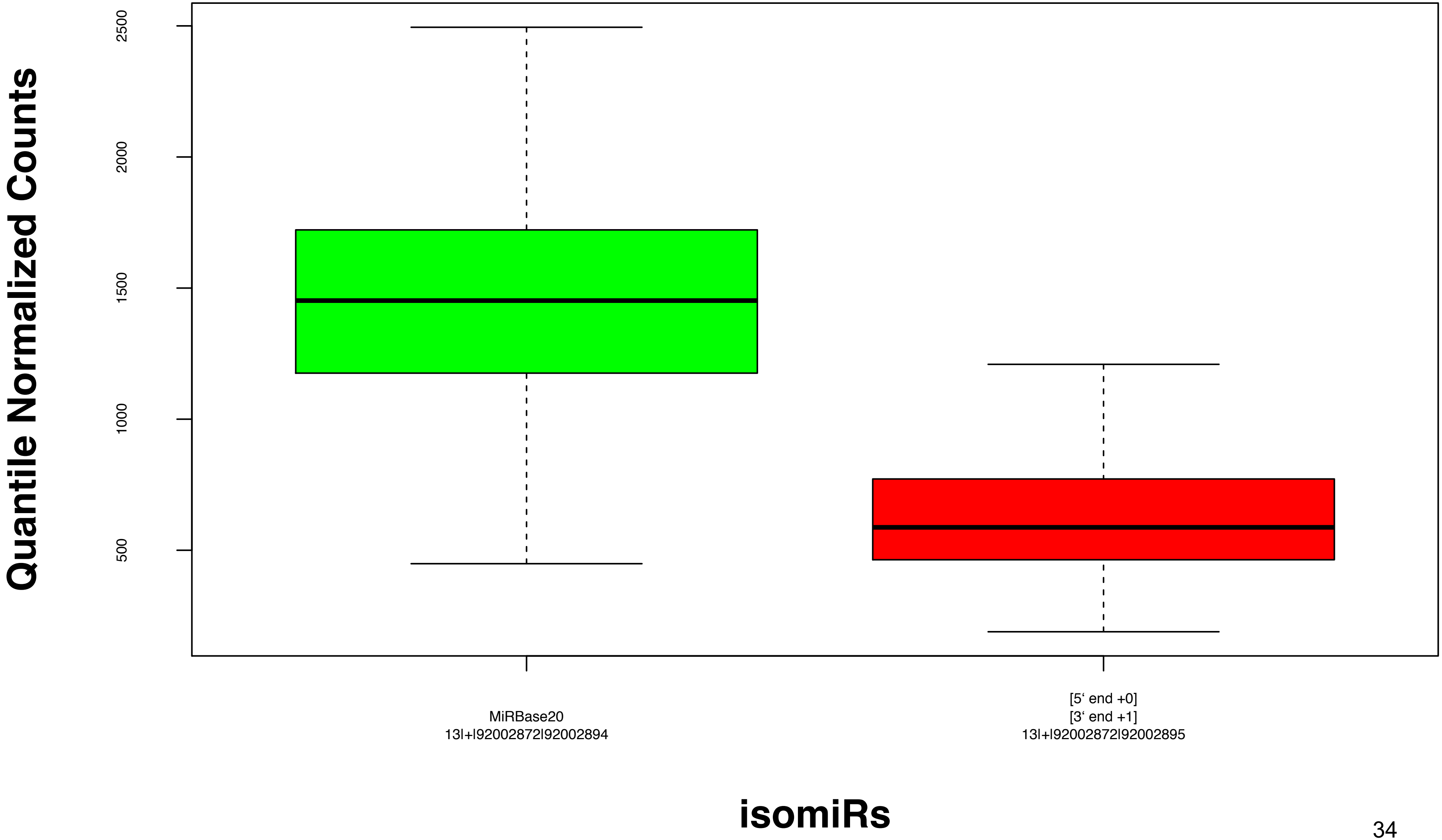

Supp. Figure 1
(pages 1 to 194)

MIMAT0000270=hsa–miR–181a–3p=MI0000289

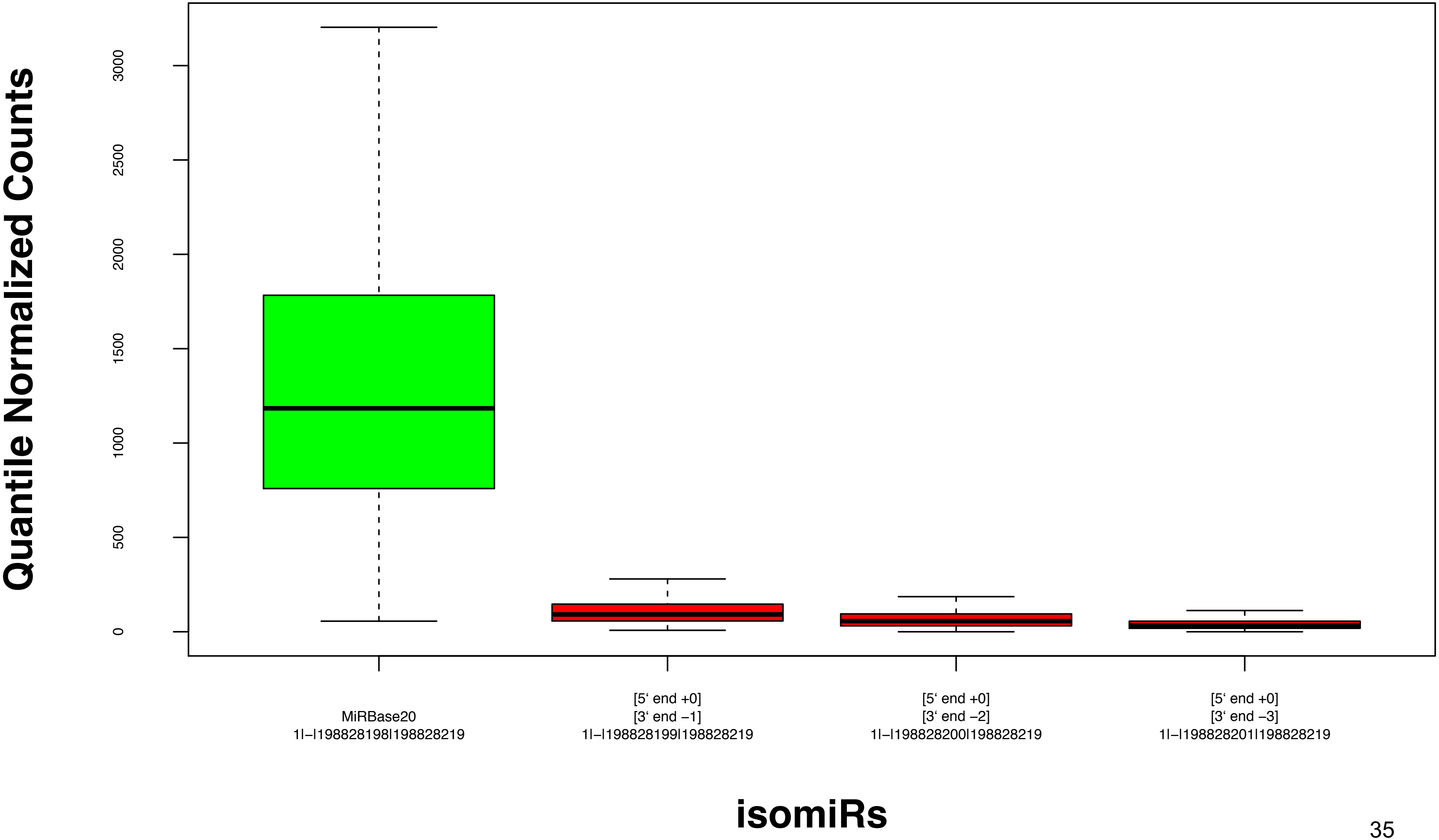

Supp. Figure 1
(pages 1 to 194)

MIMAT0003886=hsa–miR–769–5p=MI0003834

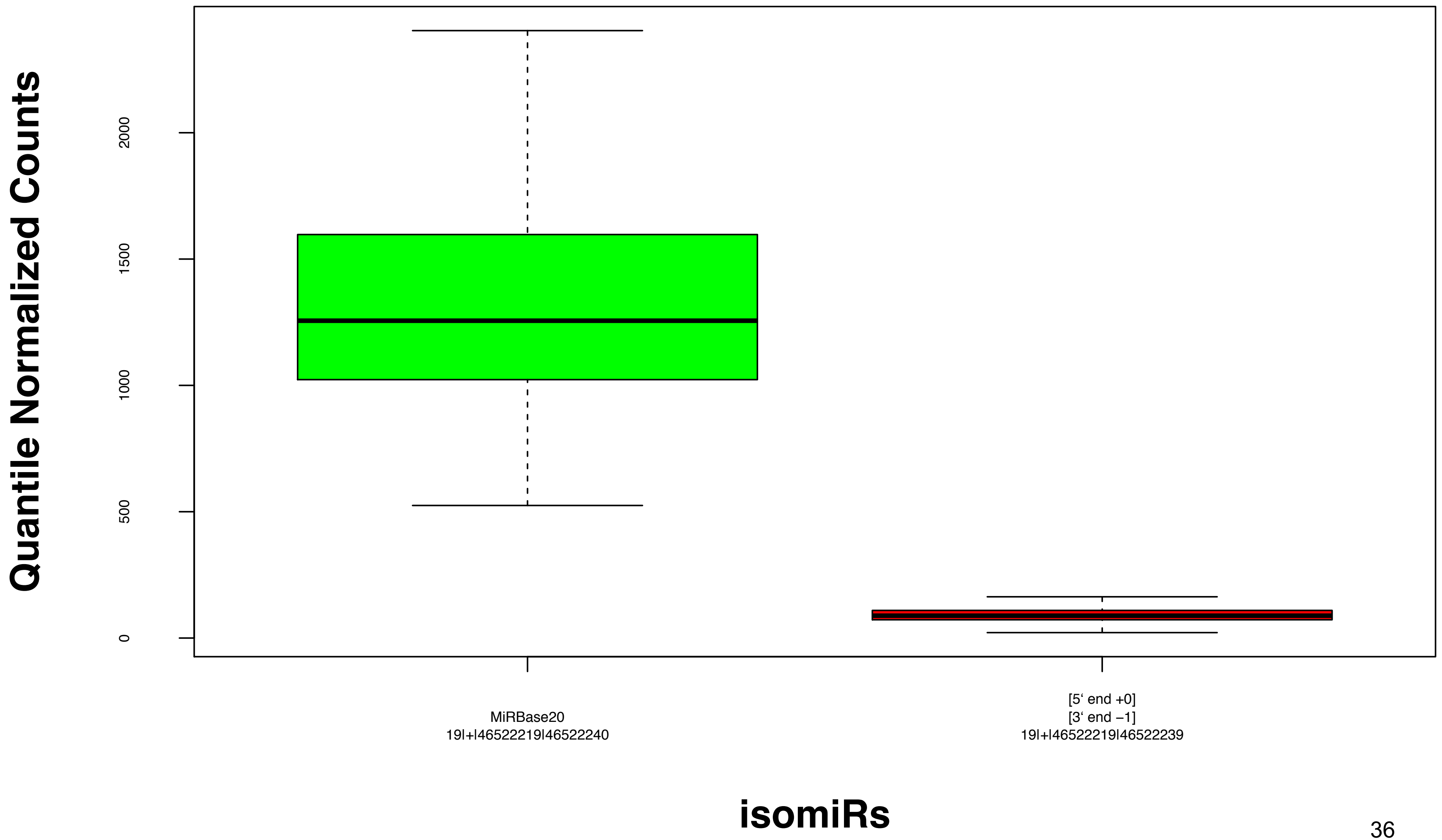

Supp. Figure 1
(pages 1 to 194)

MIMAT0000693=hsa−miR−30e−3p=MI0000749

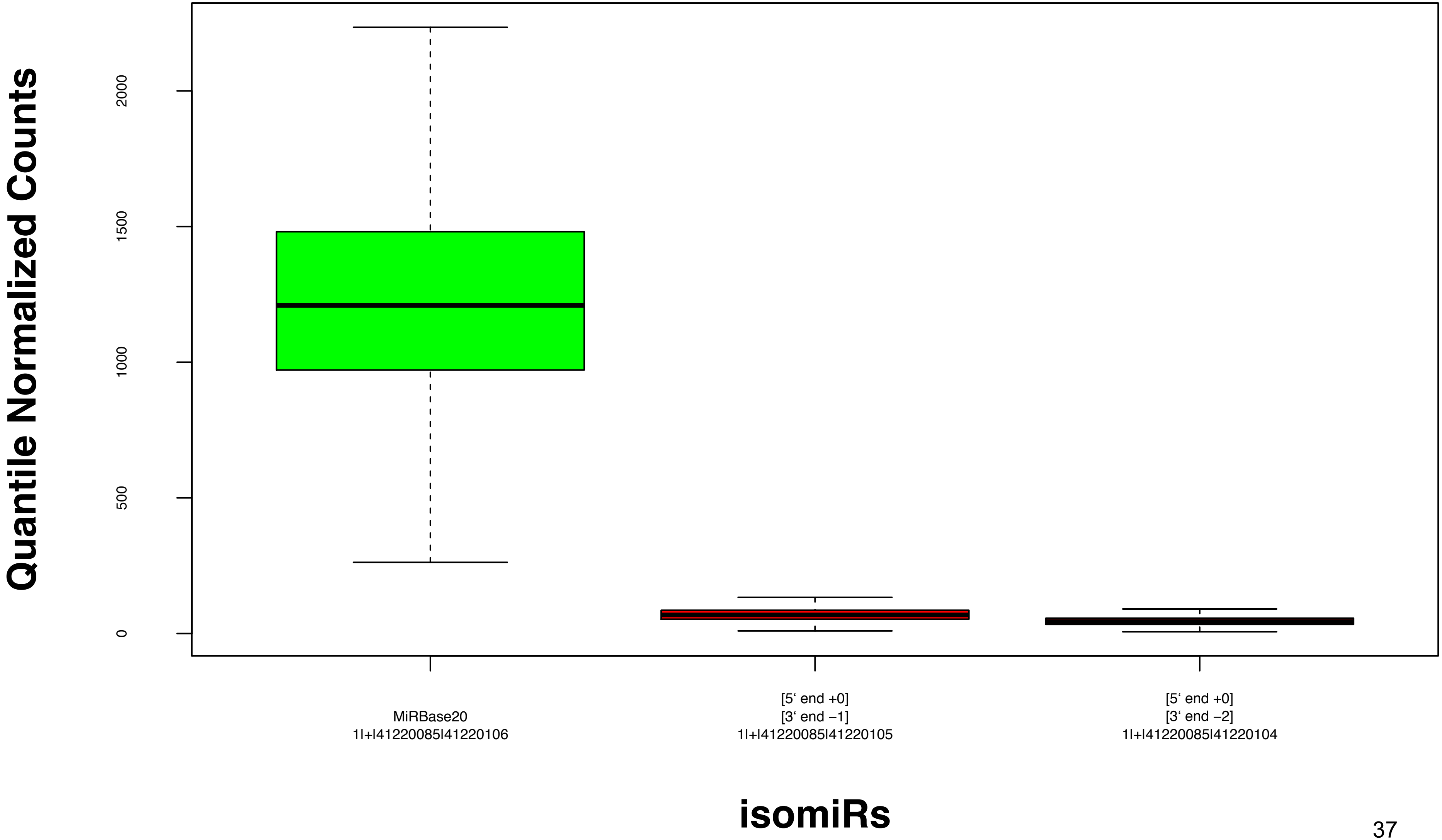

Supp. Figure 1
(pages 1 to 194)

MIMAT0000753=hsa−miR−342−3p=MI0000805

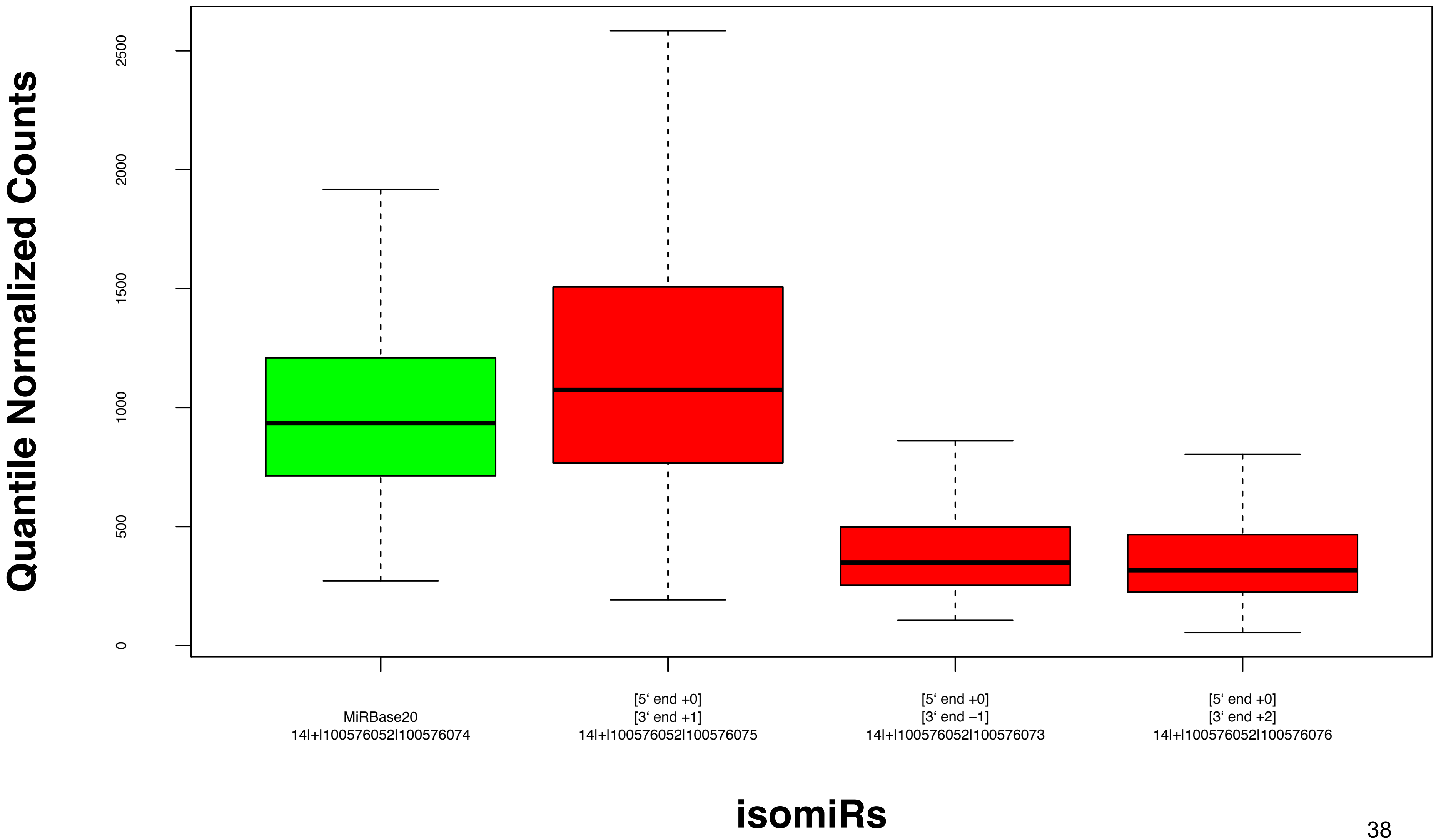

Supp. Figure 1
(pages 1 to 194)

MIMAT0000691=hsa–miR–130b–3p=MI0000748

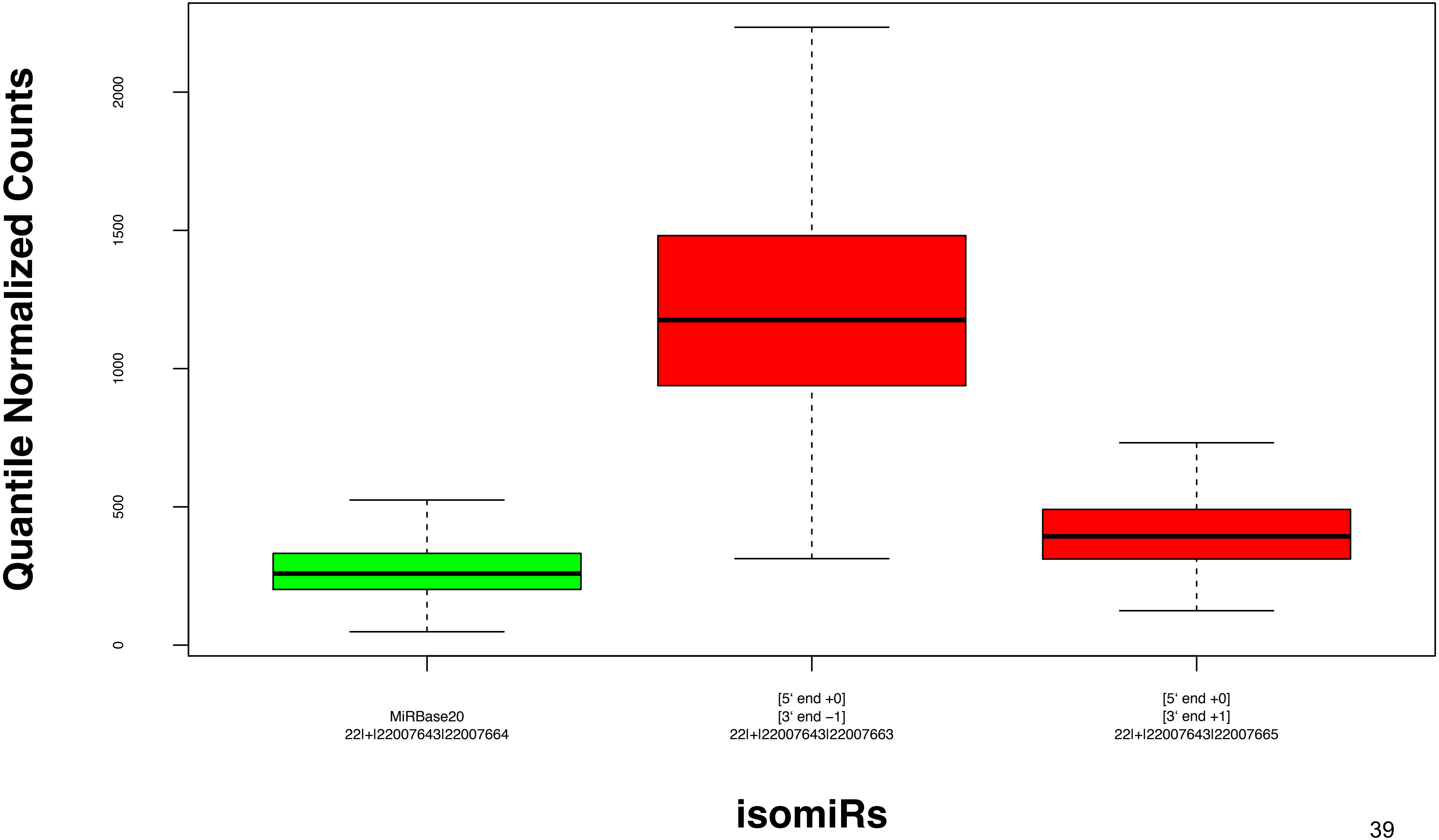

Supp. Figure 1
(pages 1 to 194)

MIMAT0000067_1=hsa−let−7f−5p=MI0000068

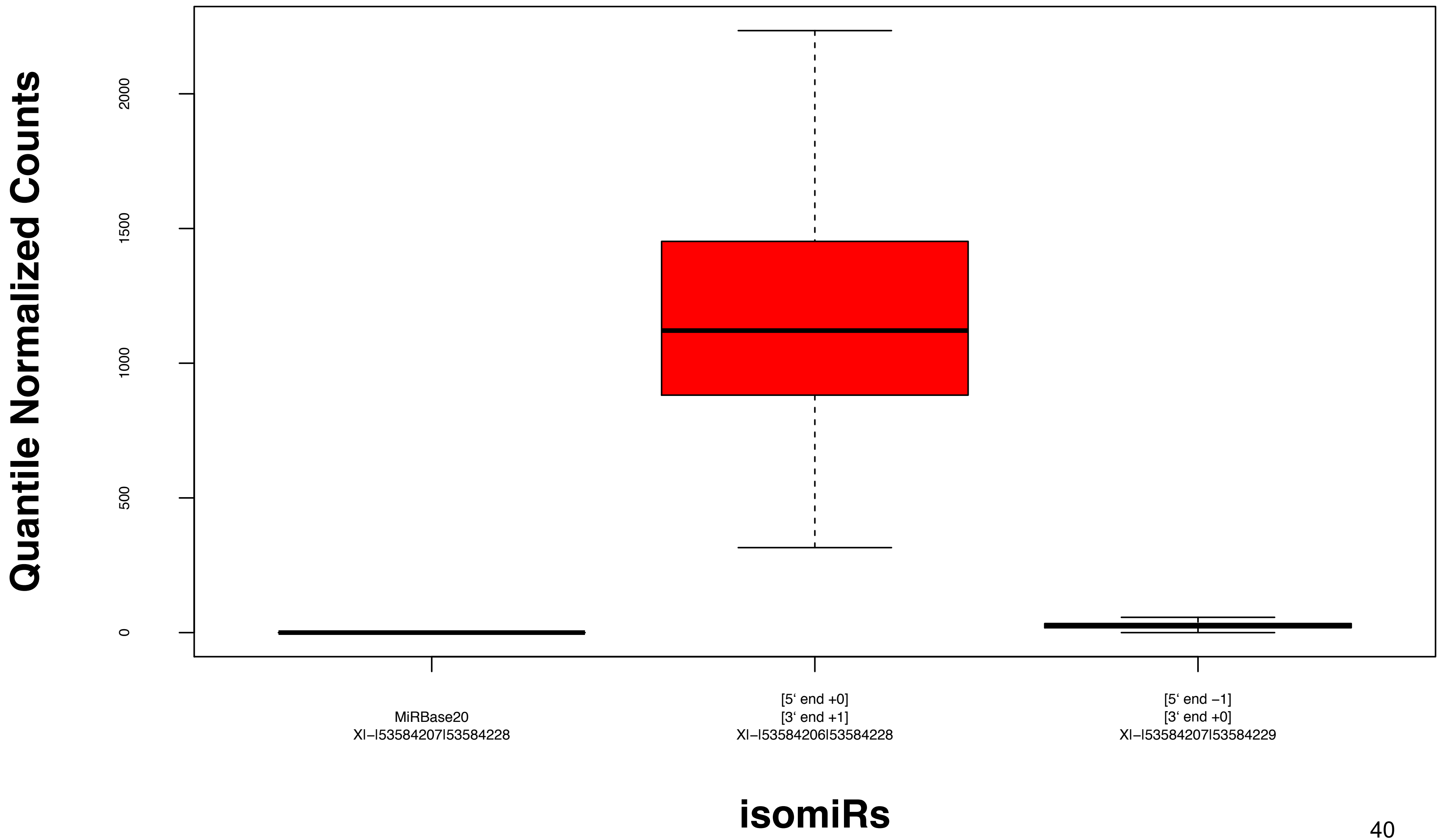

Supp. Figure 1
(pages 1 to 194)

MIMAT0000707=hsa–miR–363–3p=MI0000764

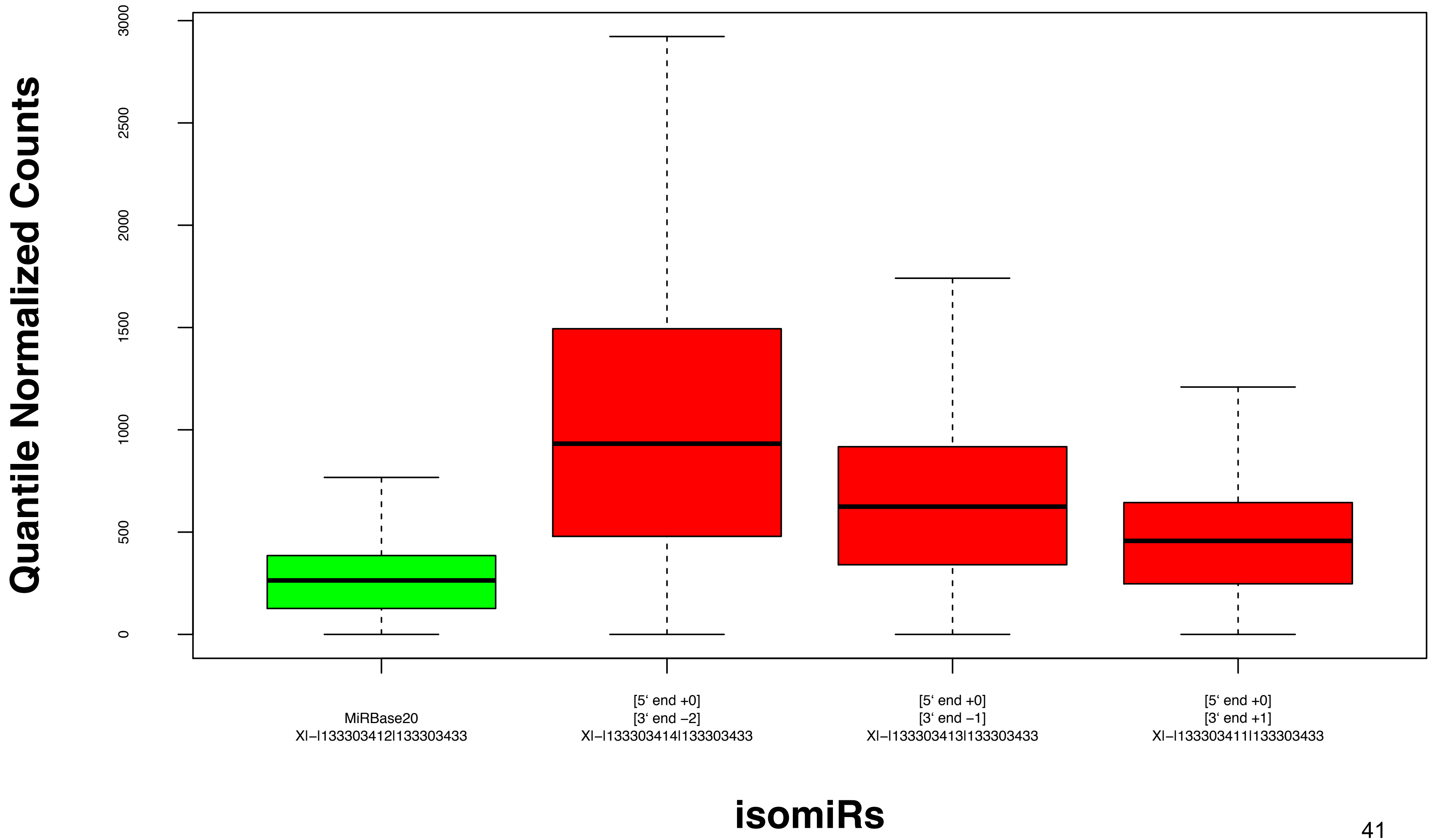

Supp. Figure 1
(pages 1 to 194)

MIMAT0003393=hsa–miR–425–5p=MI0001448

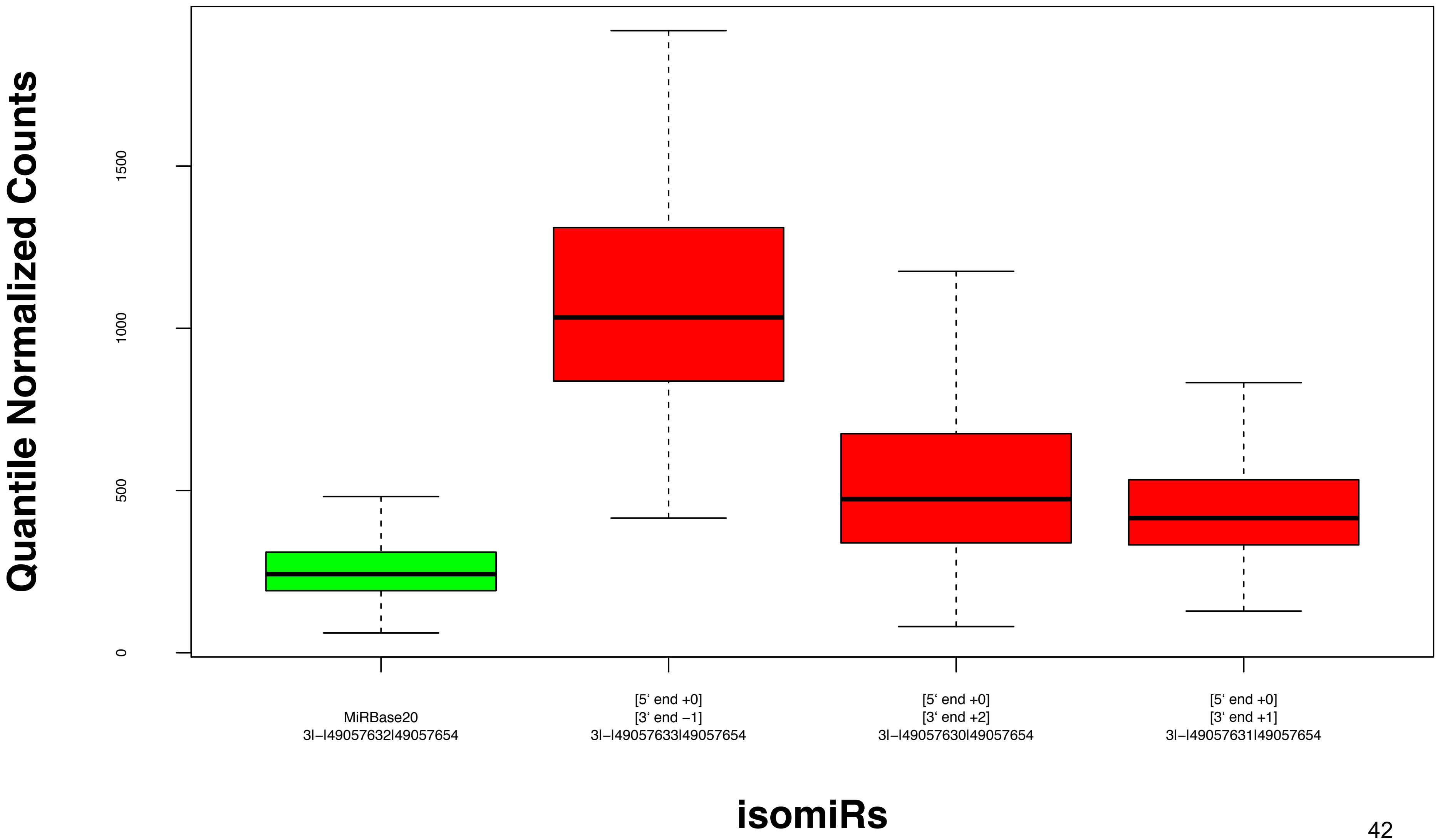

Supp. Figure 1
(pages 1 to 194)

MIMAT0000278=hsa−miR−221−3p=MI0000298

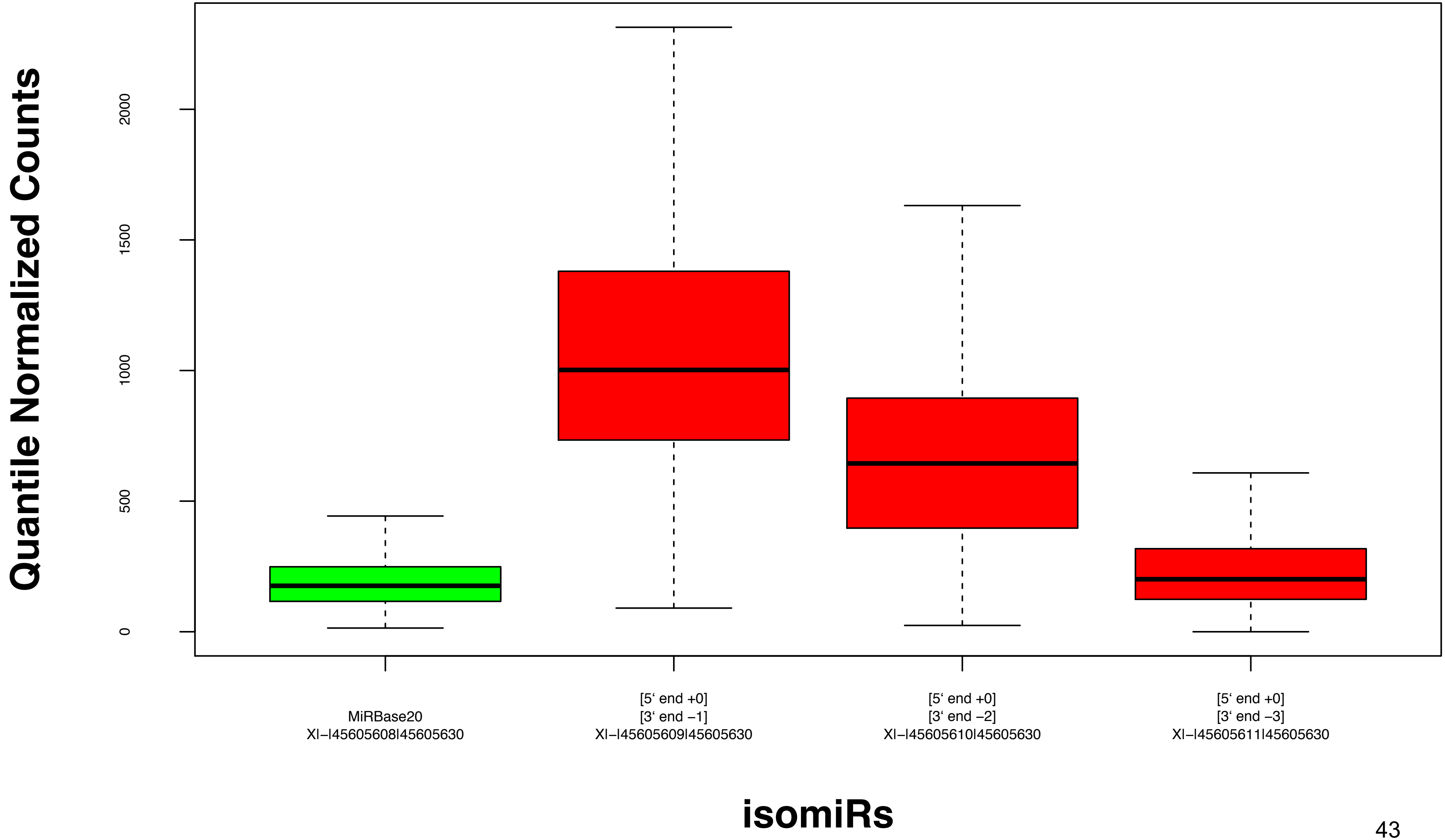

Supp. Figure 1
(pages 1 to 194)

MIMAT0003218=hsa−miR−92b−3p=MI0003560

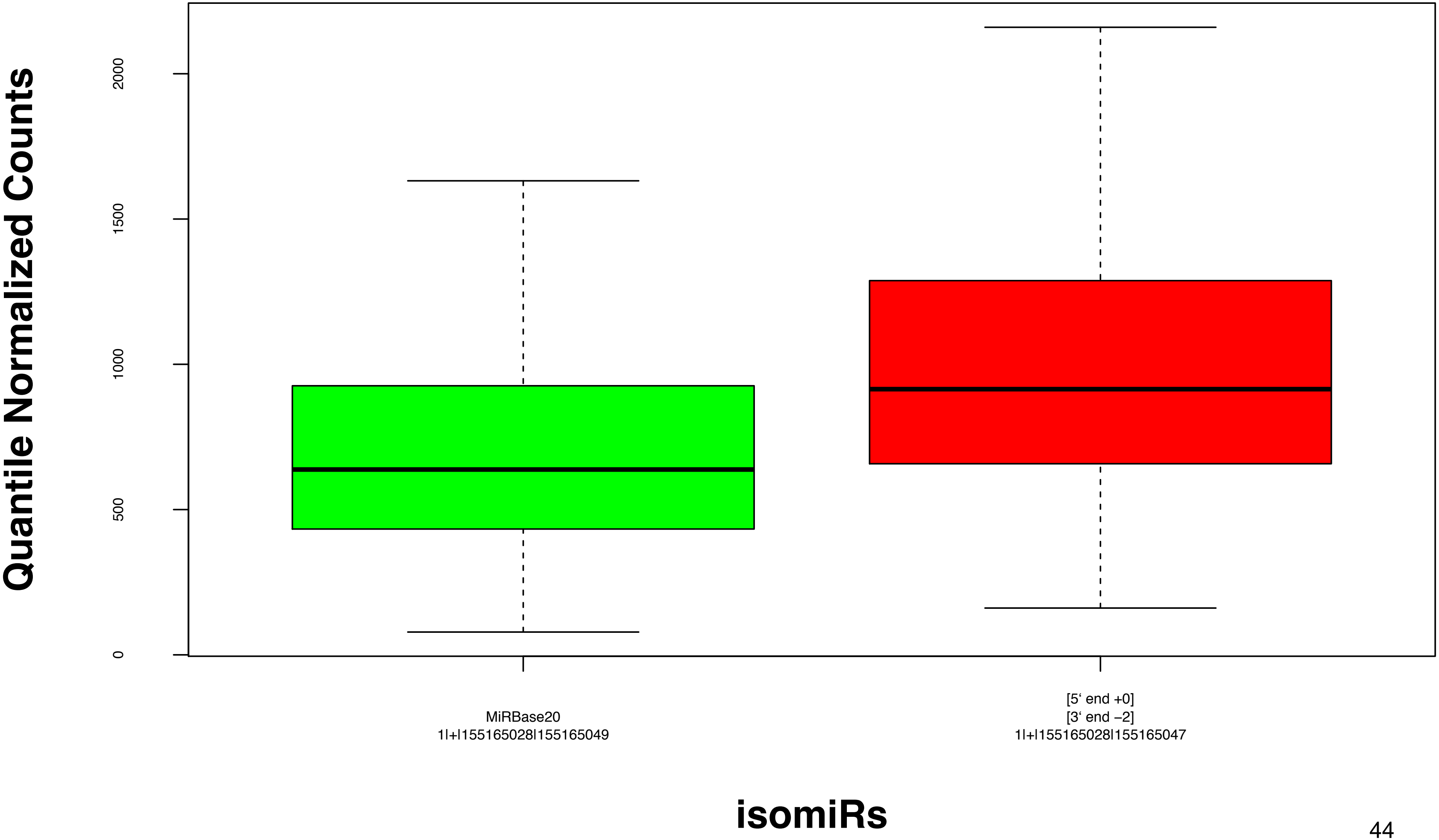

Supp. Figure 1
(pages 1 to 194)

MIMAT0000510=hsa−miR−320a=MI0000542

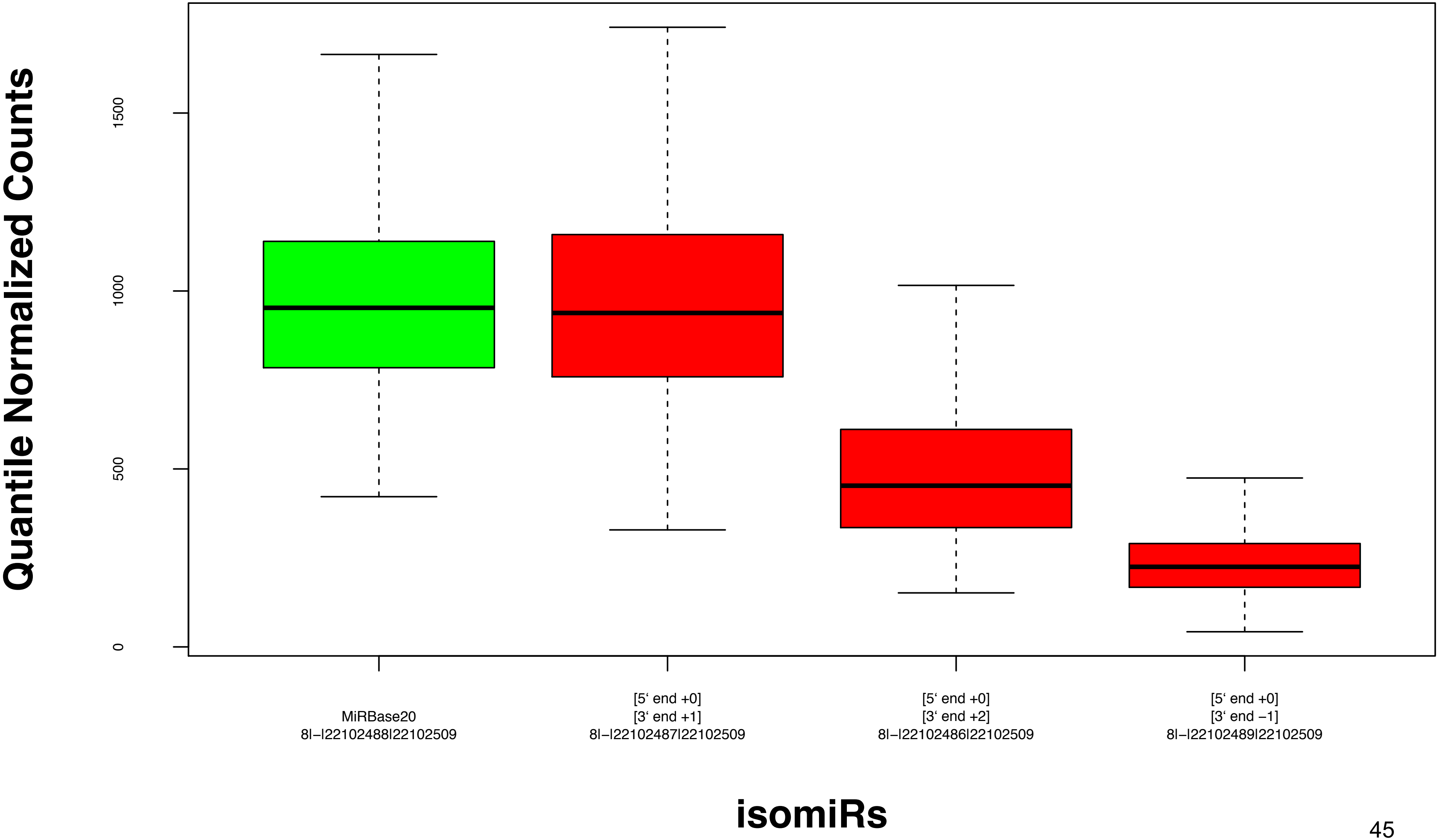

Supp. Figure 1
(pages 1 to 194)

MIMAT0000759=hsa–miR–148b–3p=MI0000811

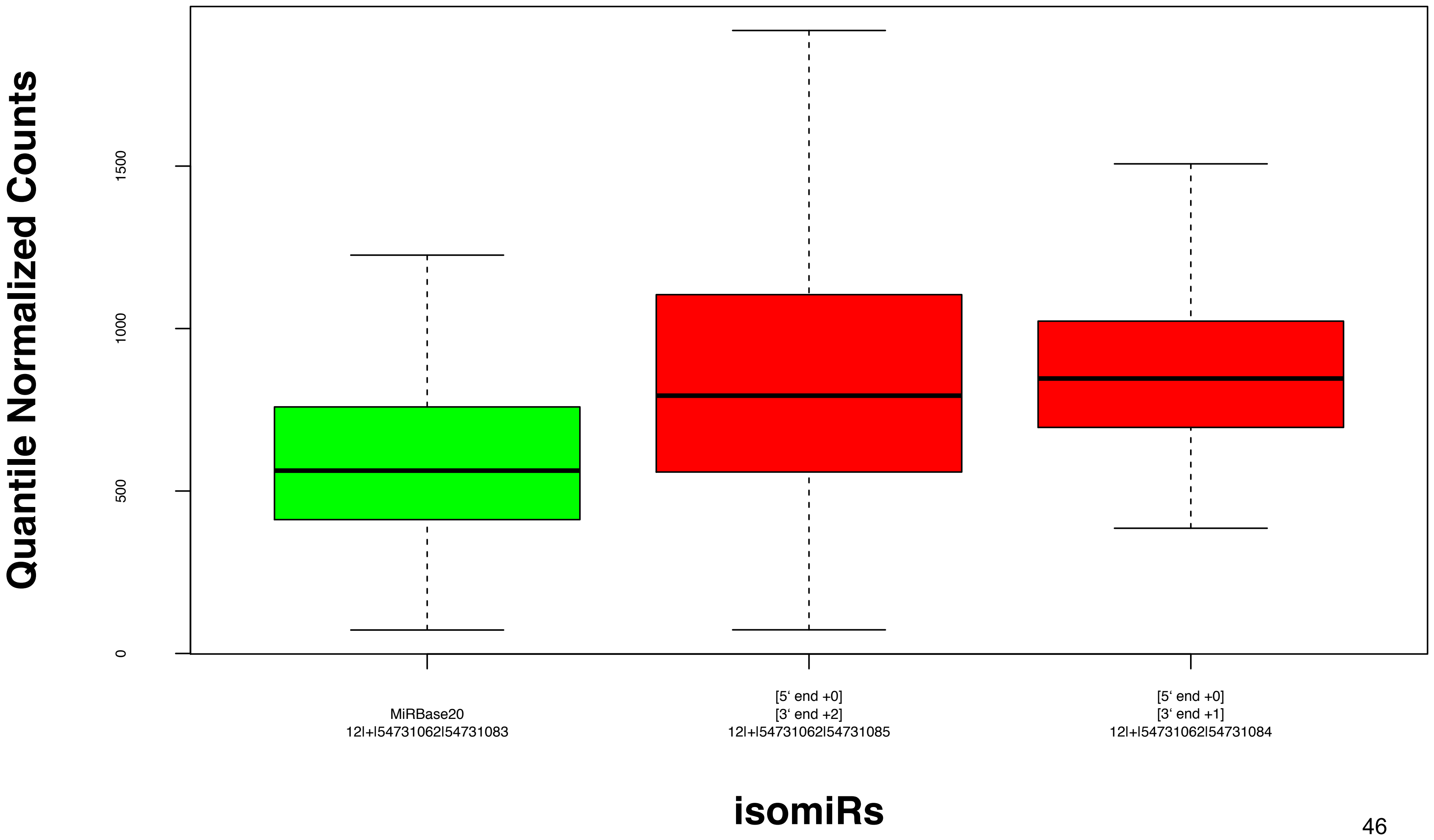

Supp. Figure 1
(pages 1 to 194)

MIMAT0004558=hsa−miR−181a−2−3p=MI0000269

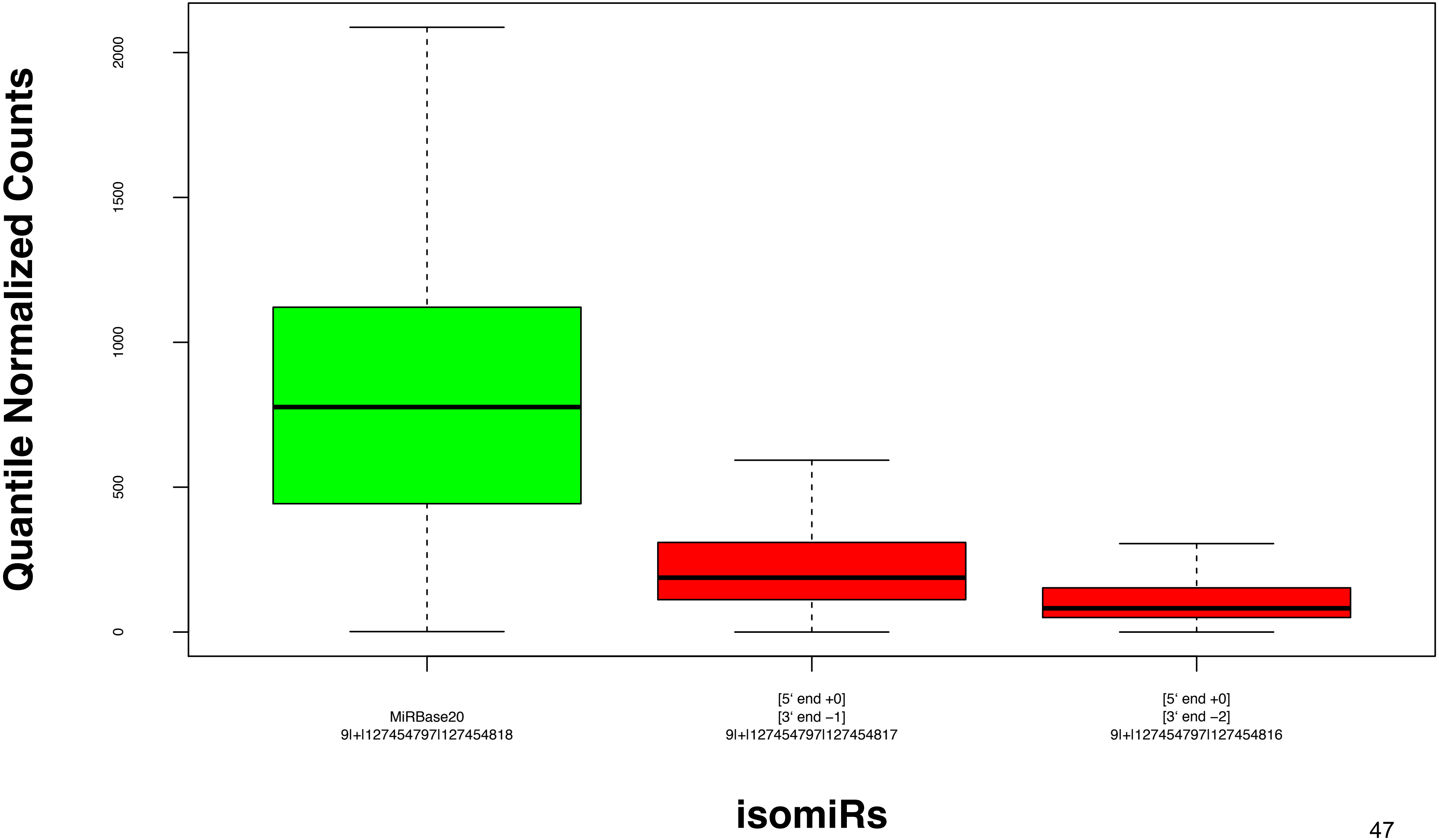

Supp. Figure 1
(pages 1 to 194)

MIMAT0000259=hsa−miR−182−5p=MI0000272

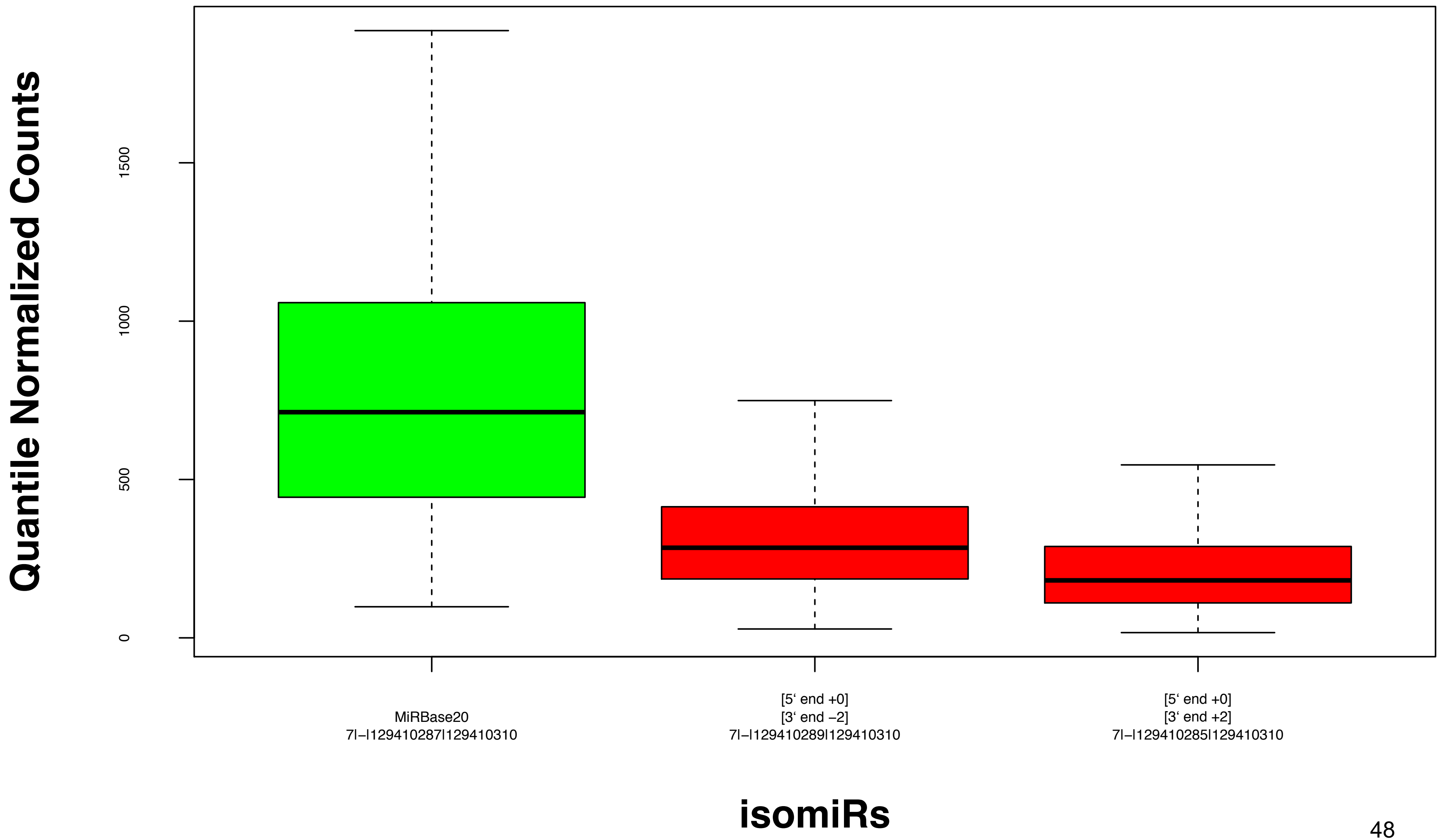

Supp. Figure 1
(pages 1 to 194)

MIMAT0000757=hsa−miR−151a−3p=MI0000809

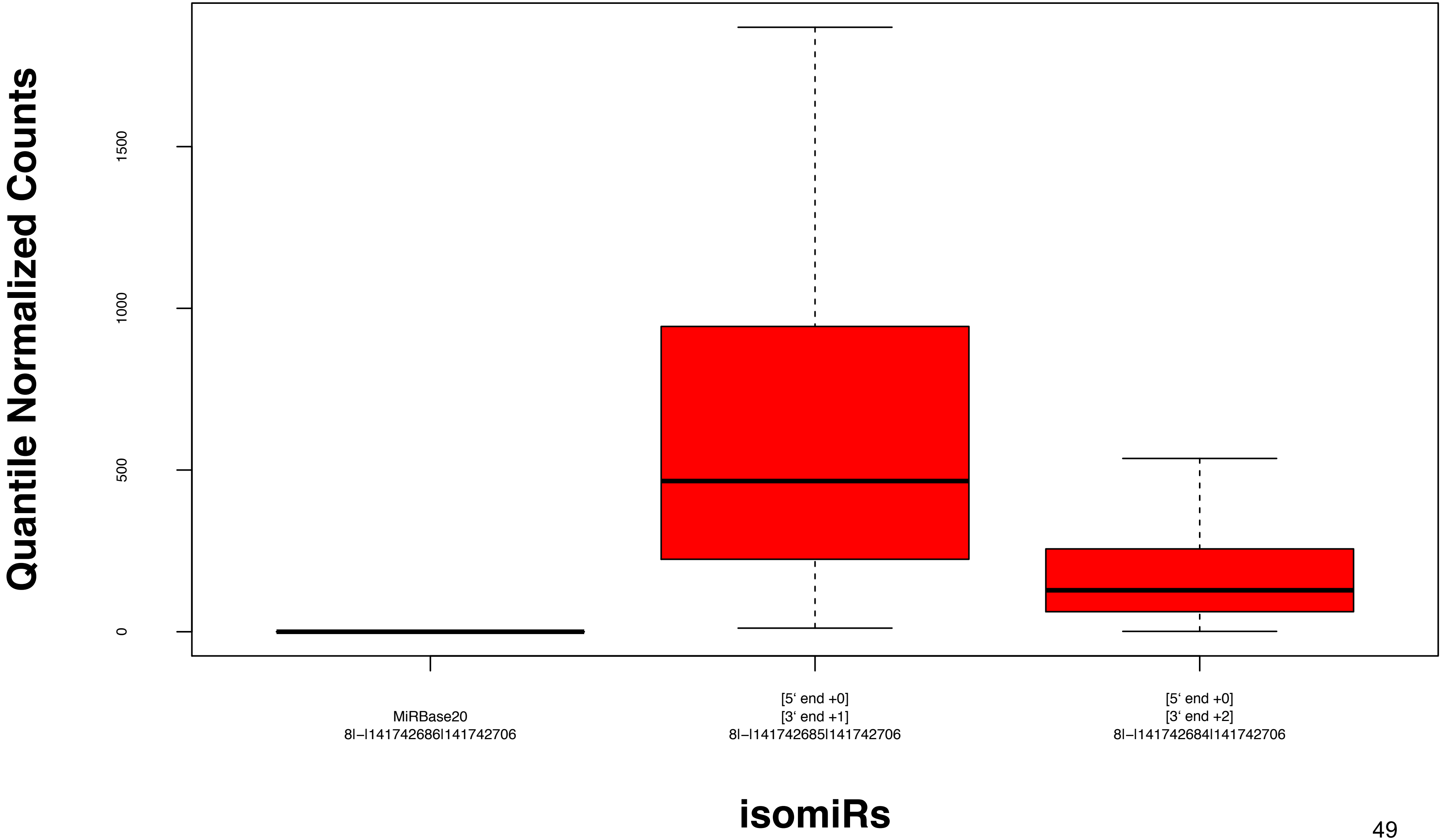

Supp. Figure 1
(pages 1 to 194)

MIMAT0000258=hsa–miR–181c–5p=MI0000271

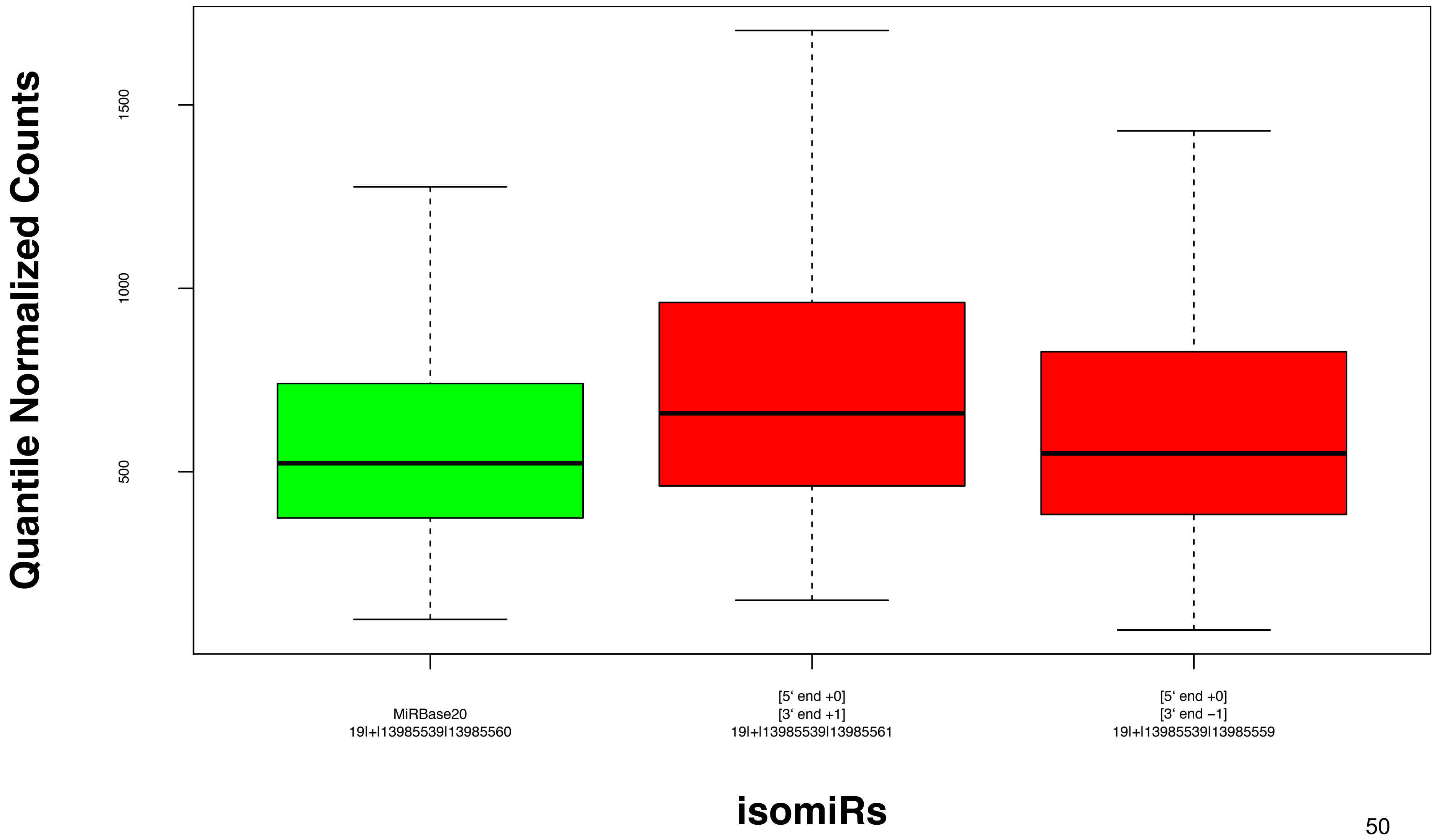

Supp. Figure 1
(pages 1 to 194)

MIMAT0000279=hsa−miR−222−3p=MI0000299

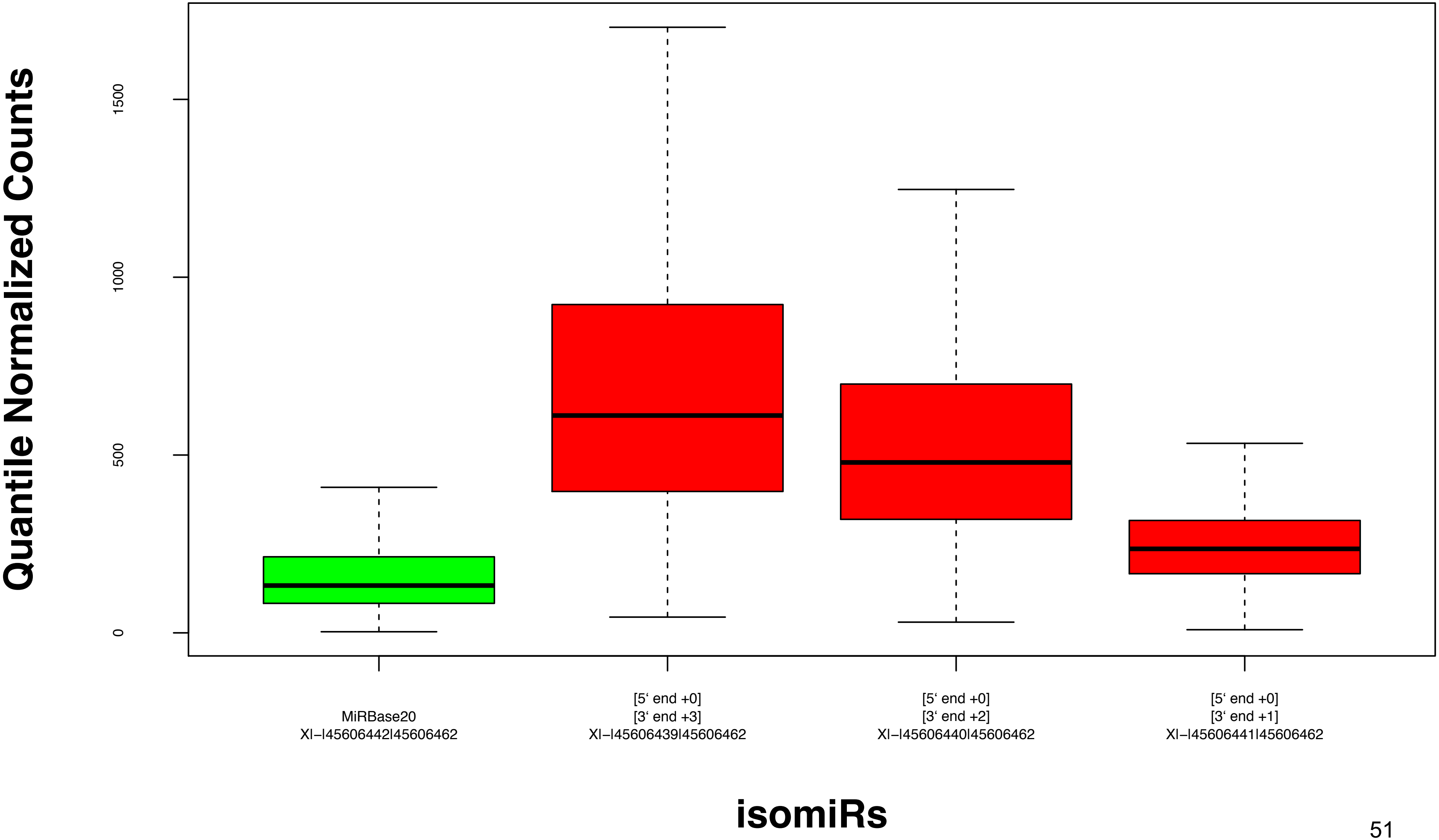

Supp. Figure 1
(pages 1 to 194)

MIMAT0004672=hsa–miR–106b–3p=MI0000734

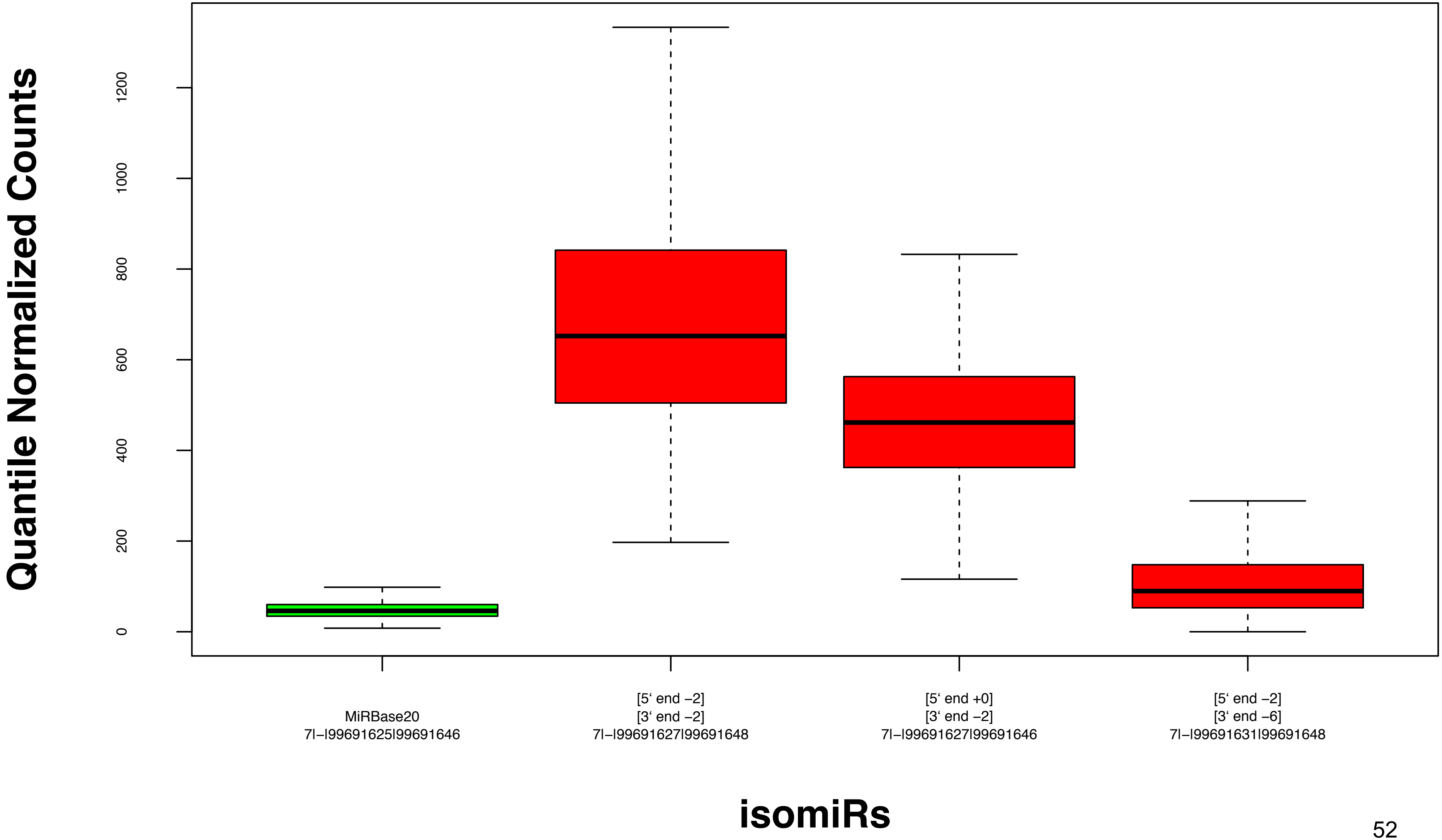

Supp. Figure 1
(pages 1 to 194)

MIMAT0004597=hsa–miR–140–3p=MI0000456

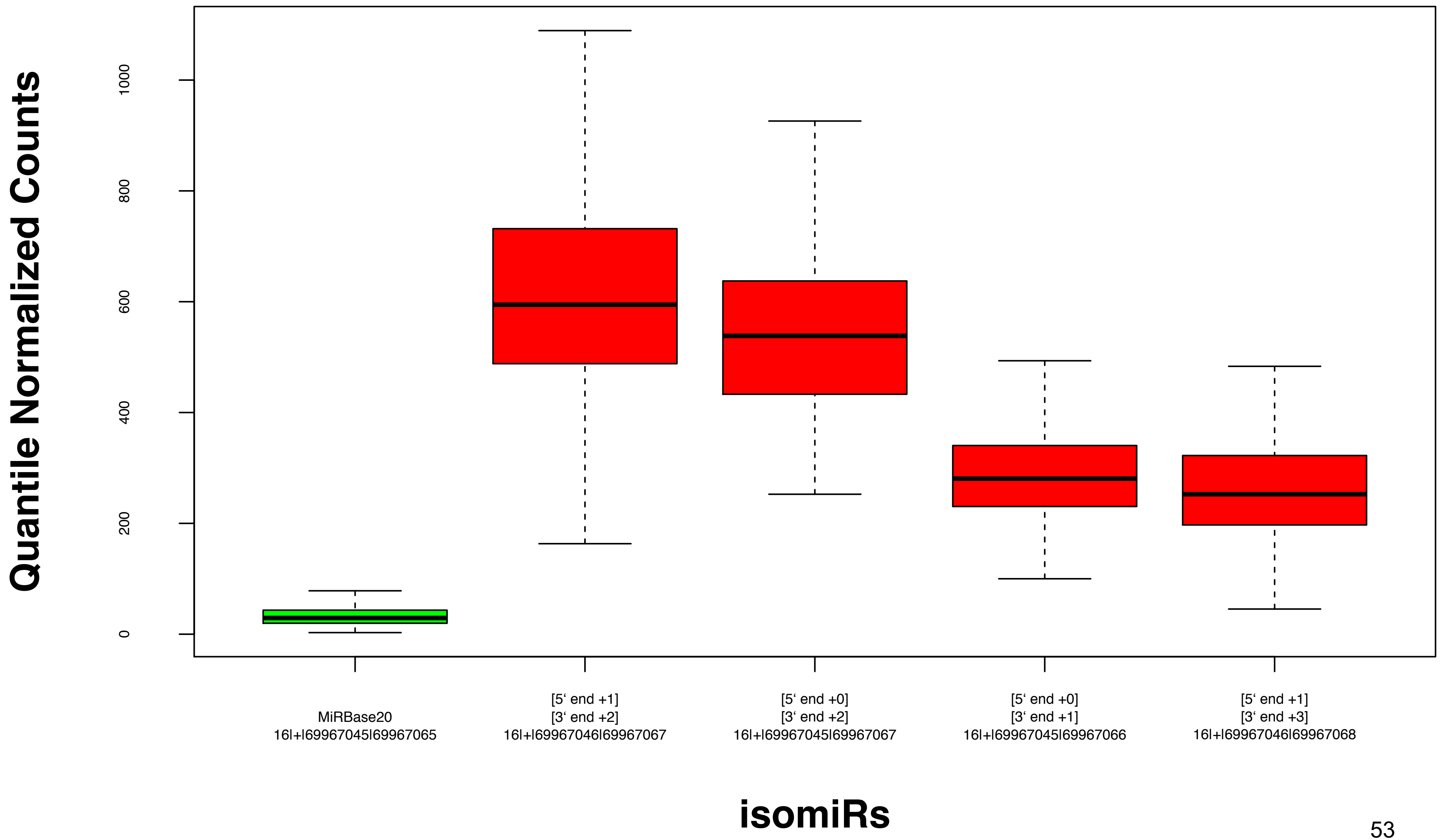

Supp. Figure 1
(pages 1 to 194)

MIMAT0004702=hsa−miR−339−3p=MI0000815

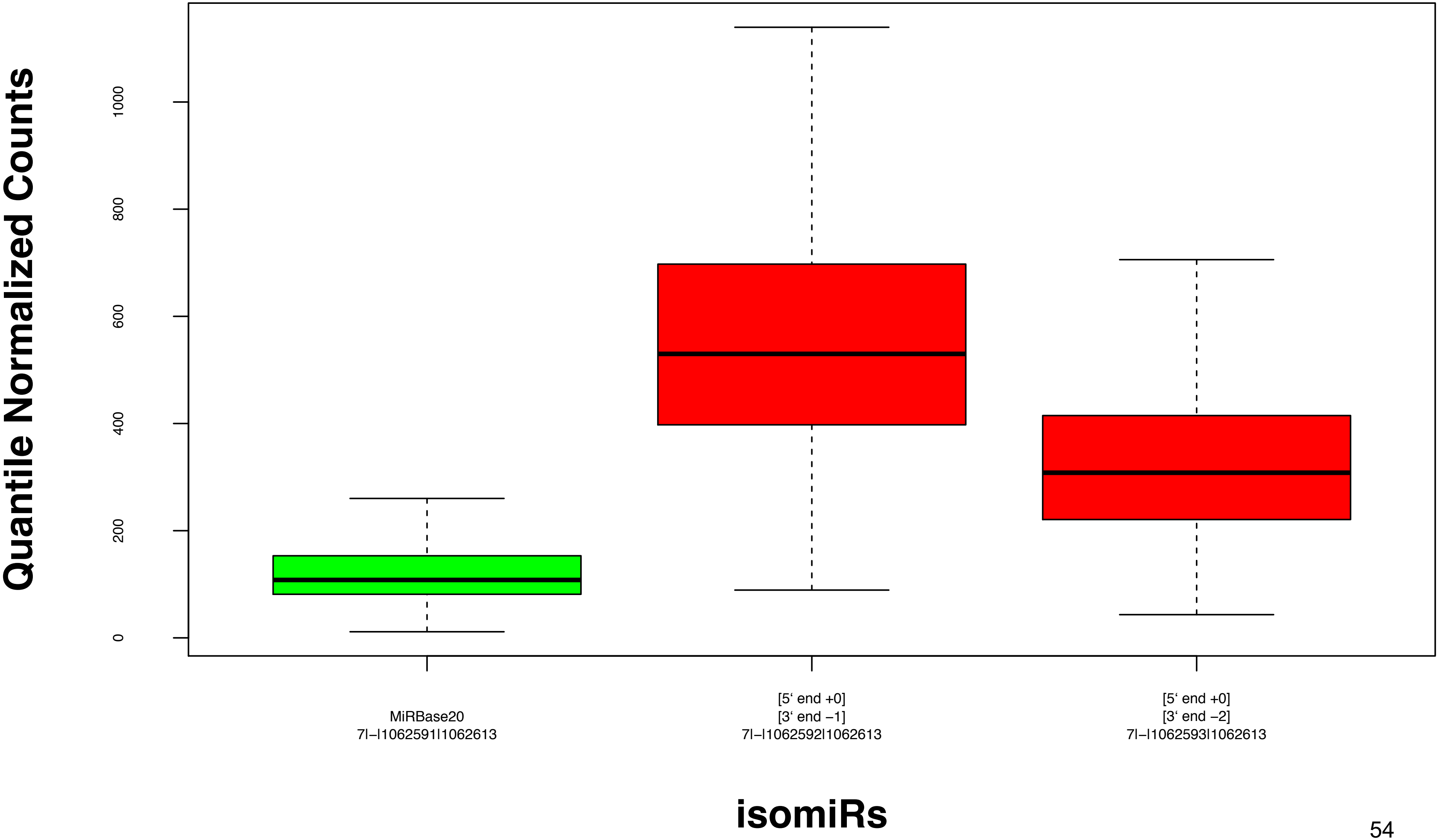

Supp. Figure 1
(pages 1 to 194)

MIMAT0000434=hsa−miR−142−3p=MI0000458

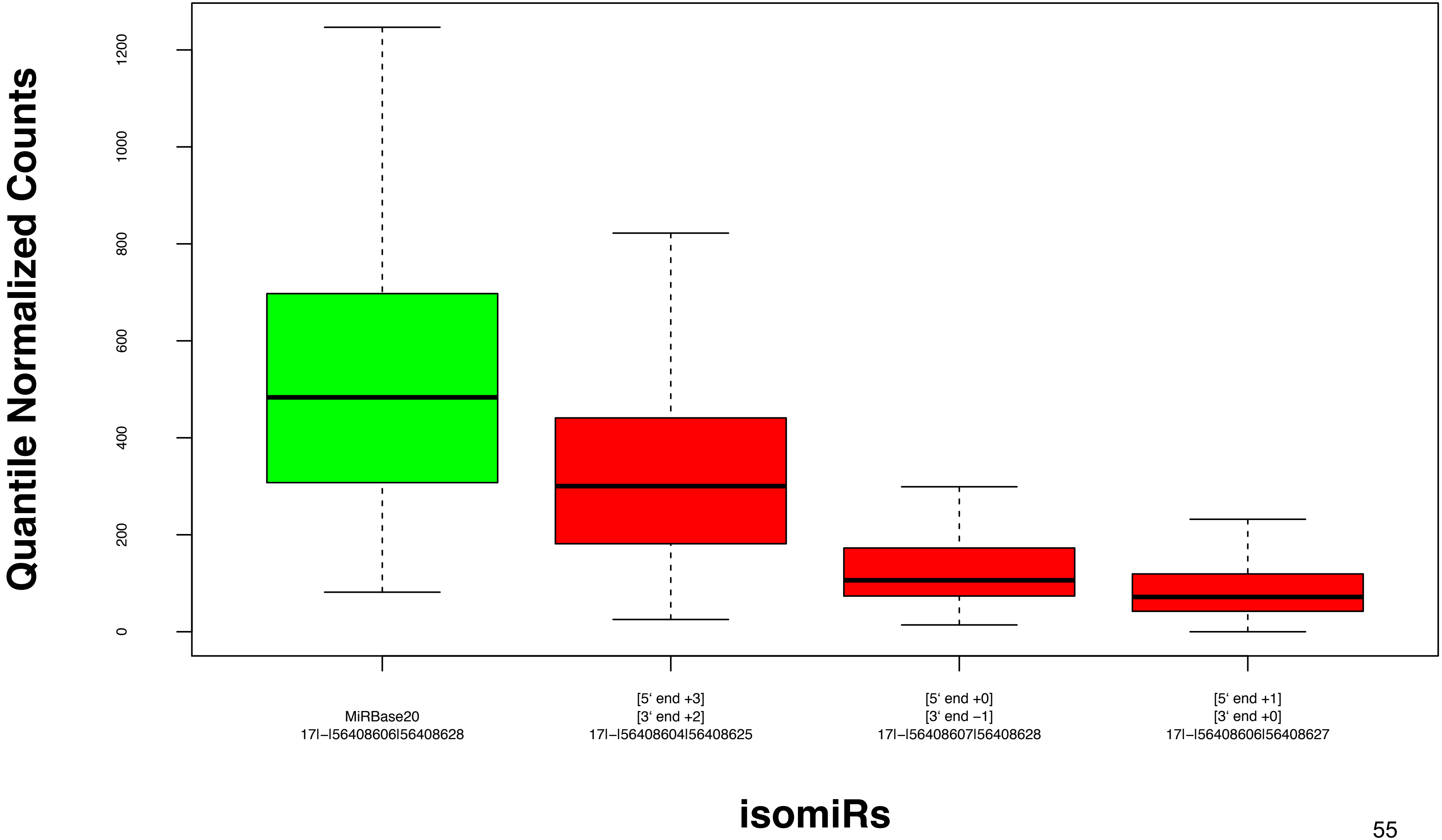

Supp. Figure 1
(pages 1 to 194)

MIMAT0000435=hsa–miR–143–3p=MI0000459

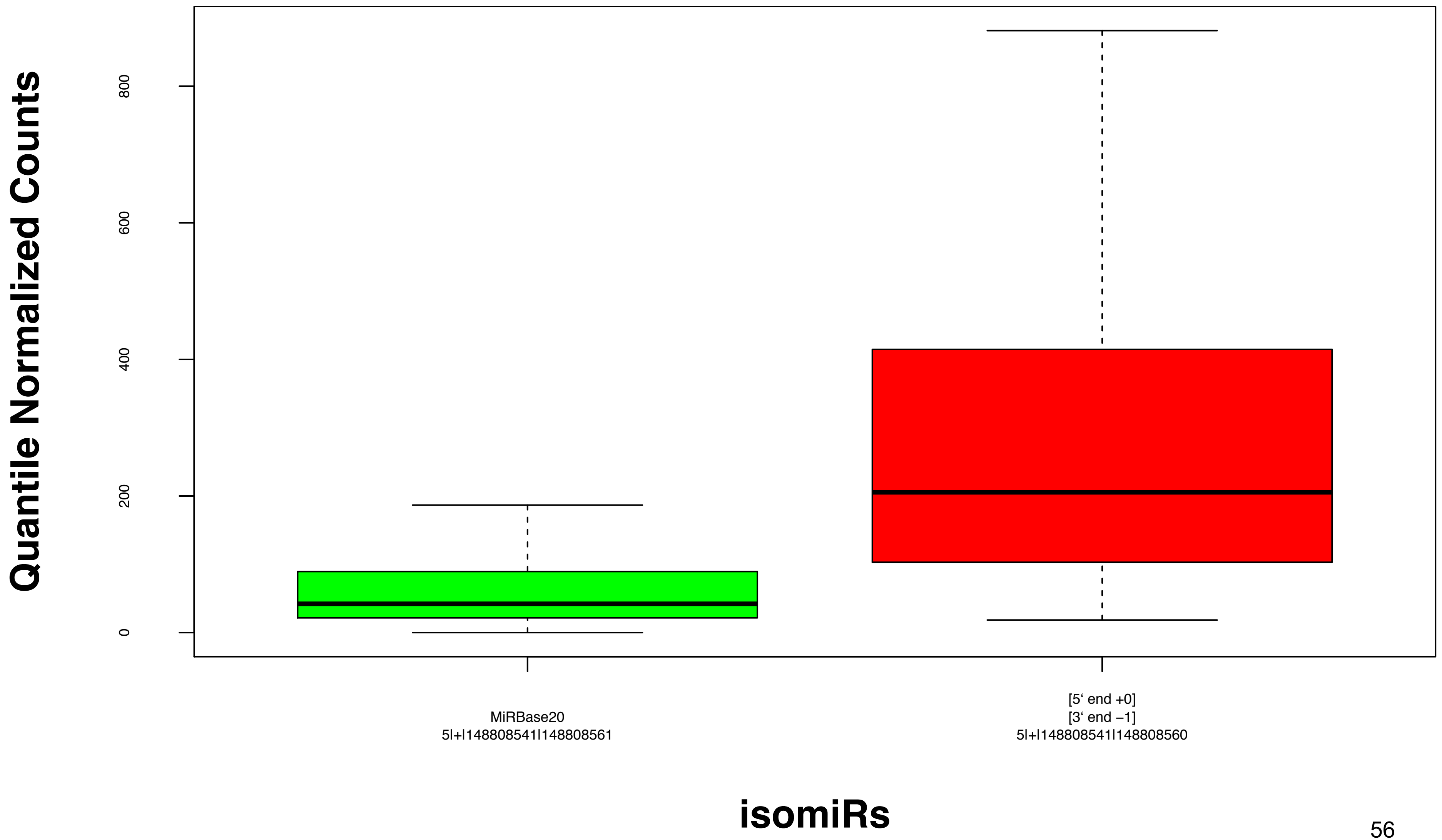

Supp. Figure 1
(pages 1 to 194)

MIMAT0000255=hsa–miR–34a–5p=MI0000268

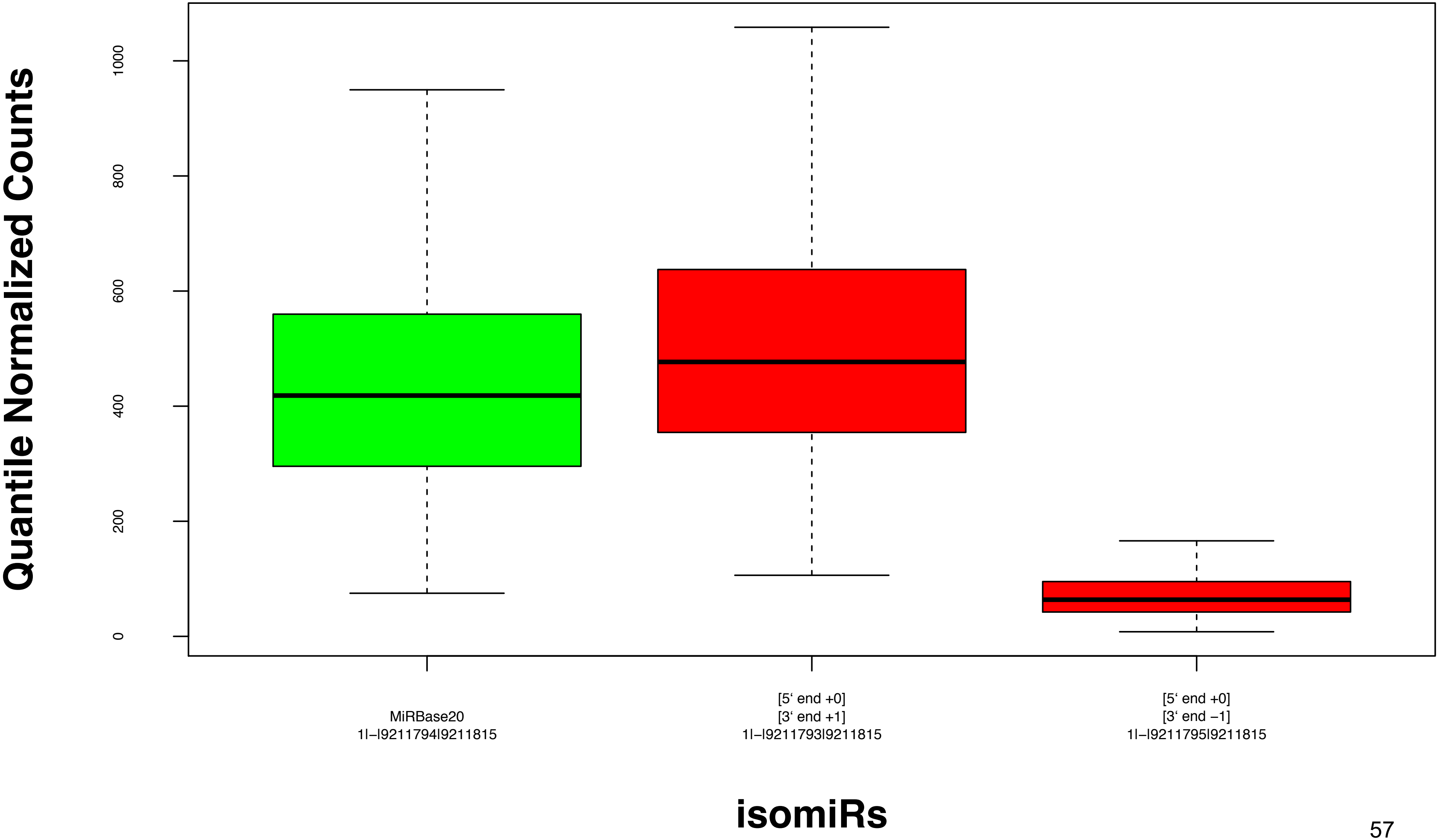

Supp. Figure 1
(pages 1 to 194)

MIMAT0030021=hsa−miR−7706=MI0025242

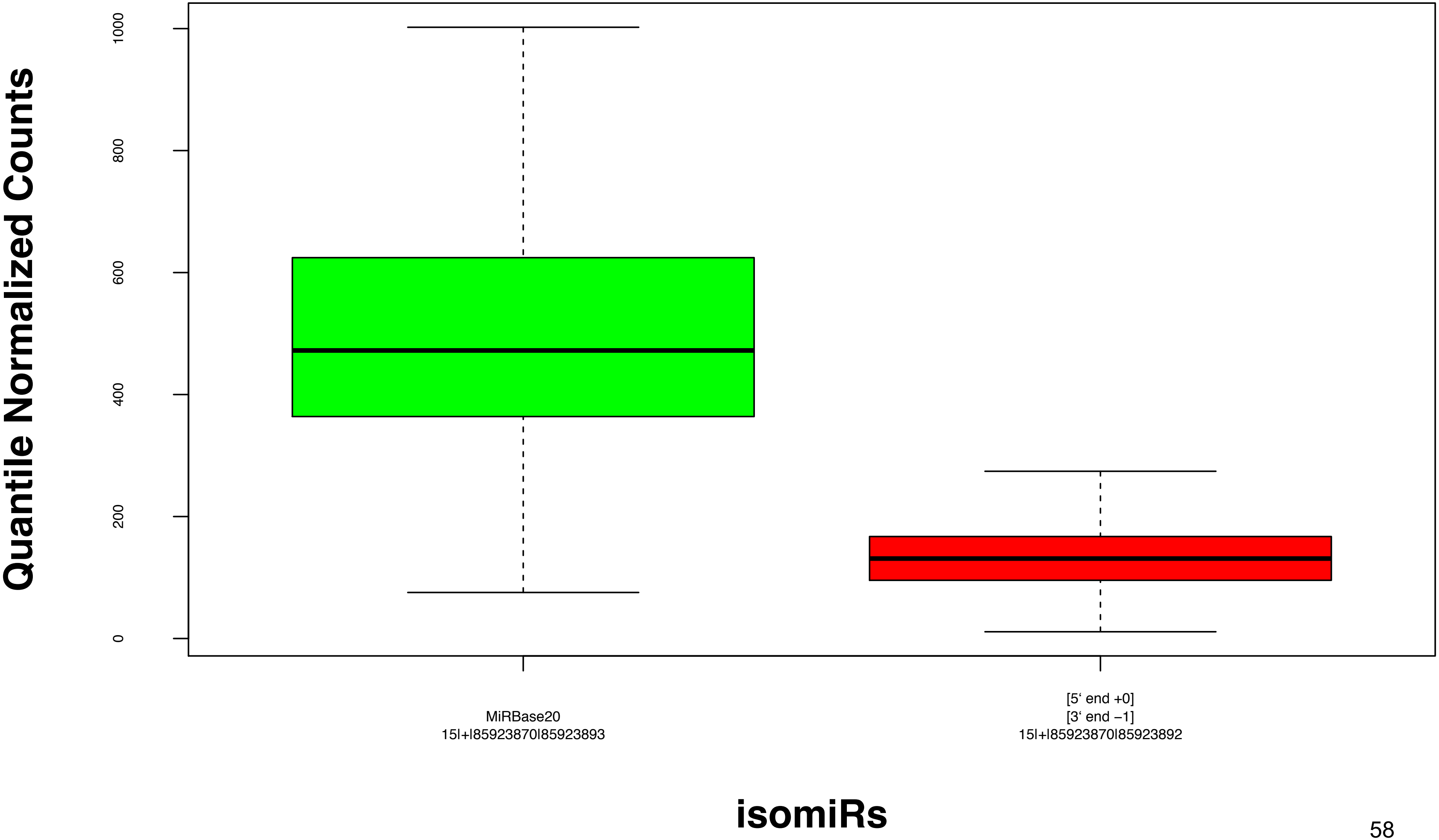

Supp. Figure 1
(pages 1 to 194)

MIMAT0004682=hsa−miR−361−3p=MI0000760

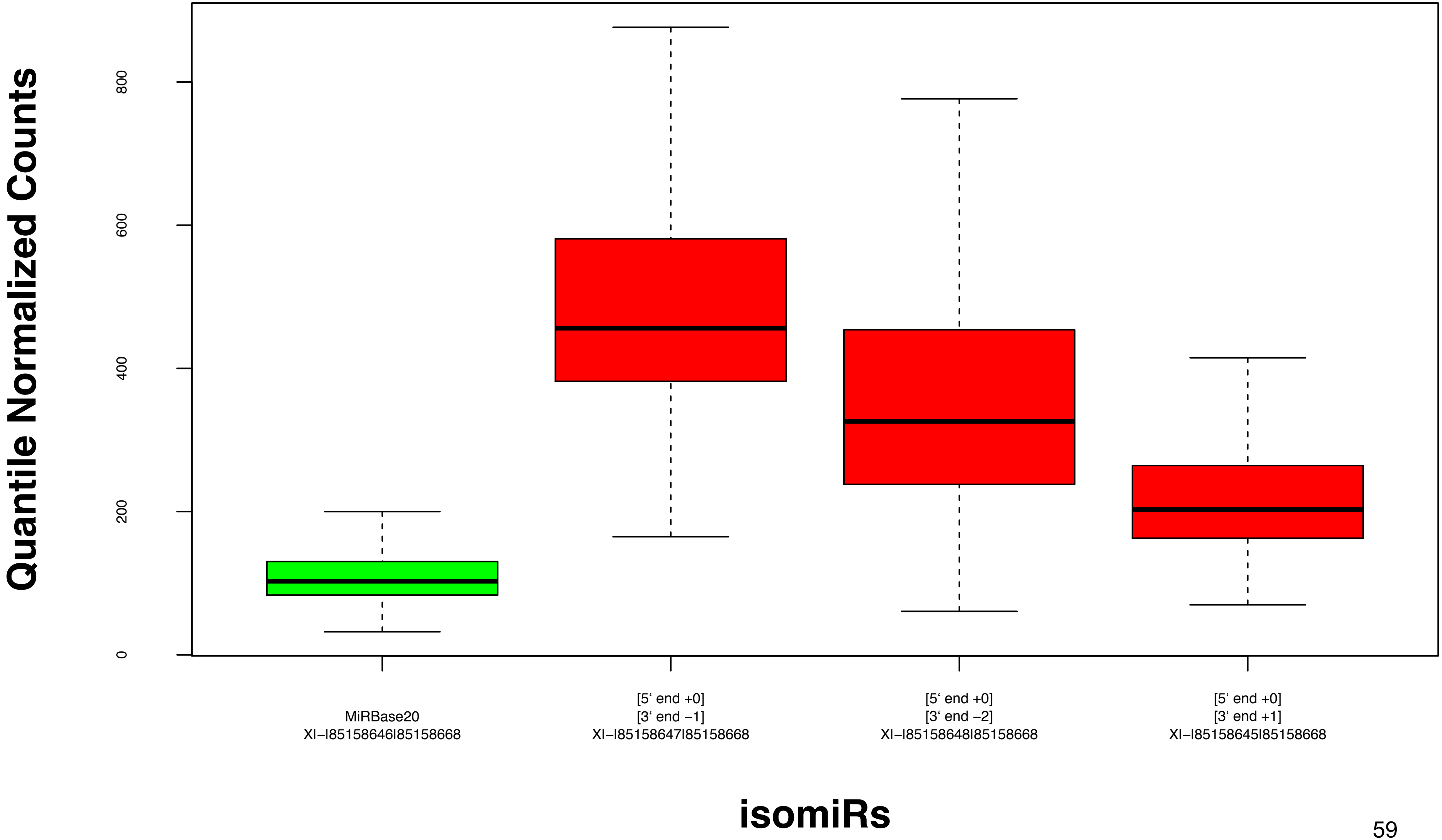

Supp. Figure 1
(pages 1 to 194)

MIMAT0002174=hsa–miR–484=MI0002468

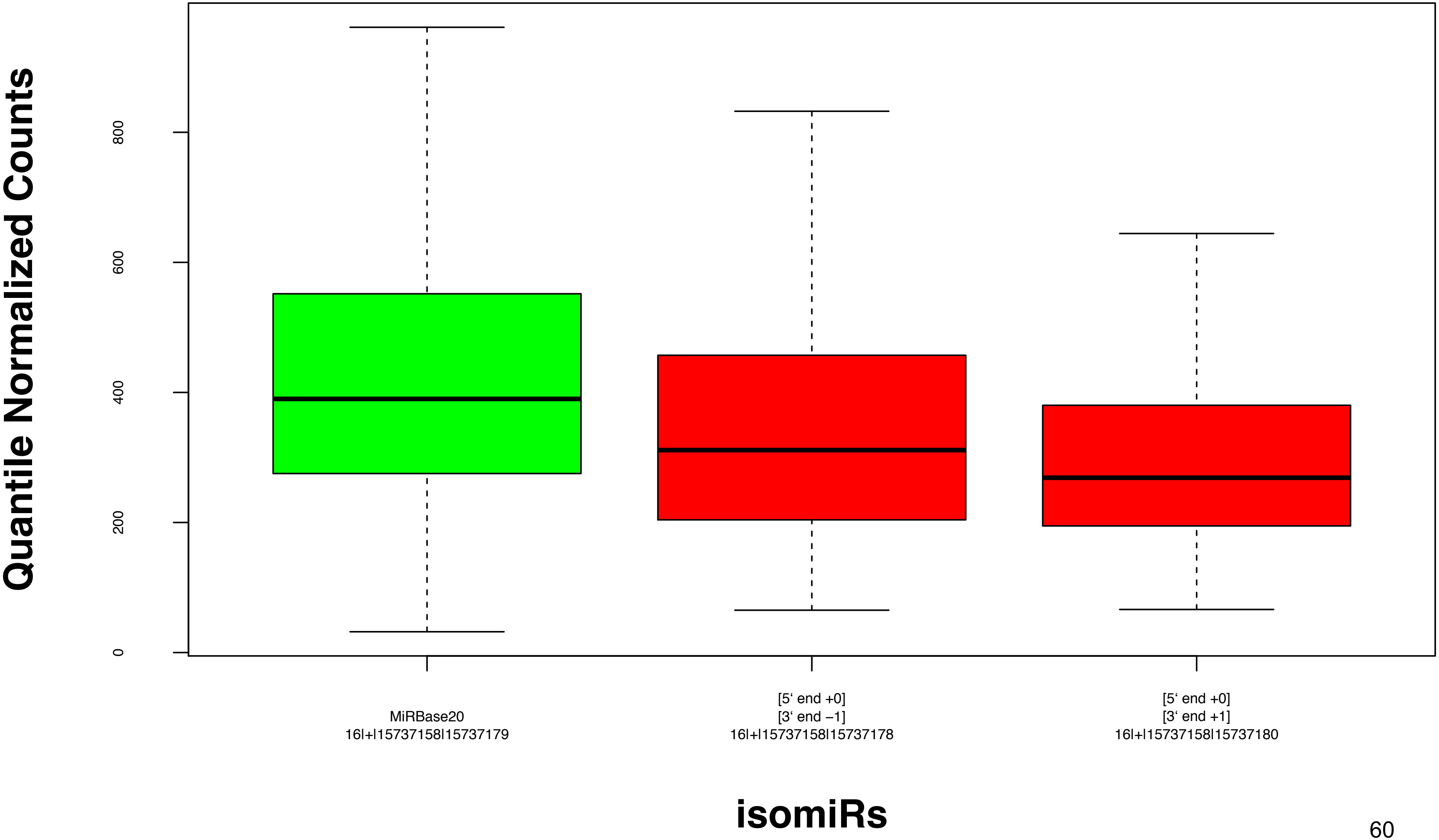

Supp. Figure 1
(pages 1 to 194)

MIMAT0002871=hsa–miR–500a–3p=MI0003184

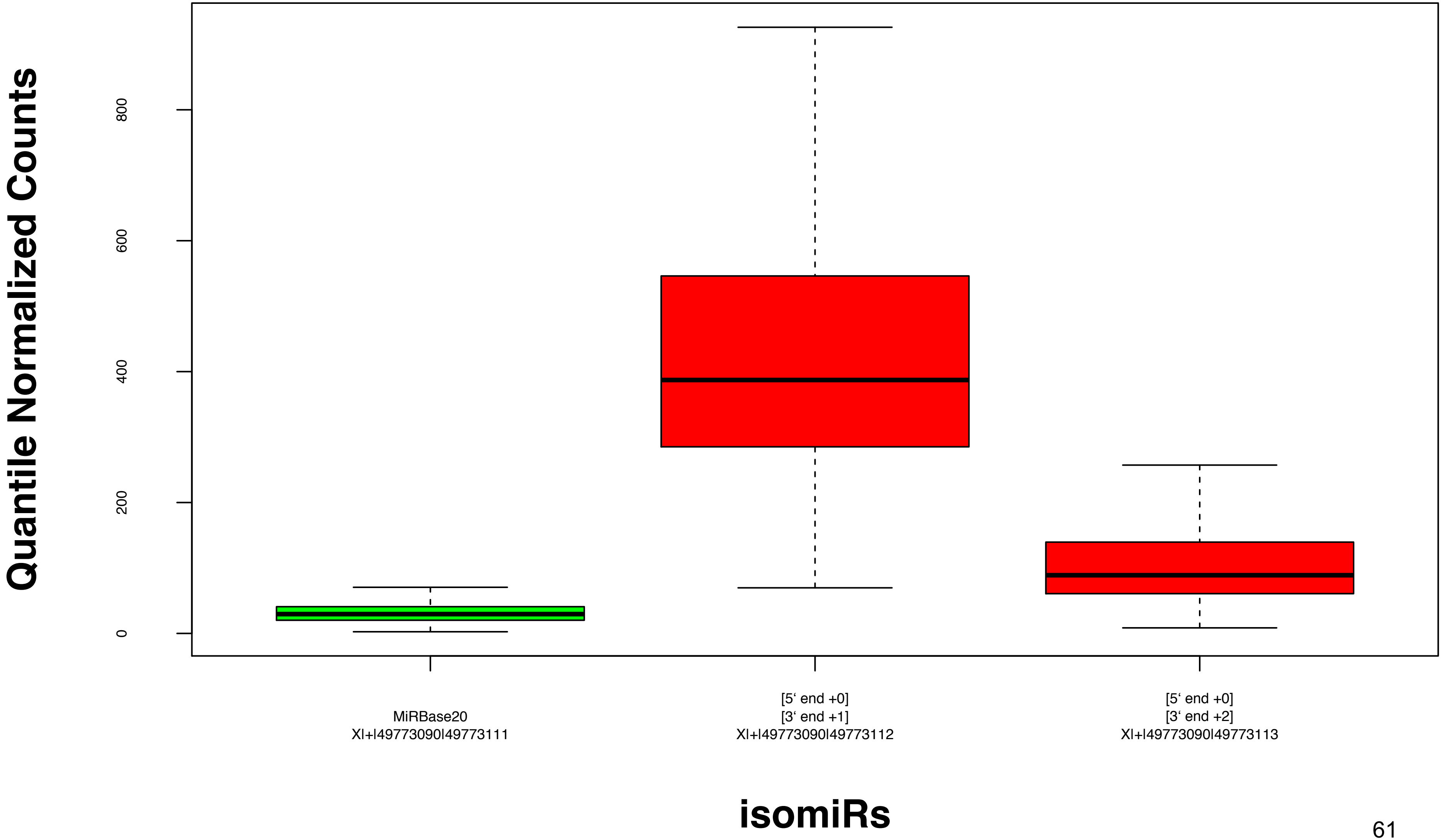

Supp. Figure 1
(pages 1 to 194)

MIMAT0000063=hsa–let–7b–5p=MI0000063

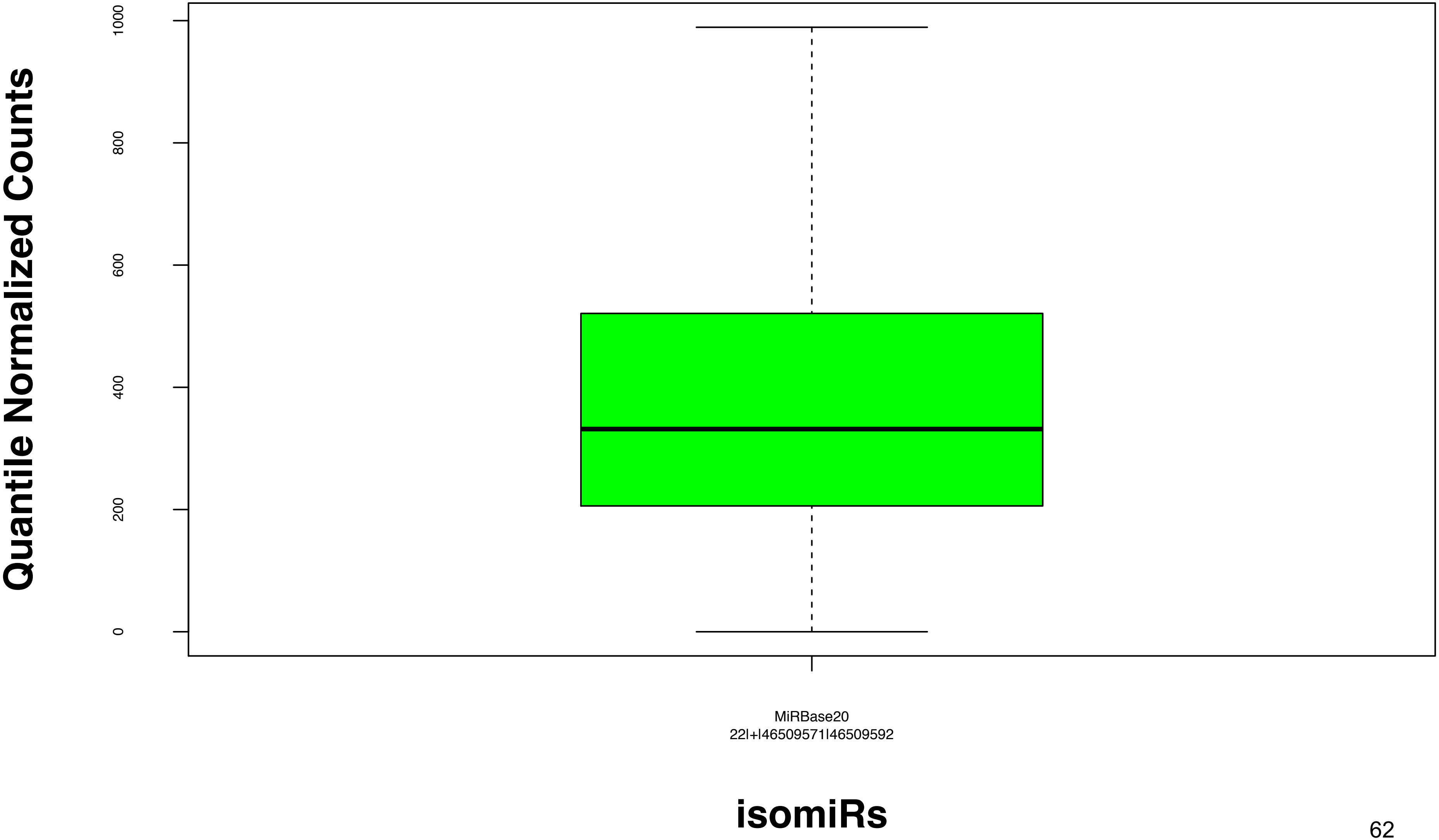

Supp. Figure 1
(pages 1 to 194)

MIMAT0031177=hsa−miR−7974=MI0025750

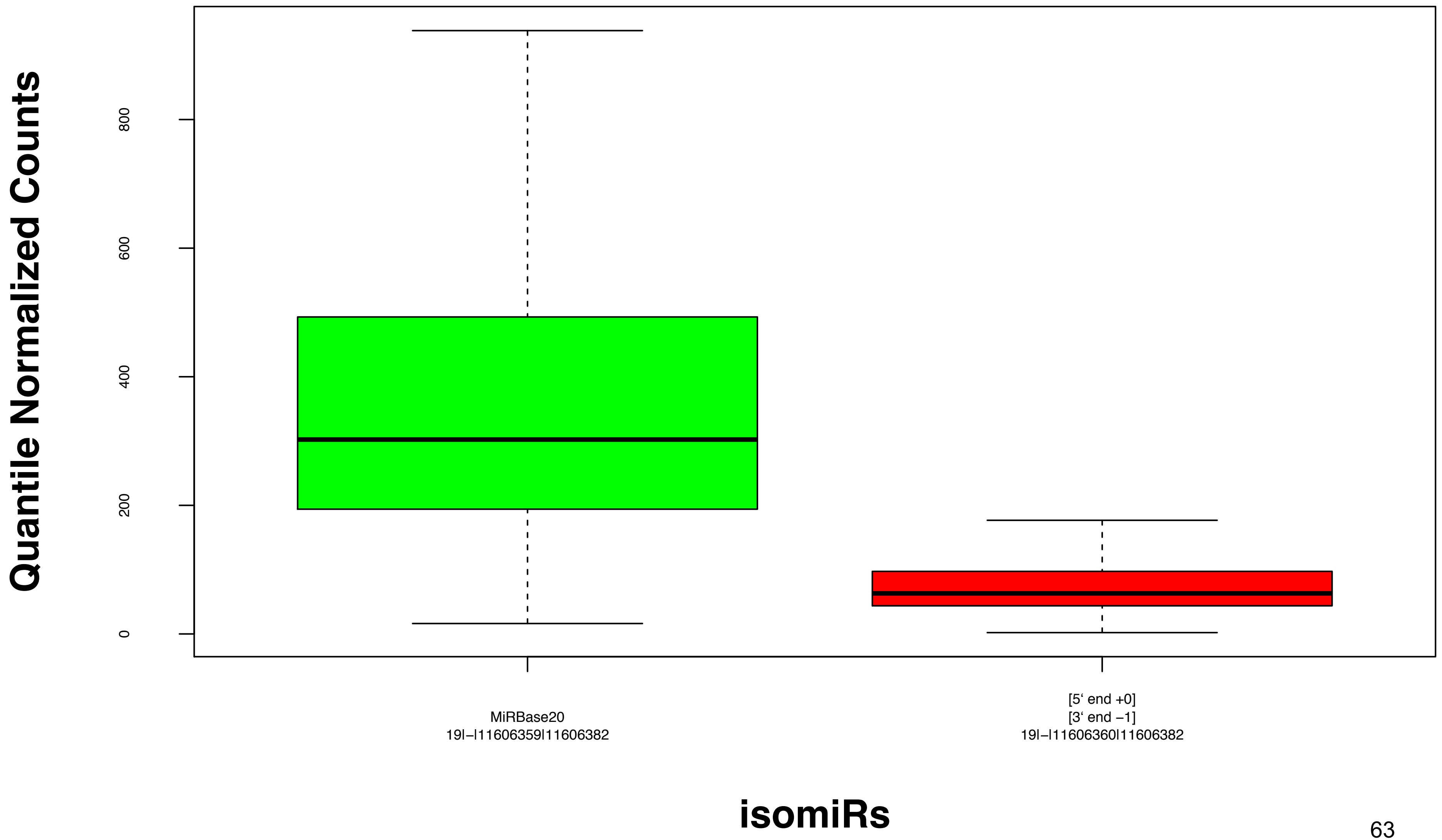

Supp. Figure 1
(pages 1 to 194)

MIMAT0000071=hsa−miR−17−3p=MI0000071

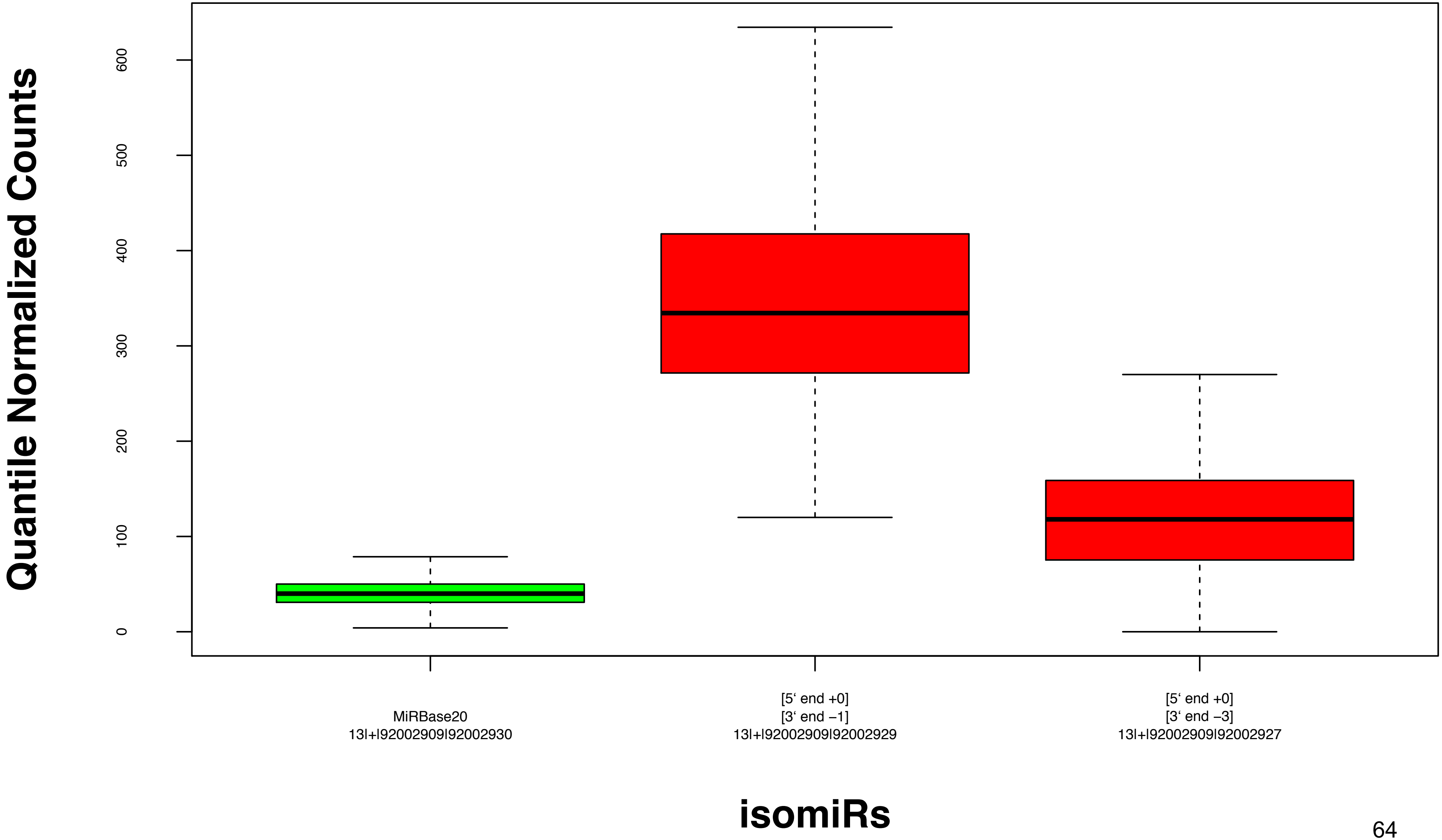

Supp. Figure 1
(pages 1 to 194)

MIMAT0000078=hsa–miR–23a–3p=MI0000079

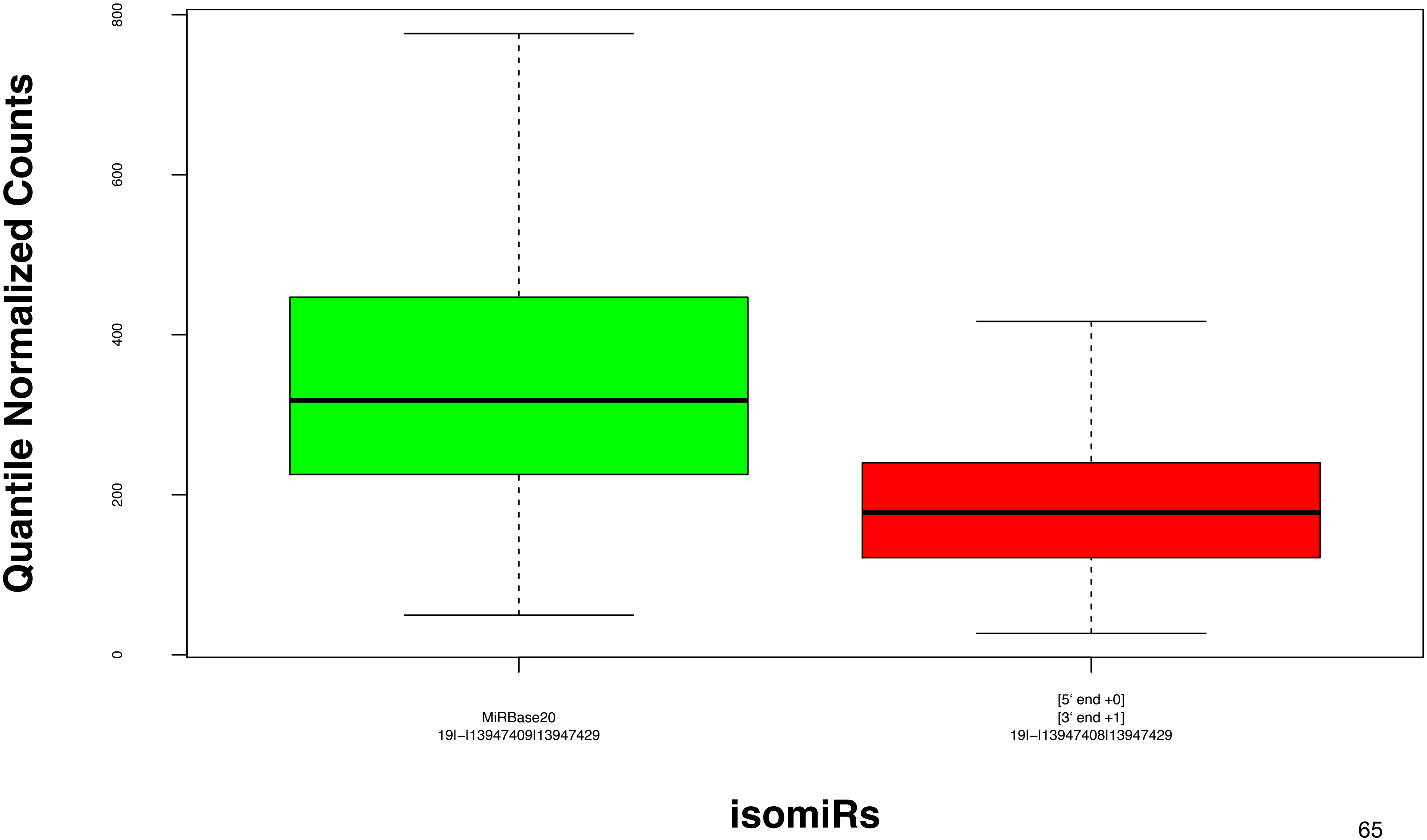

Supp. Figure 1
(pages 1 to 194)

MIMAT0022727=hsa–miR–1307–5p=MI0006444

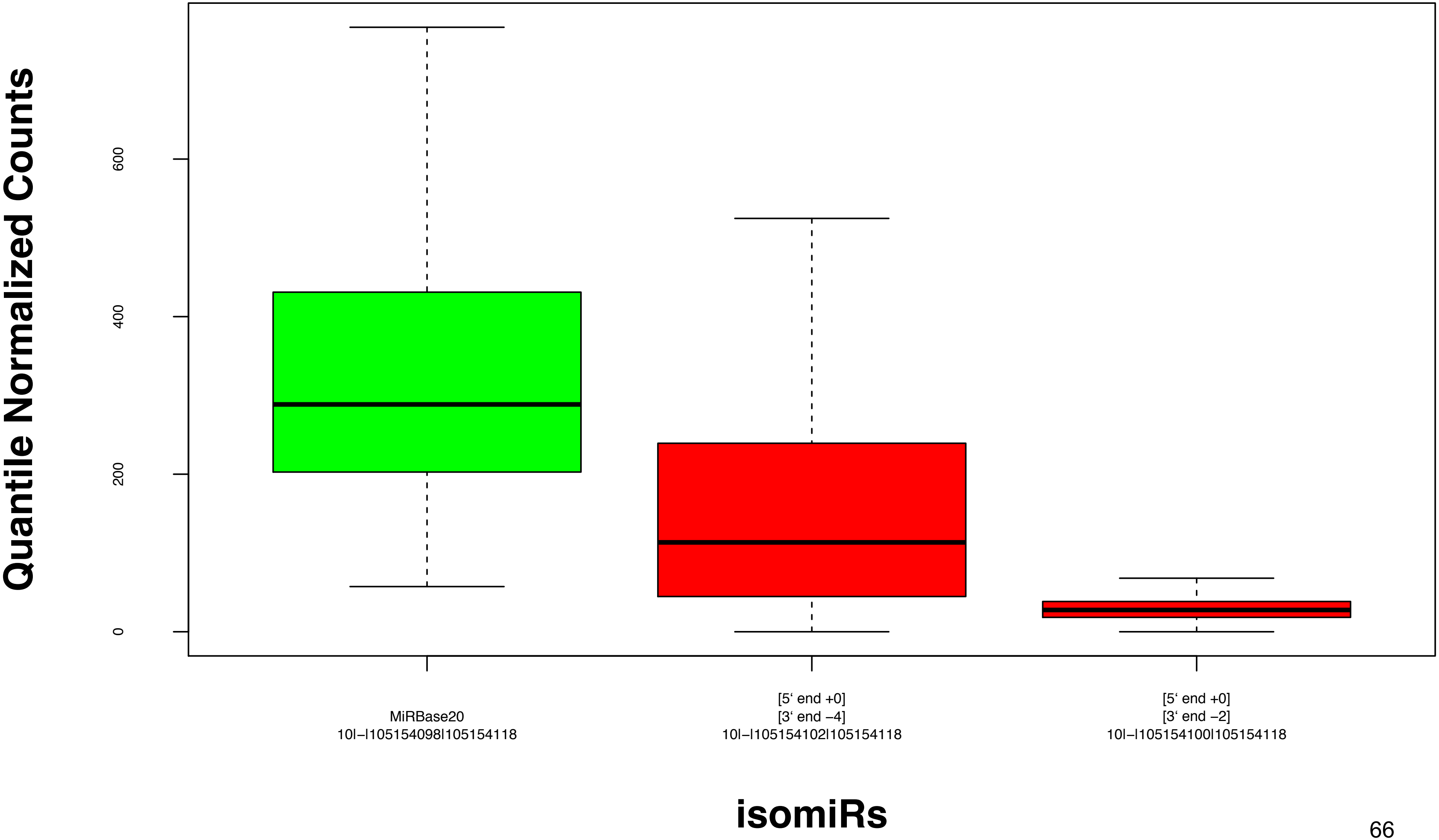

Supp. Figure 1
(pages 1 to 194)

MIMAT0026475=hsa−miR−210−5p=MI0000286

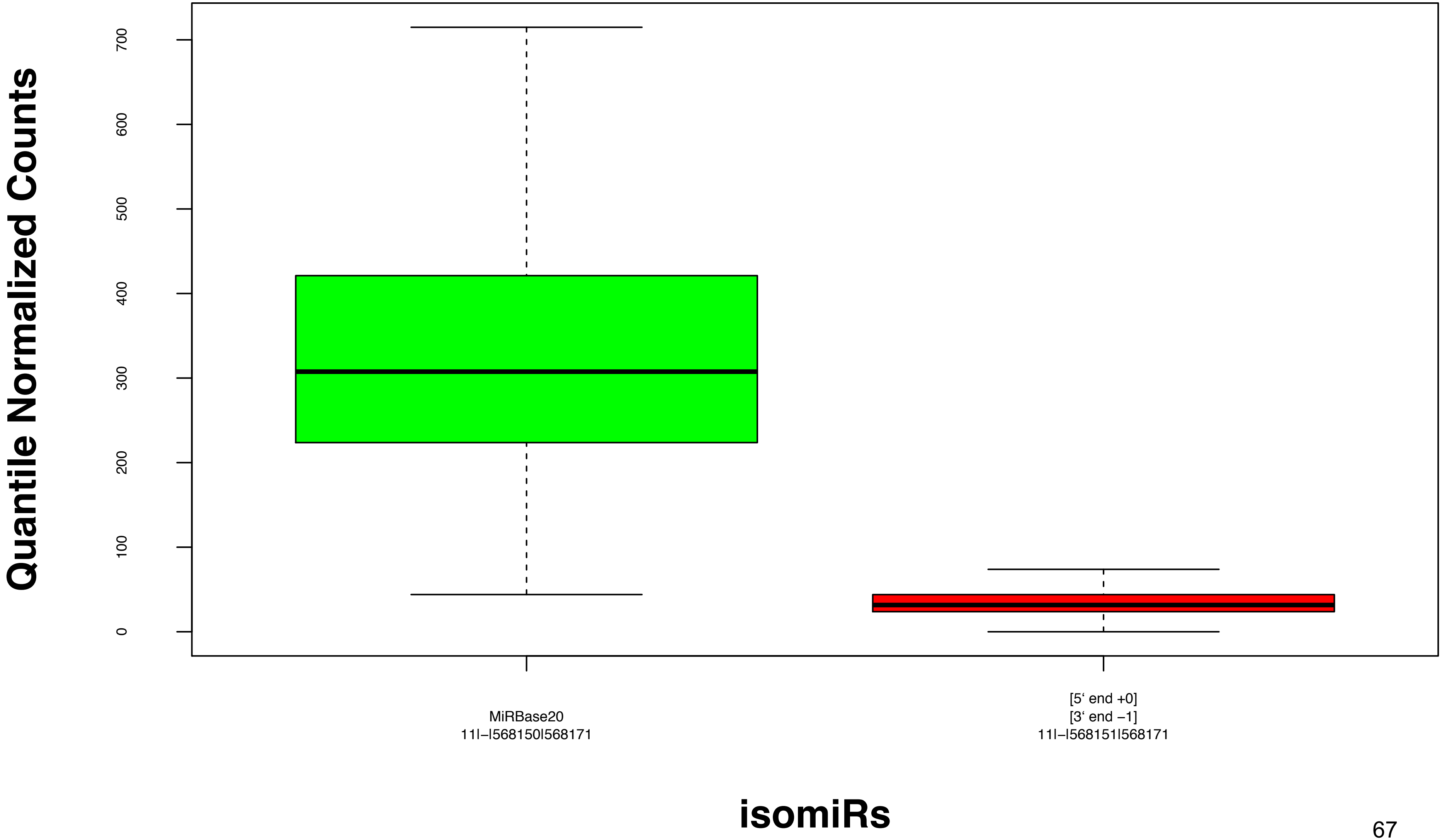

Supp. Figure 1
(pages 1 to 194)

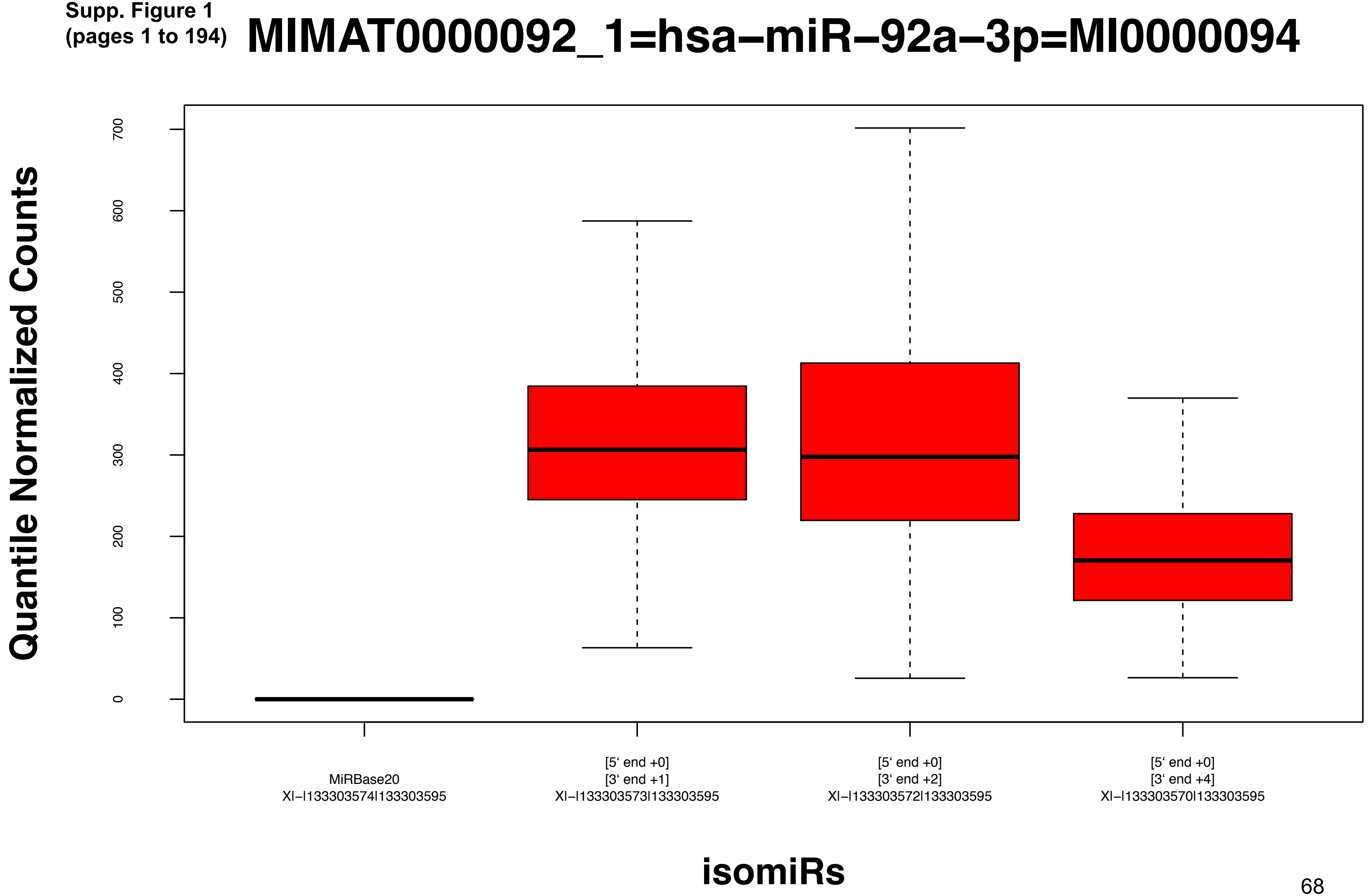

Supp. Figure 1
(pages 1 to 194)

MIMAT0002888=hsa−miR−532−5p=MI0003205

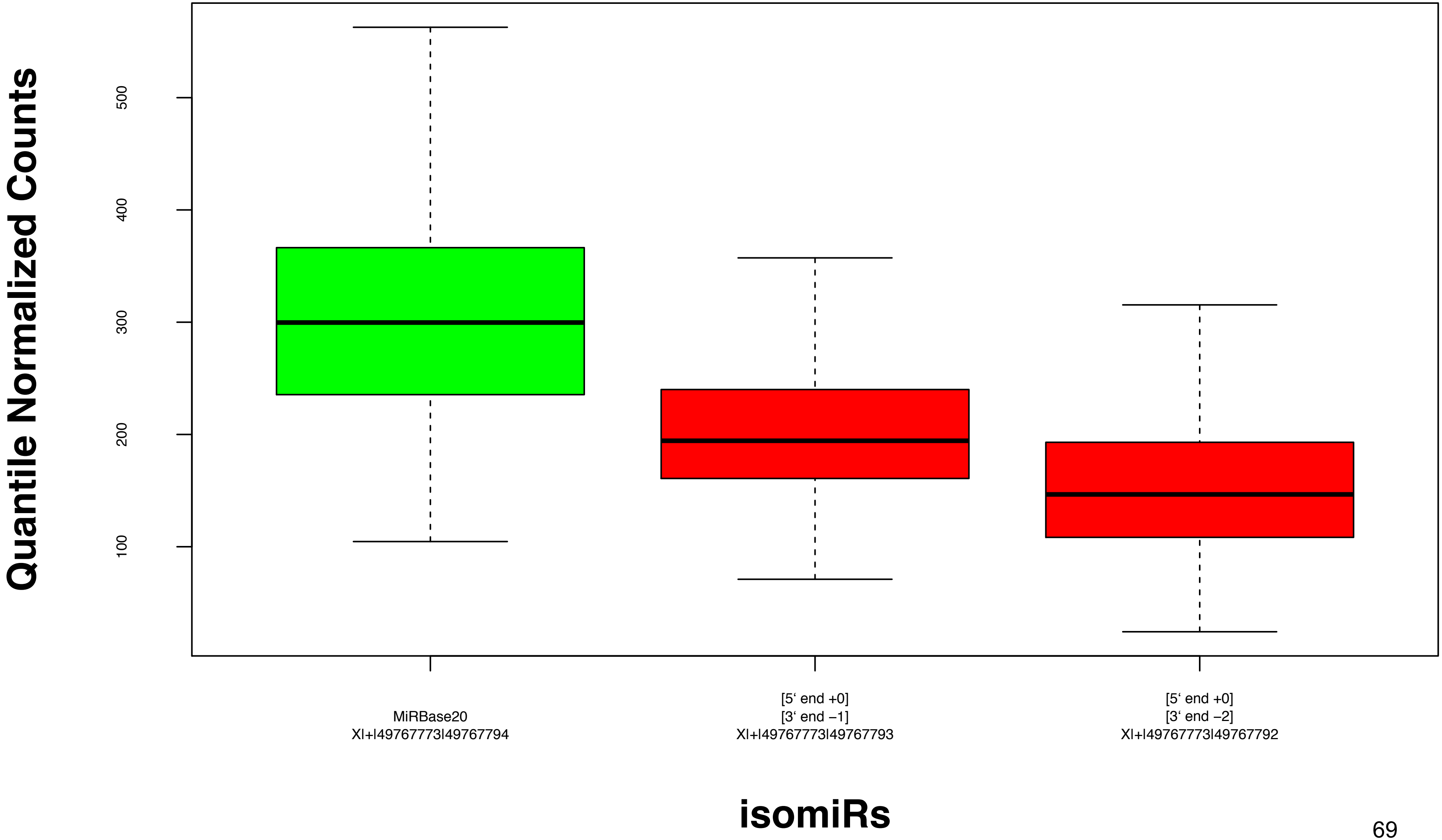

Supp. Figure 1
(pages 1 to 194)

MIMAT0000227=hsa−miR−197−3p=MI0000239

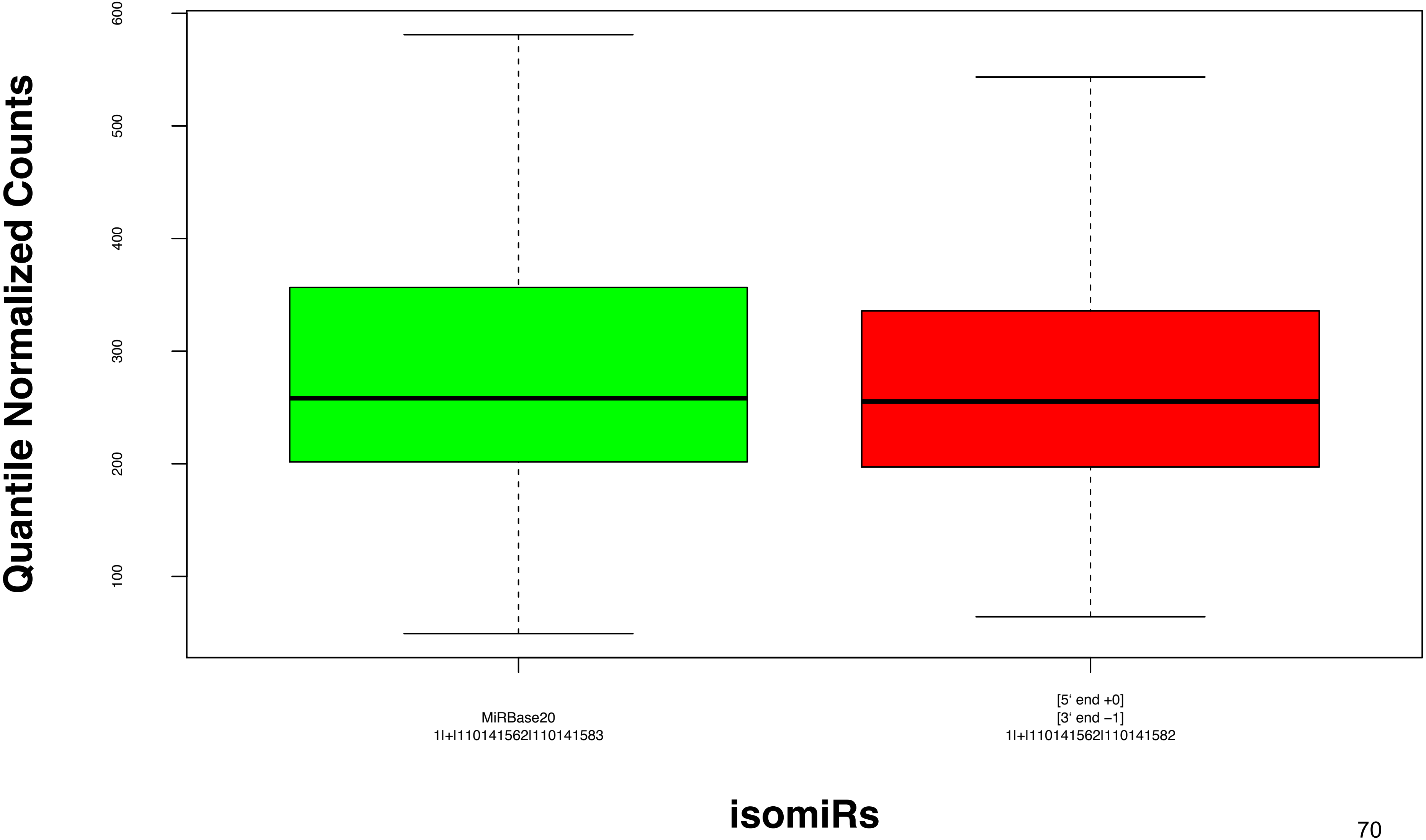

Supp. Figure 1
(pages 1 to 194)

MIMAT0000068=hsa−miR−15a−5p=MI0000069

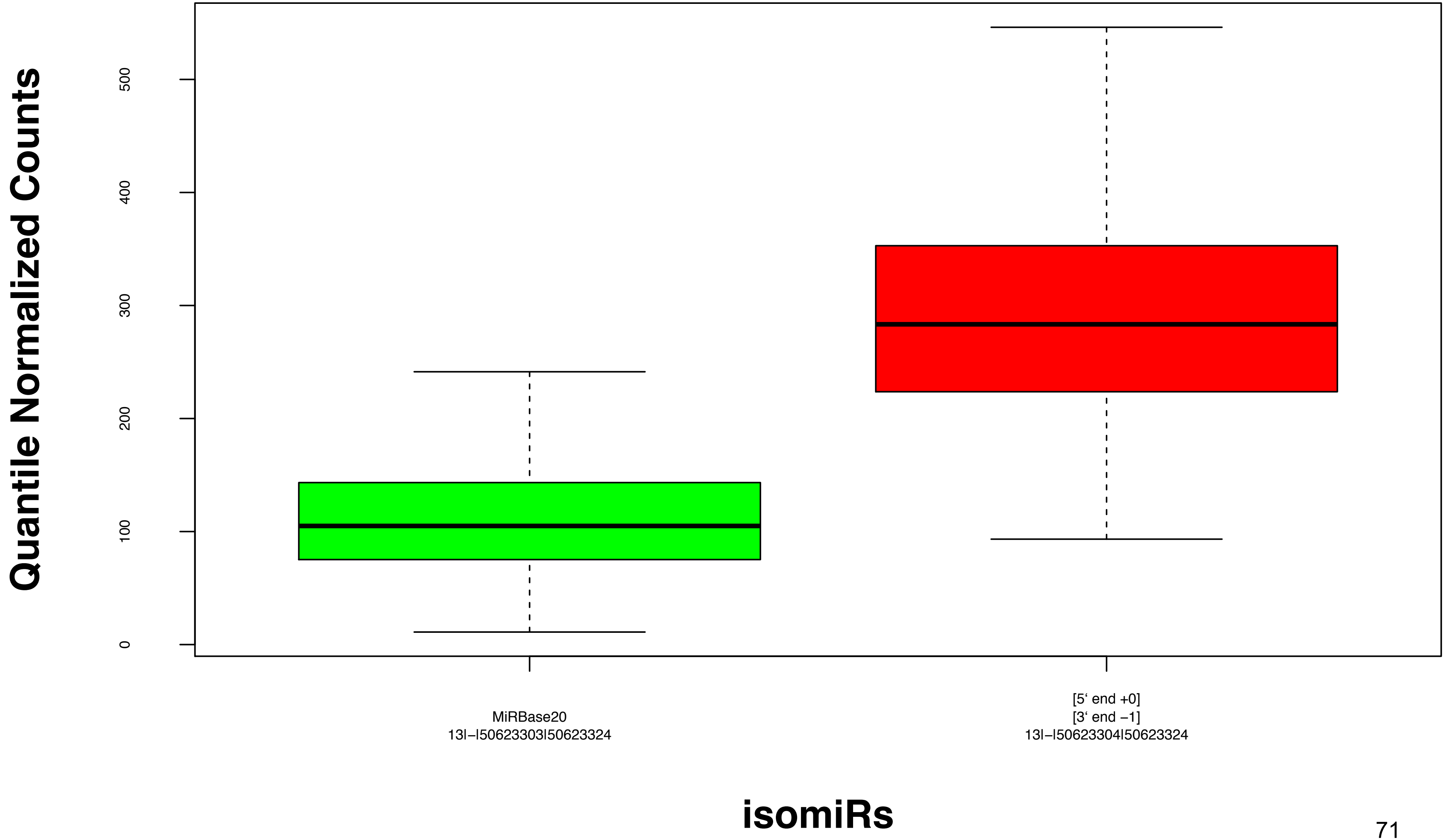

Supp. Figure 1
(pages 1 to 194)

MIMAT0000688=hsa–miR–301a–3p=MI0000745

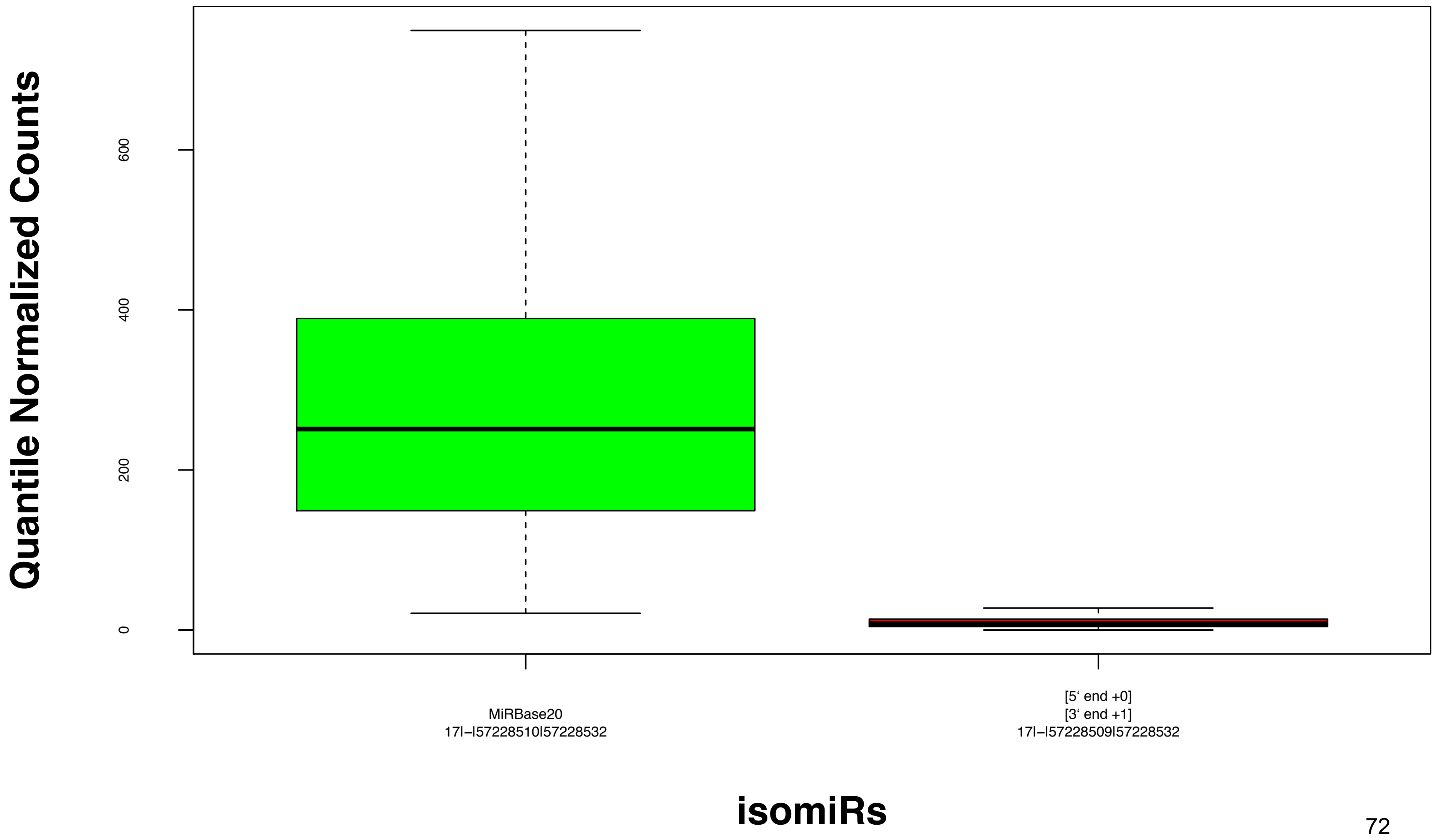

Supp. Figure 1
(pages 1 to 194)

MIMAT0004819=hsa−miR−671−3p=MI0003760

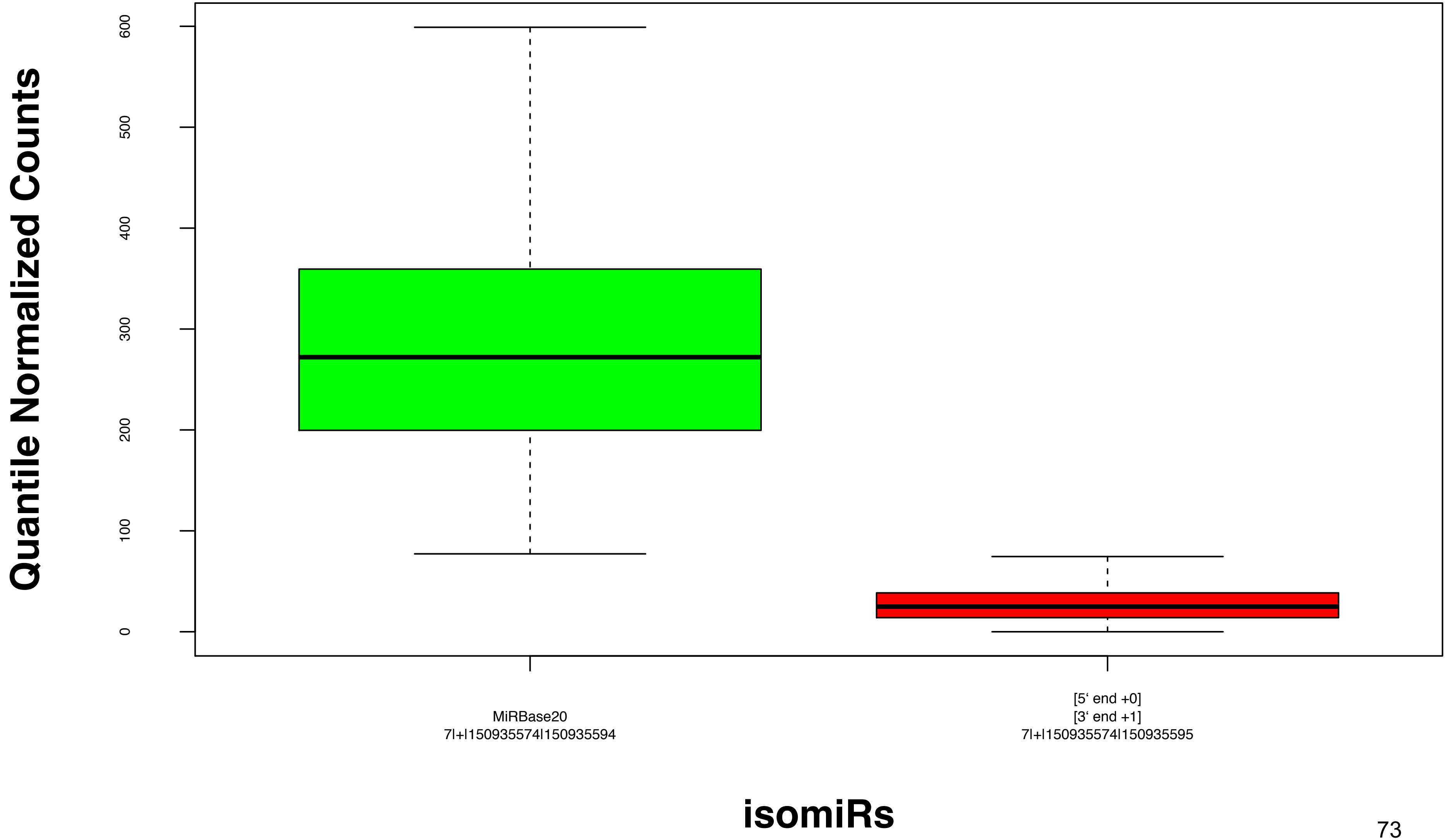

Supp. Figure 1
(pages 1 to 194)

MIMAT0000703=hsa−miR−361−5p=MI0000760

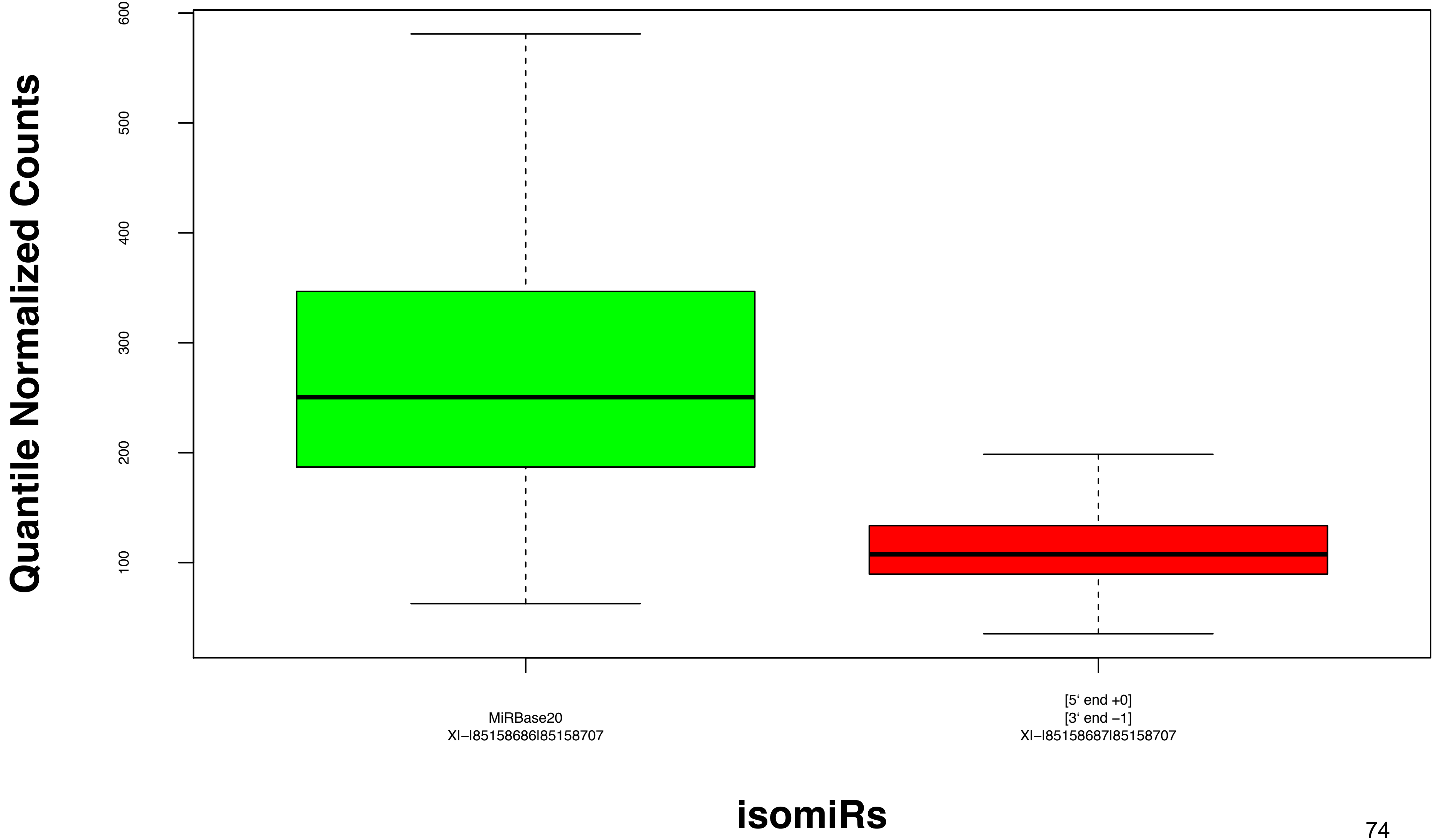

Supp. Figure 1
(pages 1 to 194)

MIMAT0004799=hsa−miR−589−5p=MI0003599

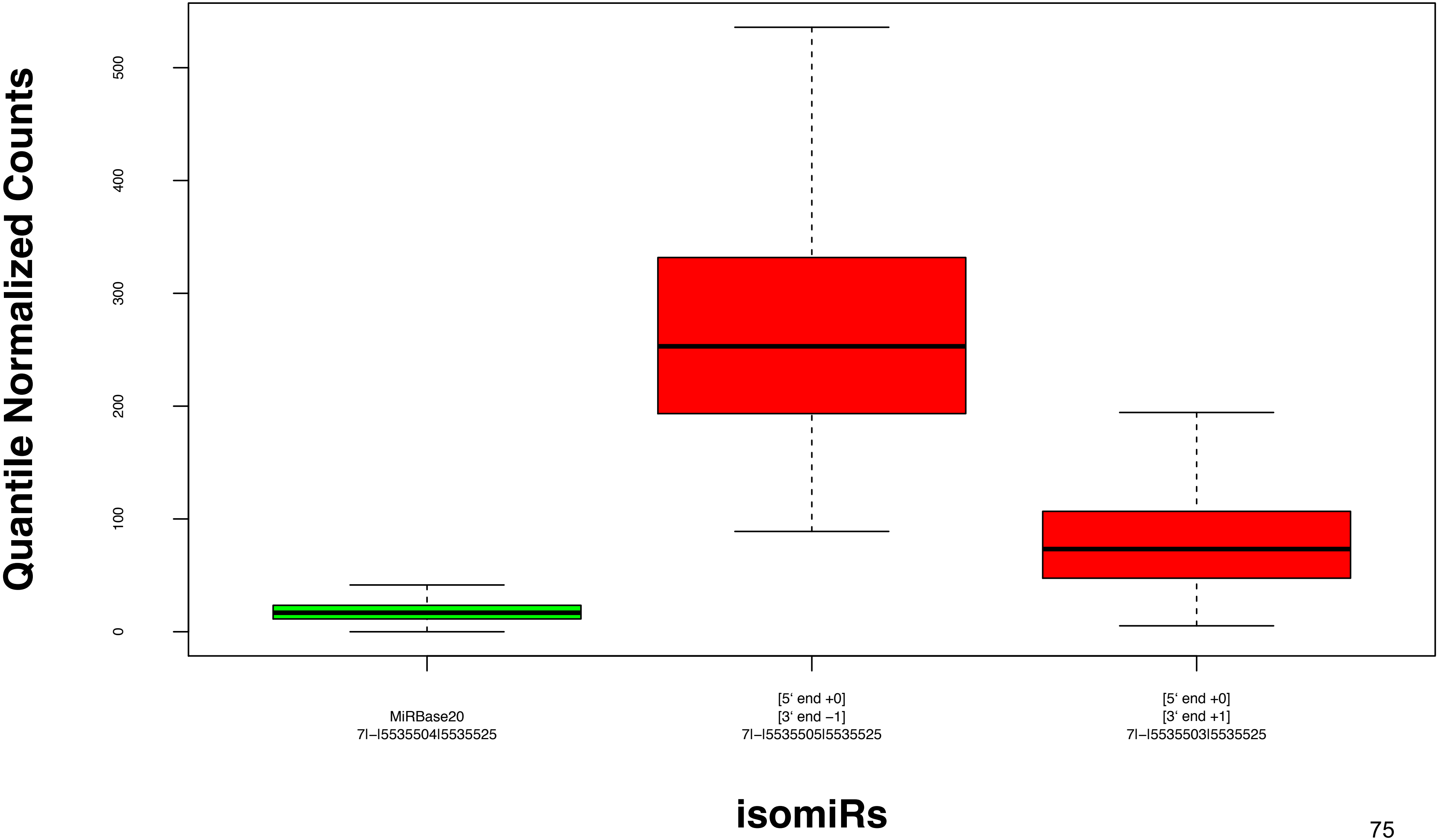

Supp. Figure 1
(pages 1 to 194)

MIMAT0001413=hsa−miR−20b−5p=MI0001519

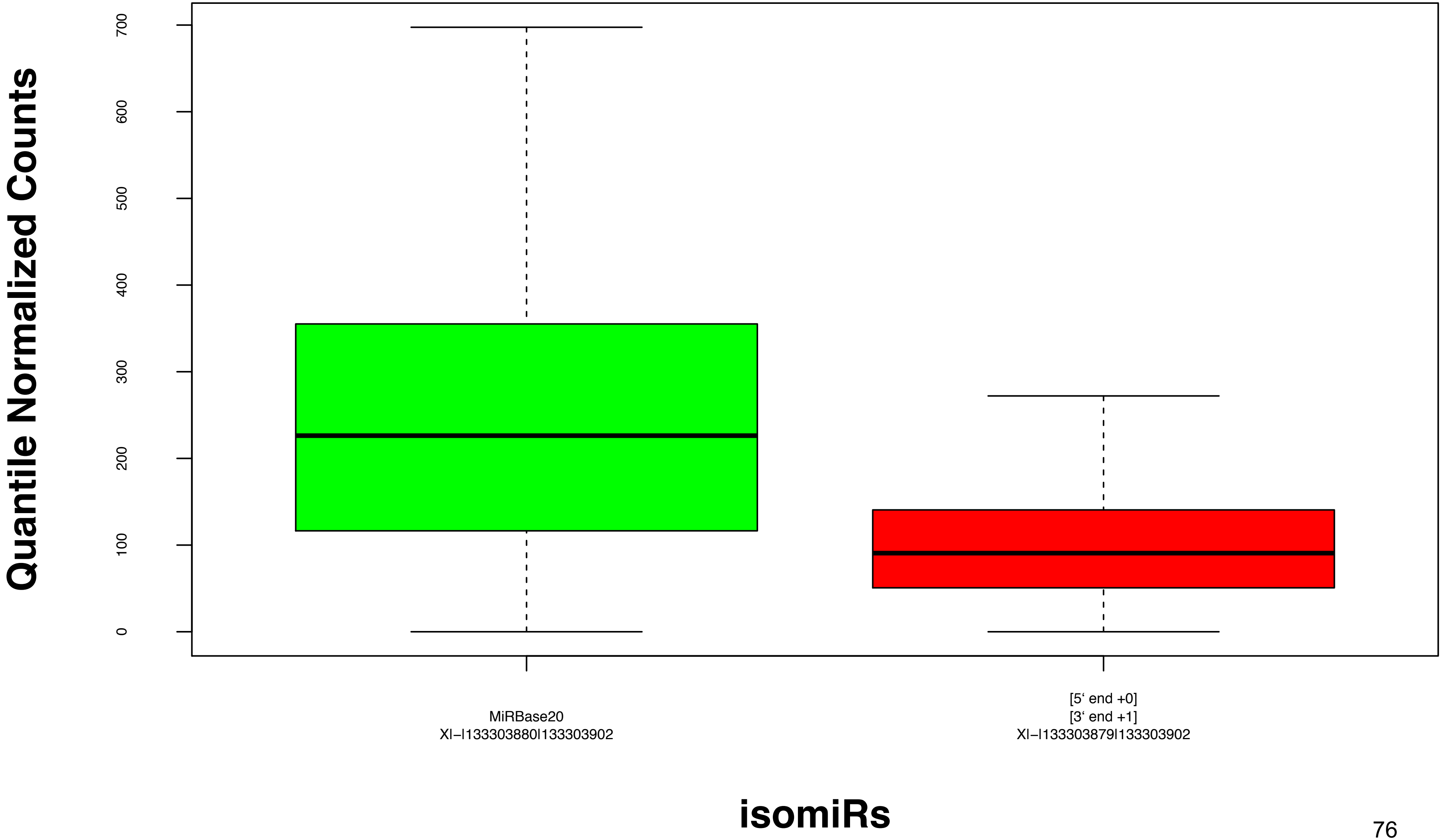

Supp. Figure 1
(pages 1 to 194)

MIMAT0000772=hsa−miR−345−5p=MI0000825

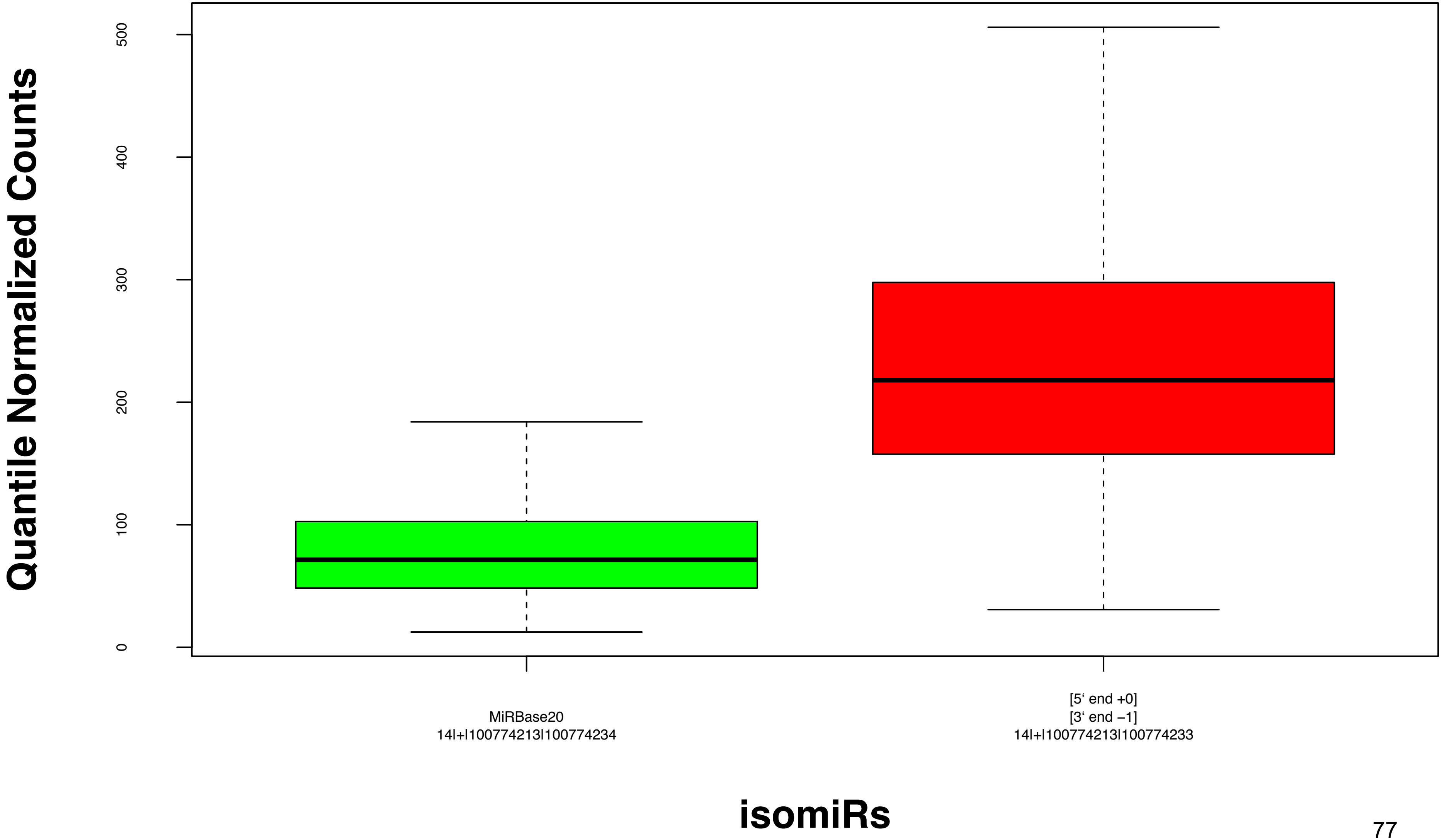

Supp. Figure 1
(pages 1 to 194)

MIMAT0003885=hsa−miR−454−3p=MI0003820

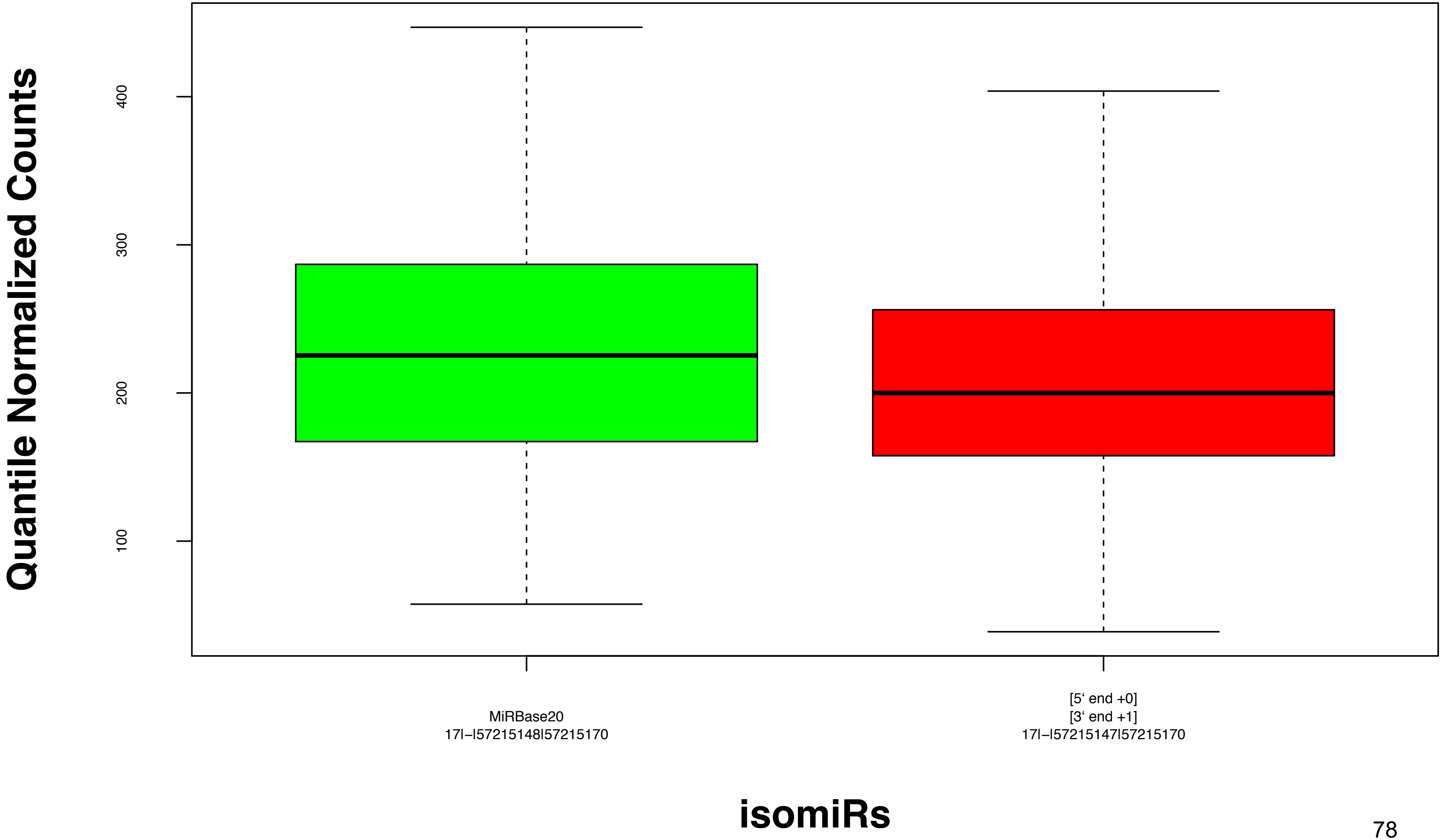

Supp. Figure 1
(pages 1 to 194)

MIMAT0000426=hsa−miR−132−3p=MI0000449

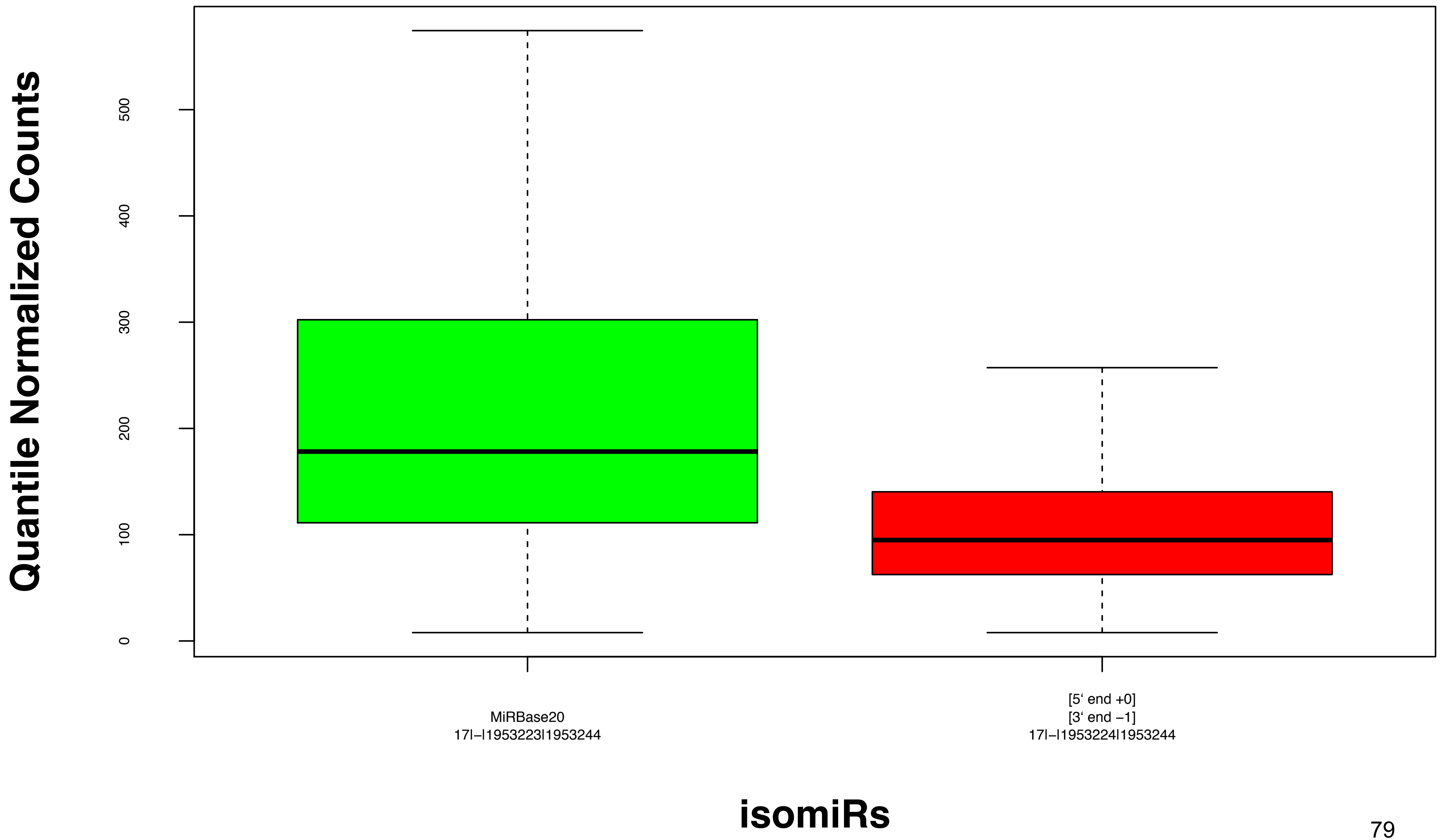

Supp. Figure 1
(pages 1 to 194)

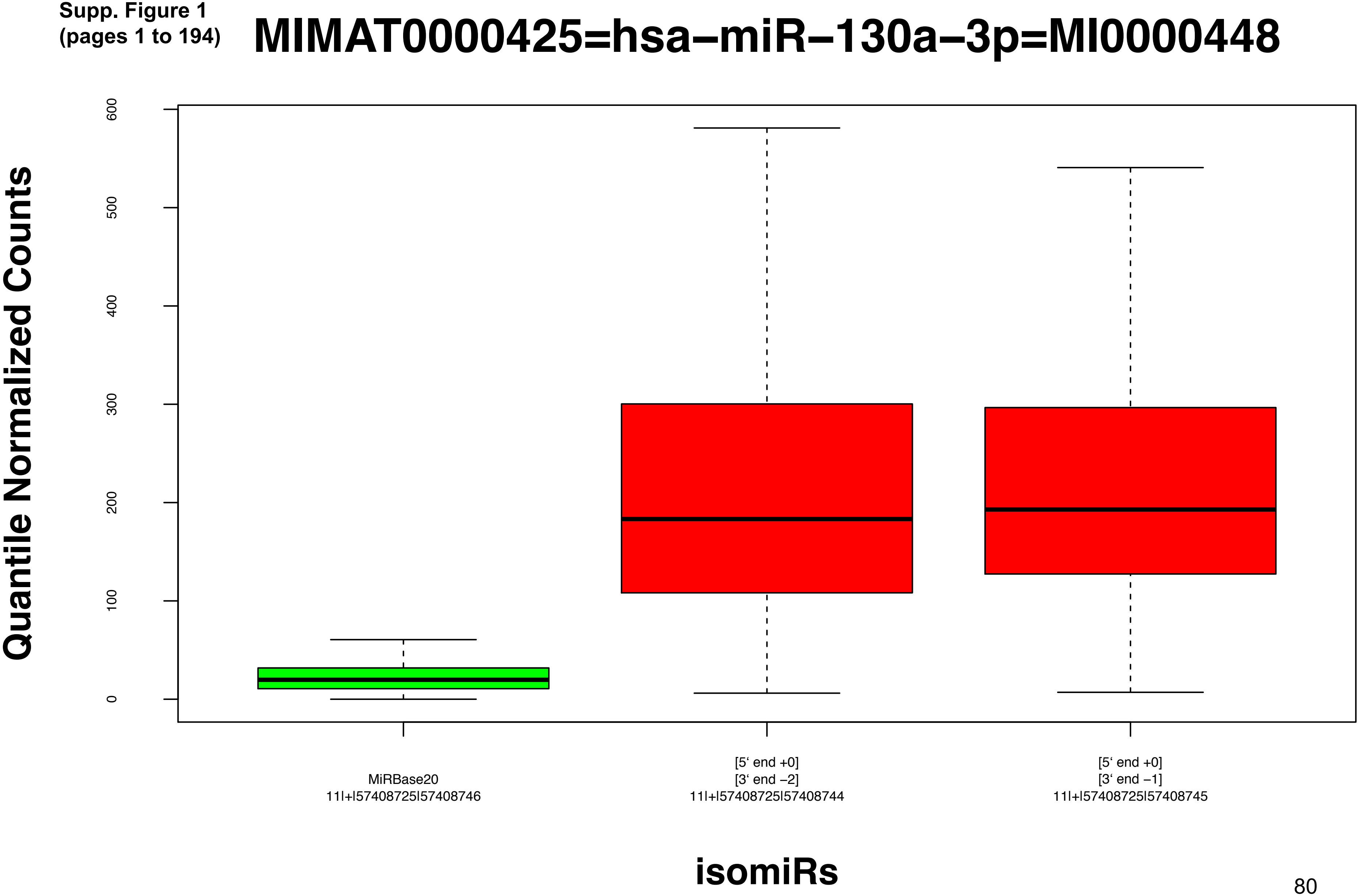

Supp. Figure 1
(pages 1 to 194)

MIMAT0000451=hsa−miR−150−5p=MI0000479

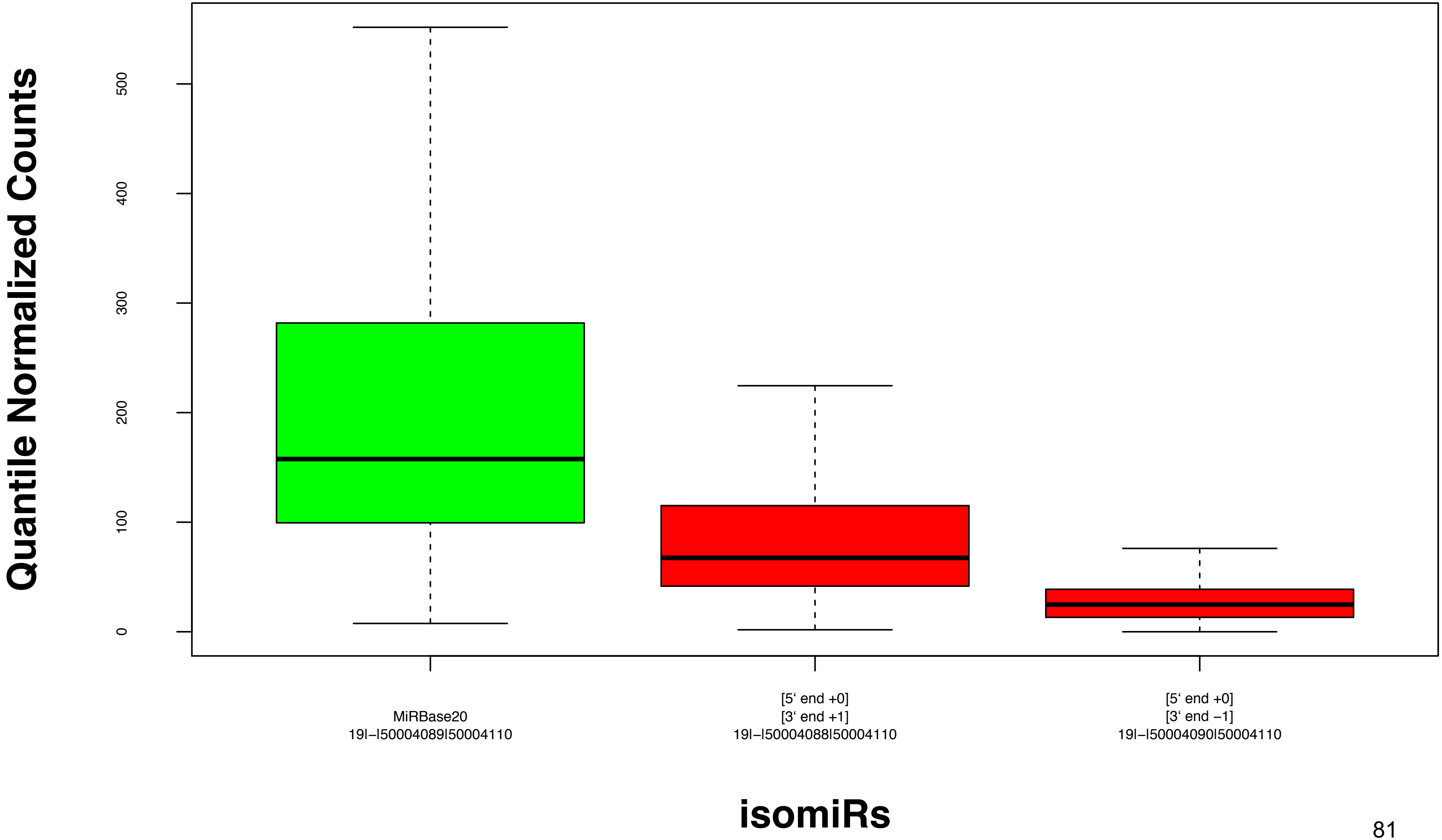

Supp. Figure 1
(pages 1 to 194)

MIMAT0004502=hsa−miR−28−3p=MI0000086

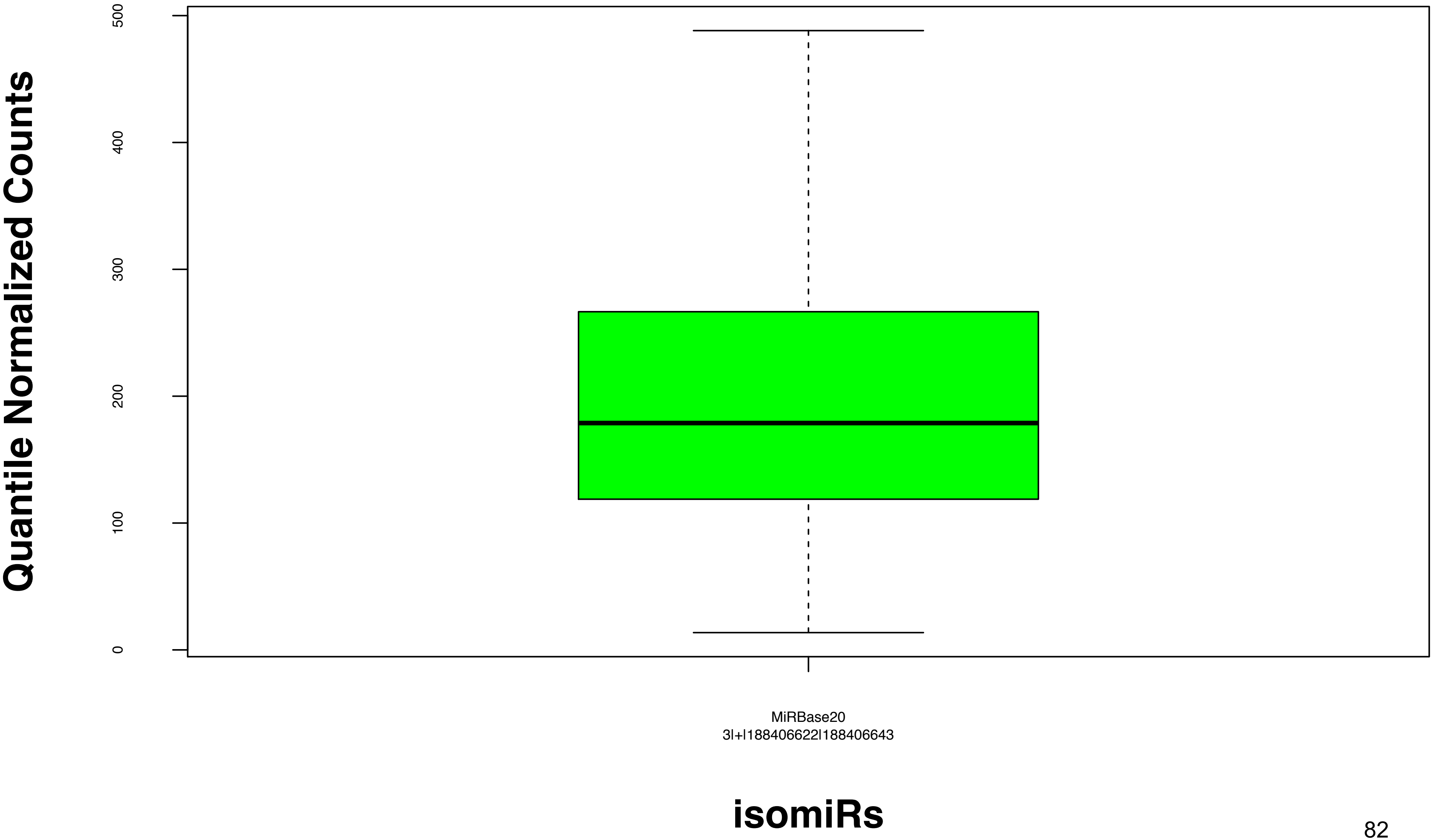

Supp. Figure 1
(pages 1 to 194)

MIMAT0000064=hsa-let-7c-5p=MI0000064

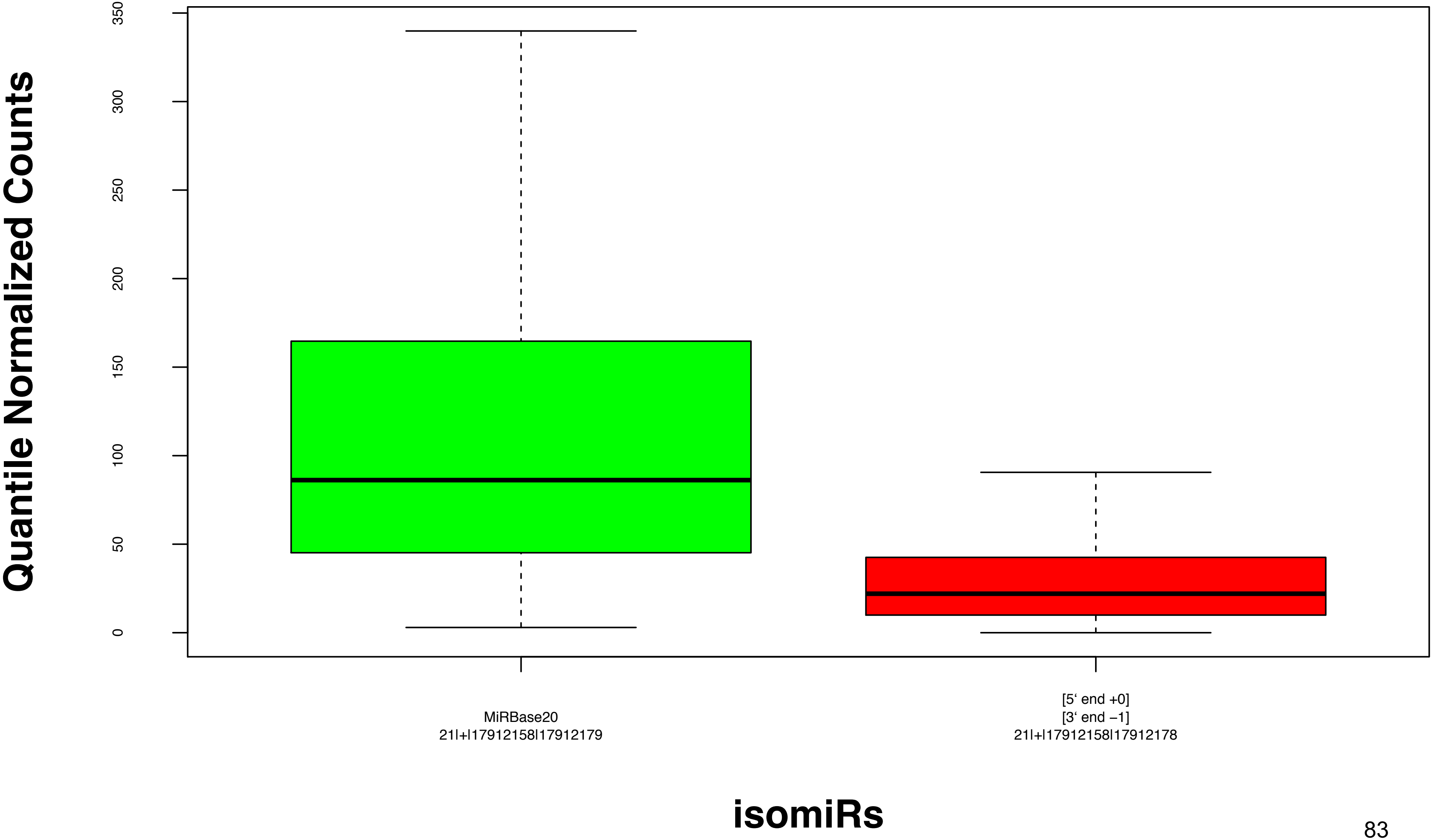

Supp. Figure 1
(pages 1 to 194)

MIMAT0005951=hsa−miR−1307−3p=MI0006444

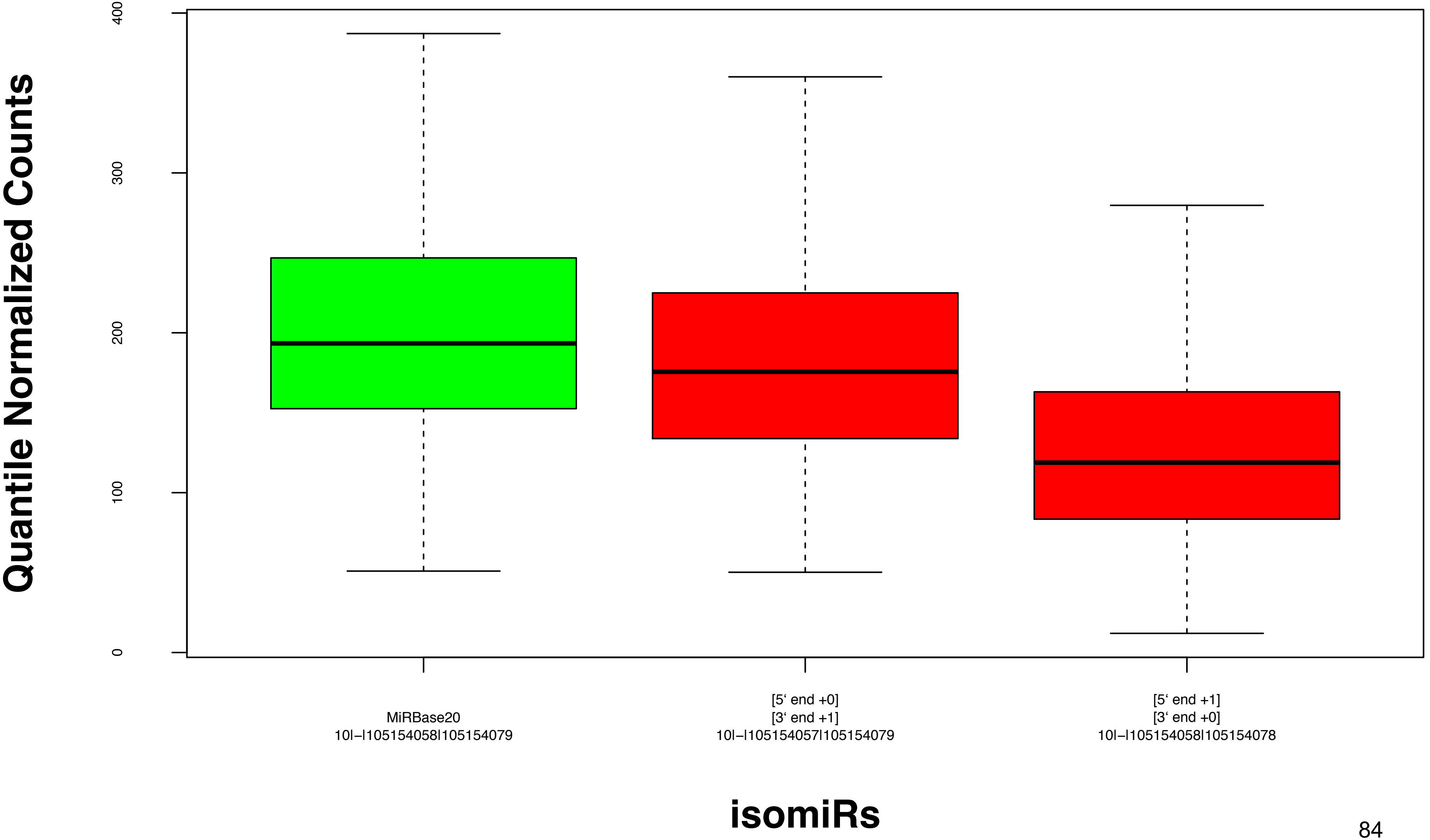

Supp. Figure 1
(pages 1 to 194)

MIMAT0004911=hsa−miR−874−3p=MI0005532

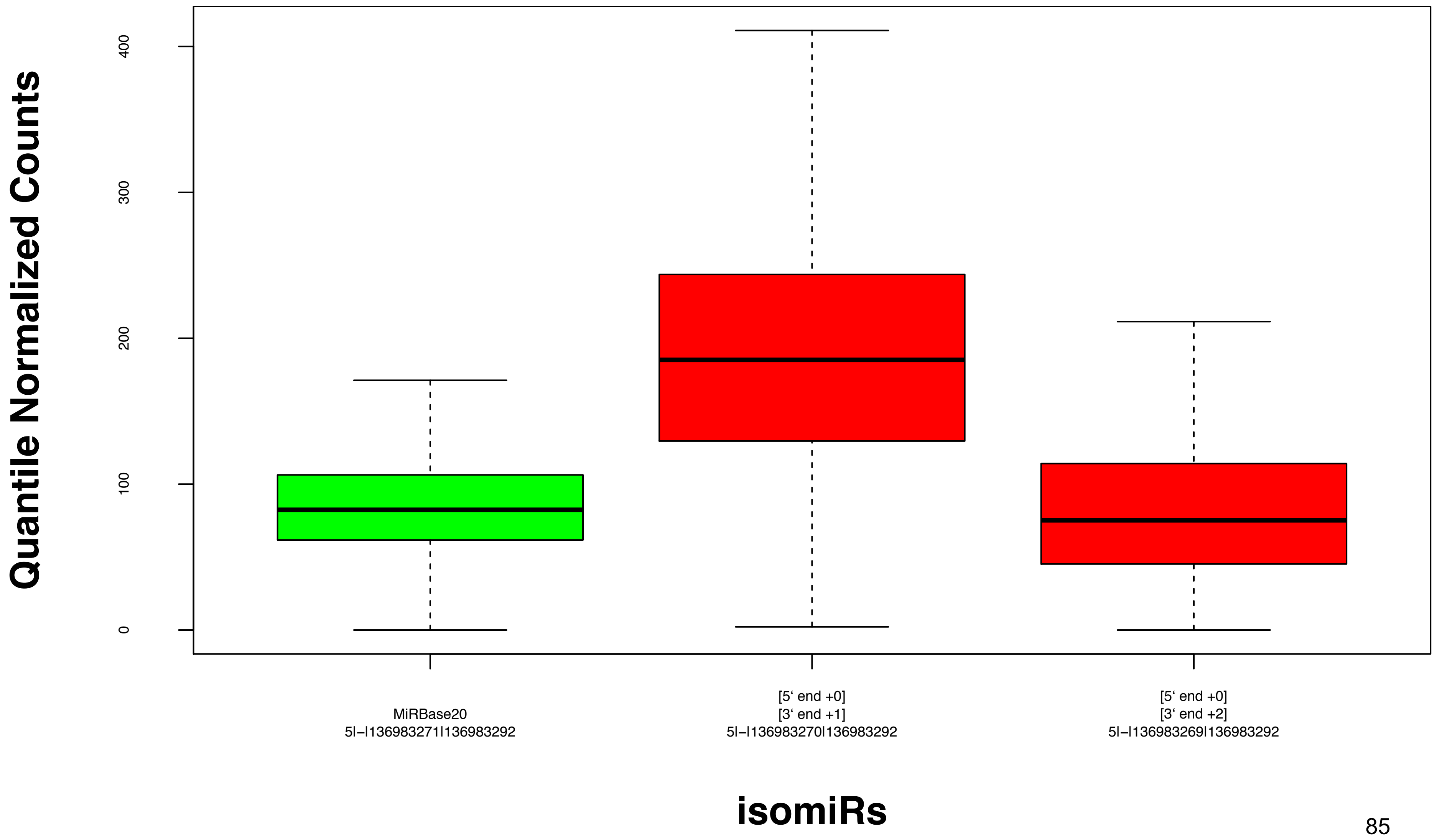

Supp. Figure 1
(pages 1 to 194)

MIMAT0004484=hsa–let–7d–3p=MI0000065

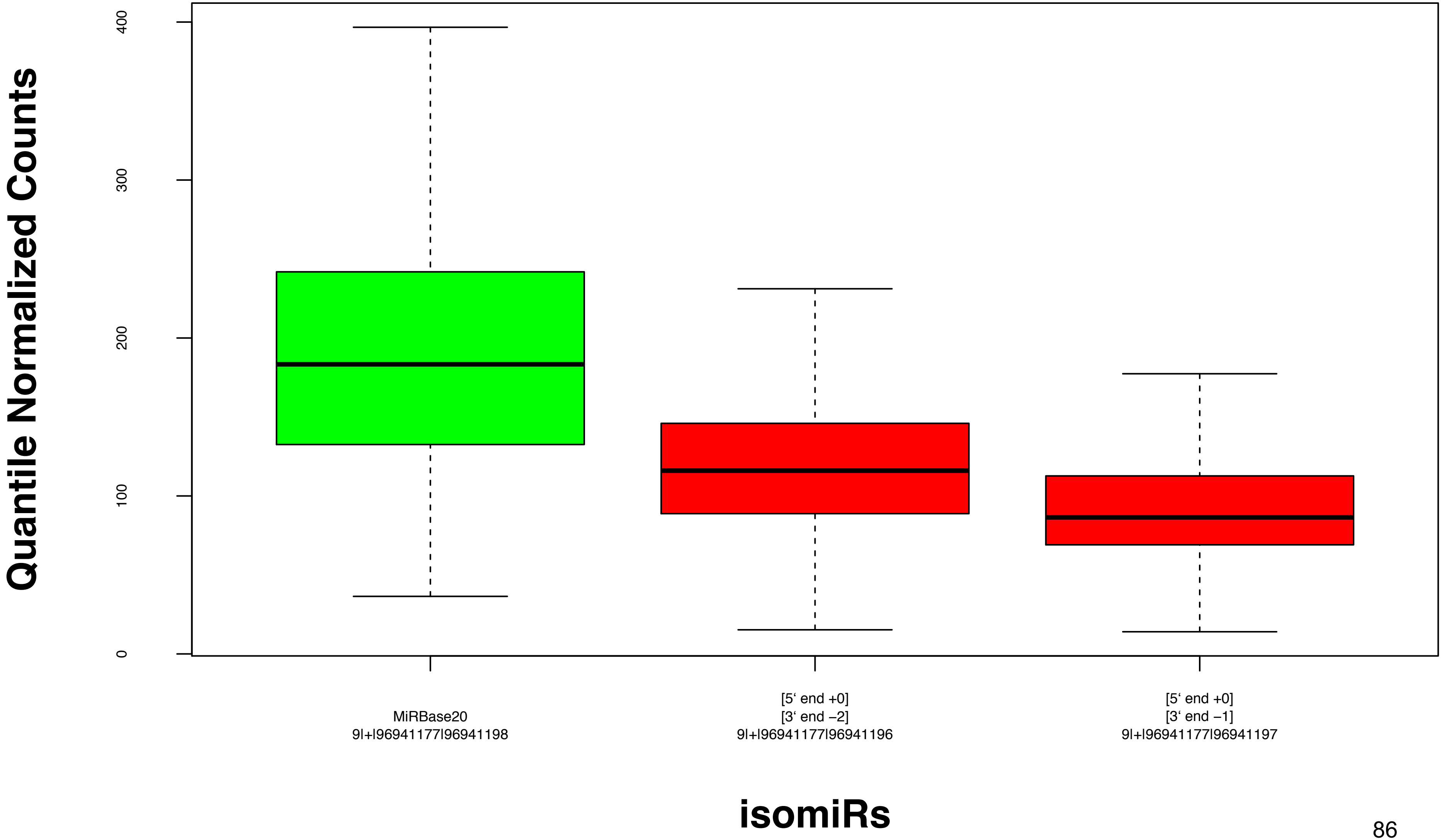

Supp. Figure 1
(pages 1 to 194)

MIMAT0000432=hsa−miR−141−3p=MI0000457

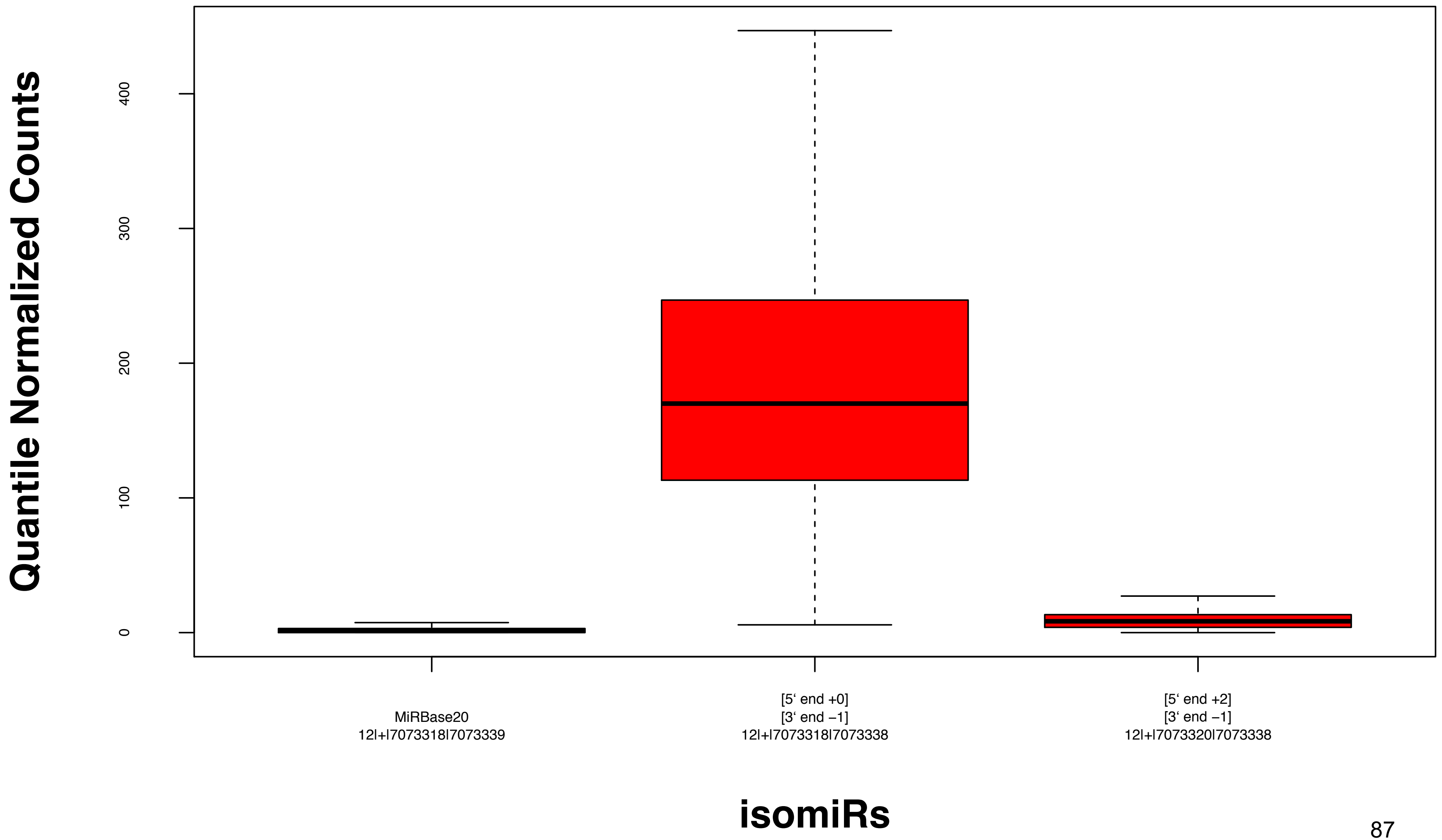

Supp. Figure 1
(pages 1 to 194)

MIMAT0000257_1=hsa–miR–181b–5p=MI0000683

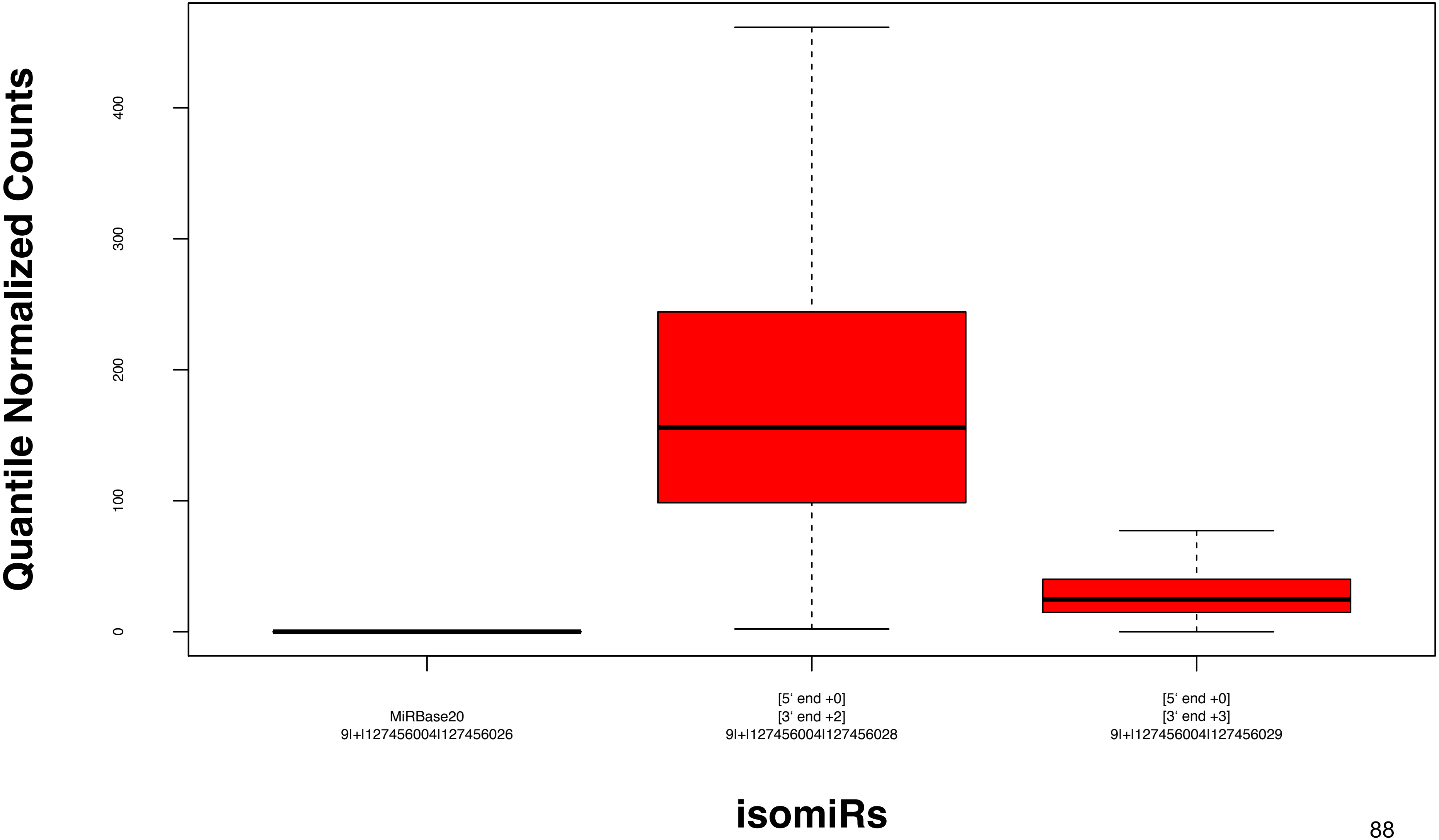

Supp. Figure 1
(pages 1 to 194)

MIMAT0004680=hsa–miR–130b–5p=MI0000748

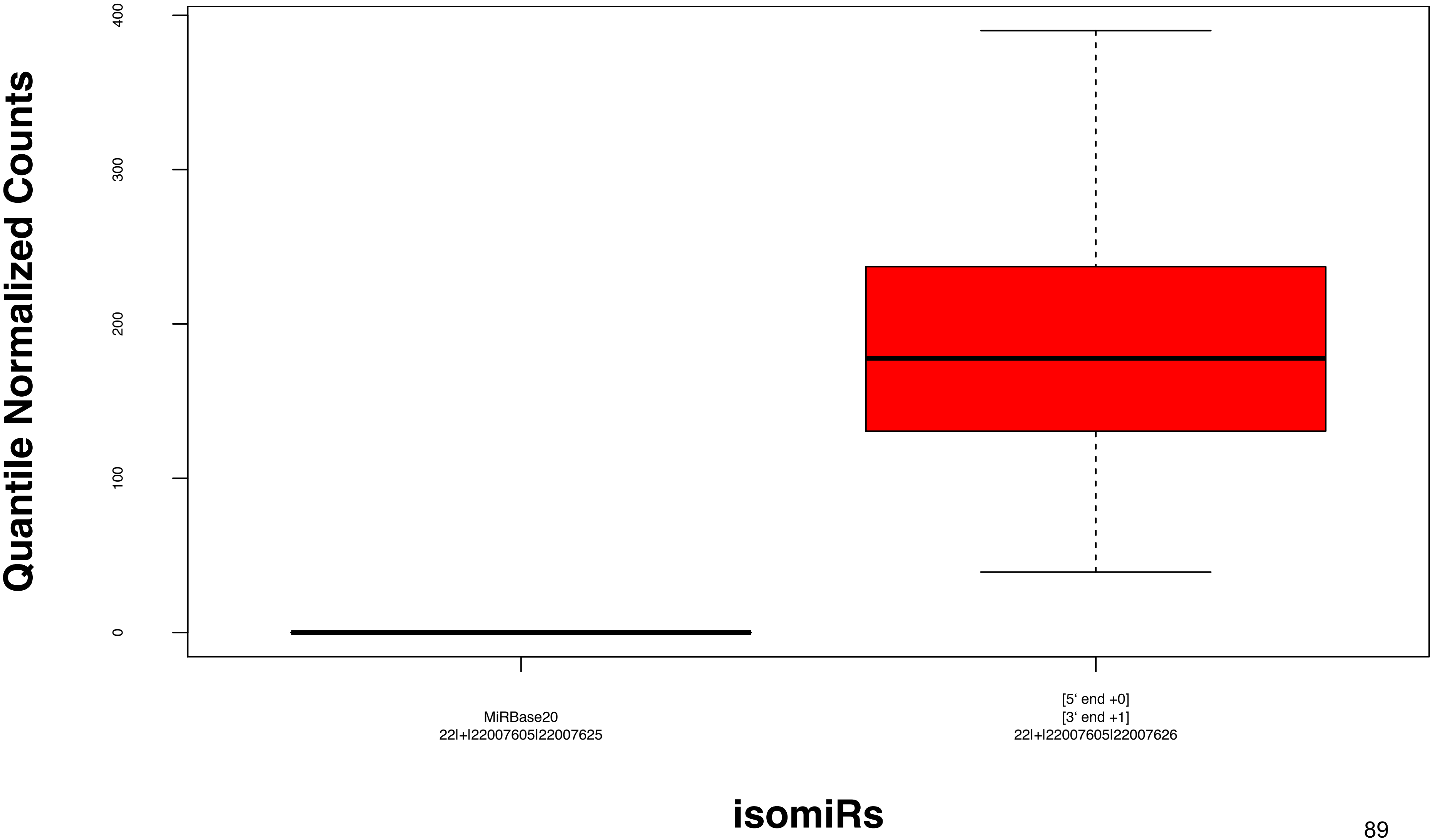

Supp. Figure 1
(pages 1 to 194)

MIMAT0000087=hsa−miR−30a−5p=MI0000088

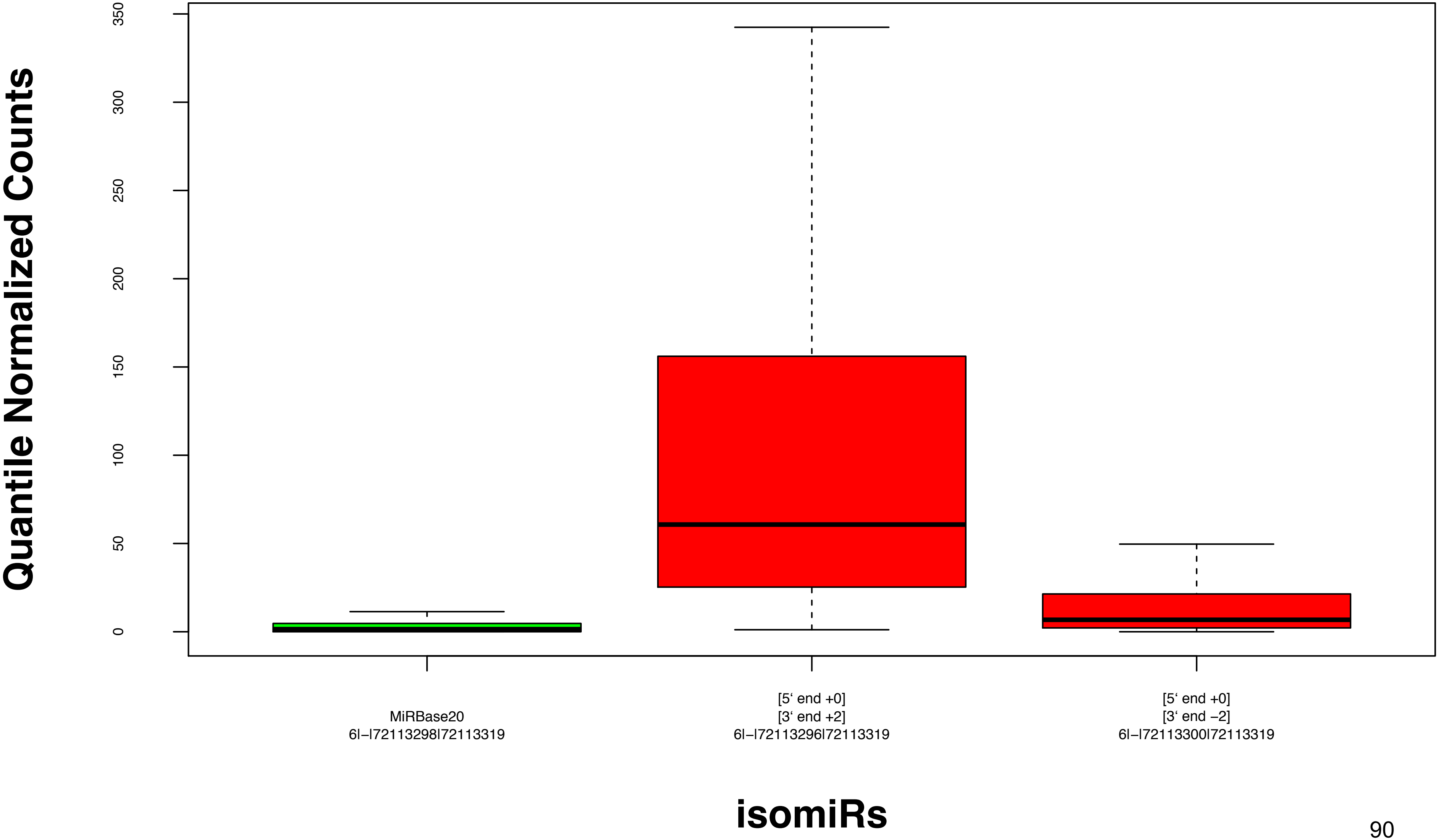

Supp. Figure 1
(pages 1 to 194)

MIMAT0000443=hsa−miR−125a−5p=MI0000469

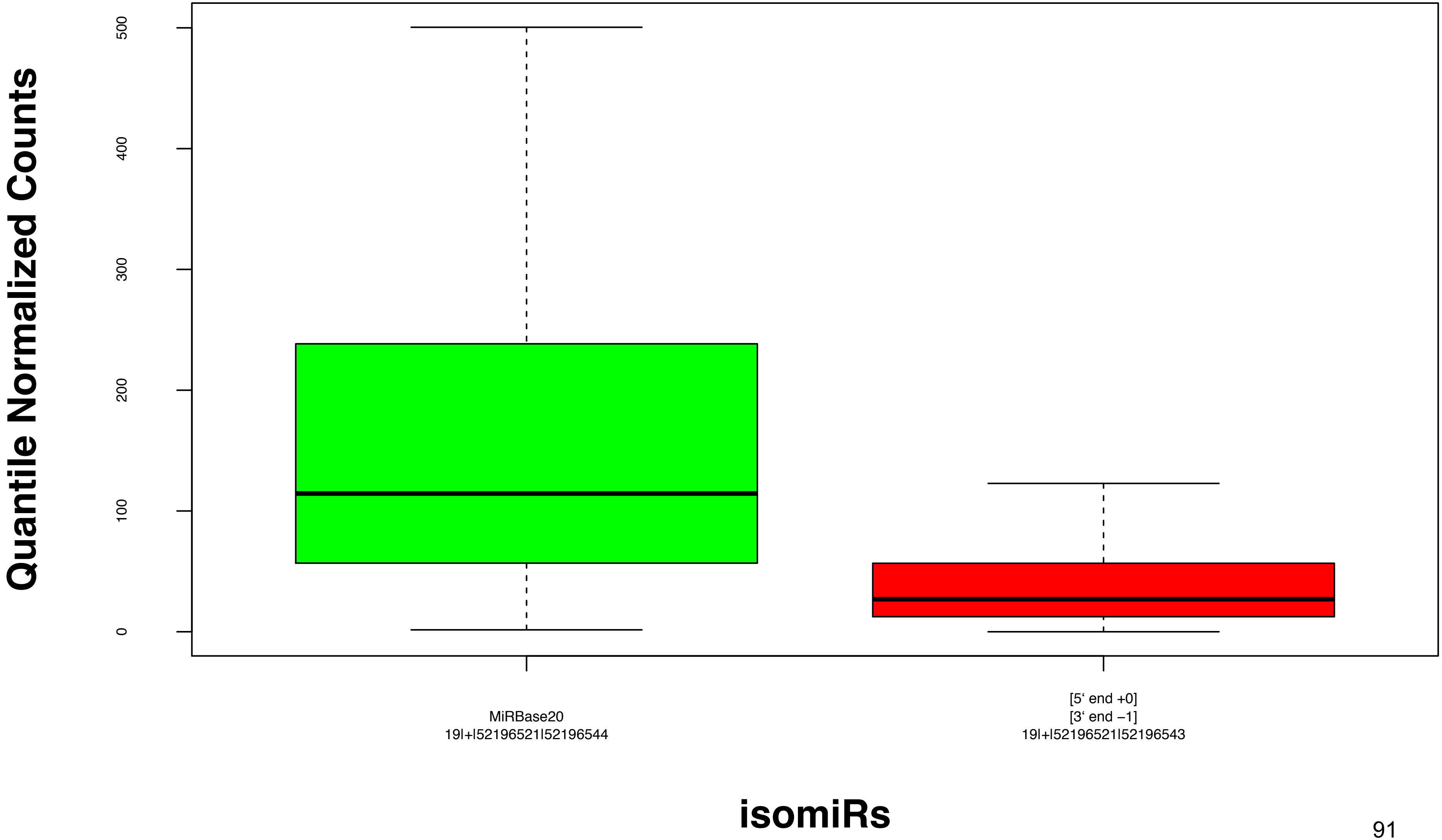

Supp. Figure 1
(pages 1 to 194)

MIMAT0004559=hsa−miR−181c−3p=MI0000271

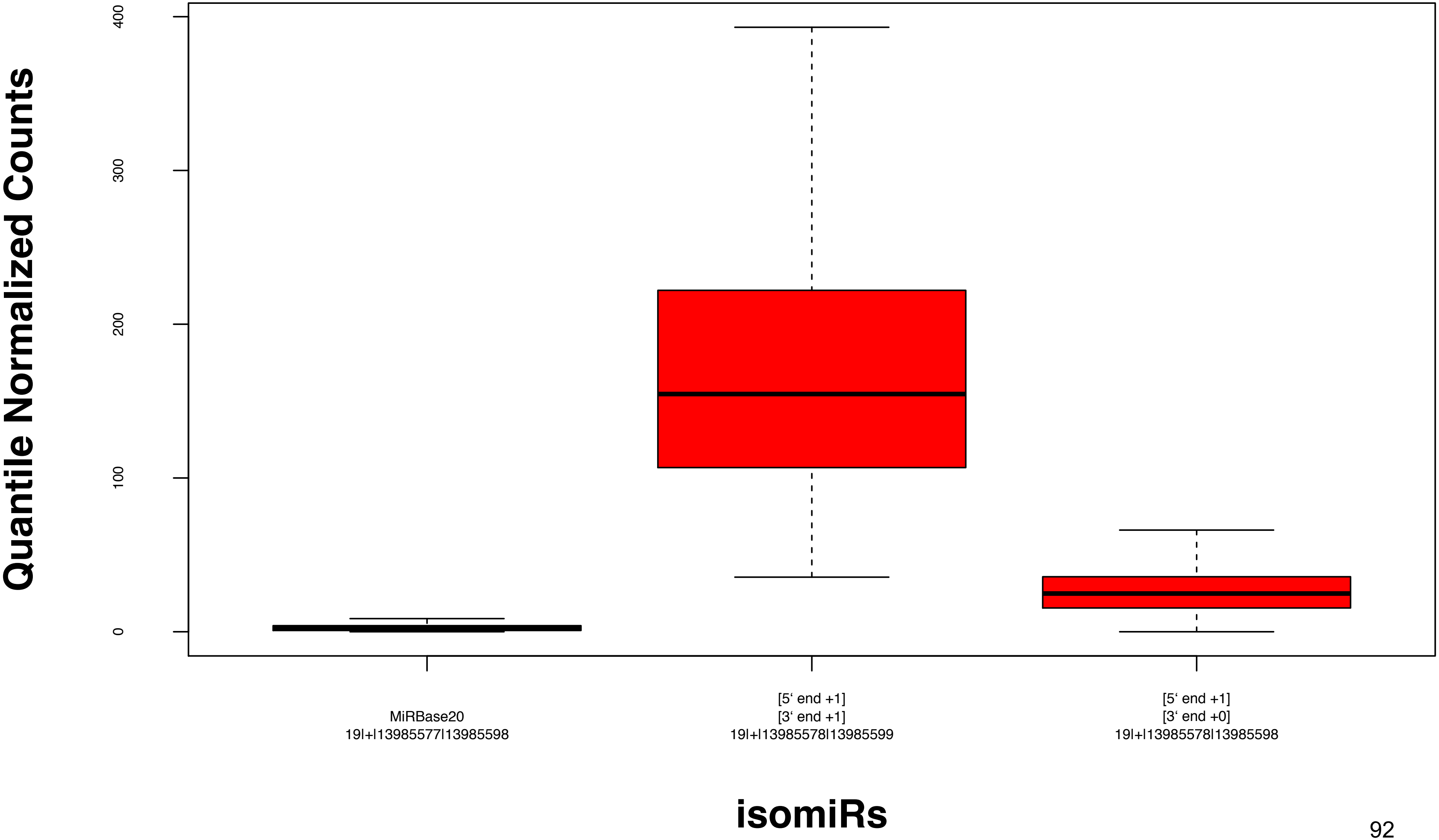

Supp. Figure 1
(pages 1 to 194)

MIMAT0000680=hsa–miR–106b–5p=MI0000734

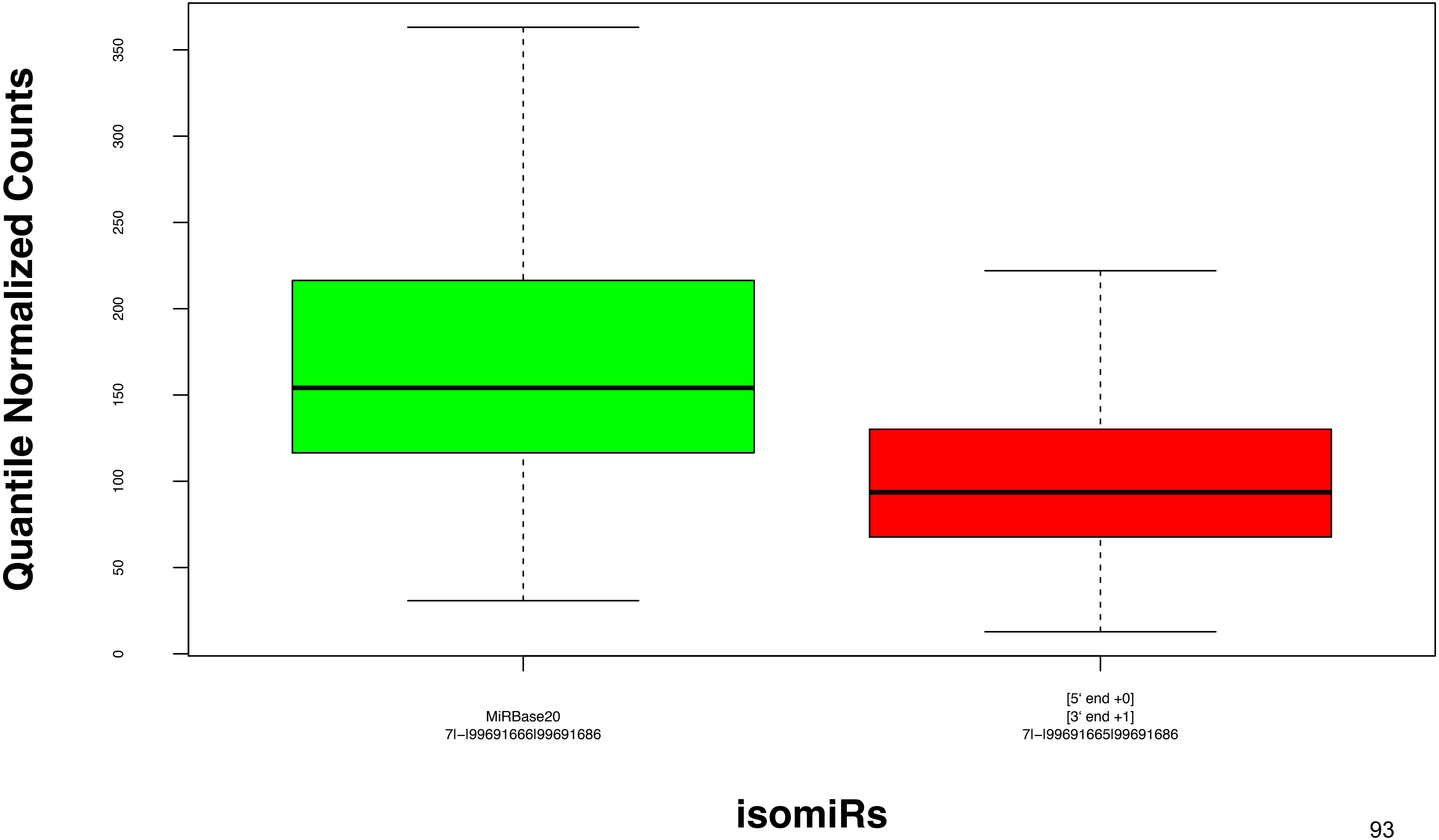

Supp. Figure 1
(pages 1 to 194)

MIMAT0000261=hsa−miR−183−5p=MI0000273

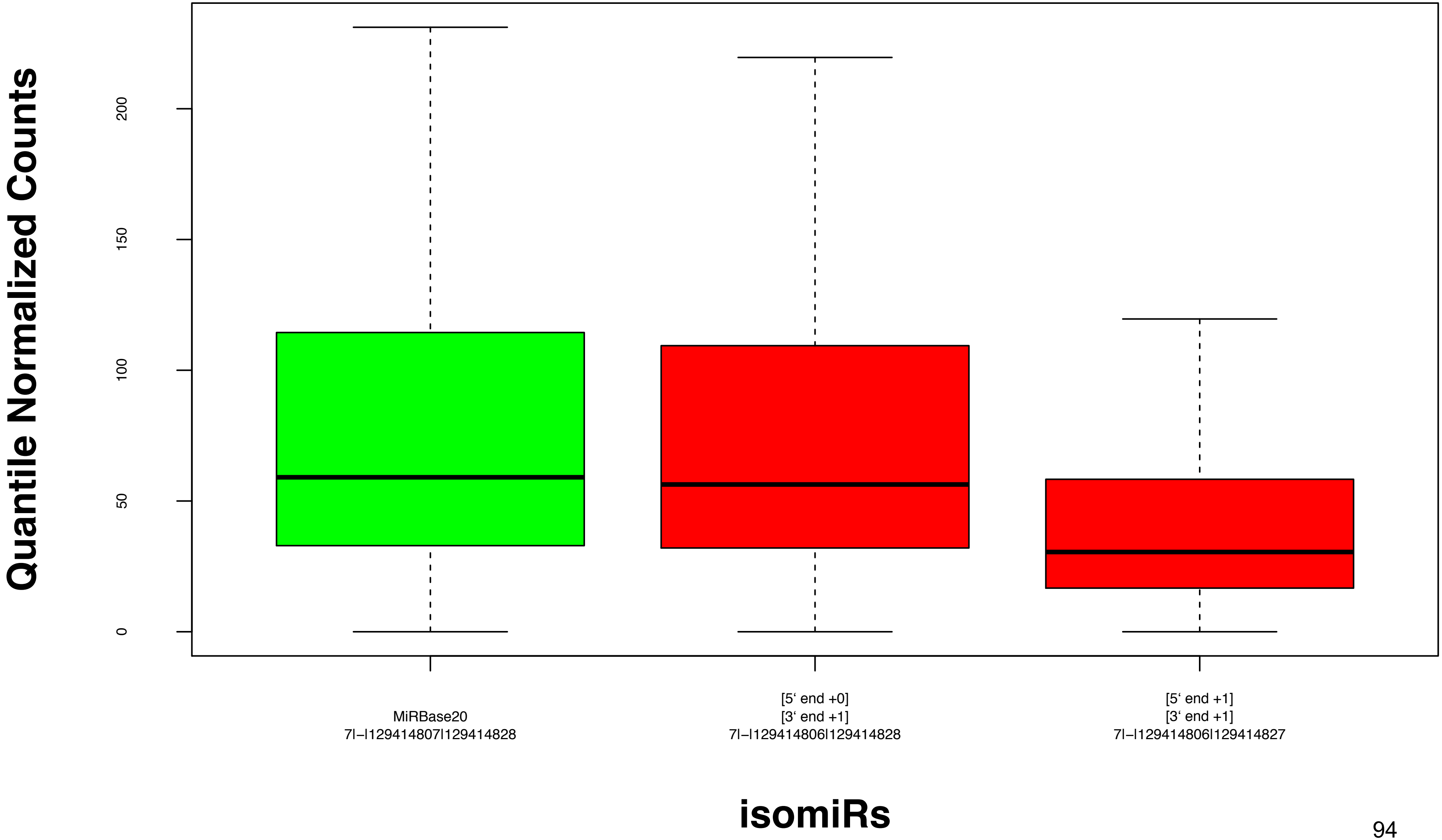

Supp. Figure 1
(pages 1 to 194)

MIMAT0004518=hsa−miR−16−2−3p=MI0000115

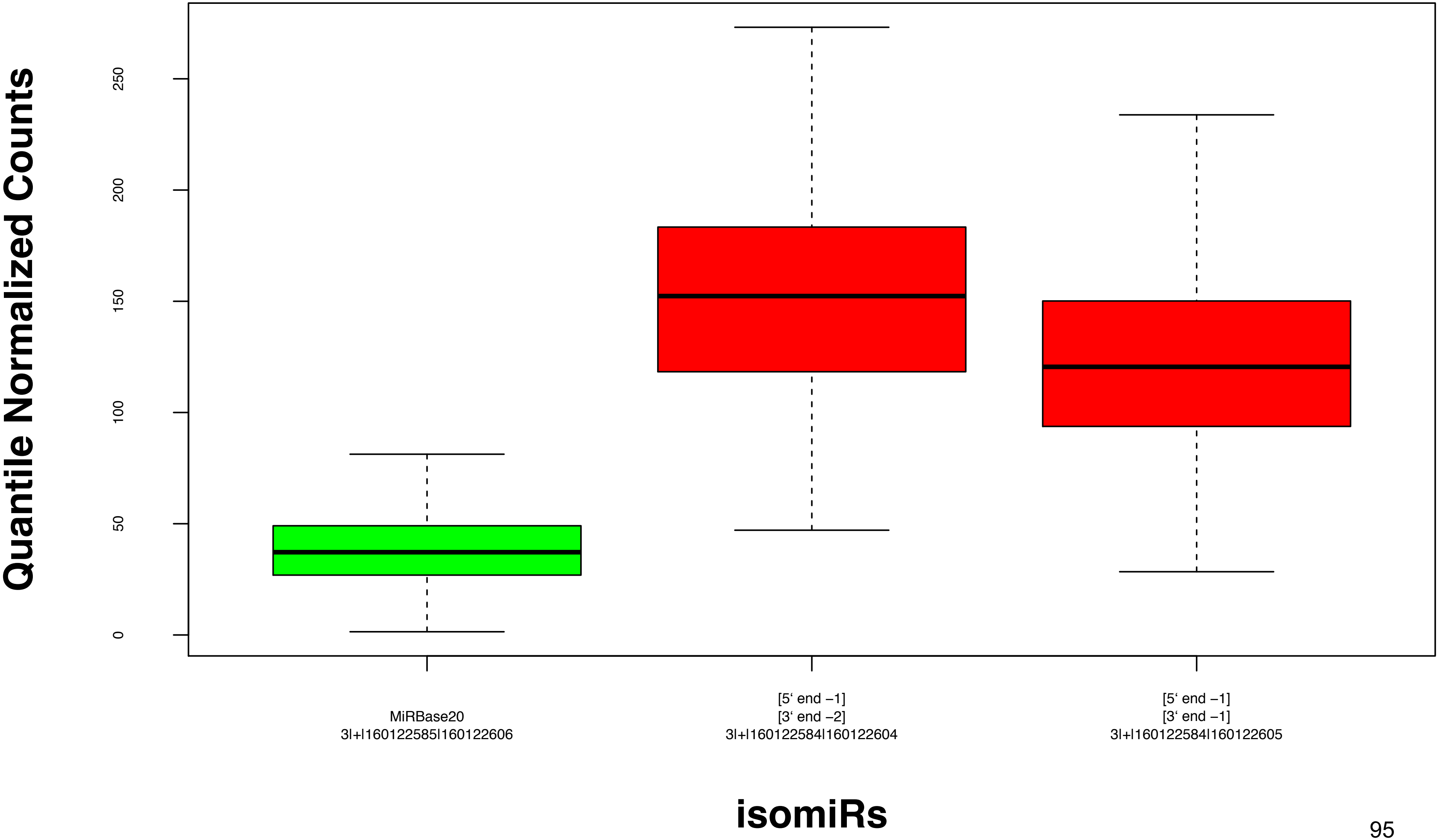

Supp. Figure 1
(pages 1 to 194)

MIMAT0000104=hsa−miR−107=MI0000114

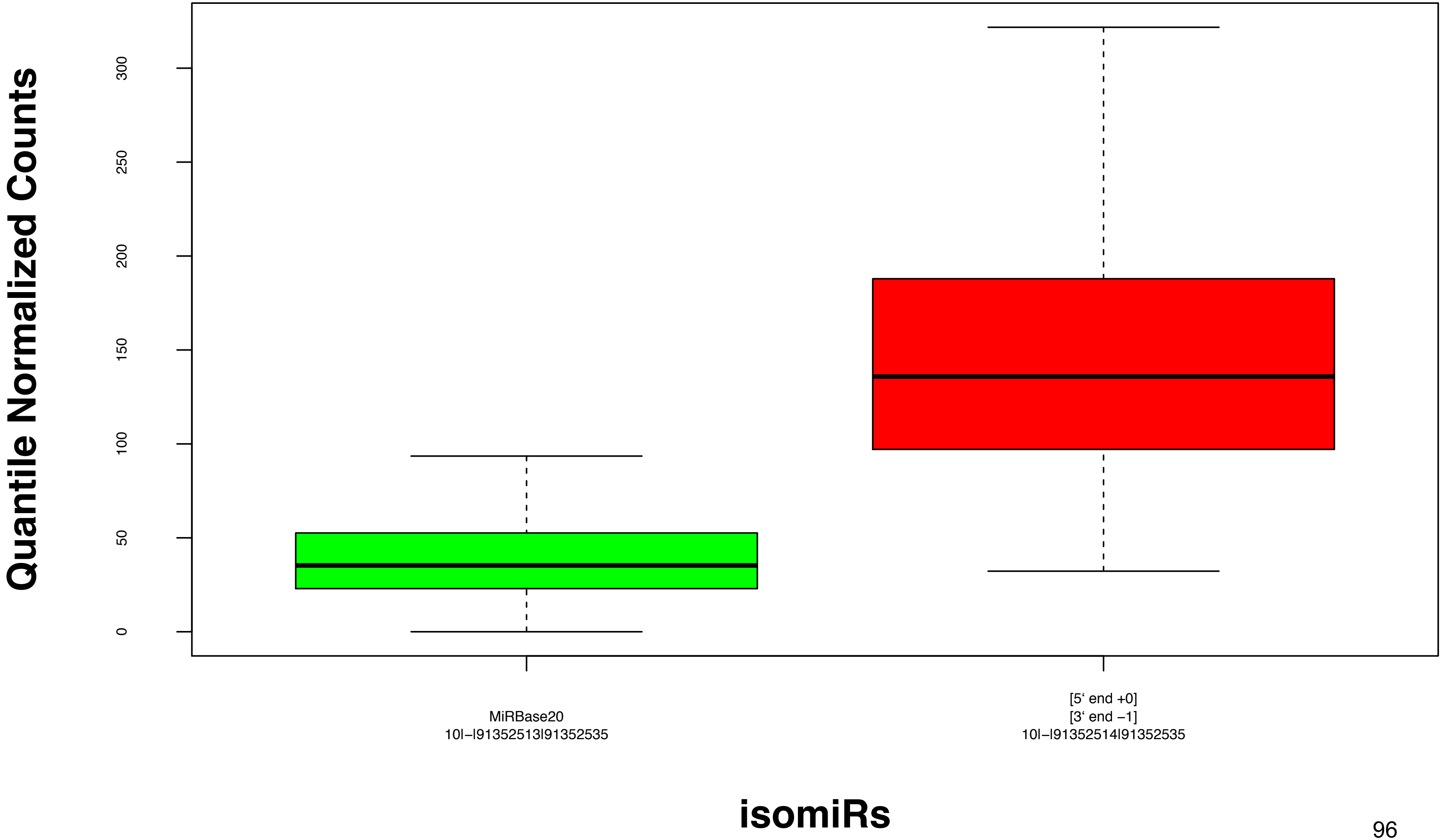

Supp. Figure 1
(pages 1 to 194)

MIMAT0000073=hsa−miR−19a−3p=MI0000073

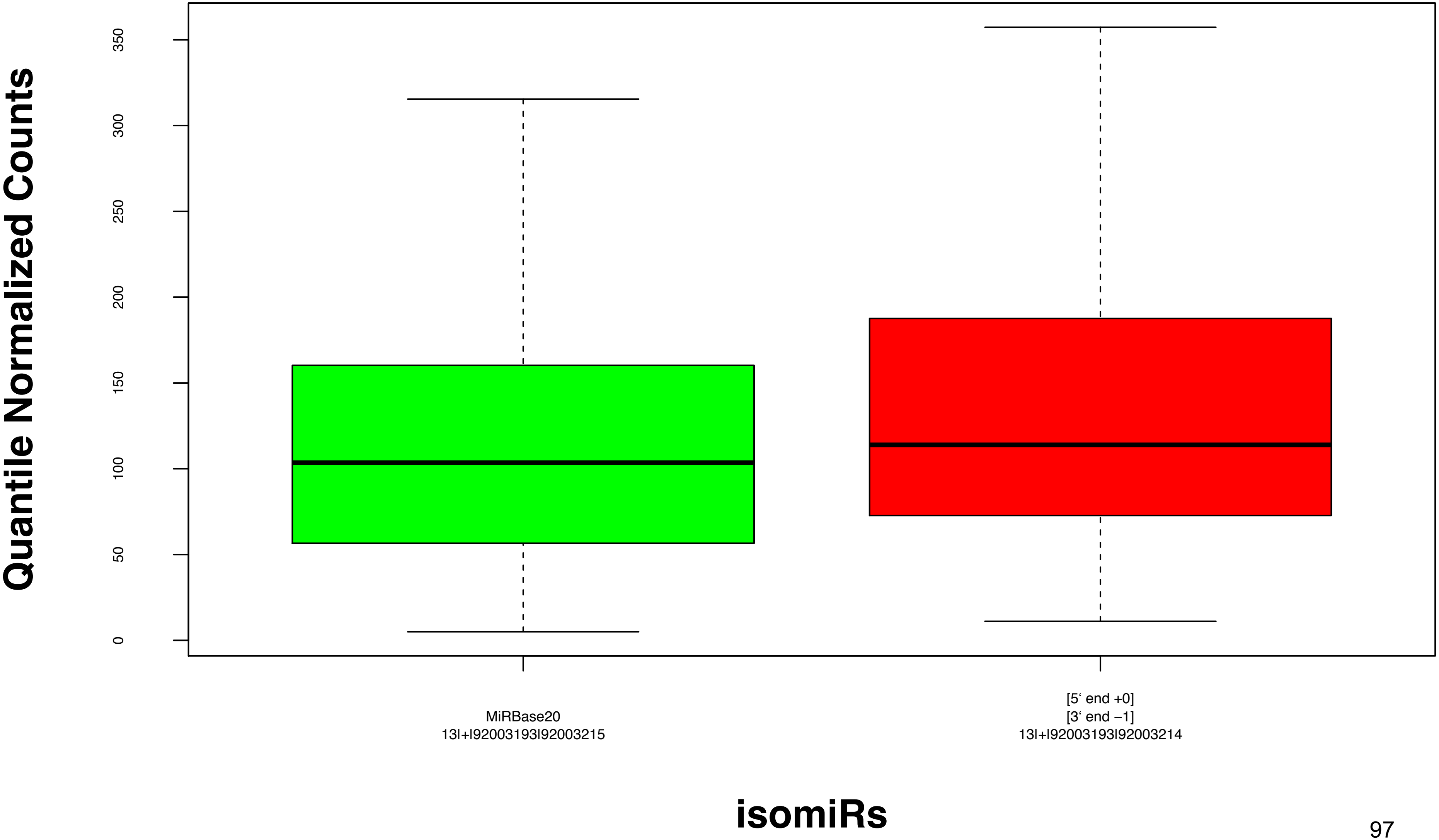

Supp. Figure 1
(pages 1 to 194)

MIMAT0005949=hsa−miR−664a−3p=MI0006442

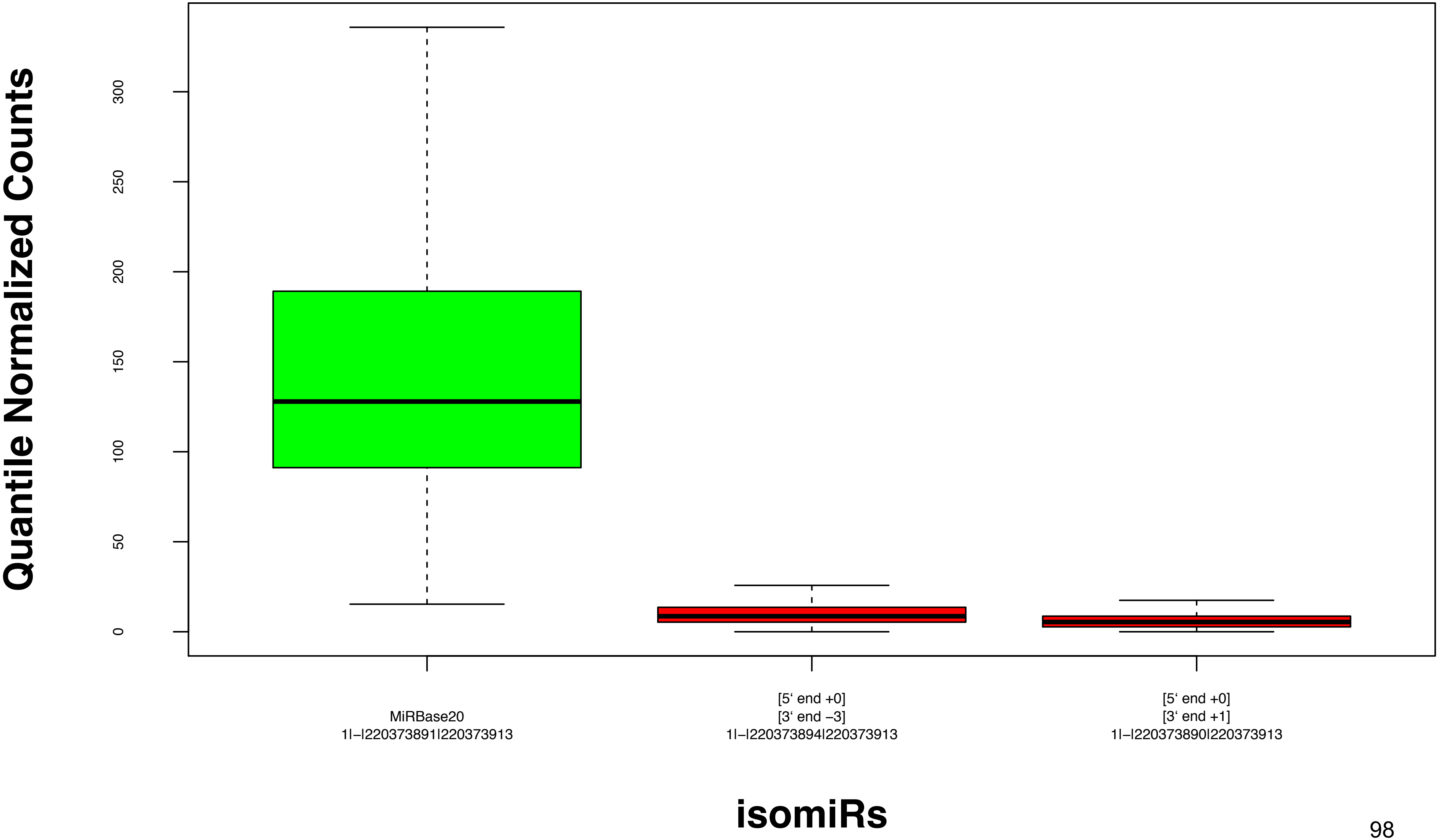

Supp. Figure 1
(pages 1 to 194)

MIMAT0027587=hsa–miR–6842–3p=MI0022688

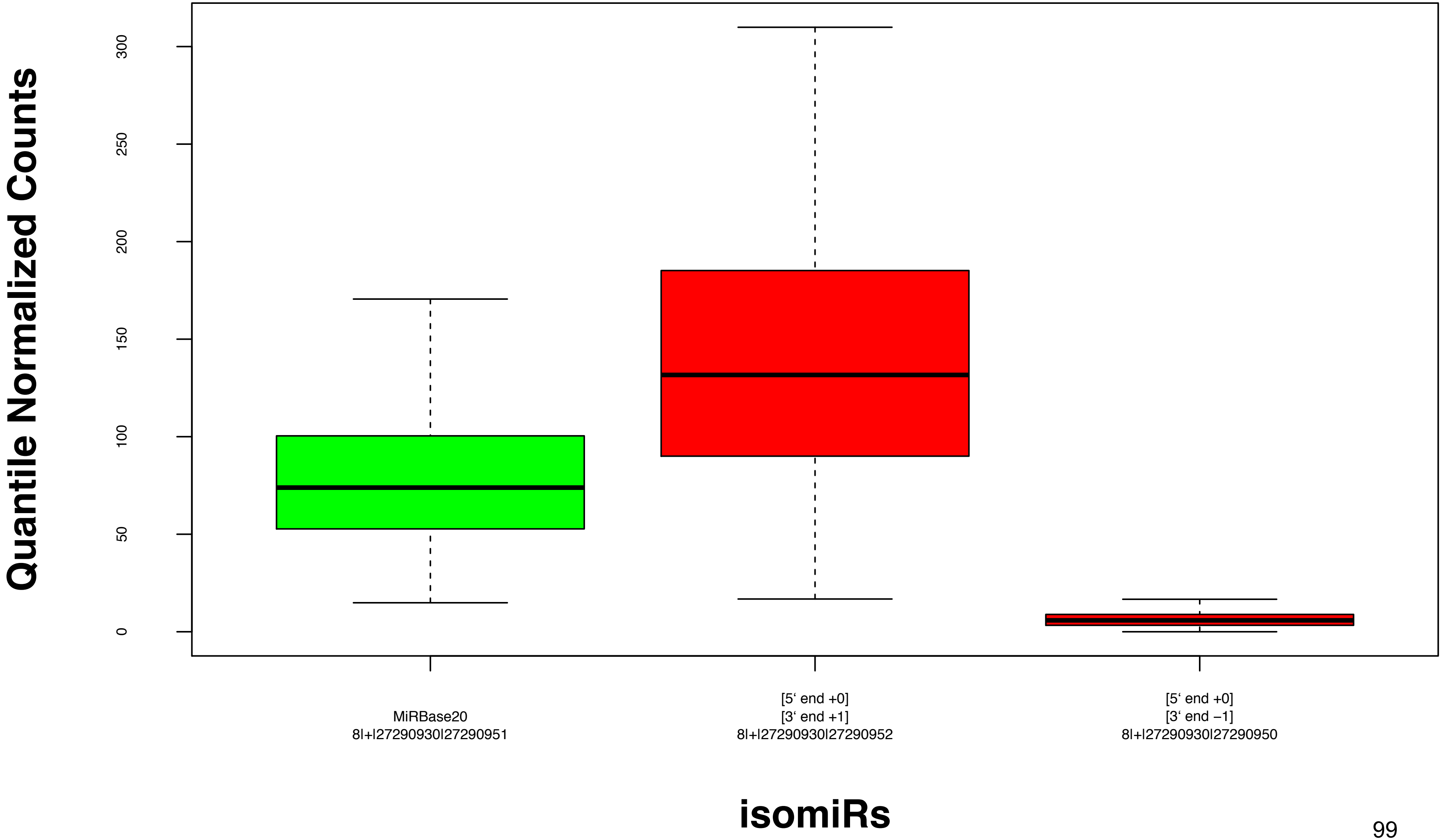

Supp. Figure 1
(pages 1 to 194)

MIMAT0004958=hsa-miR-301b=MI0005568

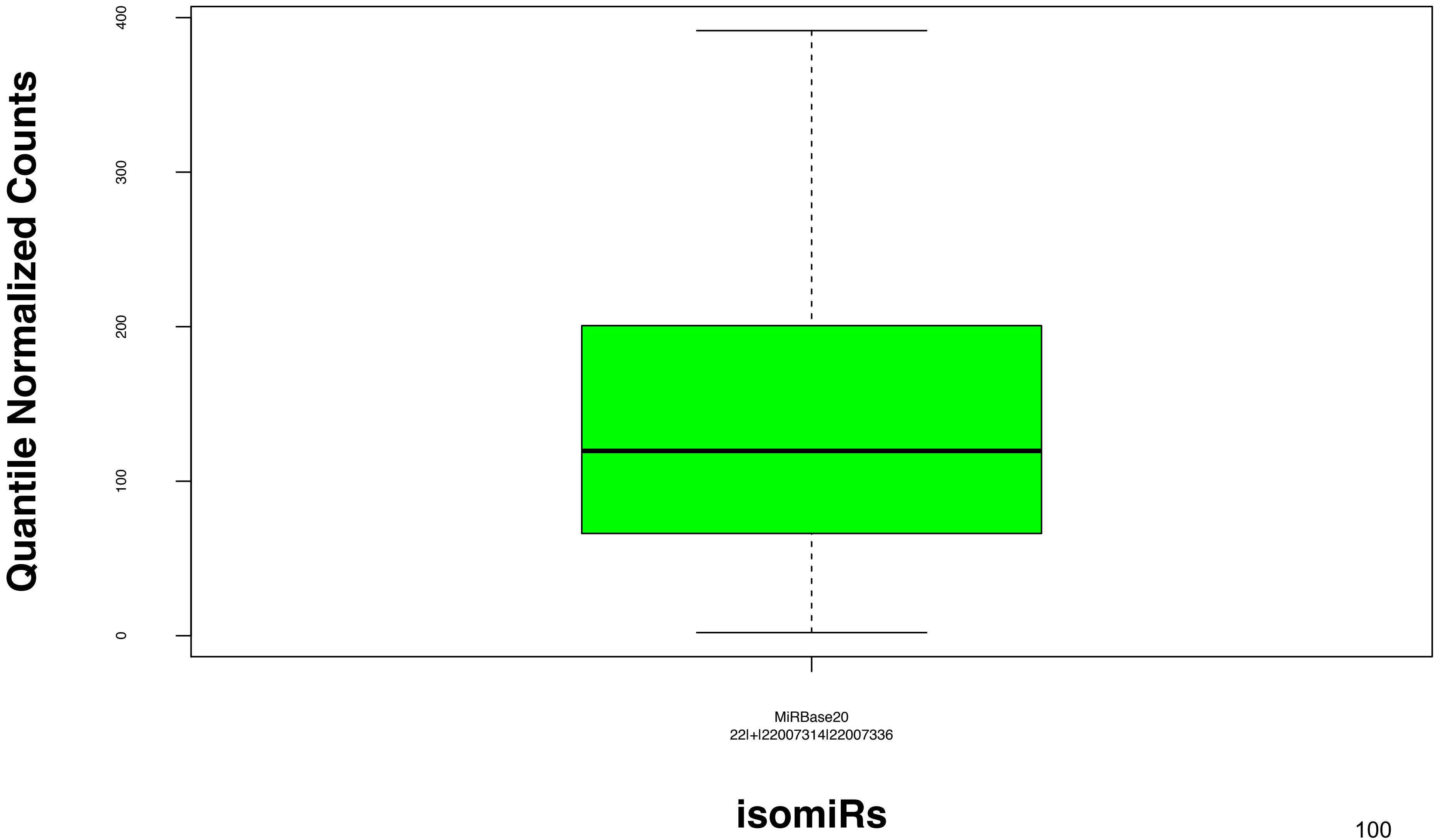

Supp. Figure 1
(pages 1 to 194)

MIMAT0003888=hsa−miR−766−3p=MI0003836

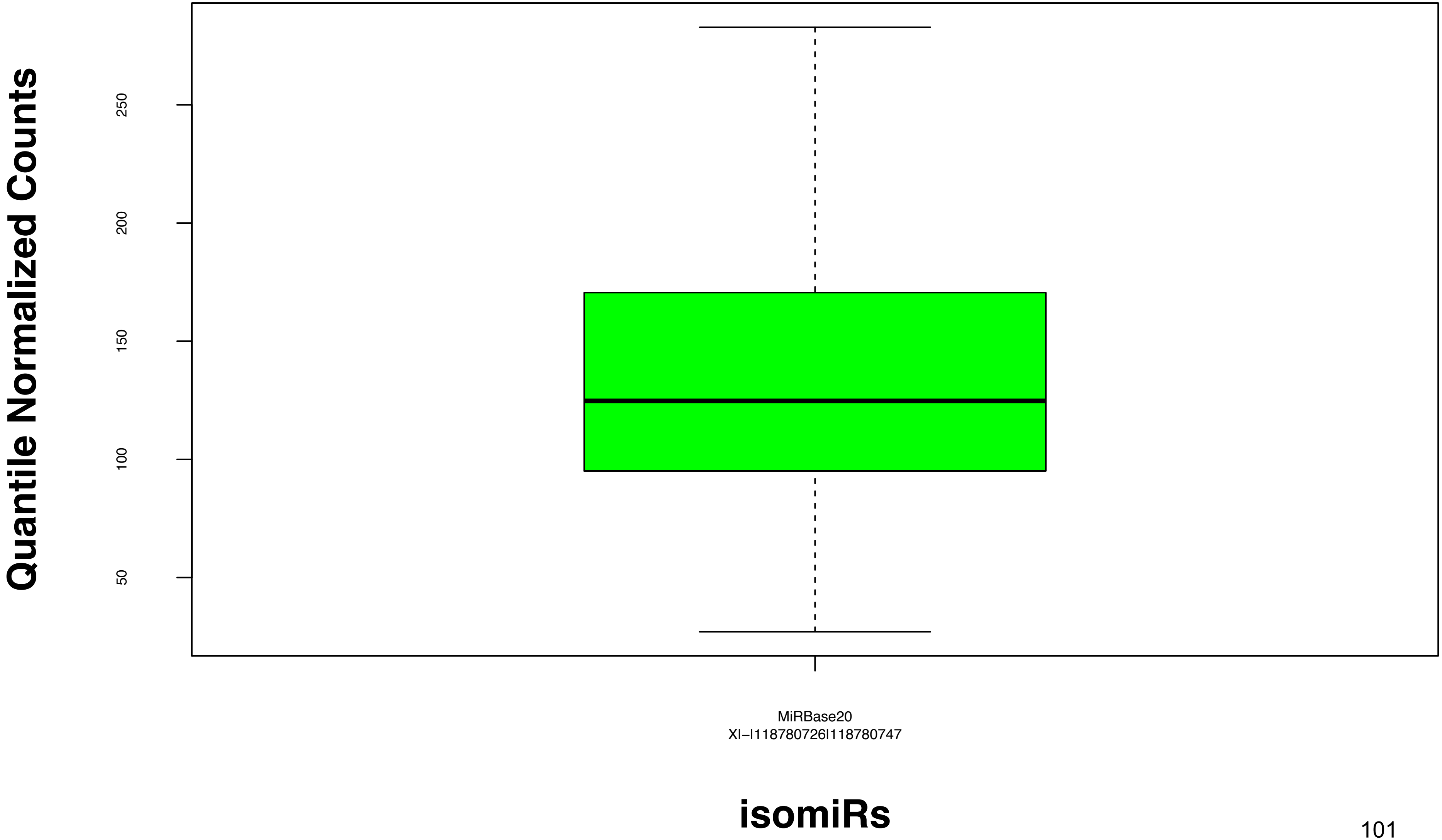

Supp. Figure 1
(pages 1 to 194)

MIMAT0004501=hsa−miR−27a−5p=MI0000085

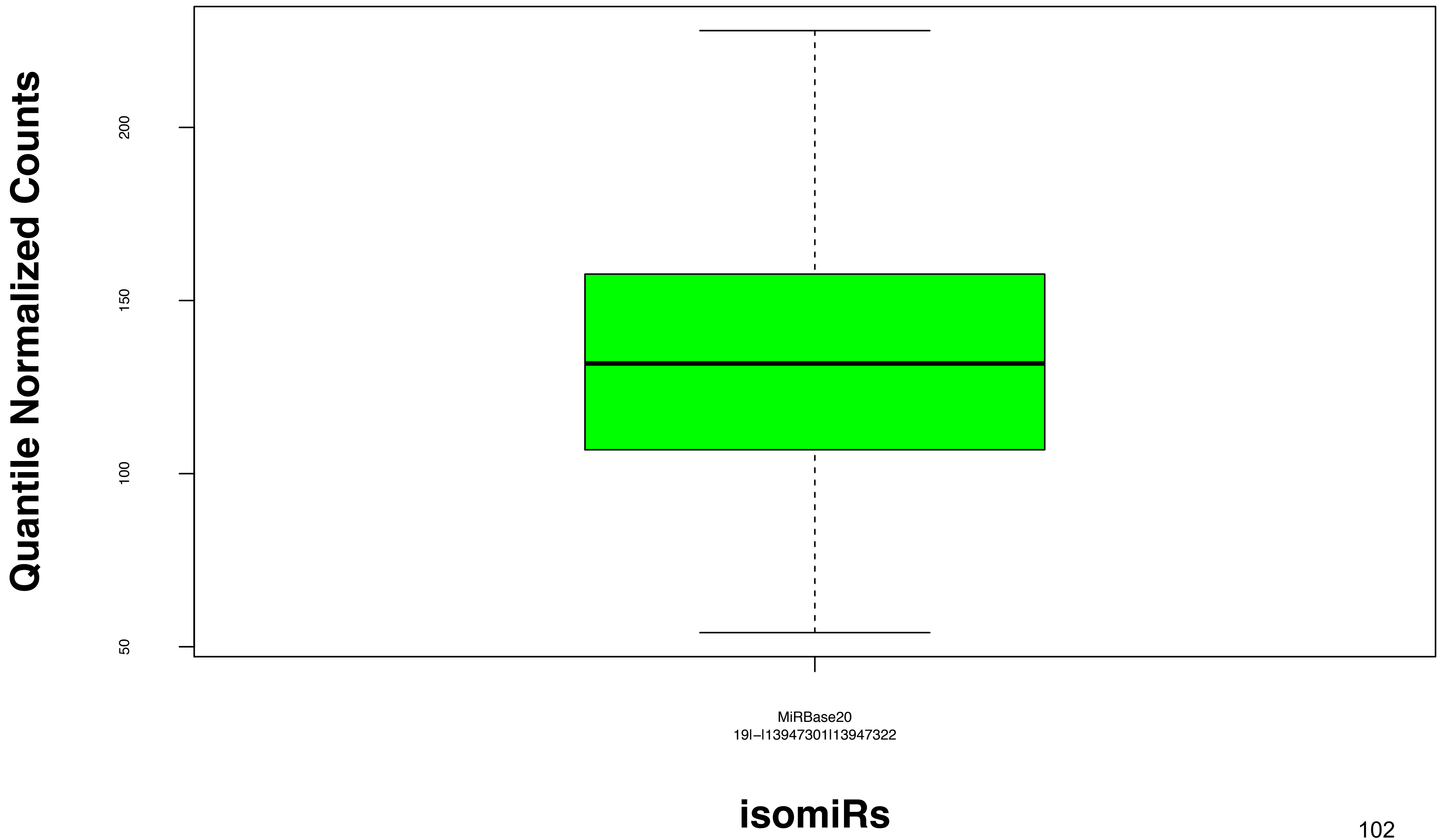

Supp. Figure 1
(pages 1 to 194)

MIMAT0004507=hsa–miR–92a–1–5p=MI0000093

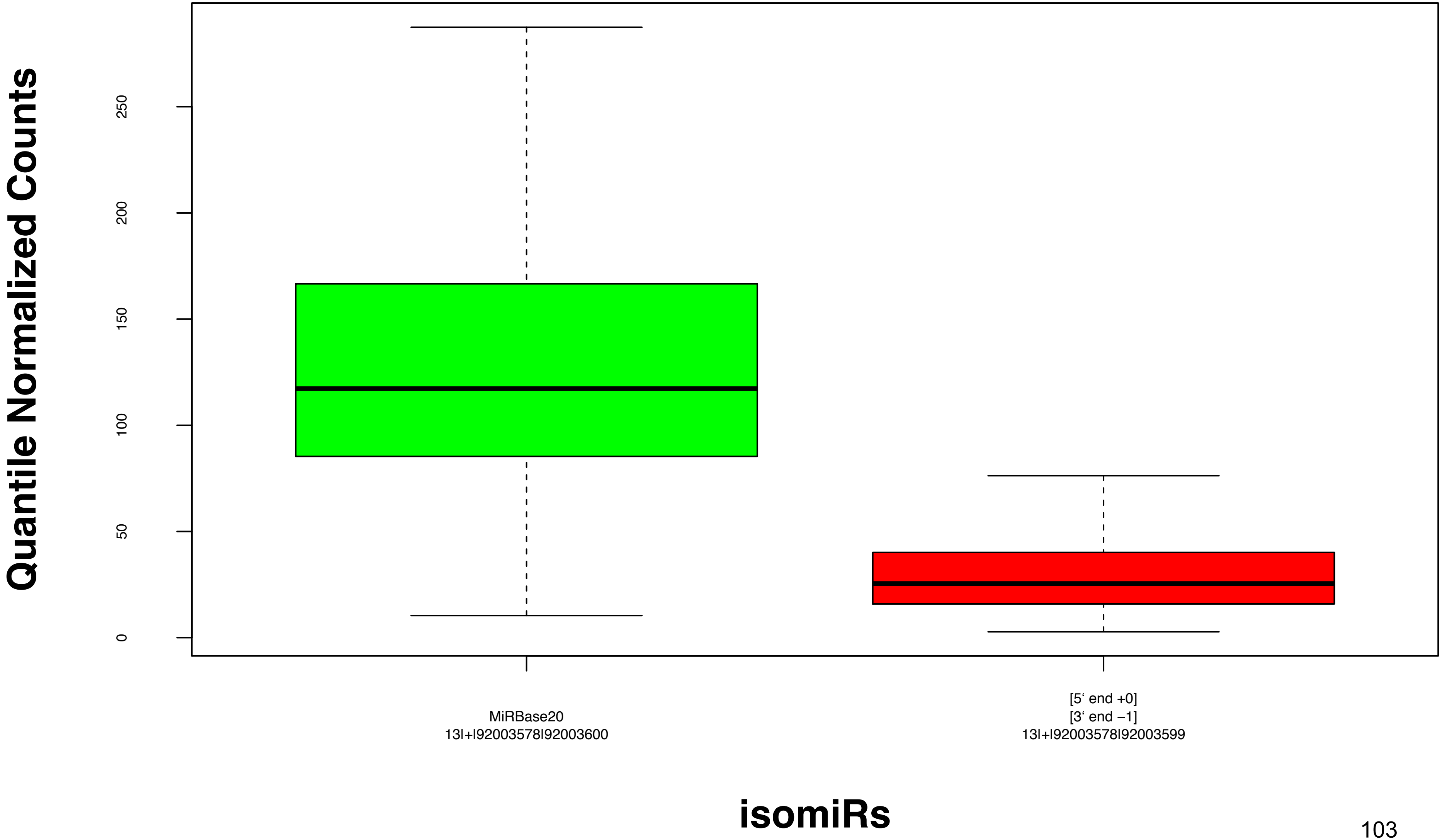

Supp. Figure 1
(pages 1 to 194)

MIMAT0003339=hsa−miR−421=MI0003685

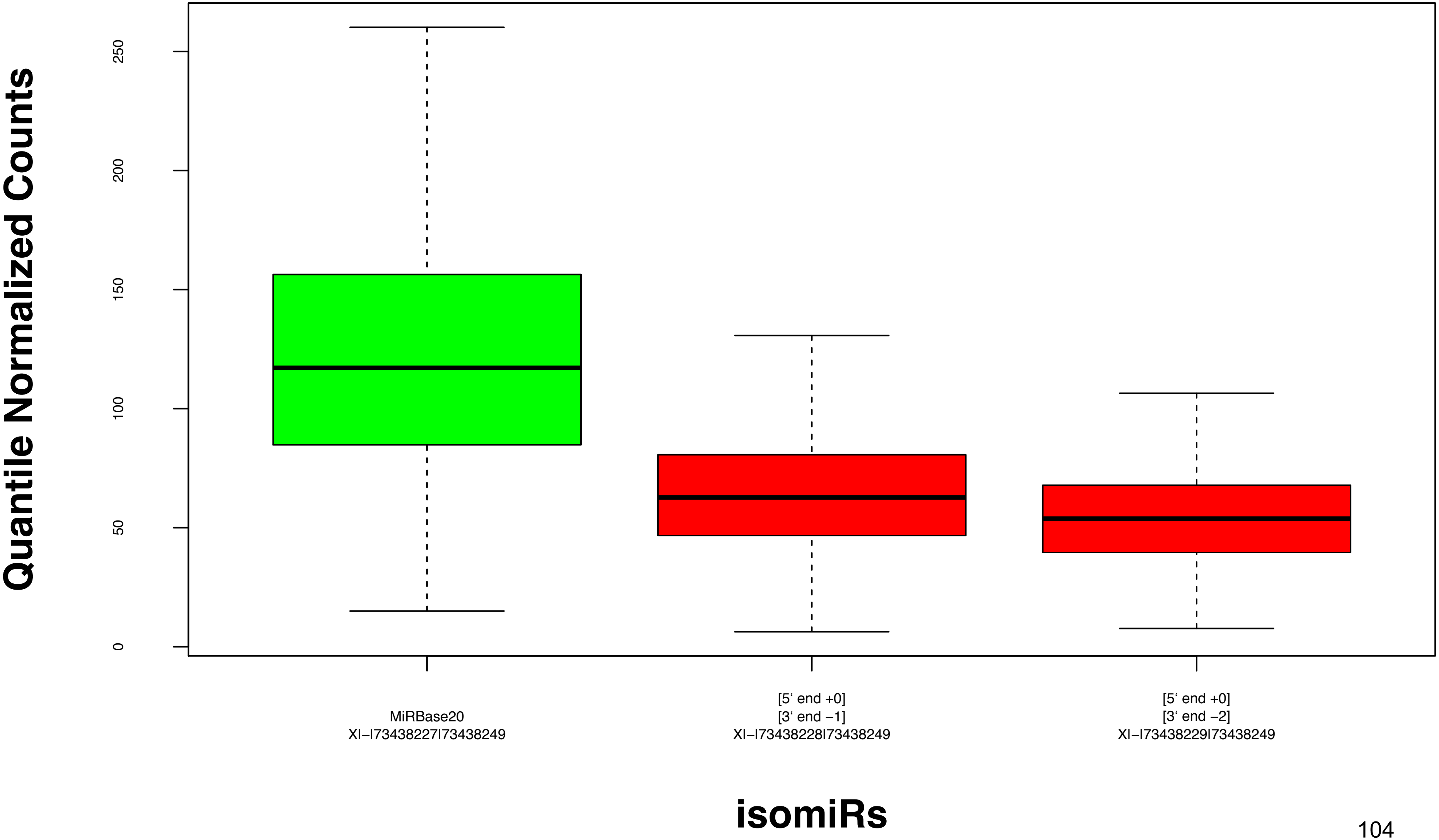

Supp. Figure 1
(pages 1 to 194)

MIMAT0004945=hsa−miR−744−5p=MI0005559

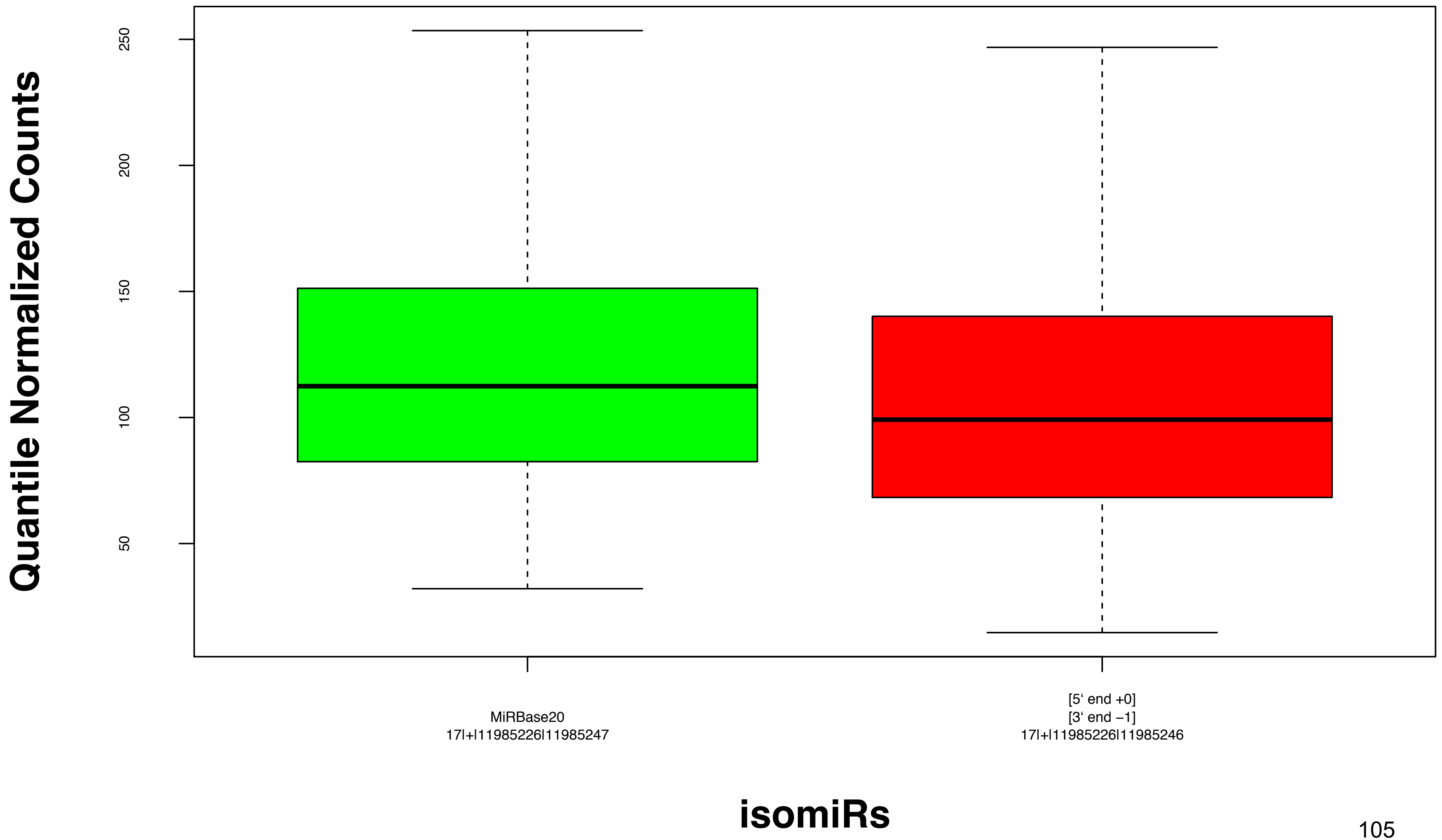

Supp. Figure 1
(pages 1 to 194)

MIMAT0004551=hsa–miR–30d–3p=MI0000255

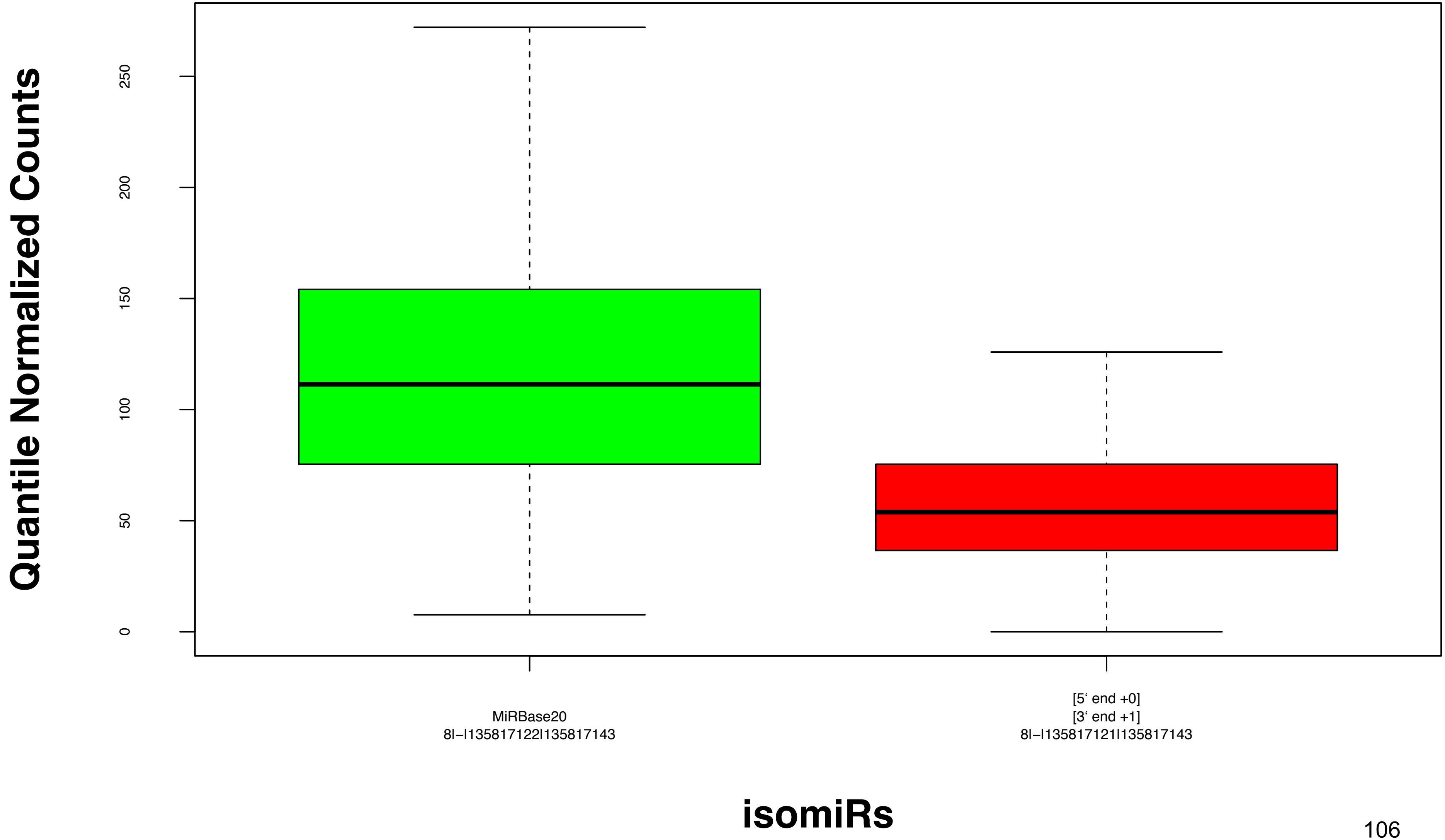

MIMAT0000760=hsa−miR−331−3p=MI0000812

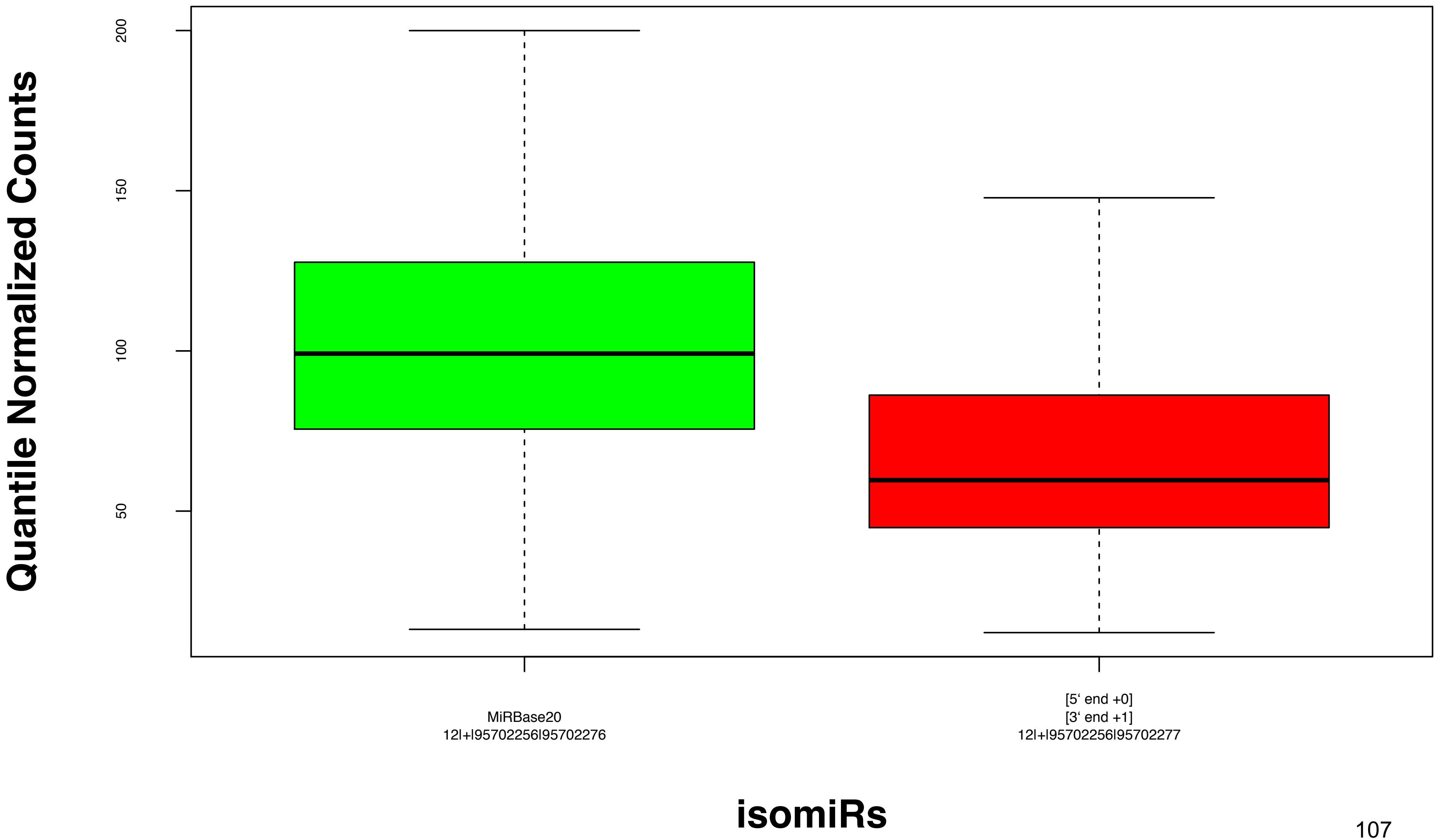

Supp. Figure 1
(pages 1 to 194)

MIMAT0003338=hsa−miR−660−5p=MI0003684

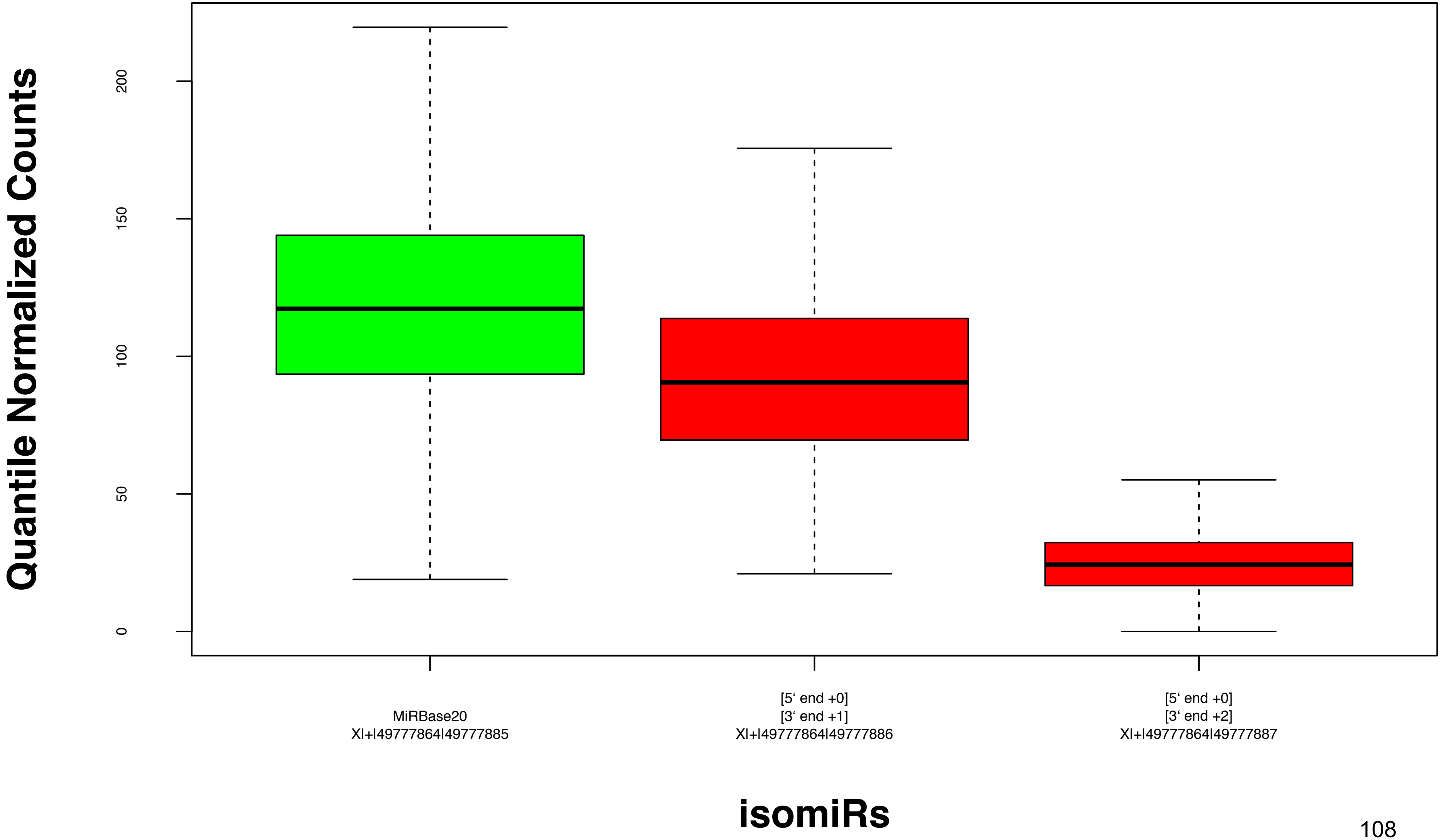

Supp. Figure 1
(pages 1 to 194)

MIMAT0004568=hsa-miR-221-5p=MI0000298

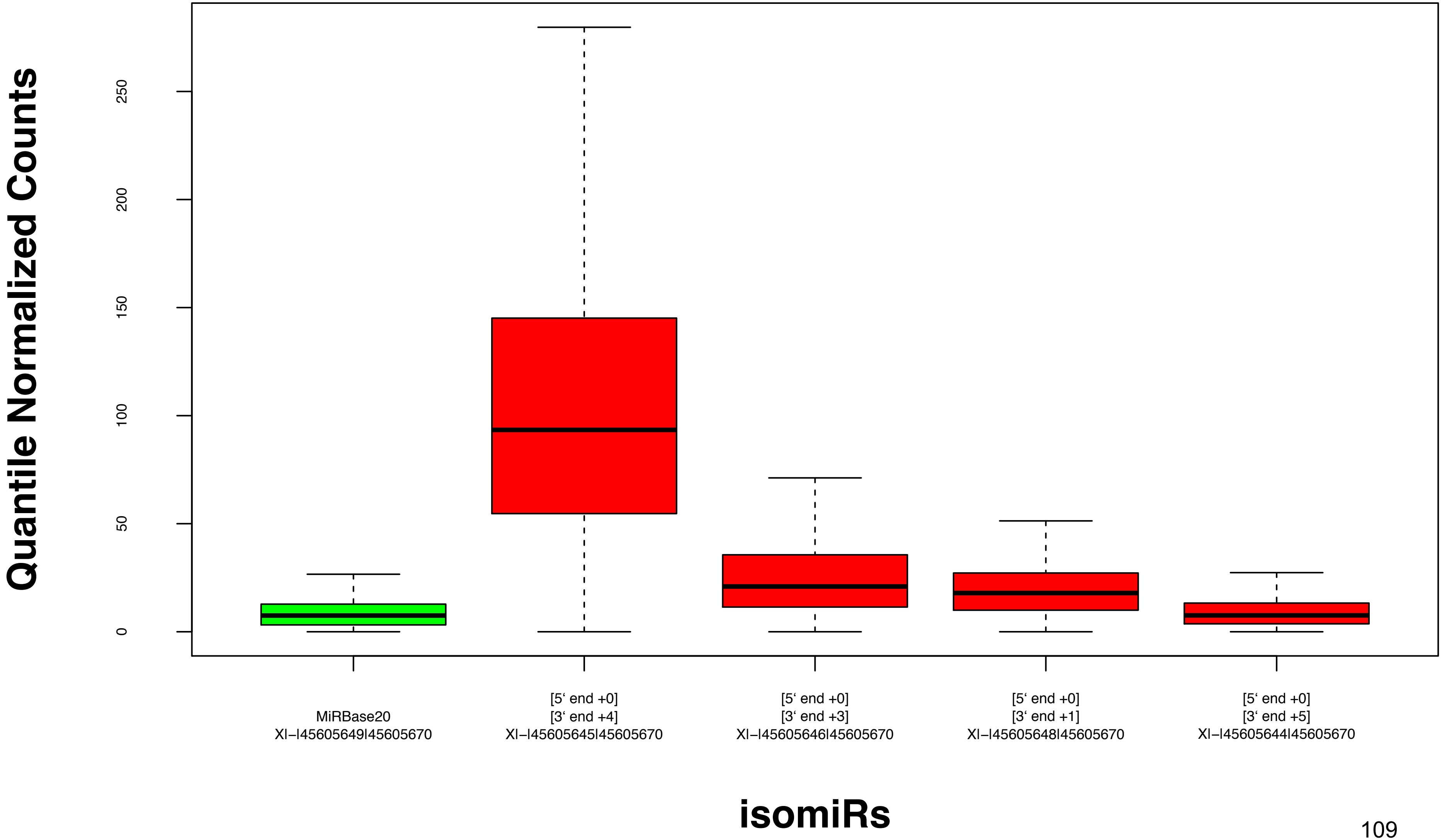

Supp. Figure 1
(pages 1 to 194)

MIMAT0004699=hsa–miR–148b–5p=MI0000811

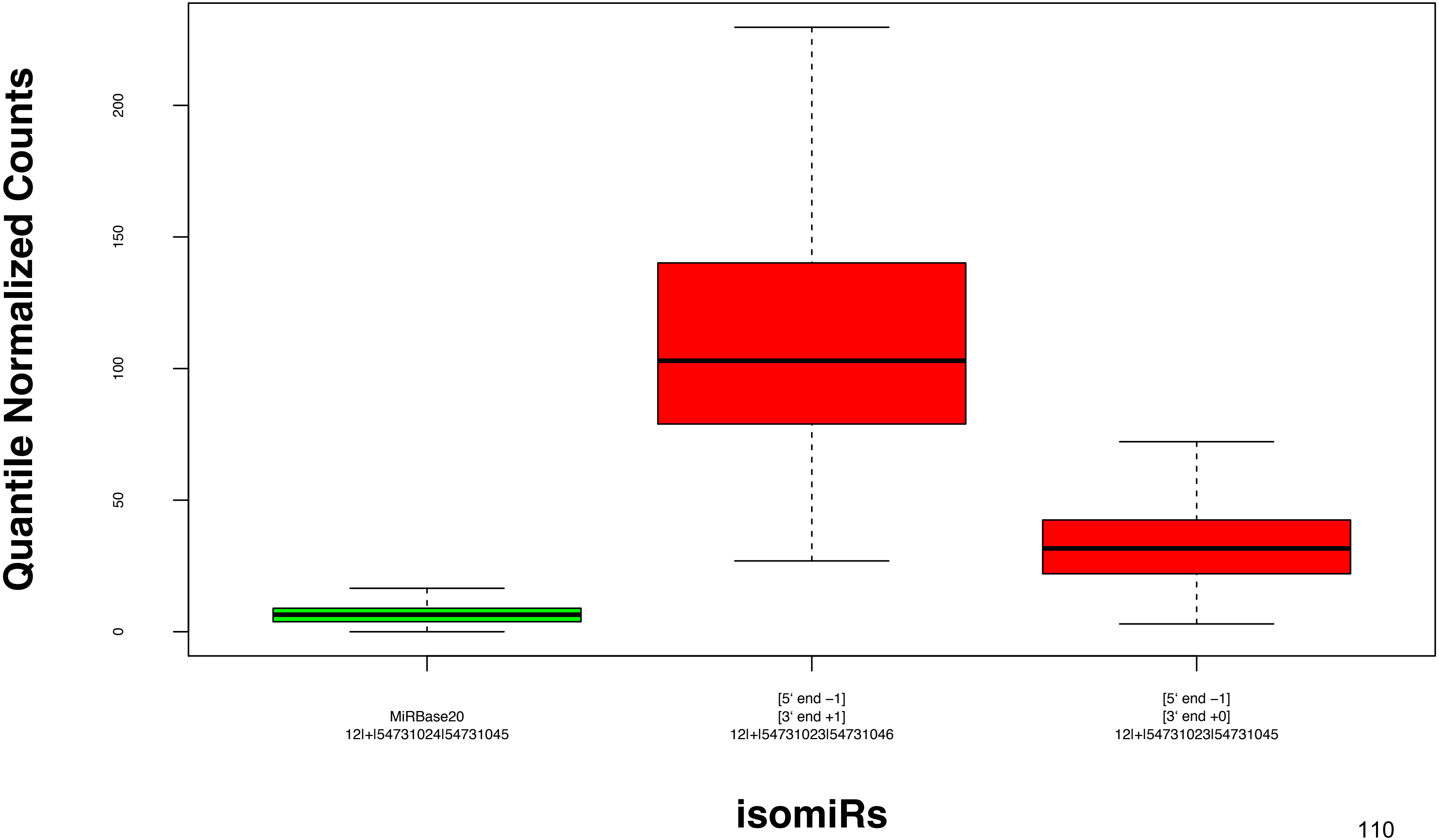

Supp. Figure 1
(pages 1 to 194)

MIMAT0000067=hsa–let–7f–5p=MI0000067

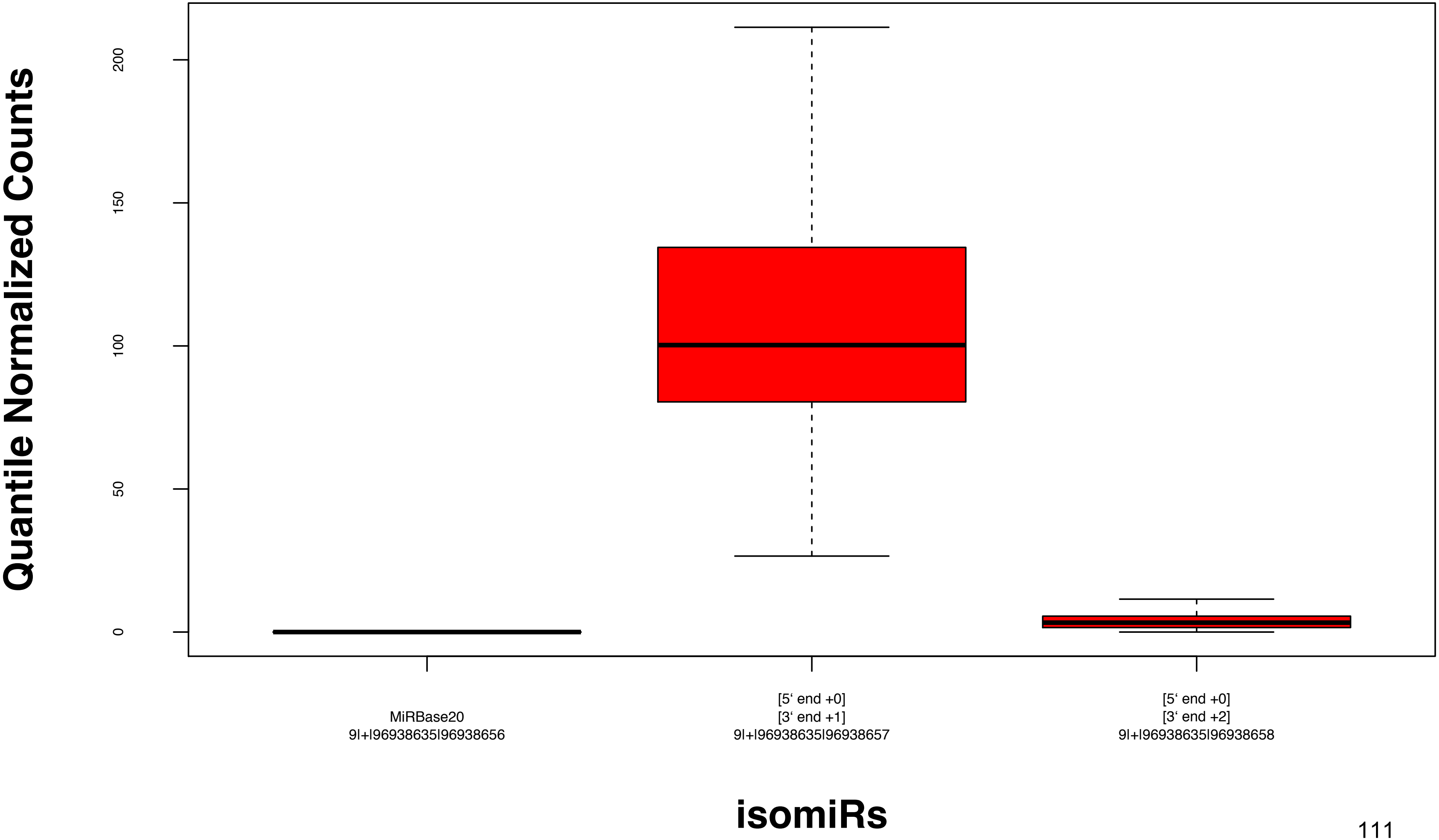

Supp. Figure 1
(pages 1 to 194)

MIMAT0004703=hsa−miR−335−3p=MI0000816

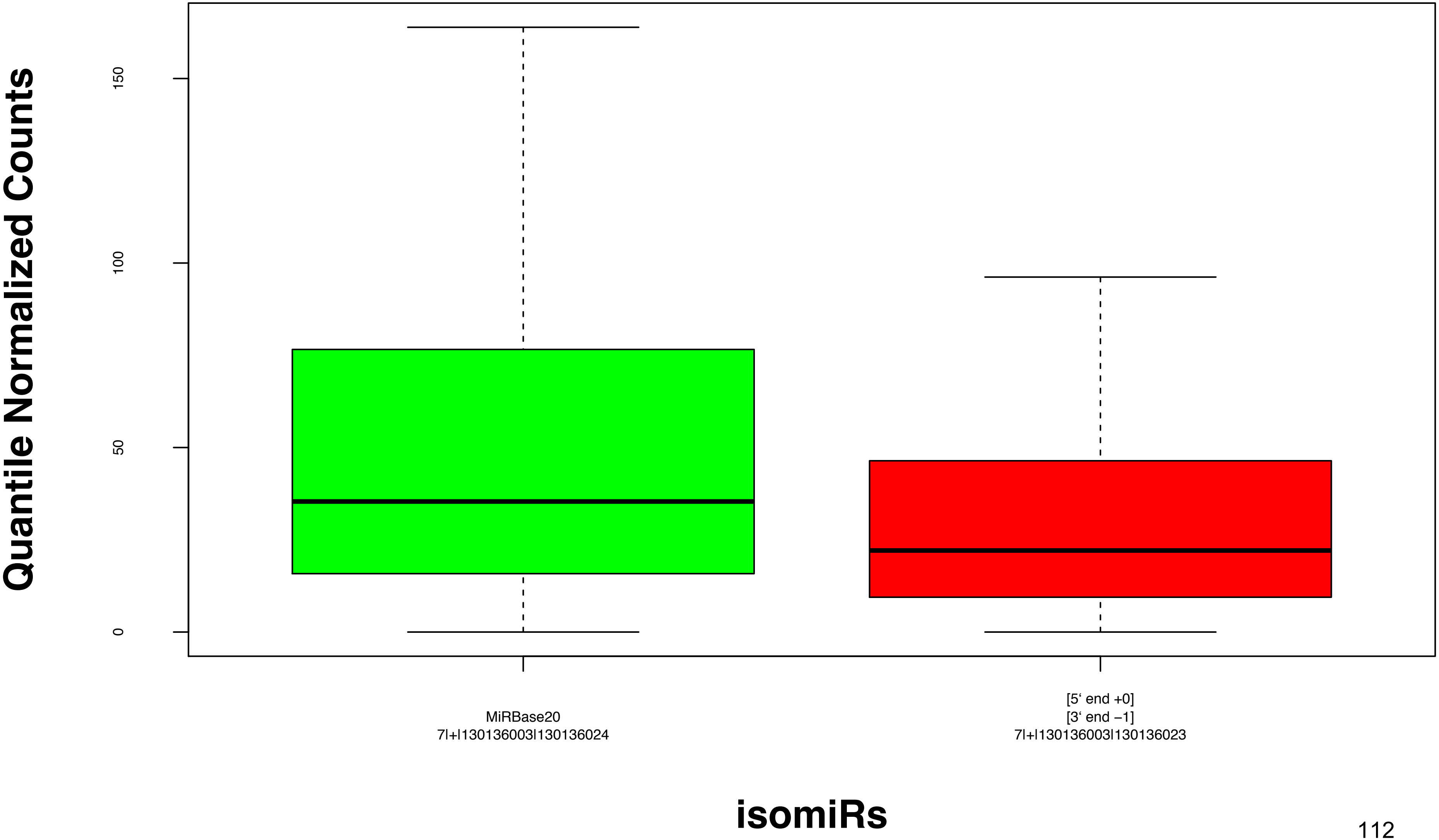

Supp. Figure 1
(pages 1 to 194)

MIMAT0000254=hsa−miR−10b−5p=MI0000267

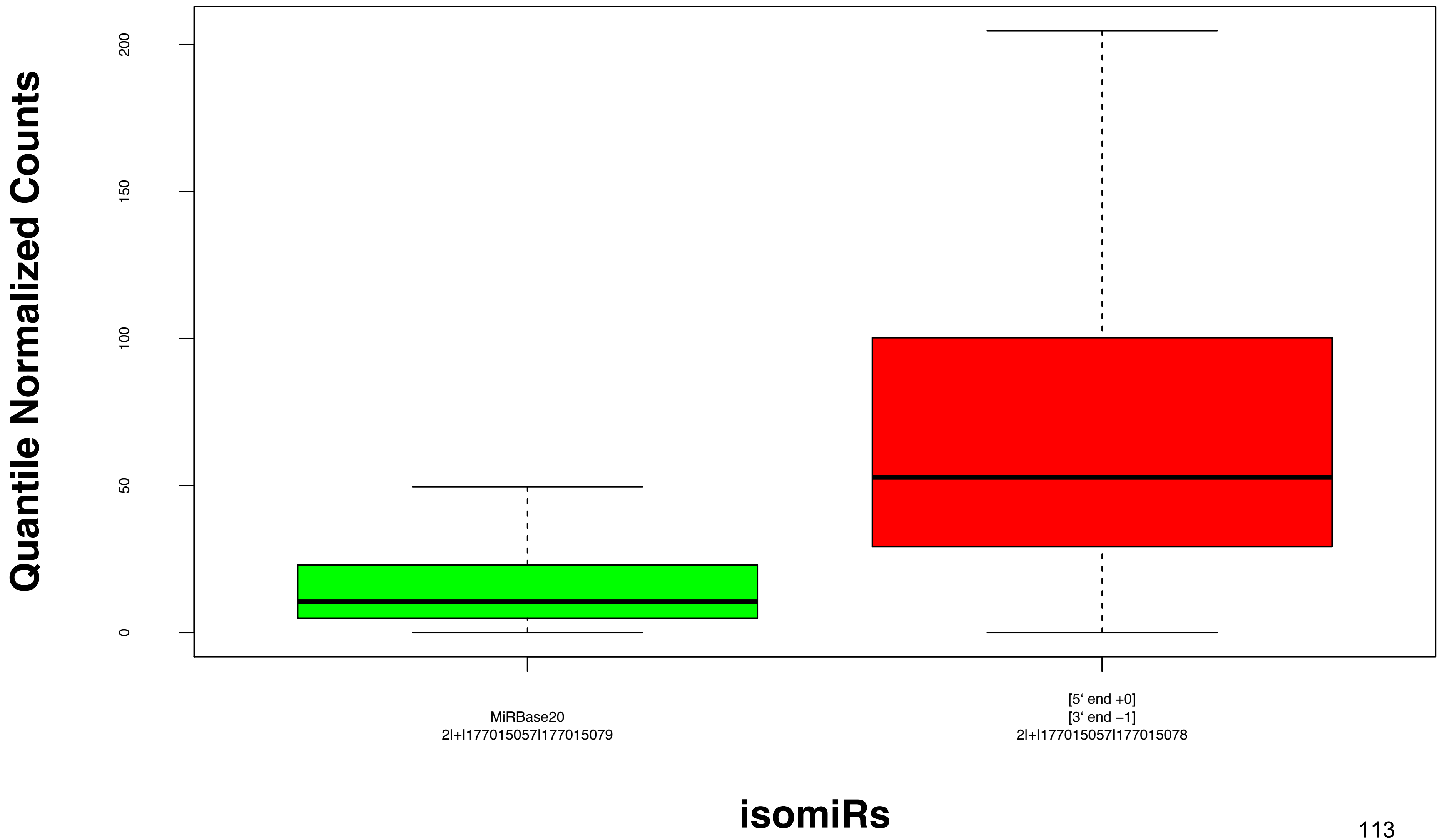

Supp. Figure 1
(pages 1 to 194)

MIMAT0004658=hsa−miR−155−3p=MI0000681

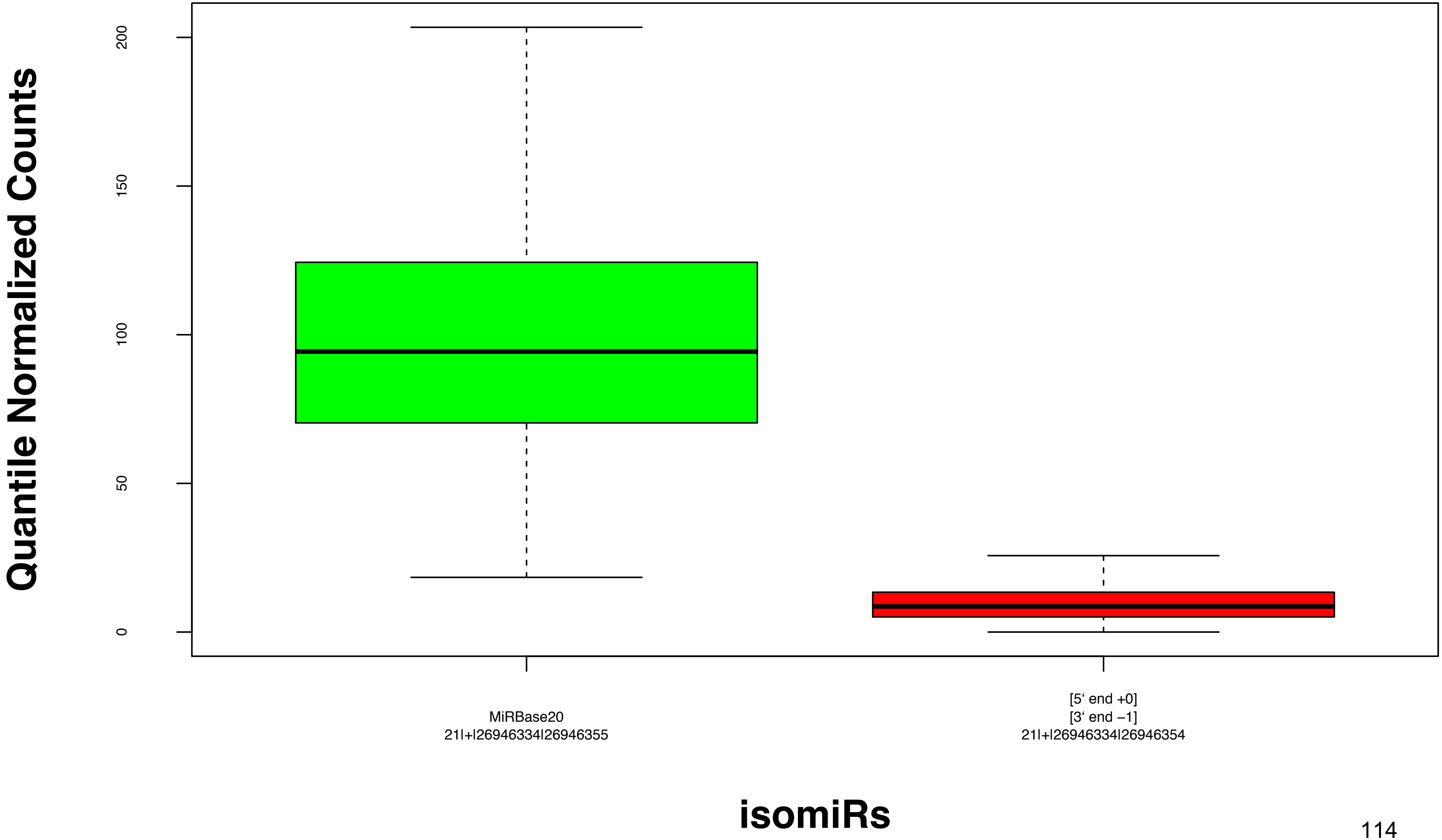

Supp. Figure 1
(pages 1 to 194)

MIMAT0026477=hsa−miR−128−1−5p=MI0000447

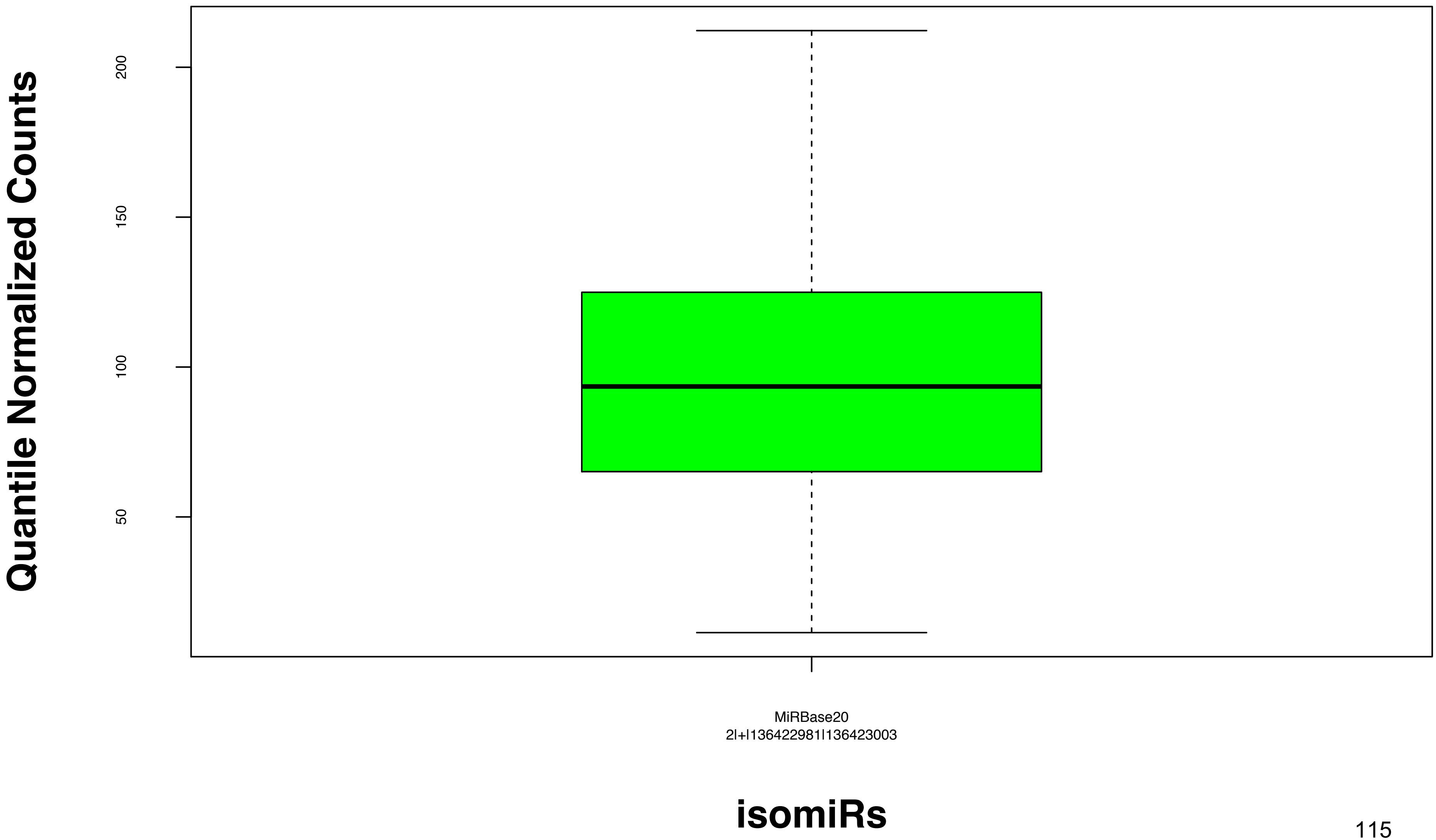

Supp. Figure 1
(pages 1 to 194)

MIMAT0000066=hsa–let–7e–5p=MI0000066

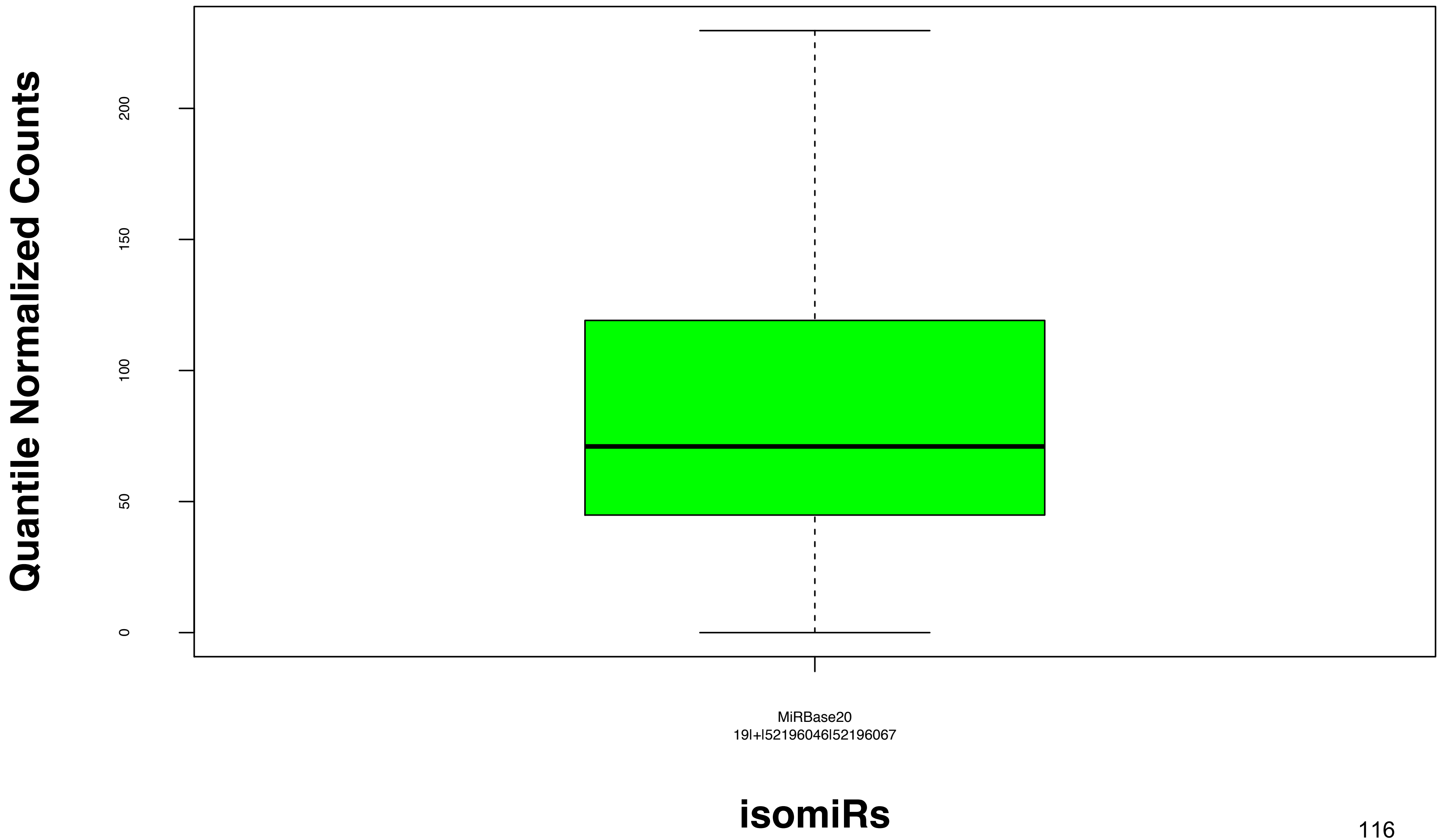

Supp. Figure 1
(pages 1 to 194)

MIMAT0000681=hsa−miR−29c−3p=MI0000735

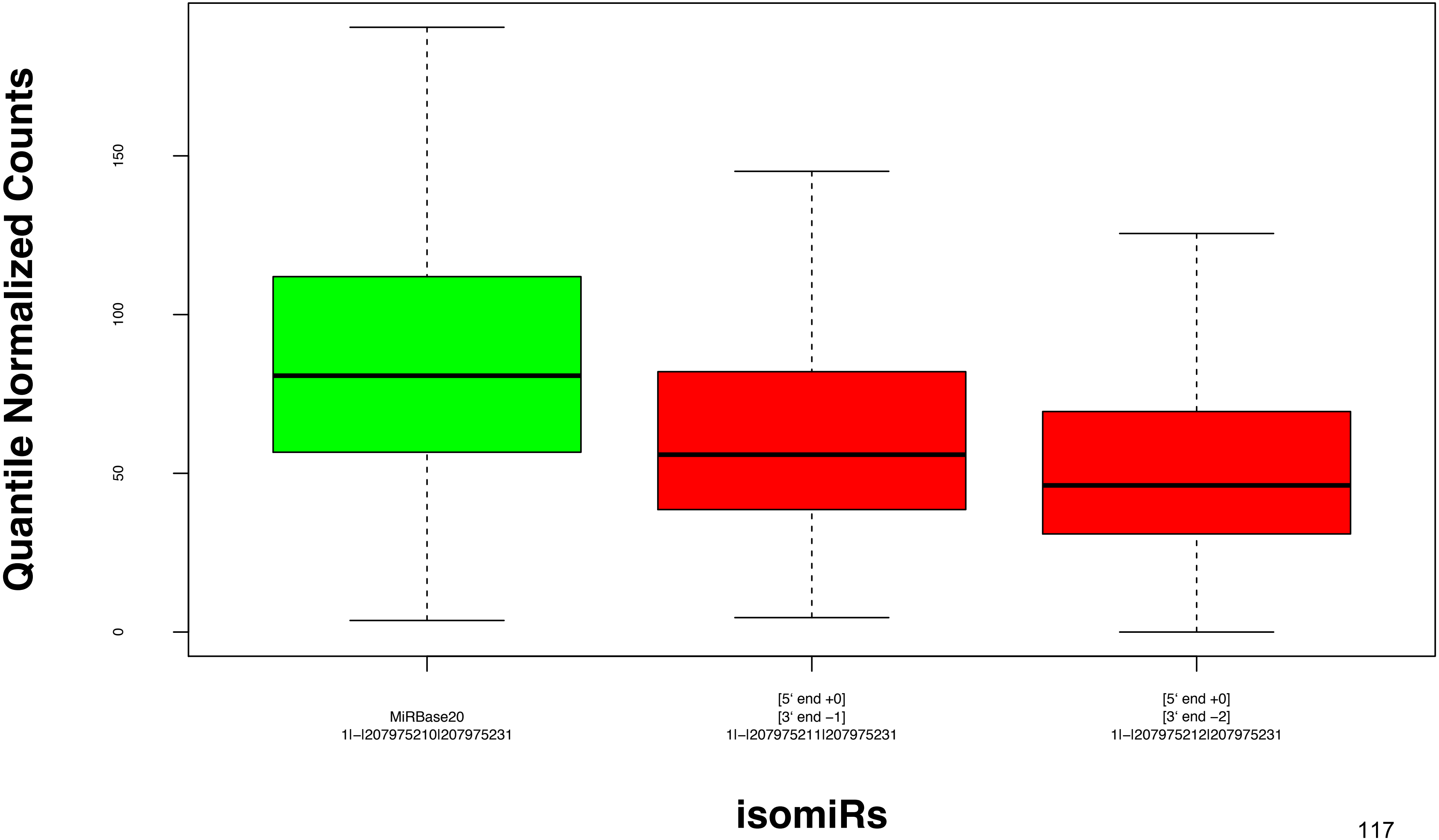

Supp. Figure 1
(pages 1 to 194)

MIMAT0000101=hsa−miR−103a−3p=MI0000108

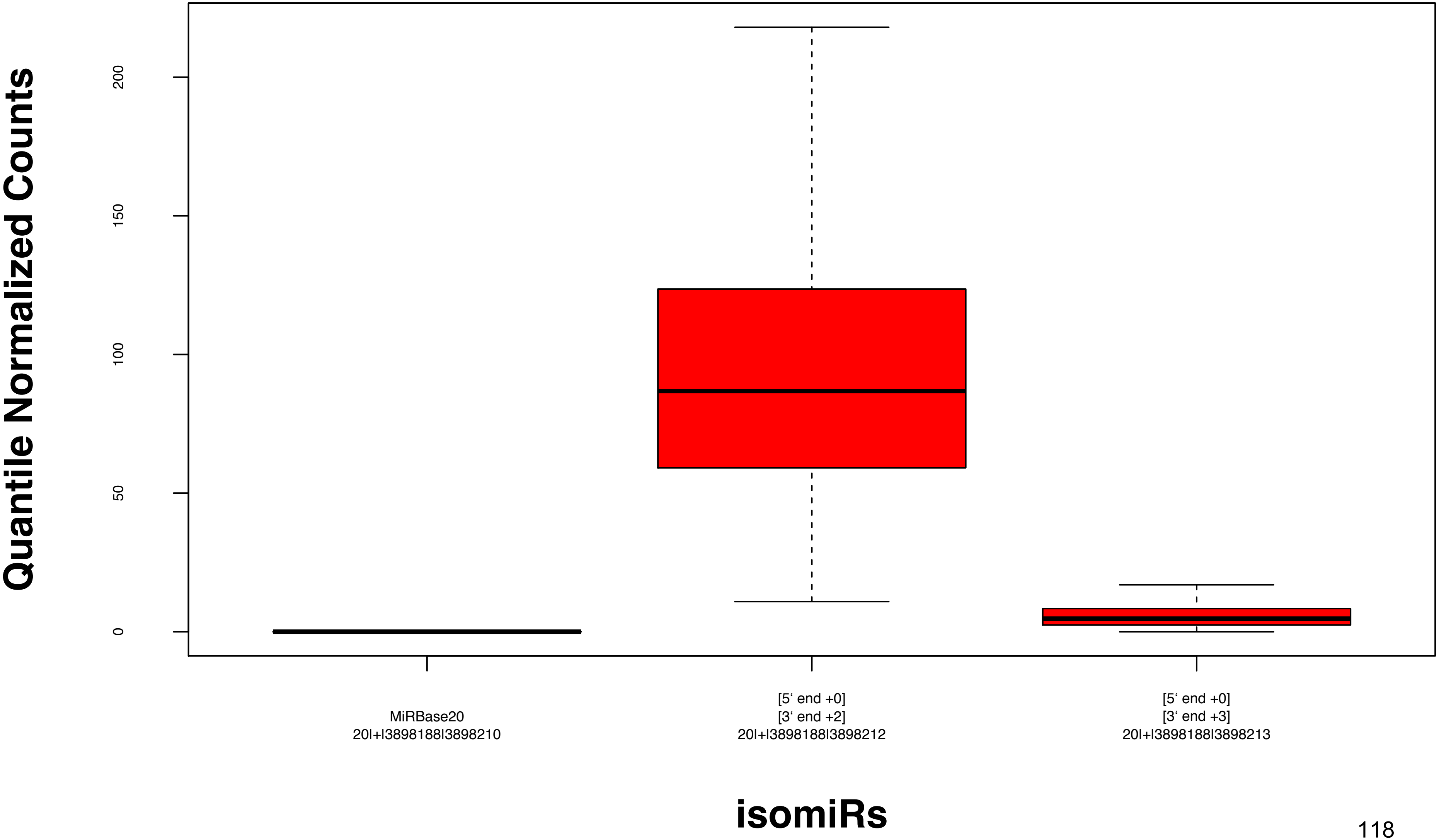

Supp. Figure 1
(pages 1 to 194)

MIMAT0000689=hsa−miR−99b−5p=MI0000746

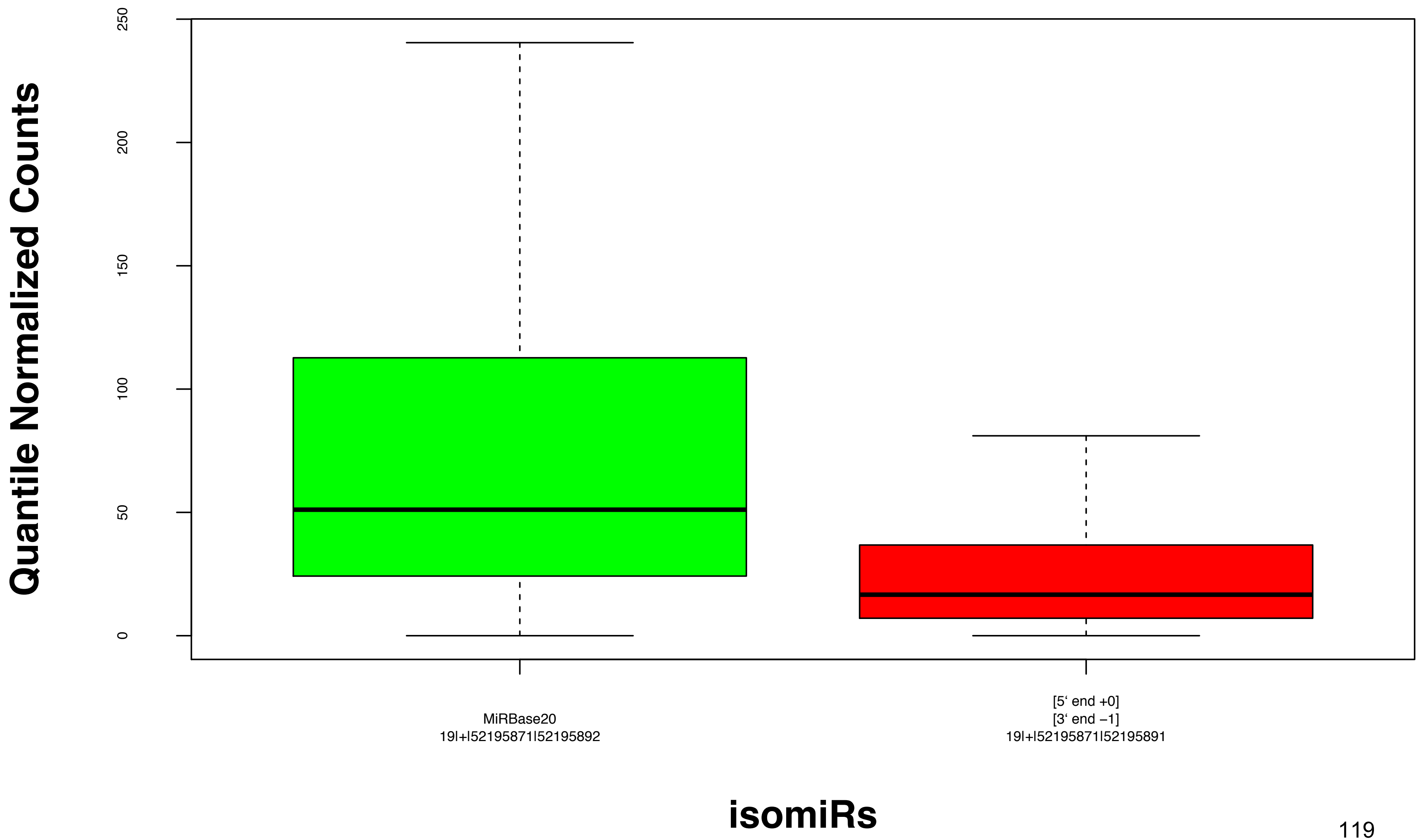

Supp. Figure 1
(pages 1 to 194)

MIMAT0004766=hsa−miR−146b−3p=MI0003129

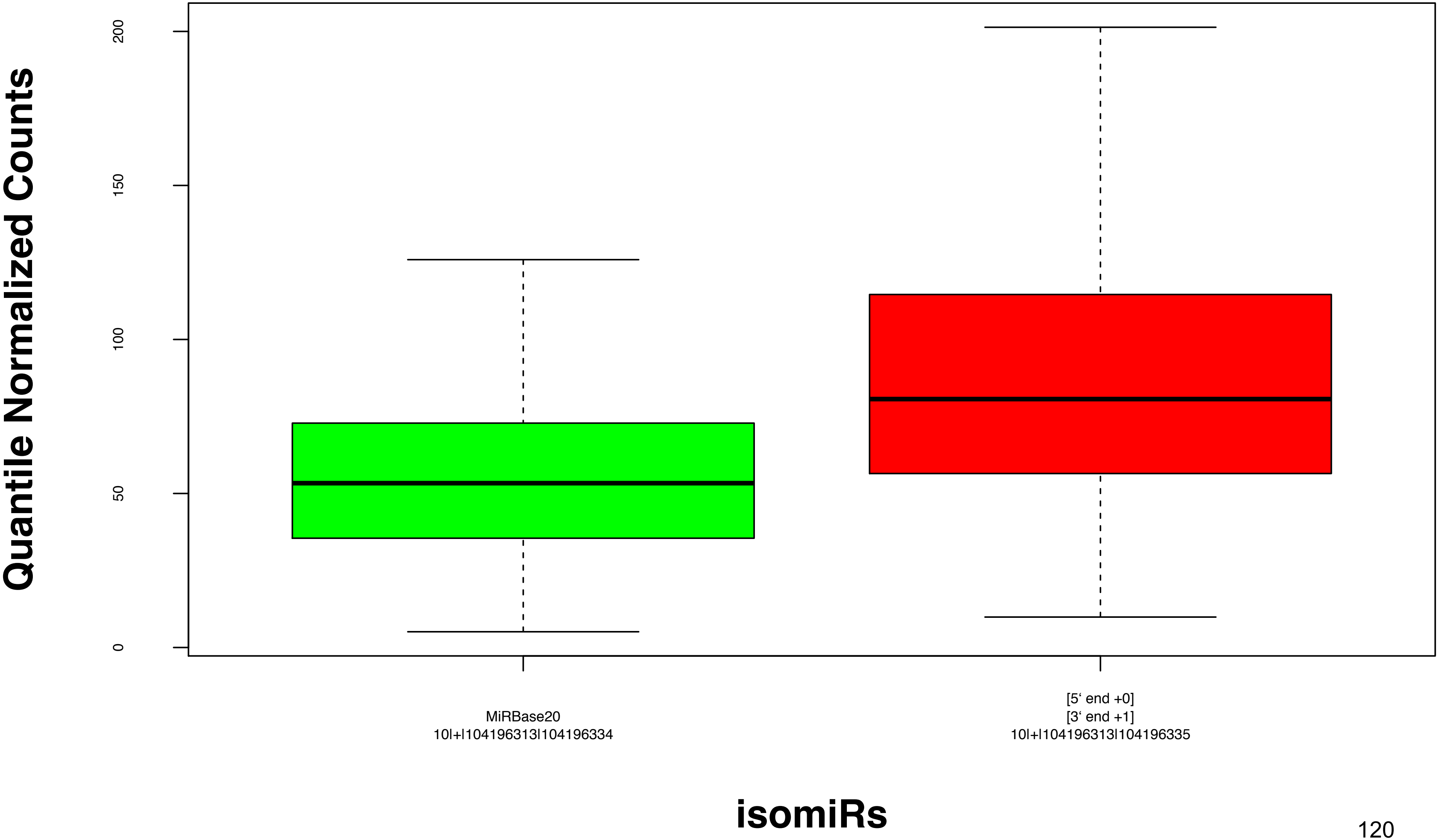

Supp. Figure 1
(pages 1 to 194)

MIMAT0004586=hsa−miR−15b−3p=MI0000438

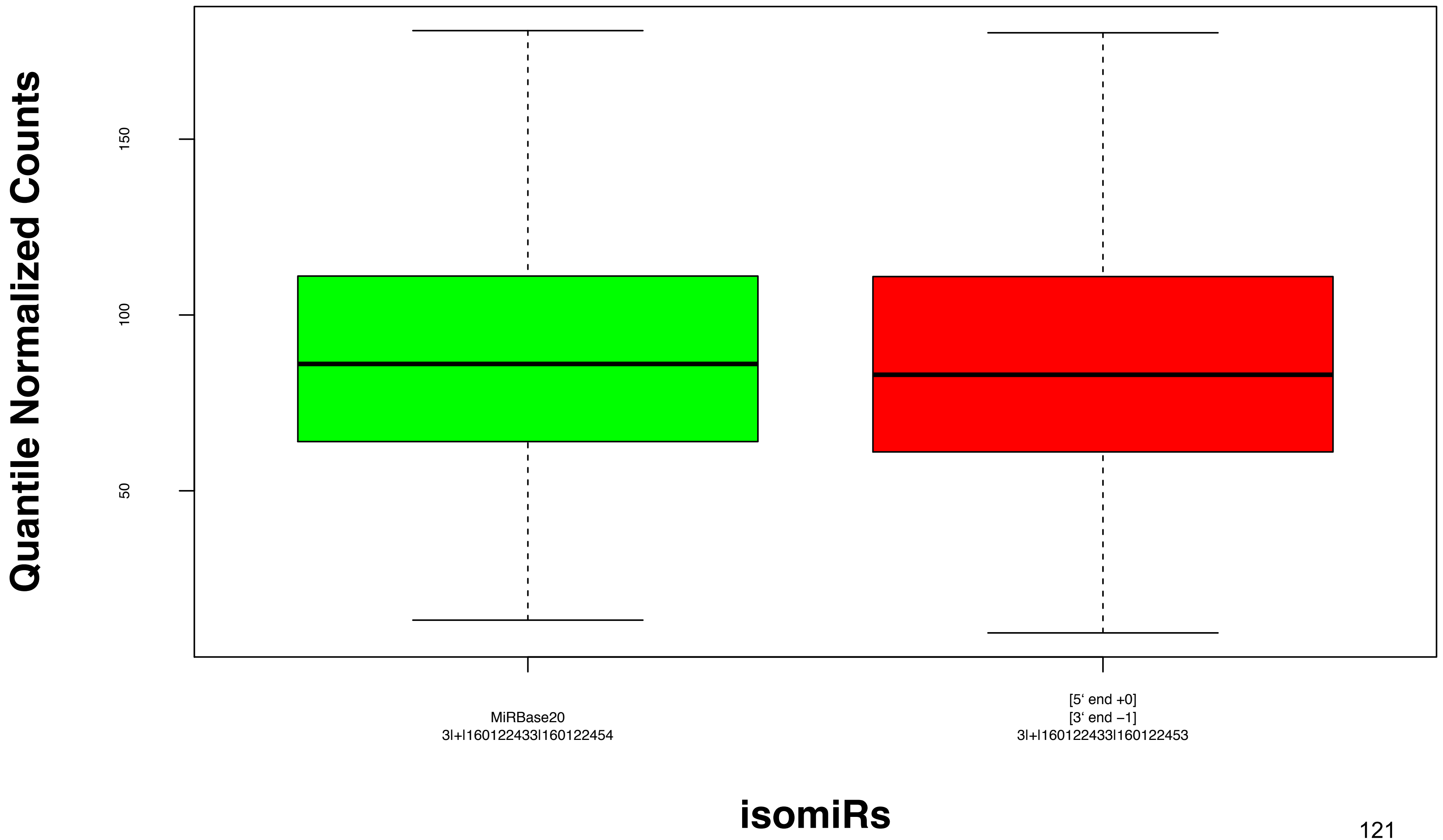

Supp. Figure 1
(pages 1 to 194)

MIMAT0004693=hsa−miR−330−5p=MI0000803

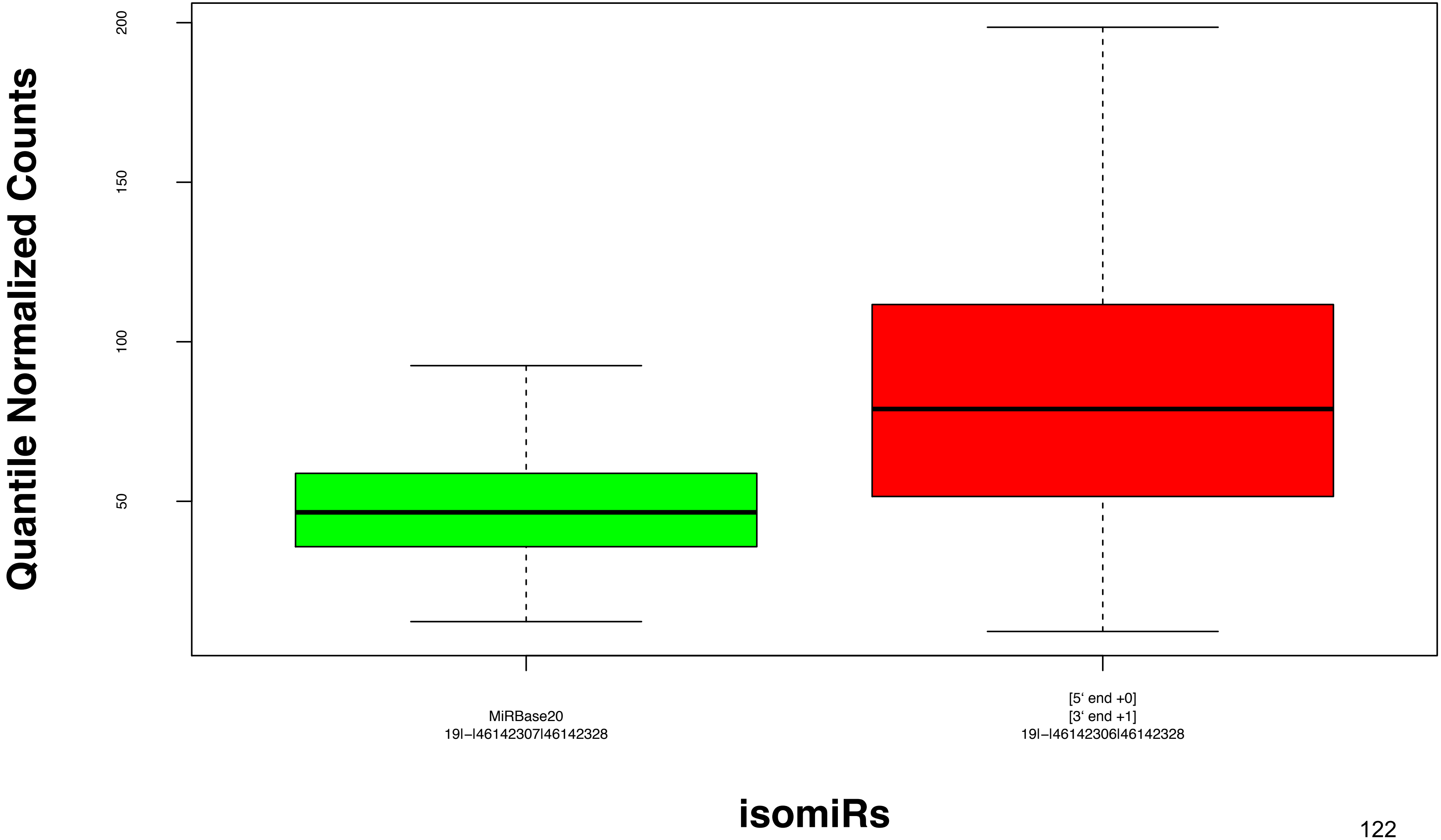

Supp. Figure 1
(pages 1 to 194)

MIMAT0000062=hsa–let–7a–5p=MI0000061

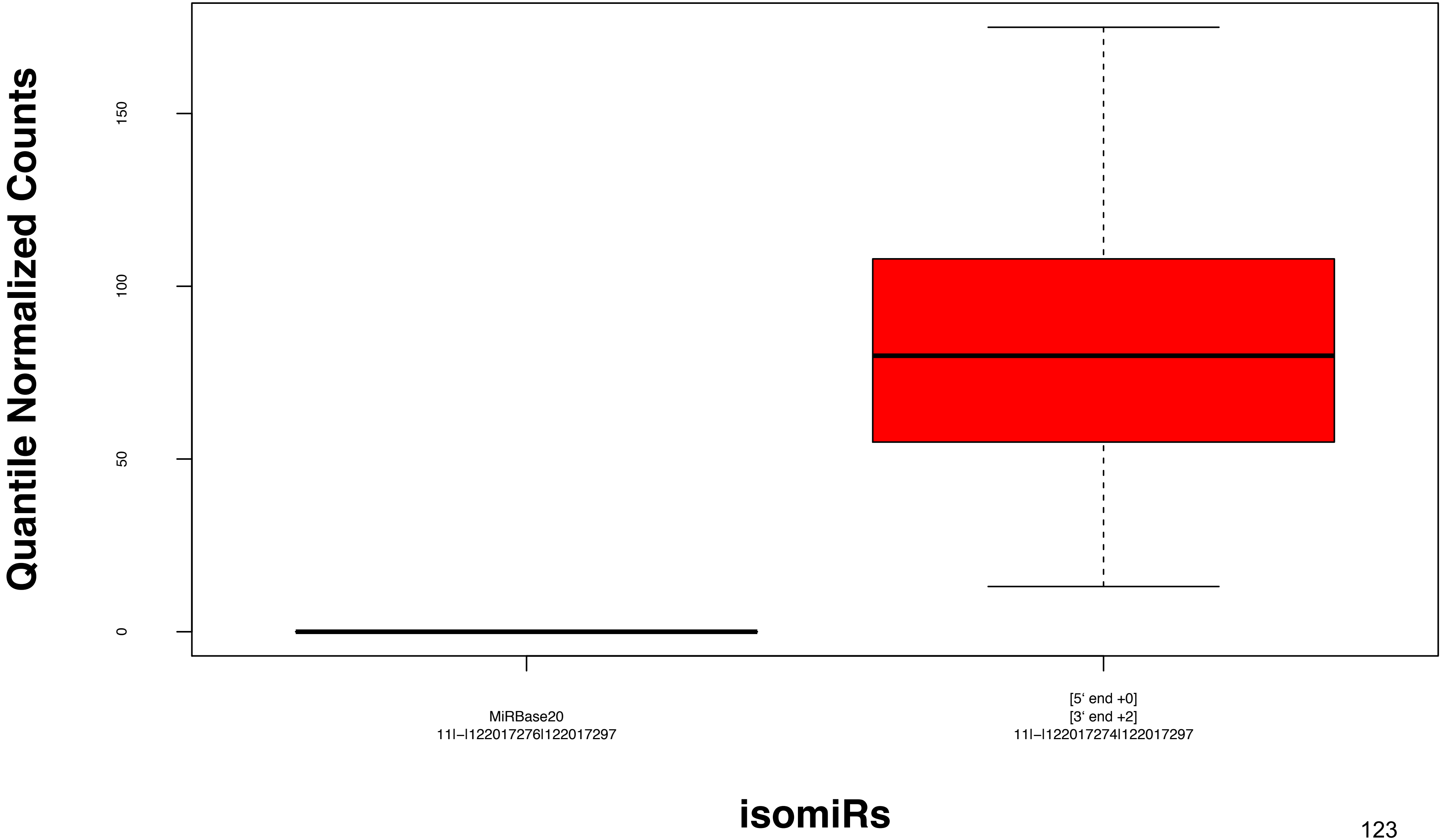

Supp. Figure 1
(pages 1 to 194)

MIMAT0000103=hsa−miR−106a−5p=MI0000113

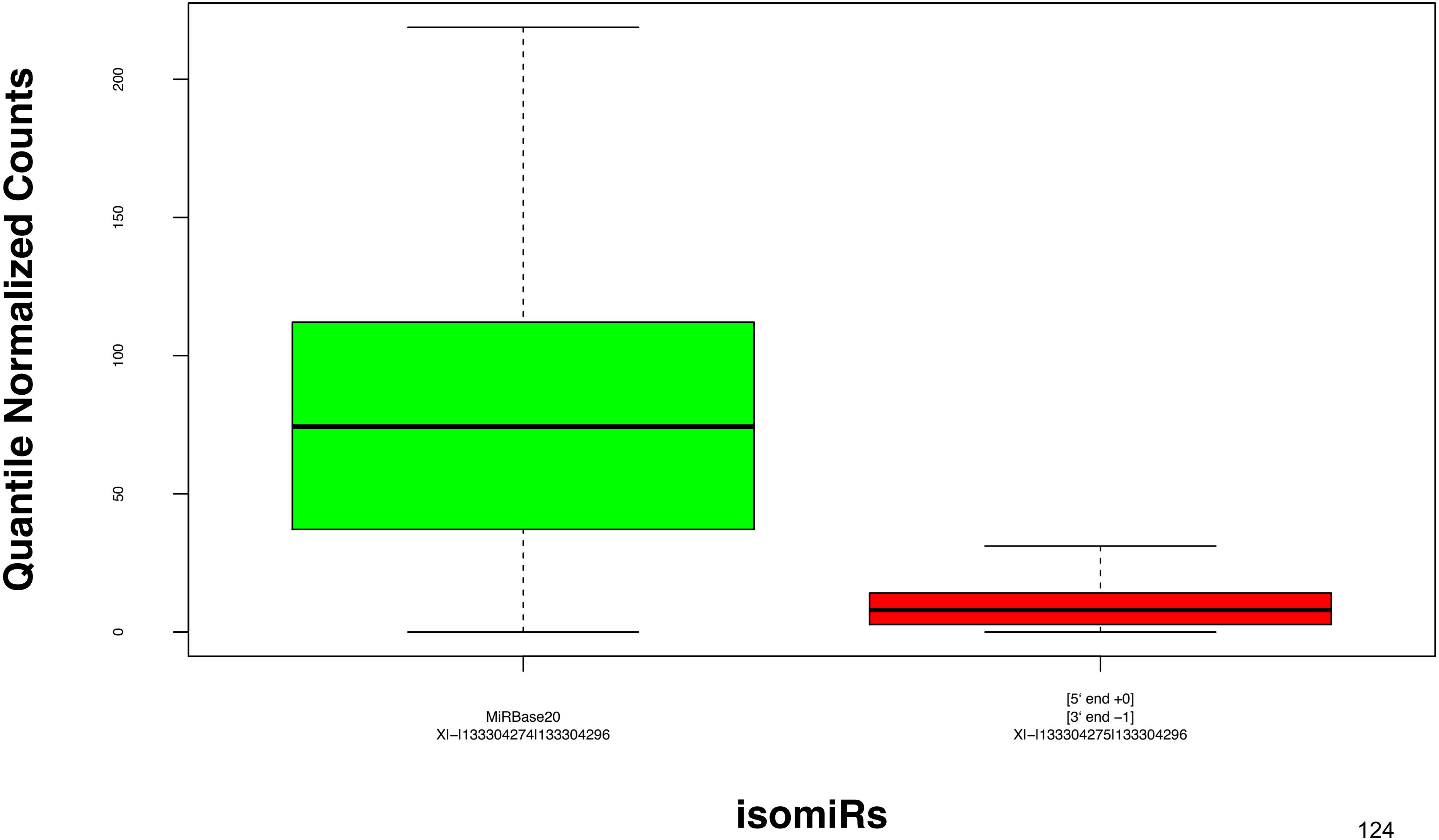

Supp. Figure 1
(pages 1 to 194)

MIMAT0000764=hsa−miR−339−5p=MI0000815

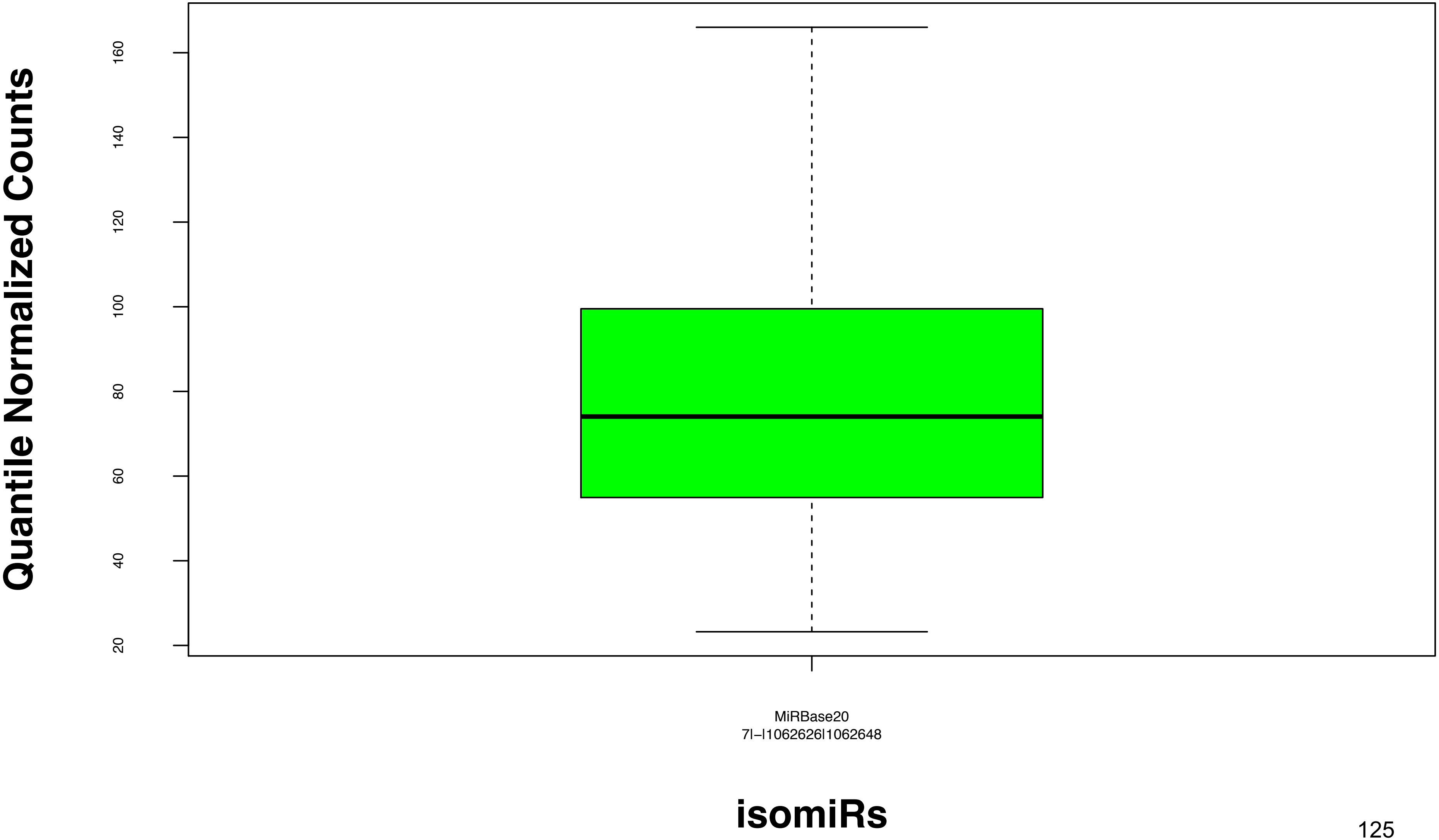

Supp. Figure 1
(pages 1 to 194)

MIMAT0001343=hsa−miR−425−3p=MI0001448

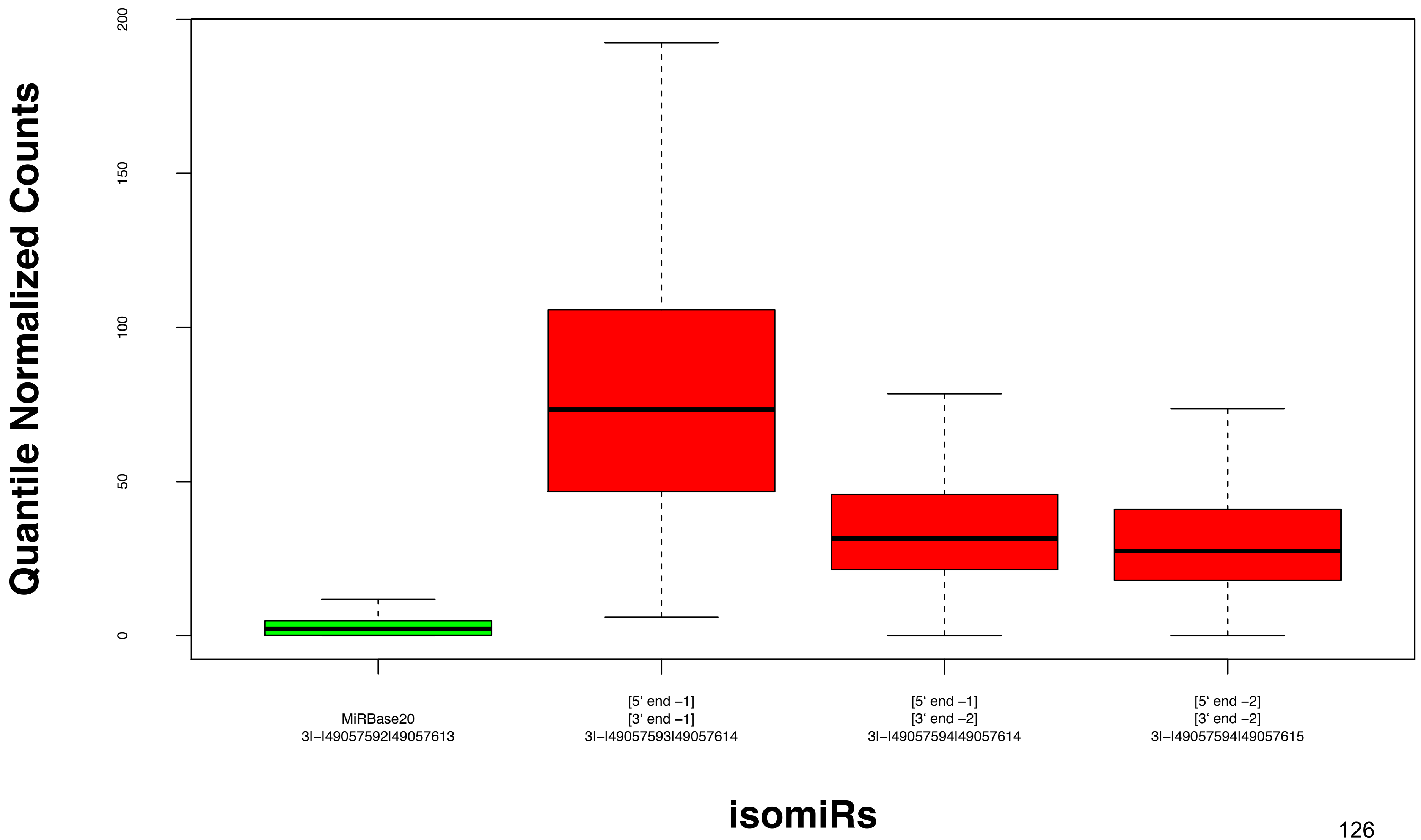

Supp. Figure 1
(pages 1 to 194)

MIMAT0018108=hsa–miR–3681–5p=MI0016082

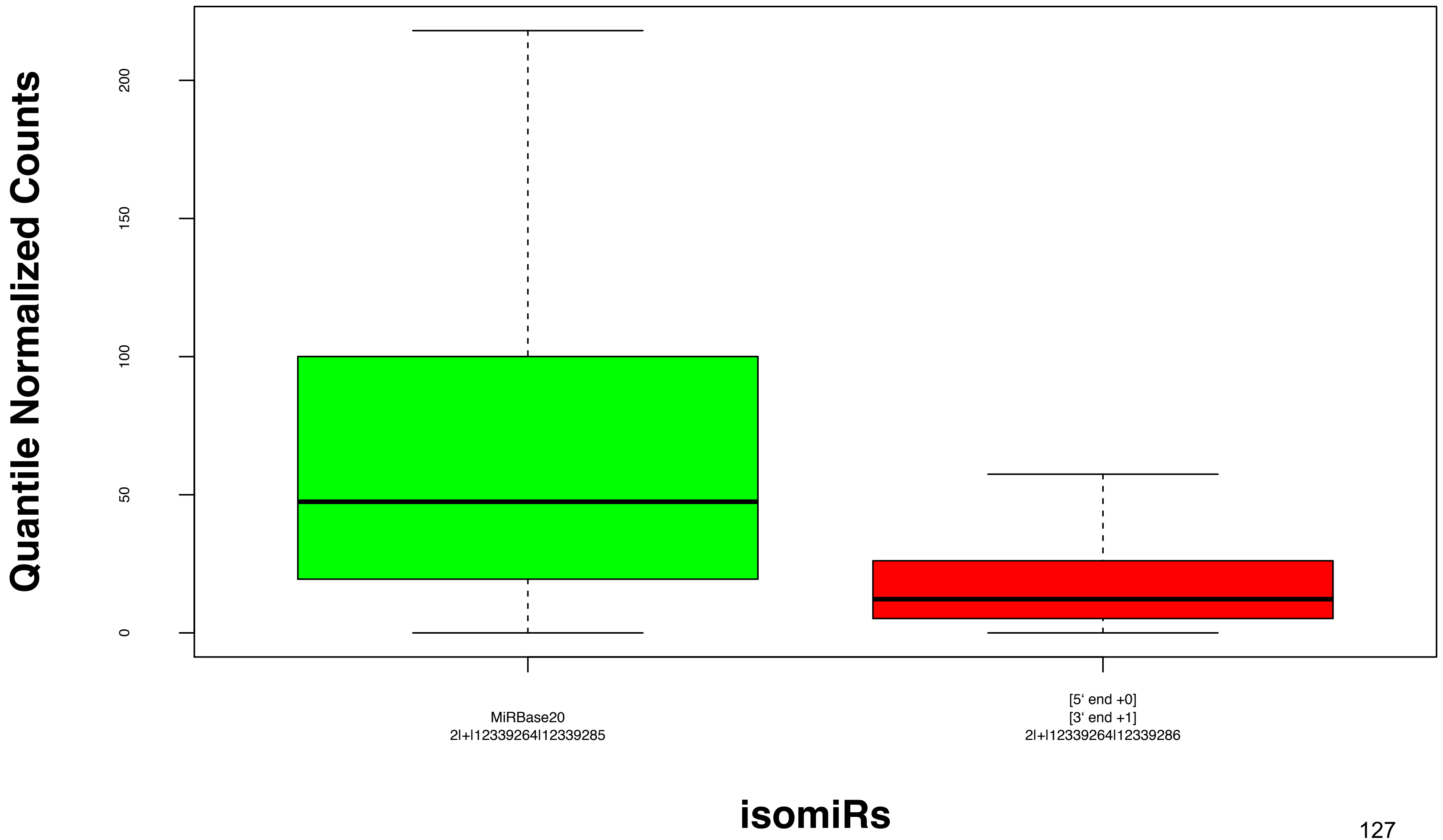

Supp. Figure 1
(pages 1 to 194)

MIMAT0004688=hsa−miR−374a−3p=MI0000782

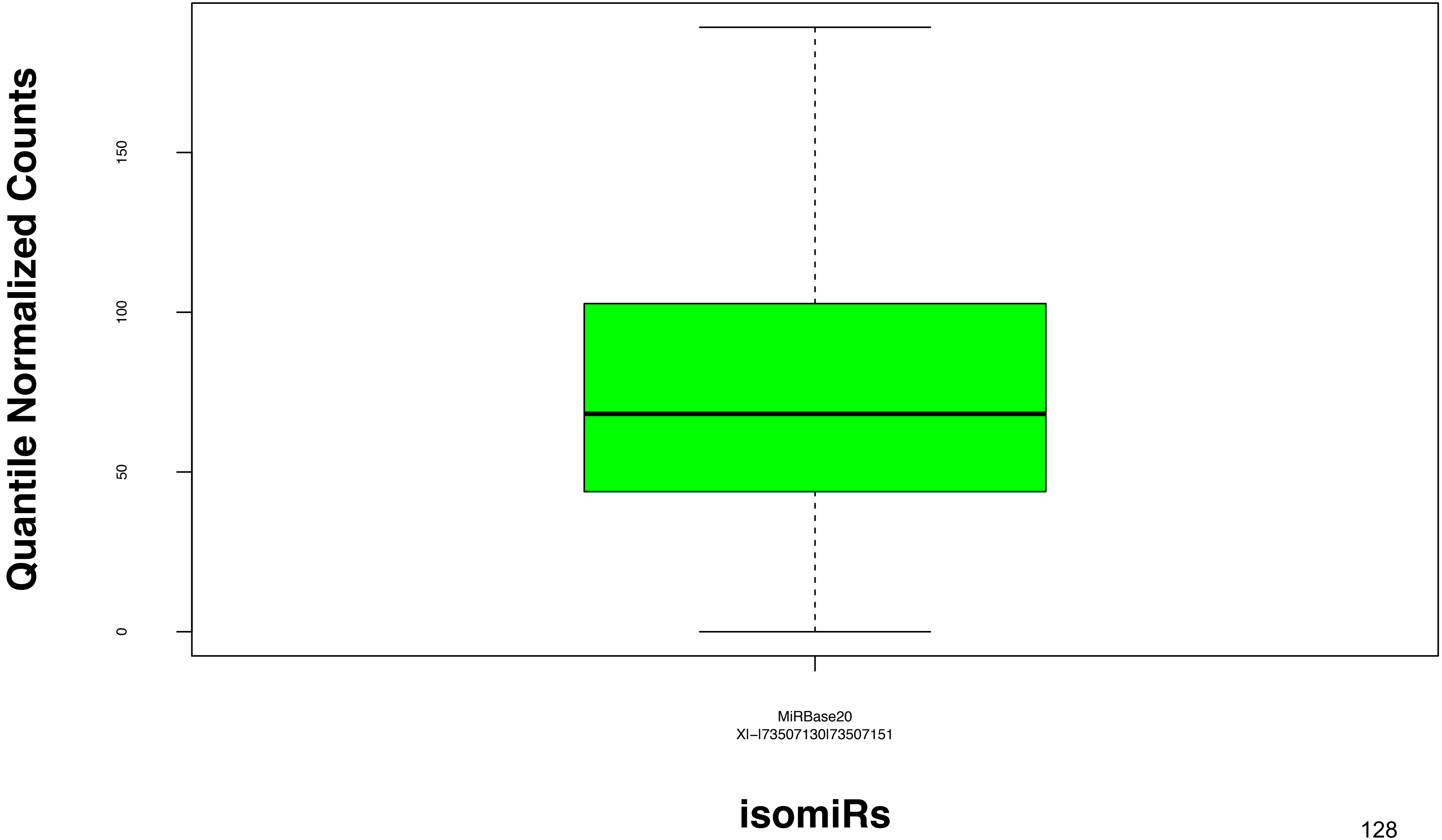

Supp. Figure 1
(pages 1 to 194)

MIMAT0017994=hsa−miR−3615=MI0016005

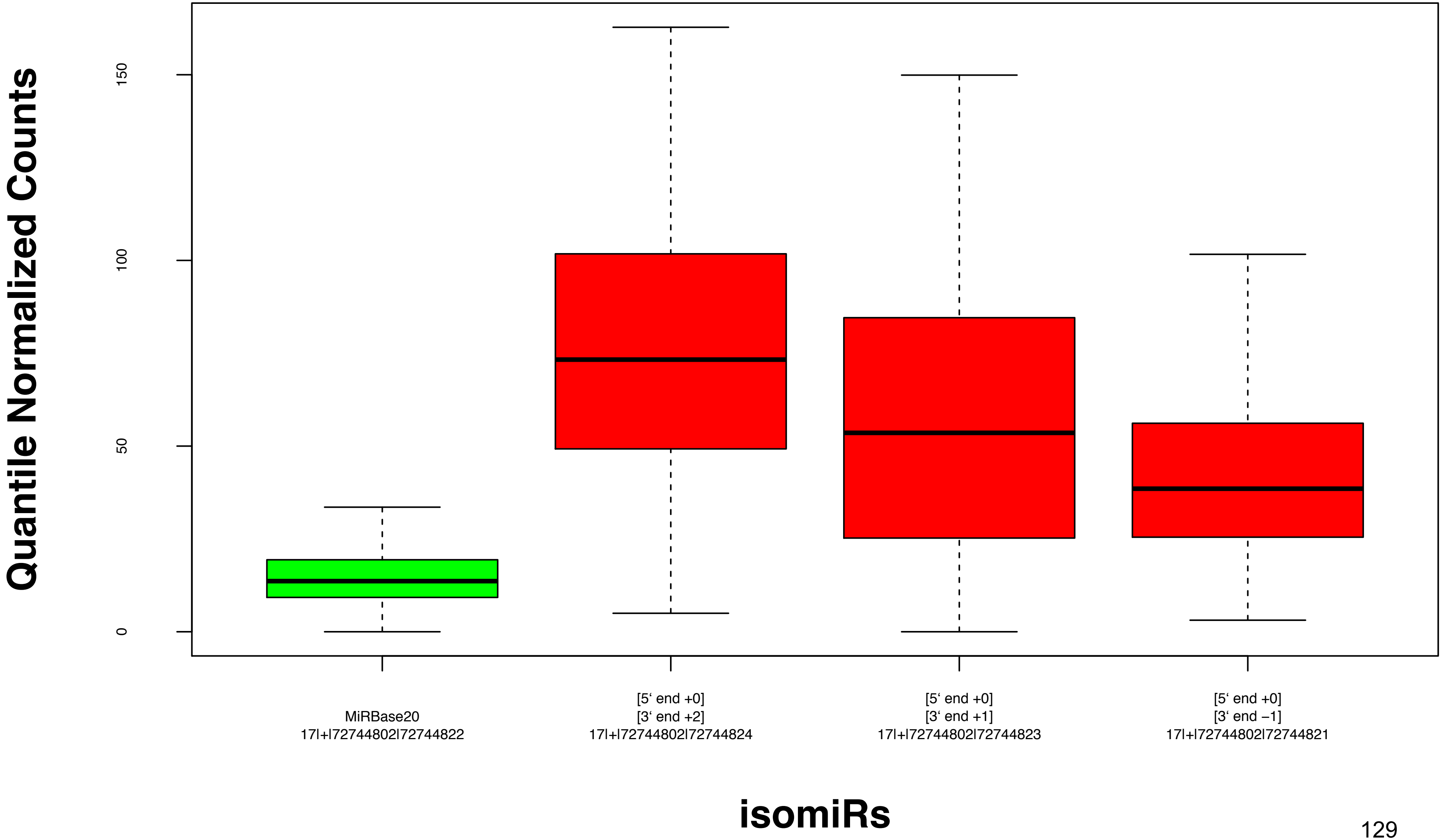

Supp. Figure 1
(pages 1 to 194)

MIMAT0002821=hsa–miR–181d–5p=MI0003139

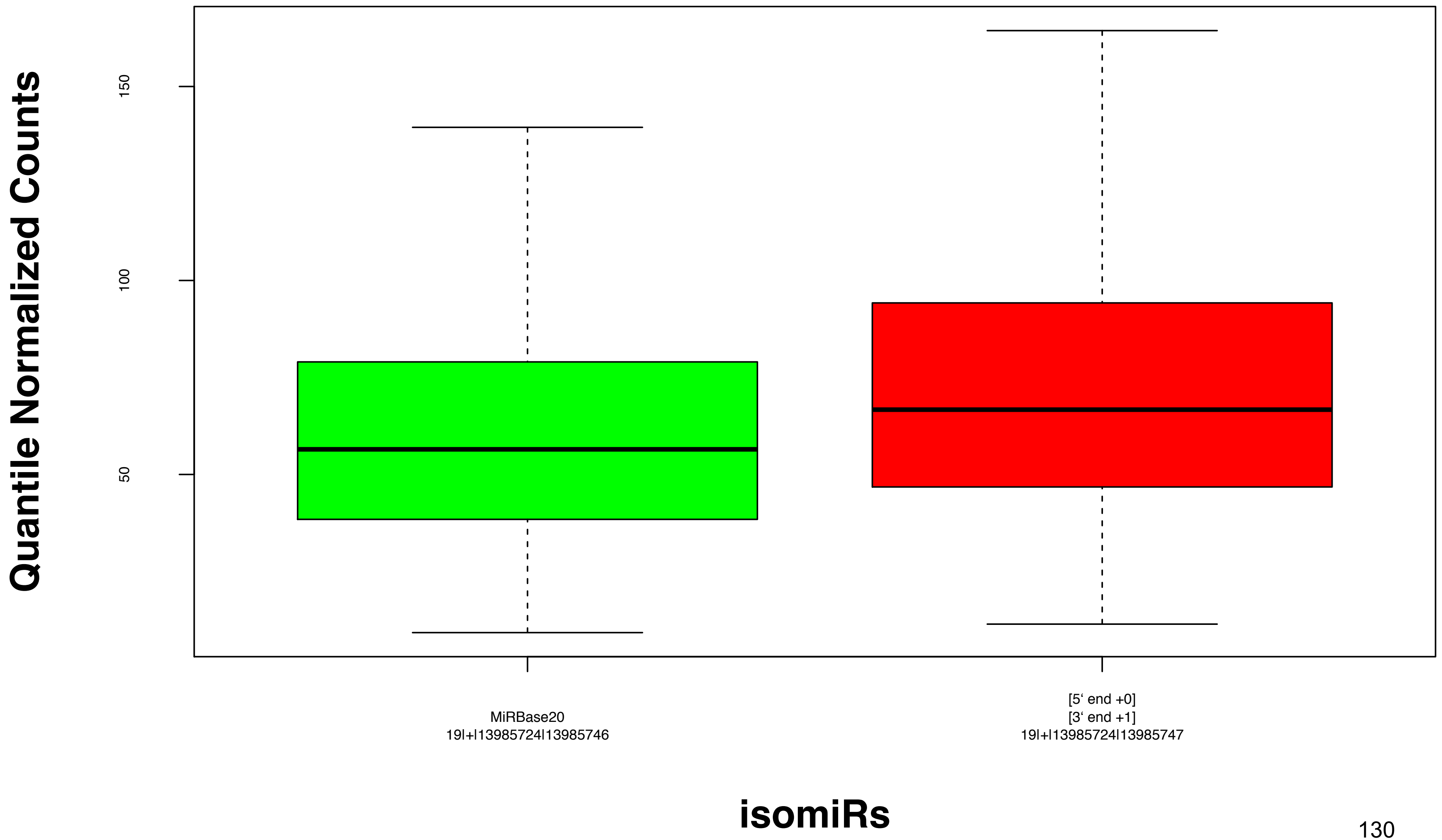

Supp. Figure 1
(pages 1 to 194)

MIMAT0004674=hsa−miR−30c−1−3p=MI0000736

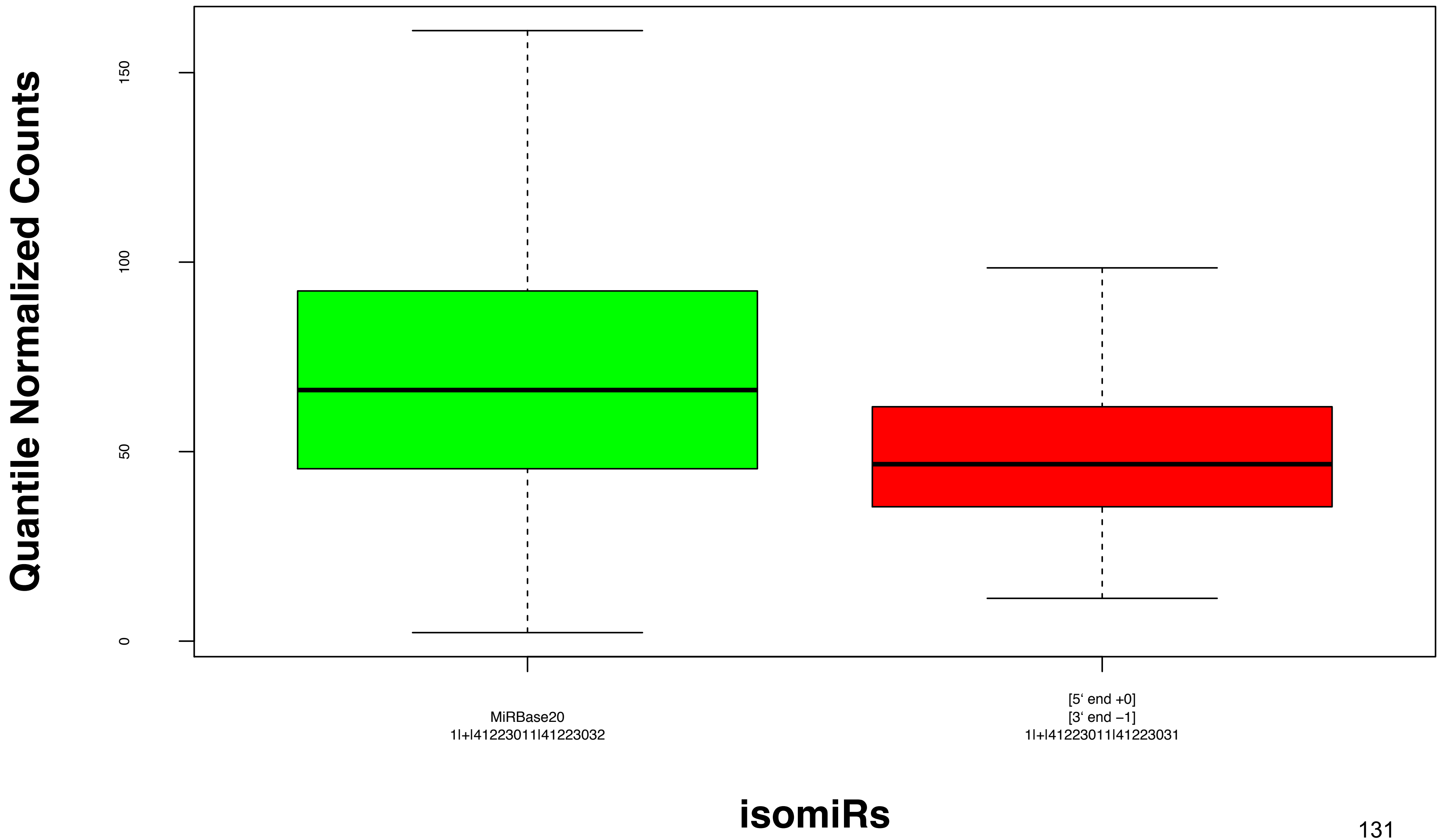

Supp. Figure 1
(pages 1 to 194)

MIMAT0002177=hsa−miR−486−5p=MI0002470

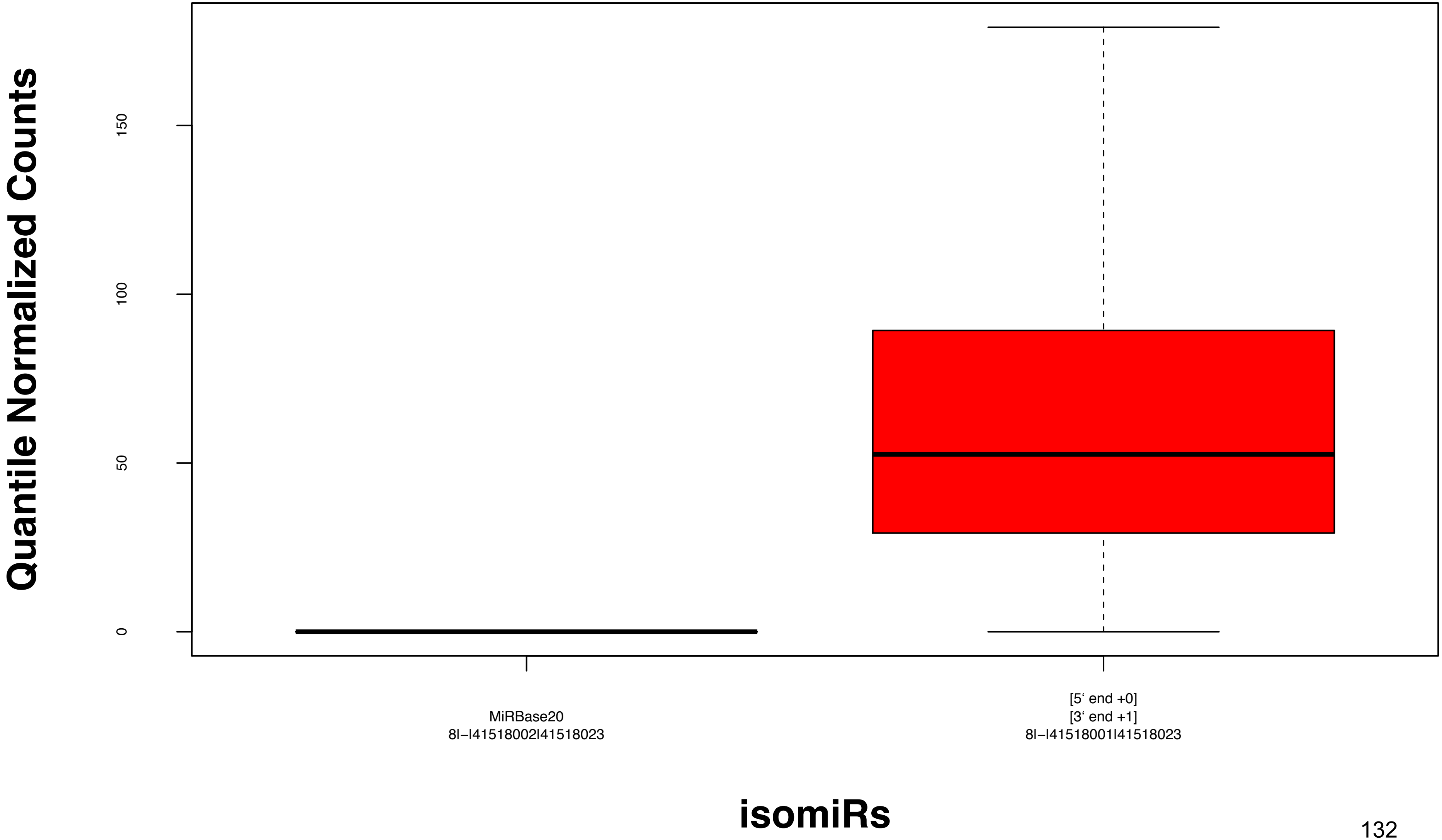

Supp. Figure 1
(pages 1 to 194)

MIMAT0003322=hsa−miR−652−3p=MI0003667

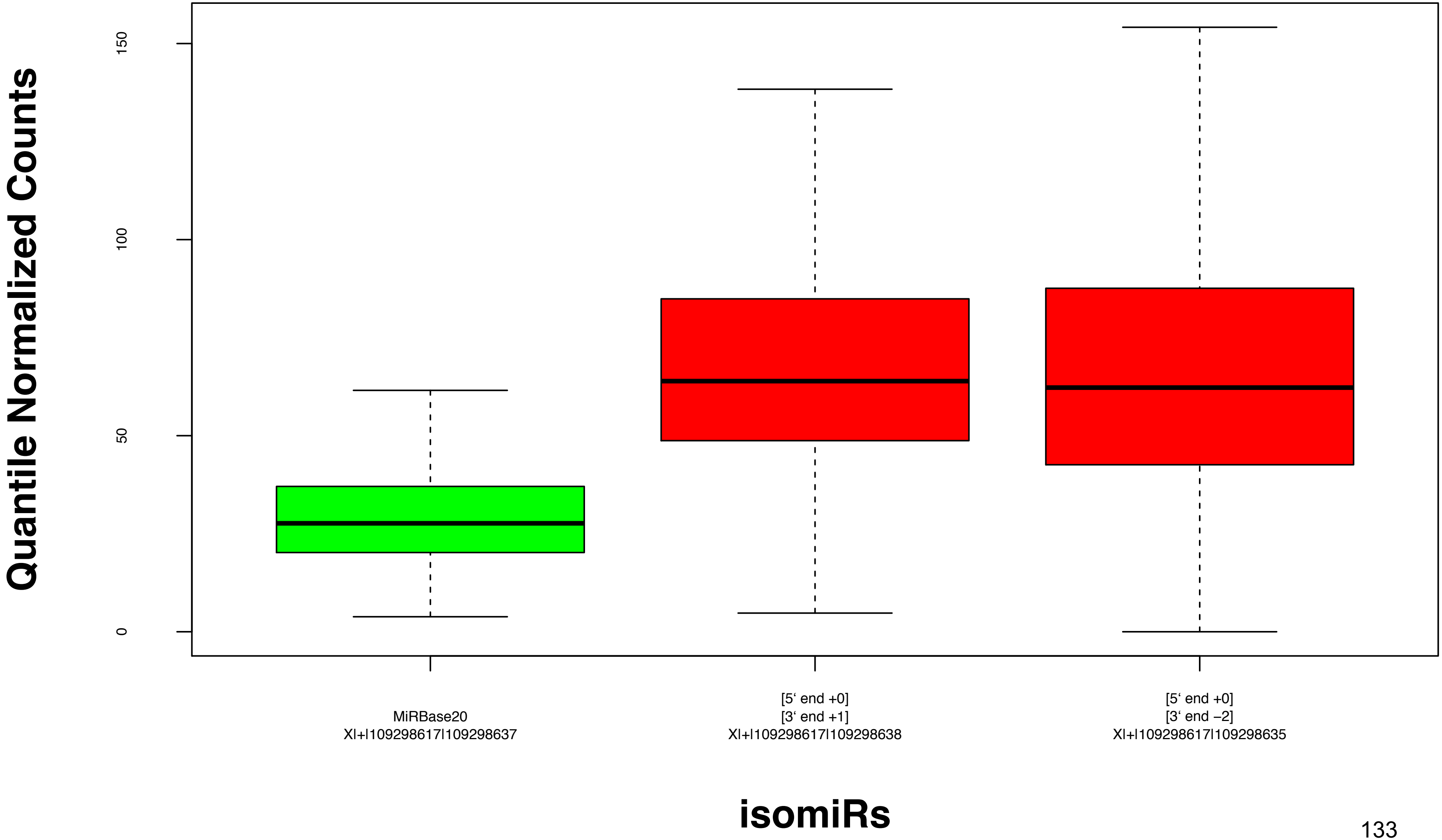

Supp. Figure 1
(pages 1 to 194)

MIMAT0000072=hsa−miR−18a−5p=MI0000072

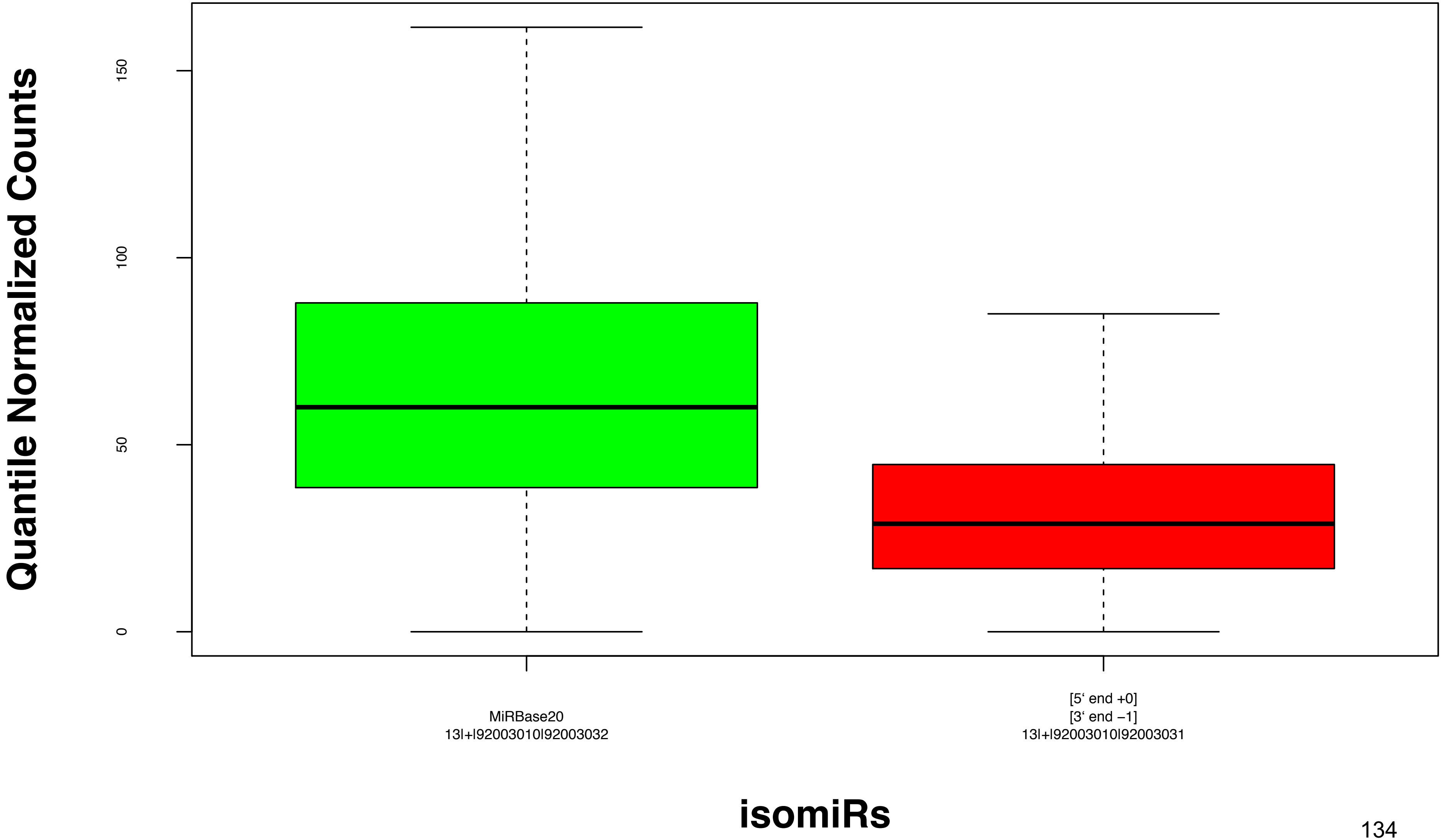

Supp. Figure 1
(pages 1 to 194)

MIMAT0004585=hsa–let–7i–3p=MI0000434

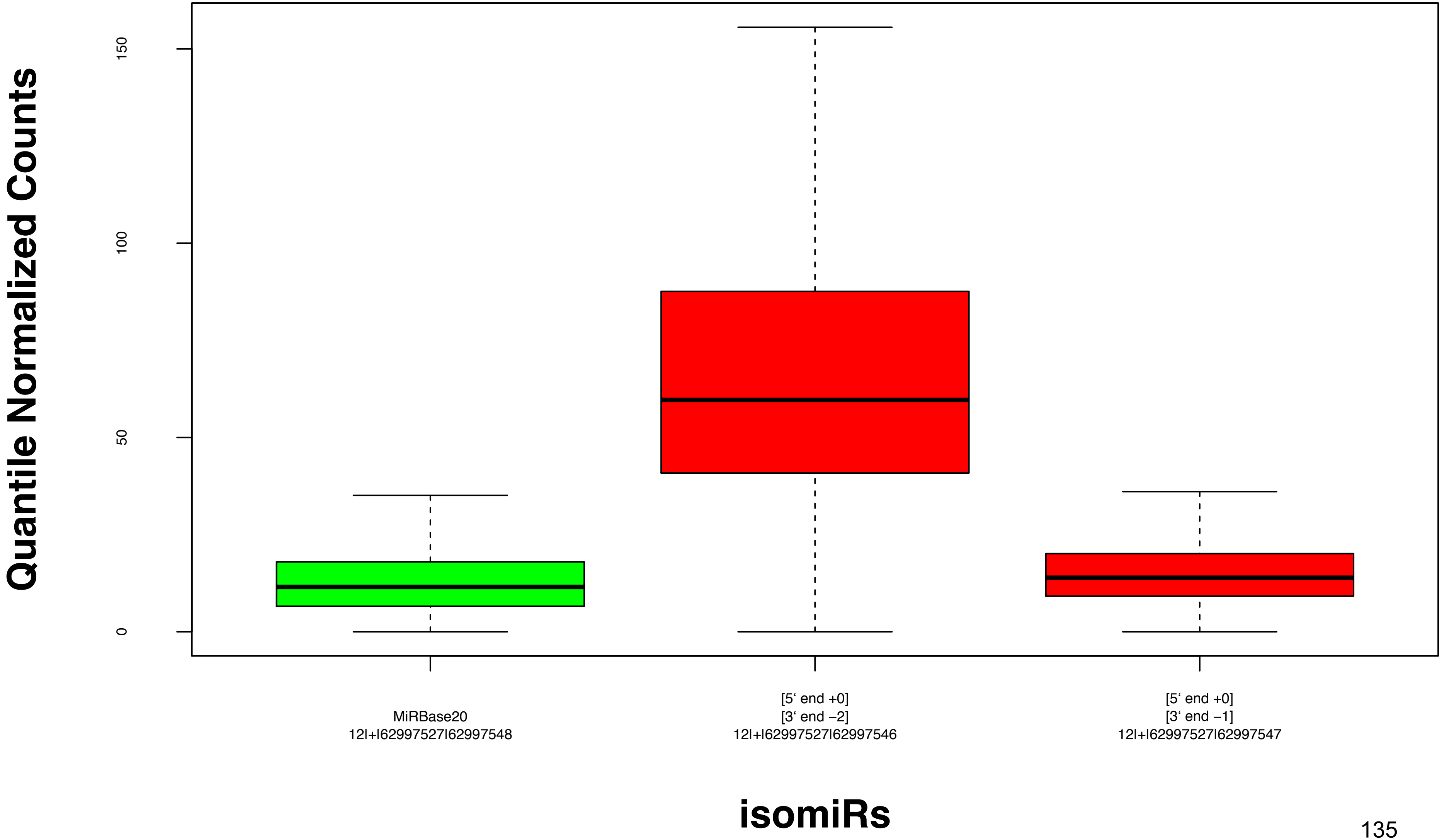

Supp. Figure 1
(pages 1 to 194)

MIMAT0000418=hsa−miR−23b−3p=MI0000439

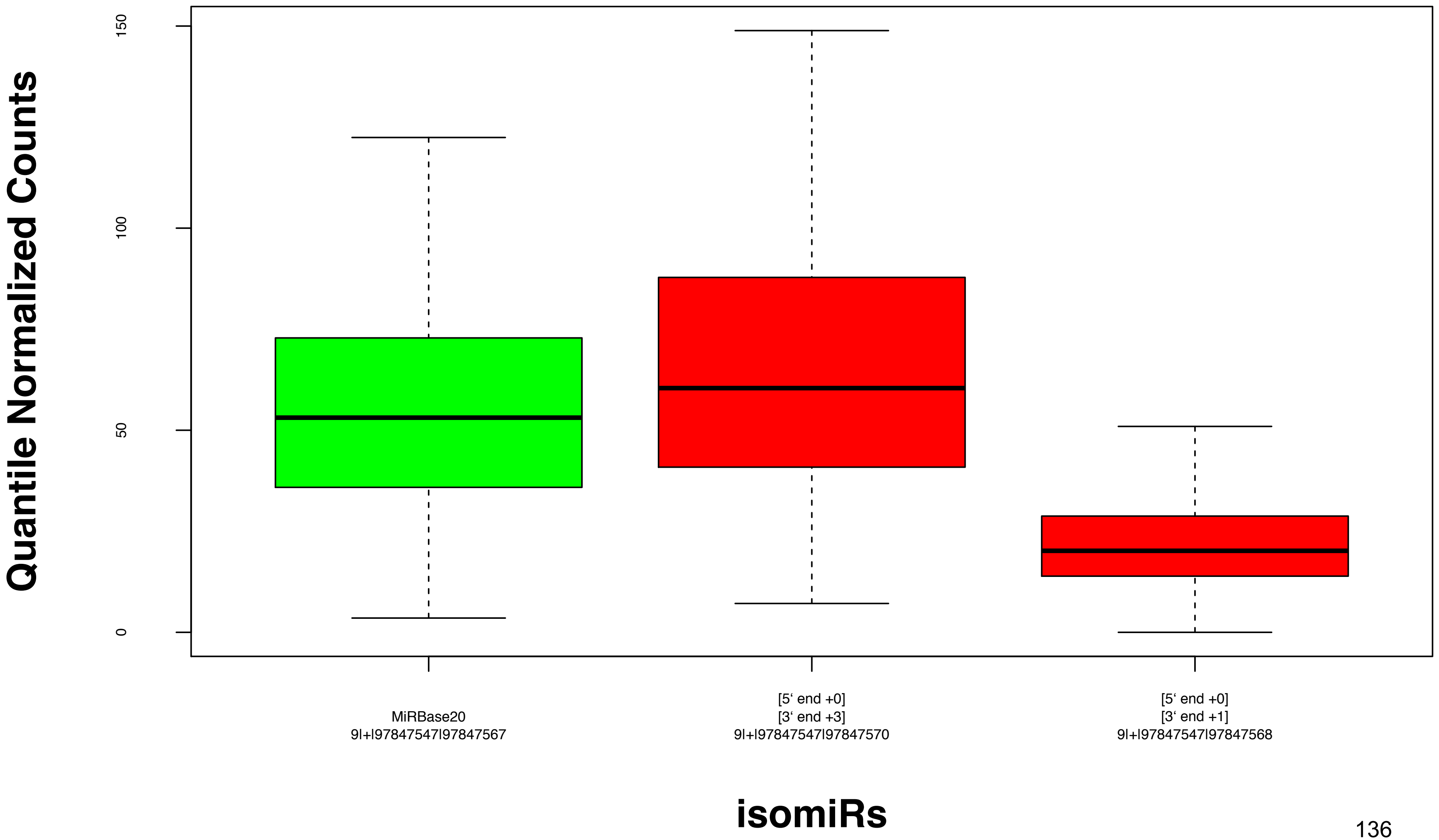

Supp. Figure 1
(pages 1 to 194)

MIMAT0031180=hsa–miR–7977=MI0025753

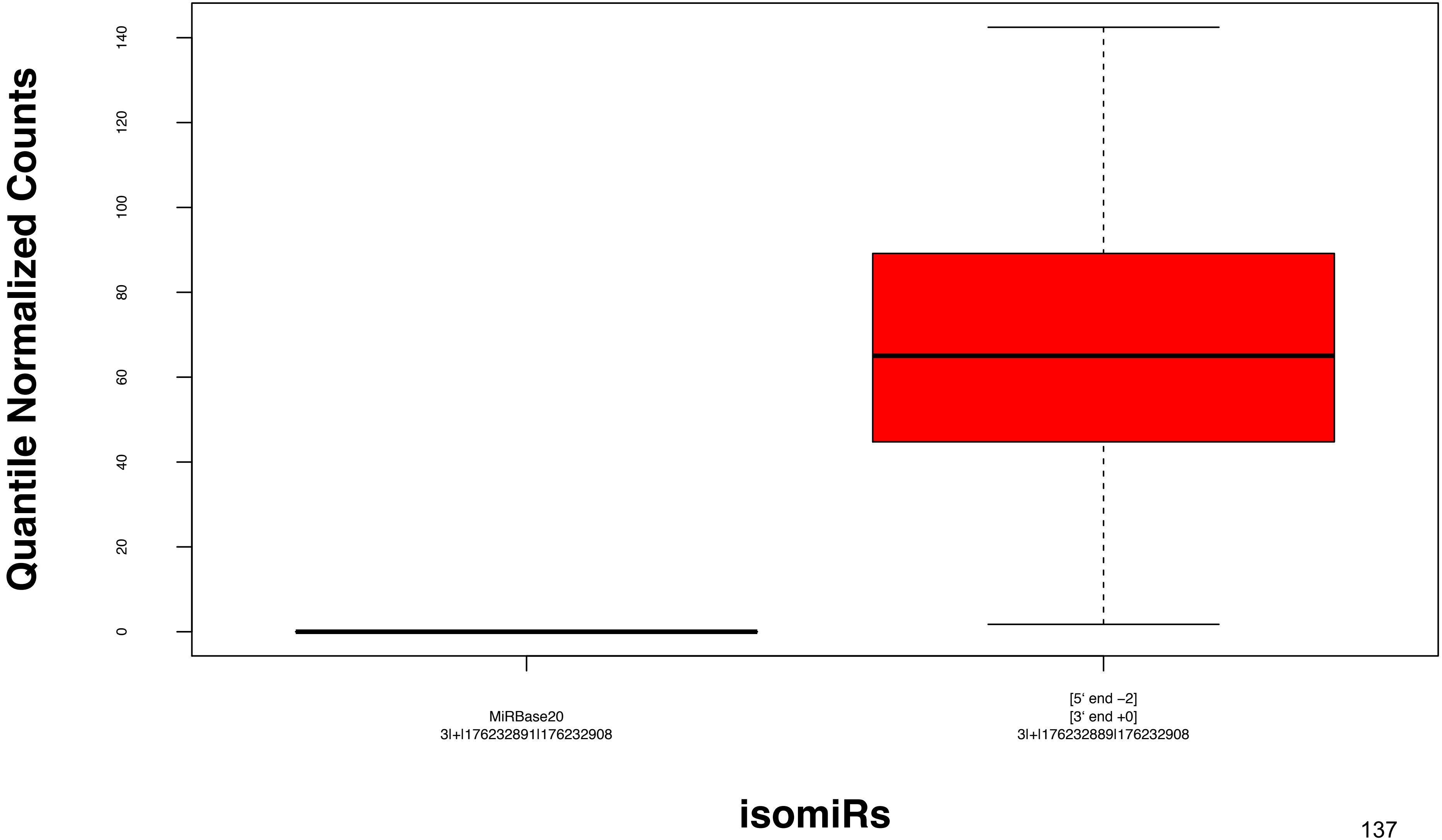

Supp. Figure 1
(pages 1 to 194)

MIMAT0004498=hsa–miR–25–5p=MI0000082

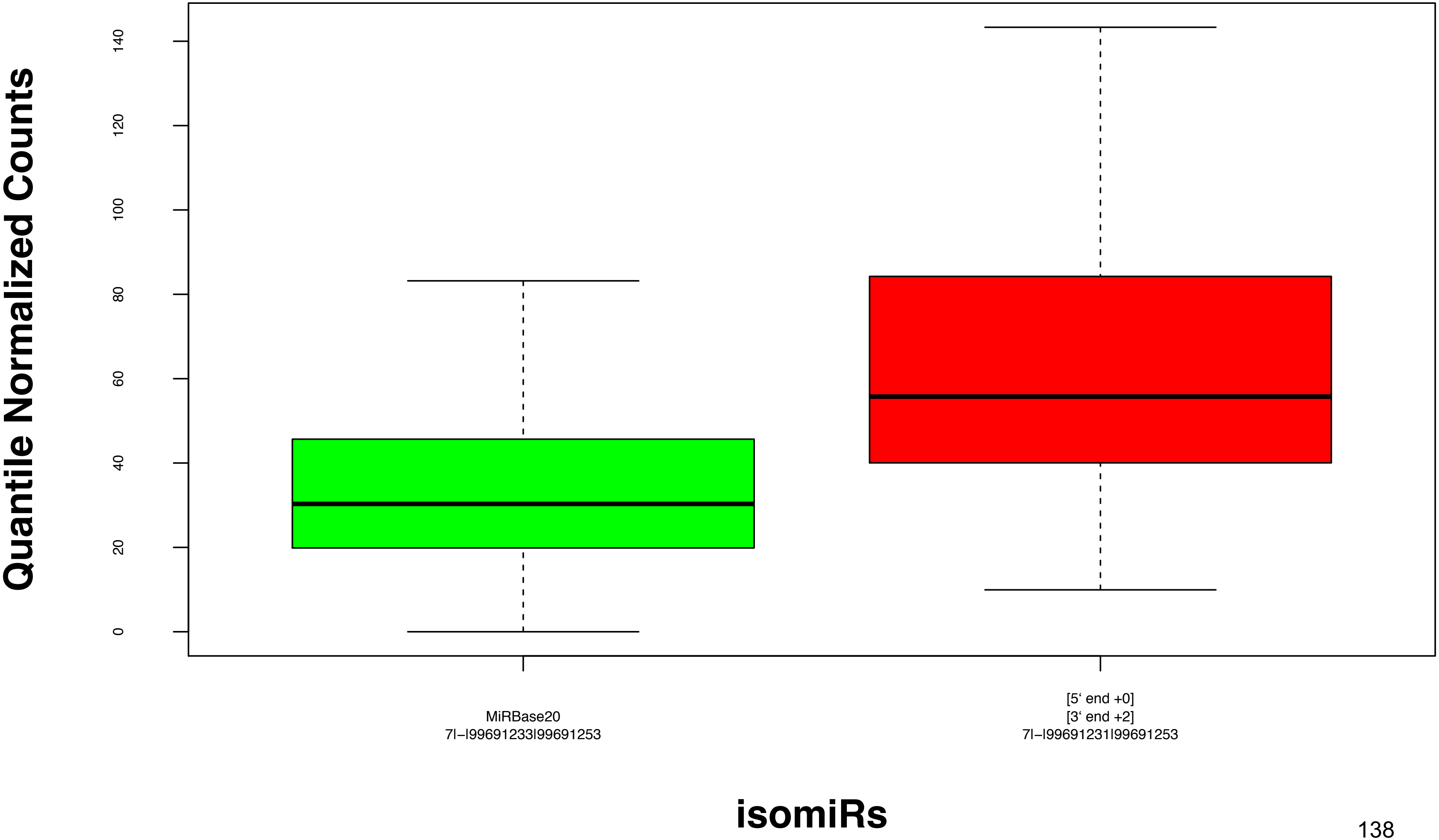

Supp. Figure 1
(pages 1 to 194)

MIMAT0004749=hsa−miR−424−3p=MI0001446

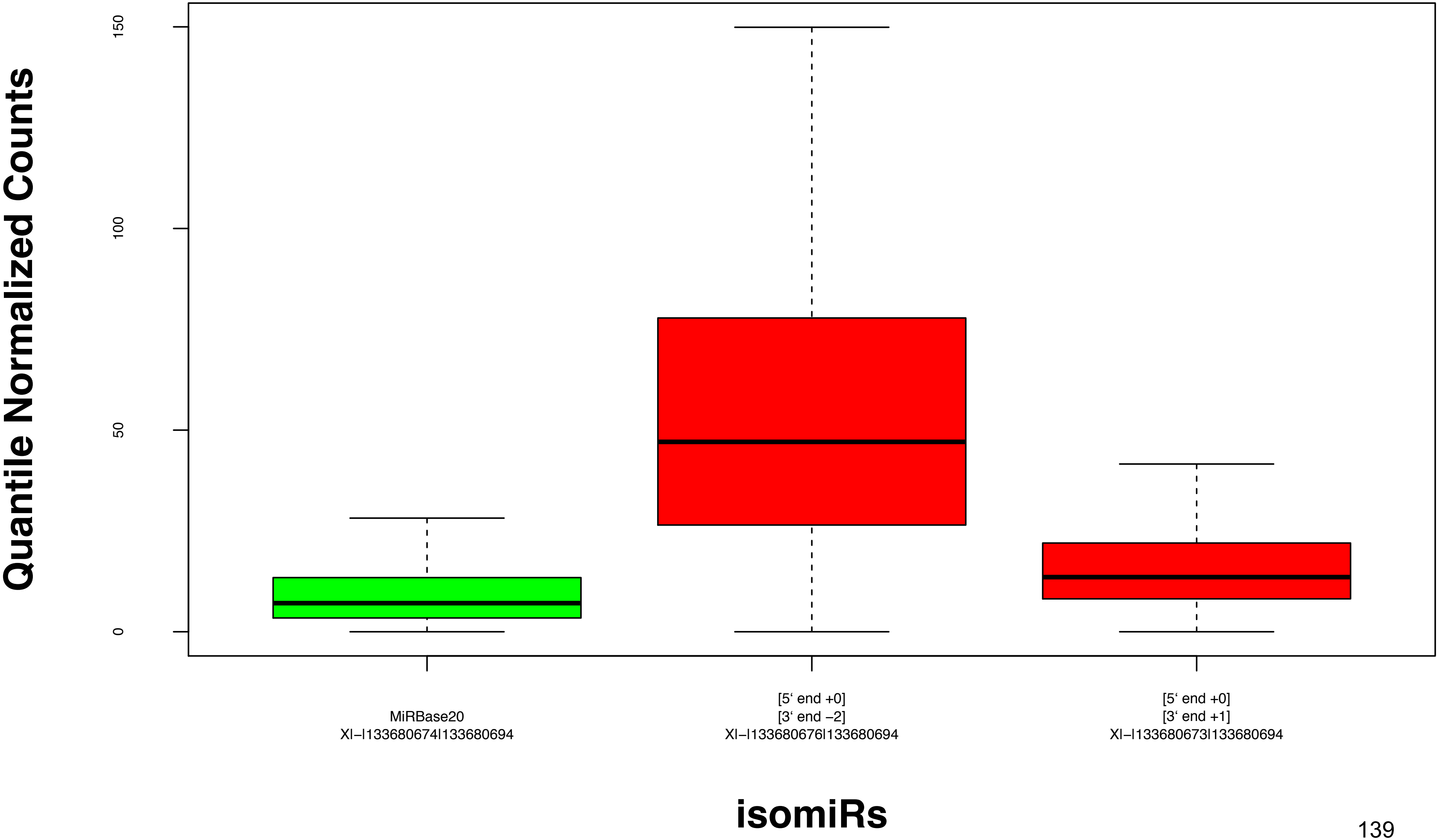

Supp. Figure 1
(pages 1 to 194)

MIMAT0022720=hsa–miR–1304–3p=MI0006371

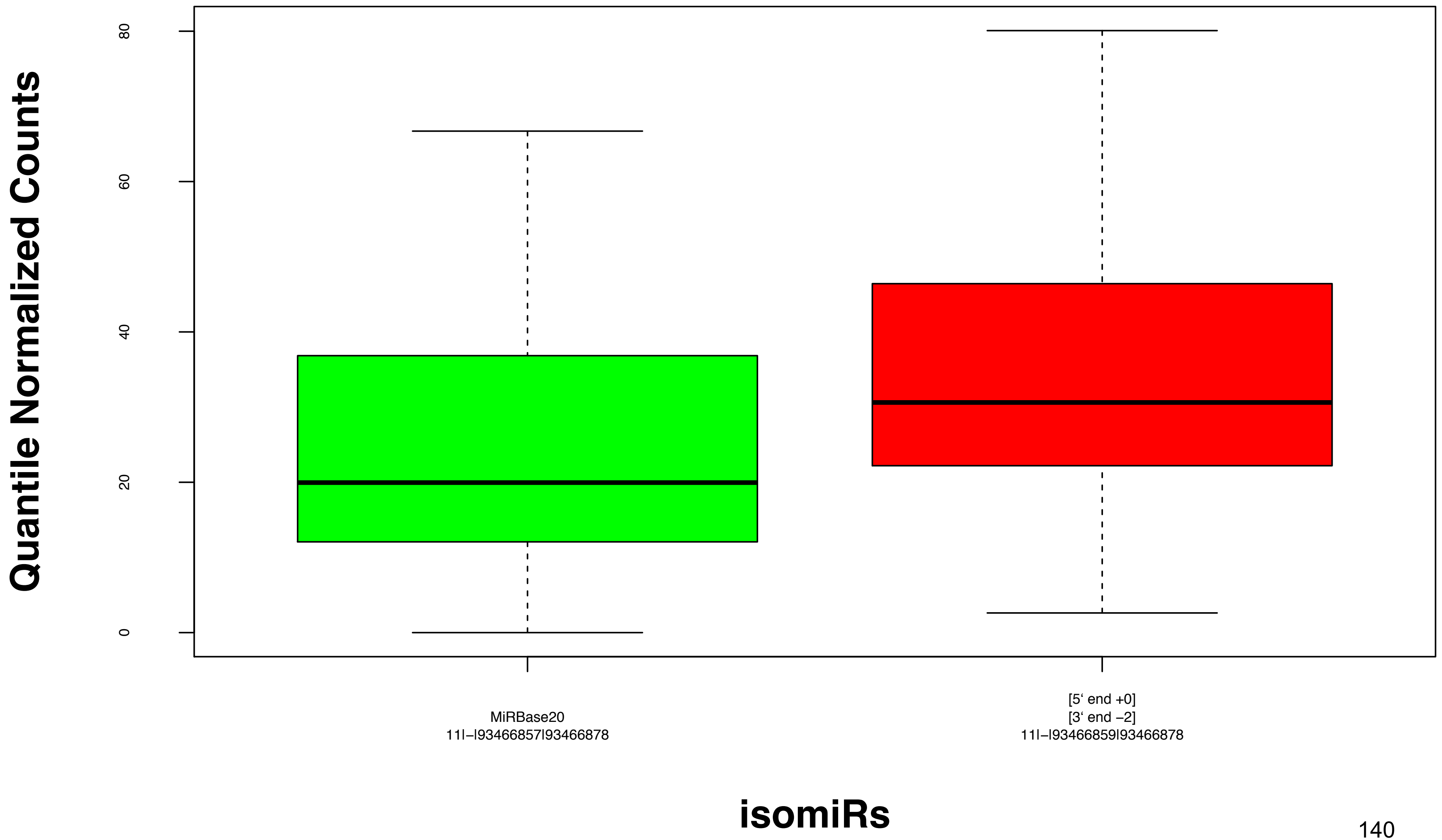

Supp. Figure 1
(pages 1 to 194)

MIMAT0004496=hsa−miR−23a−5p=MI0000079

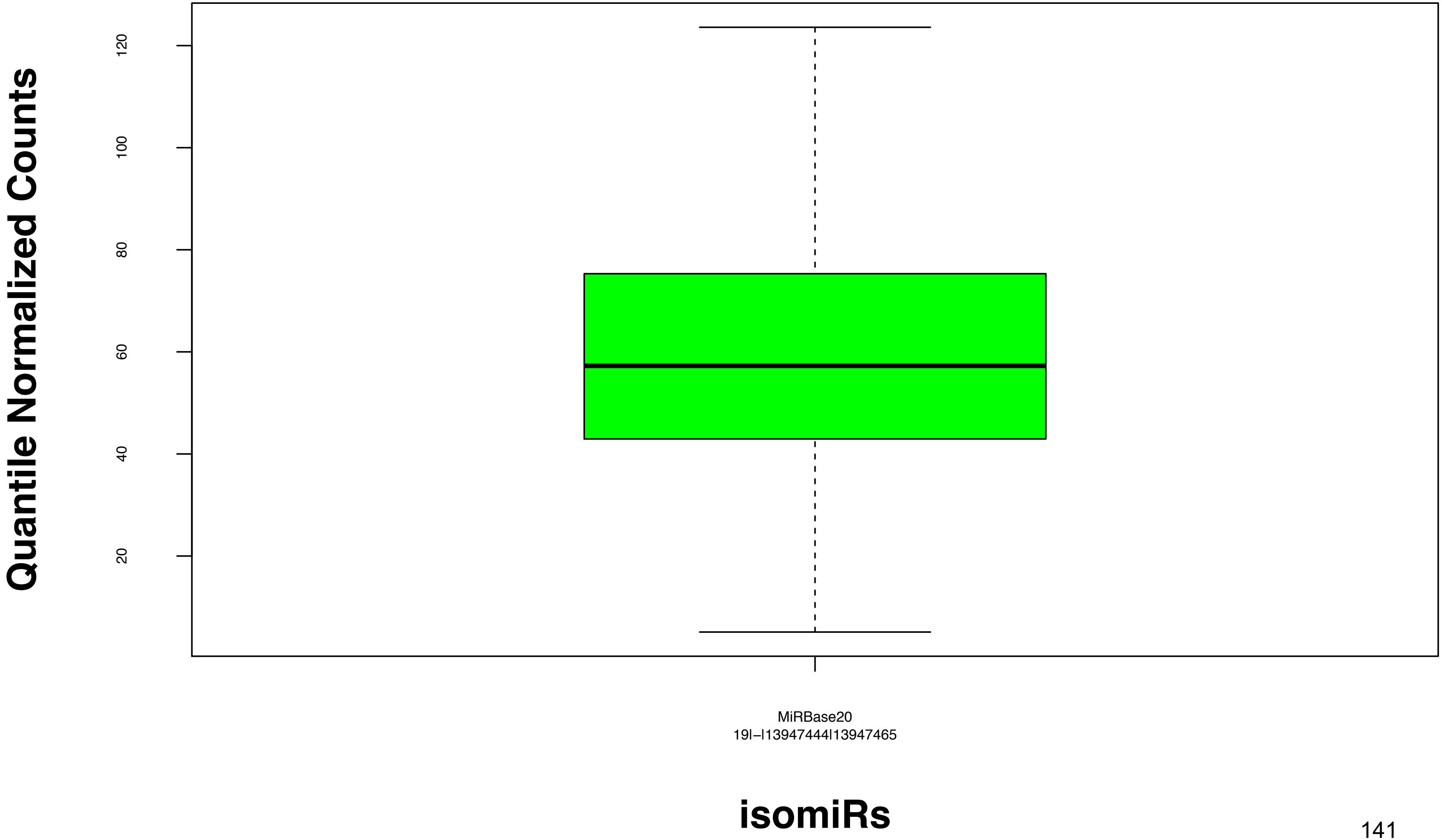

Supp. Figure 1
(pages 1 to 194)

MIMAT0005882=hsa−miR−548k=MI0006354

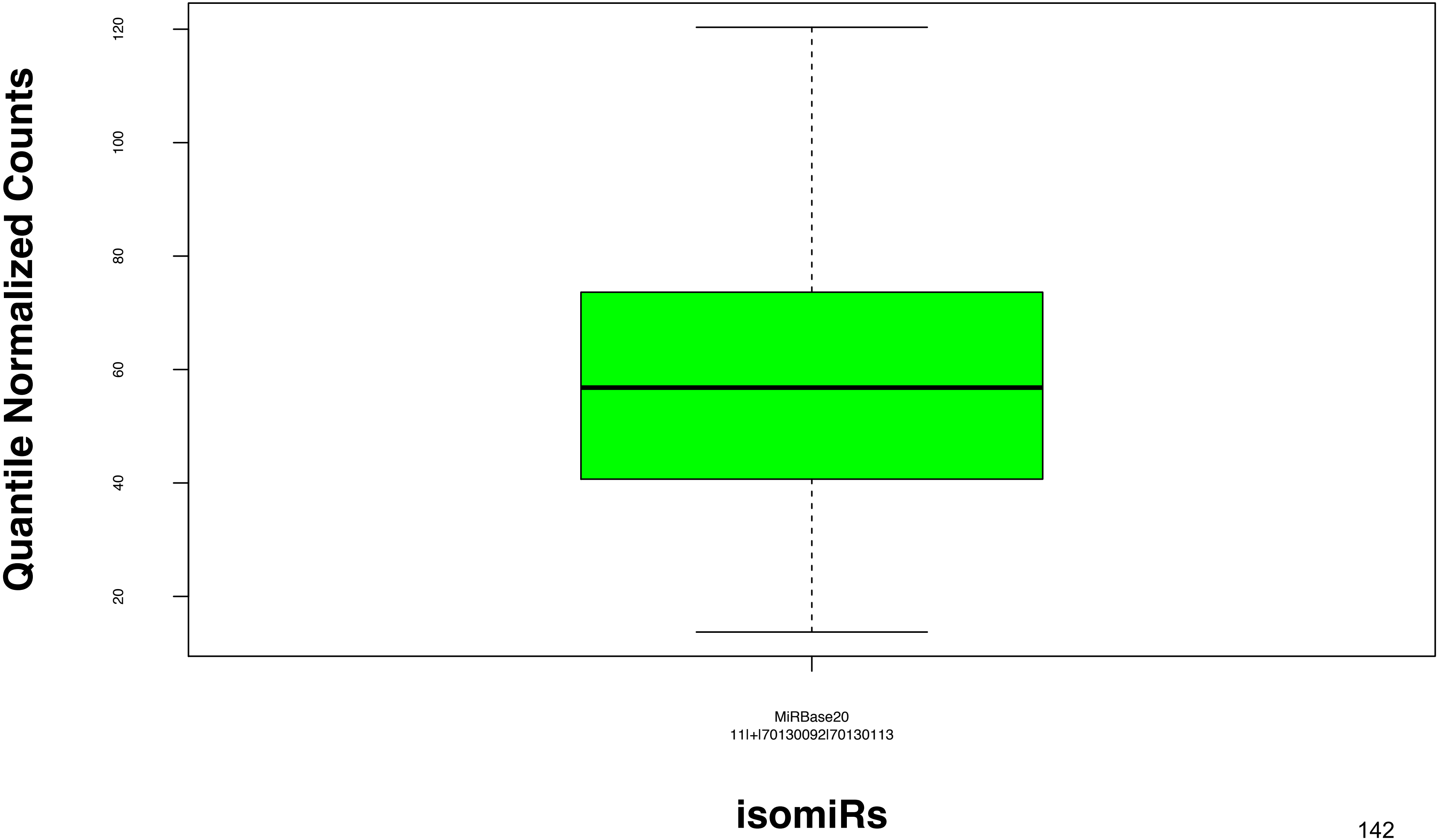

Supp. Figure 1
(pages 1 to 194)

MIMAT0004774=hsa−miR−501−3p=MI0003185

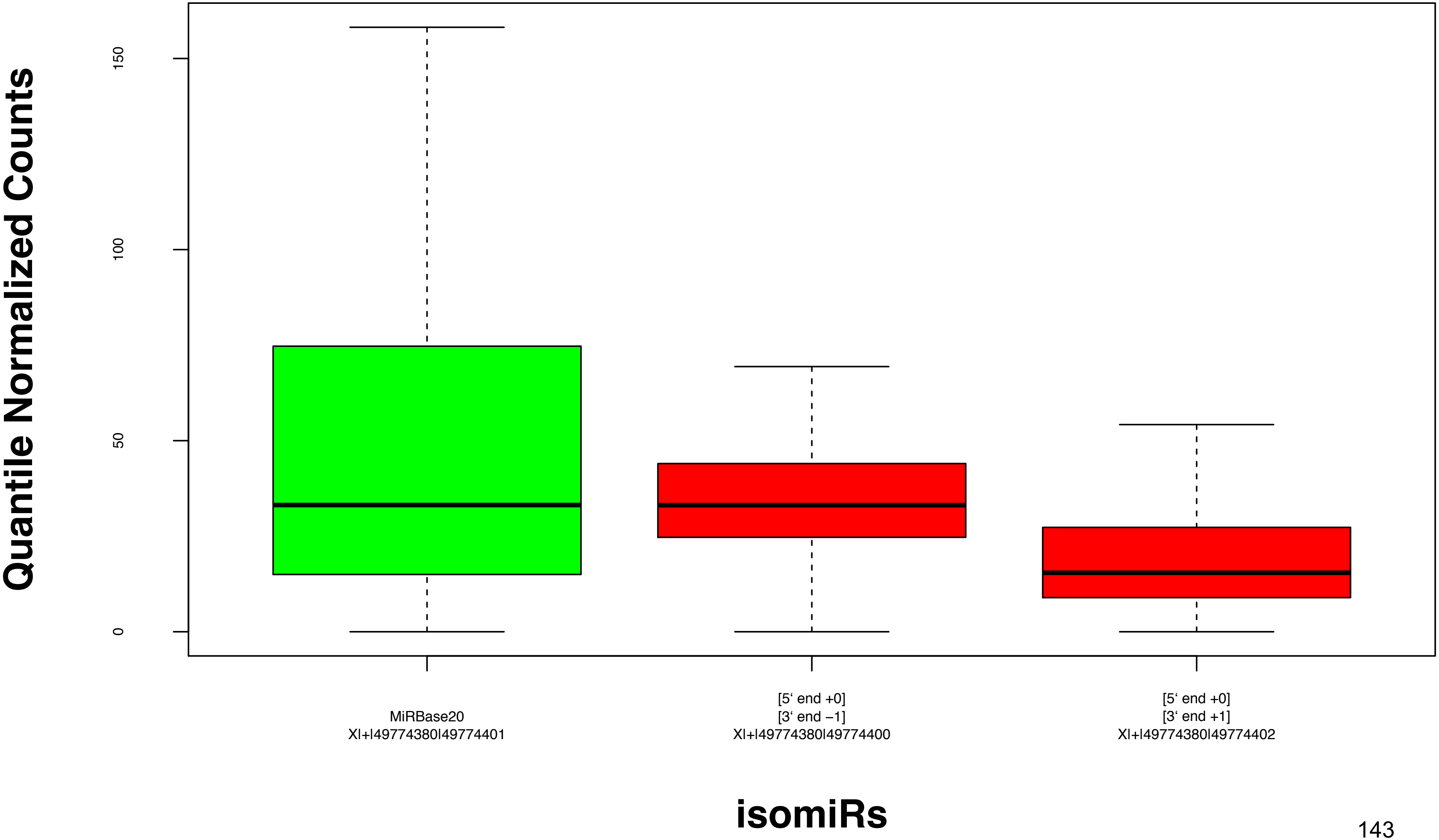

Supp. Figure 1
(pages 1 to 194)

MIMAT0000617=hsa–miR–200c–3p=MI0000650

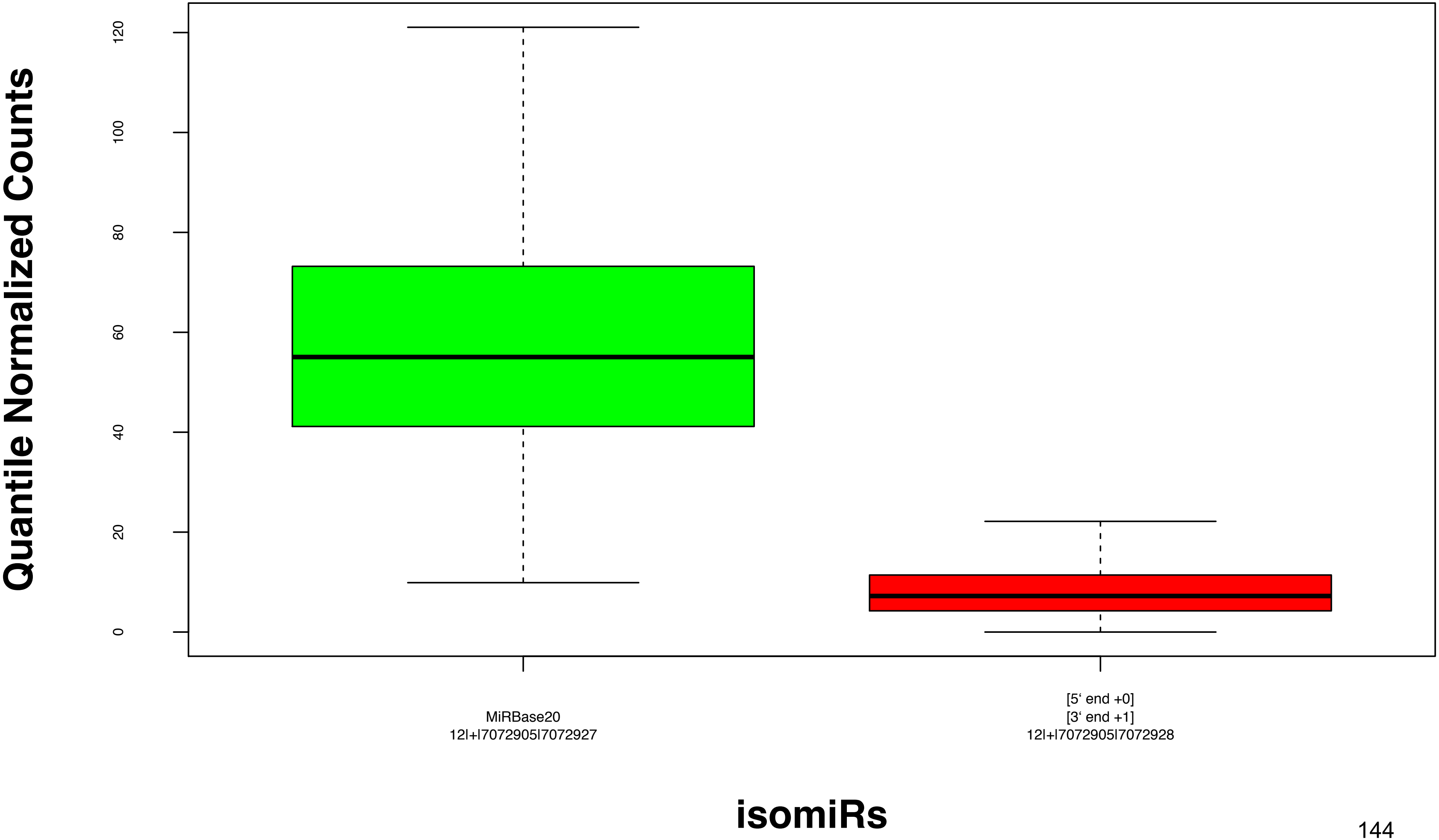

Supp. Figure 1
(pages 1 to 194)

MIMAT0002891=hsa−miR−18a−3p=MI0000072

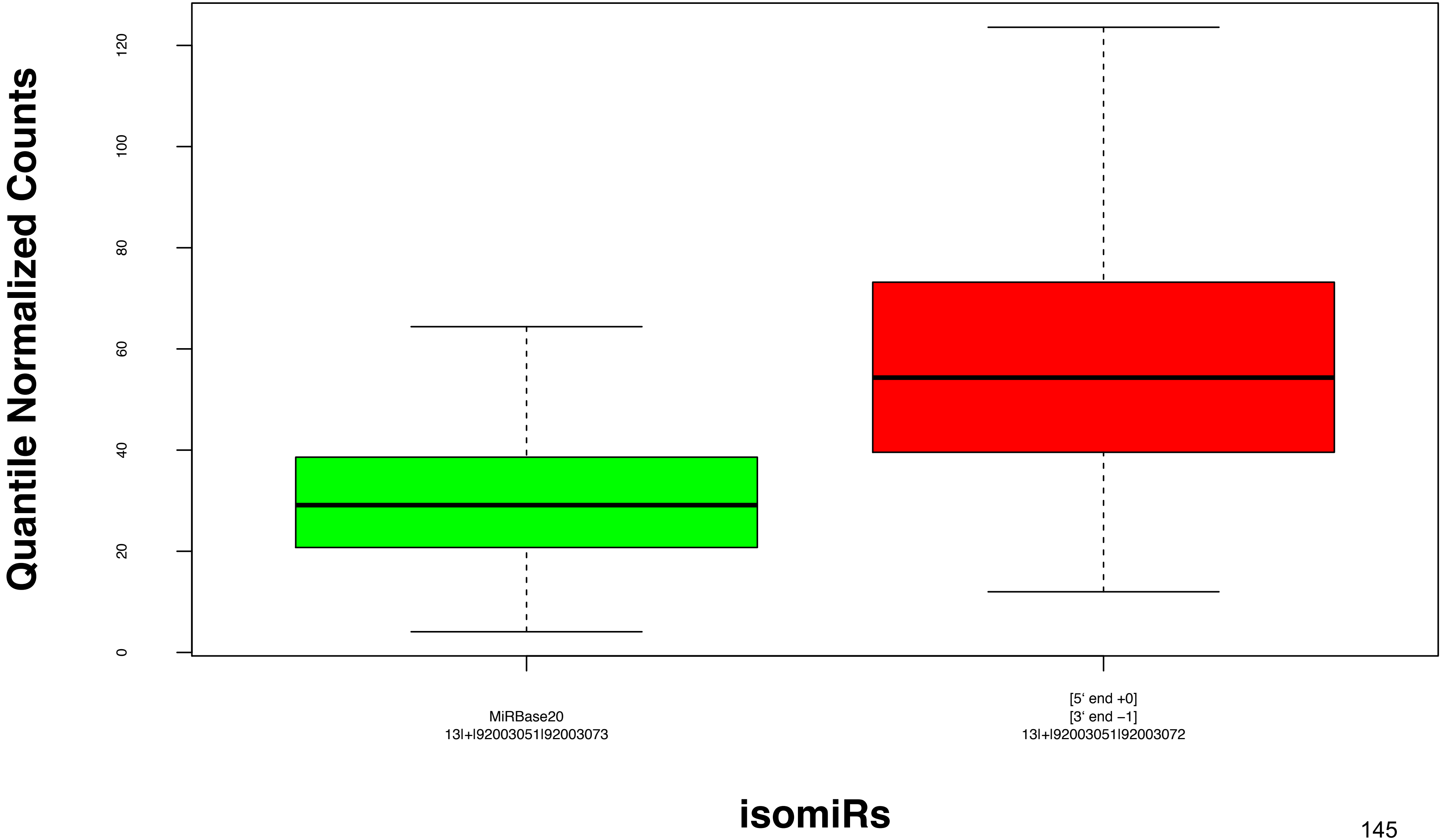

Supp. Figure 1
(pages 1 to 194)

MIMAT0004810=hsa−miR−629−5p=MI0003643

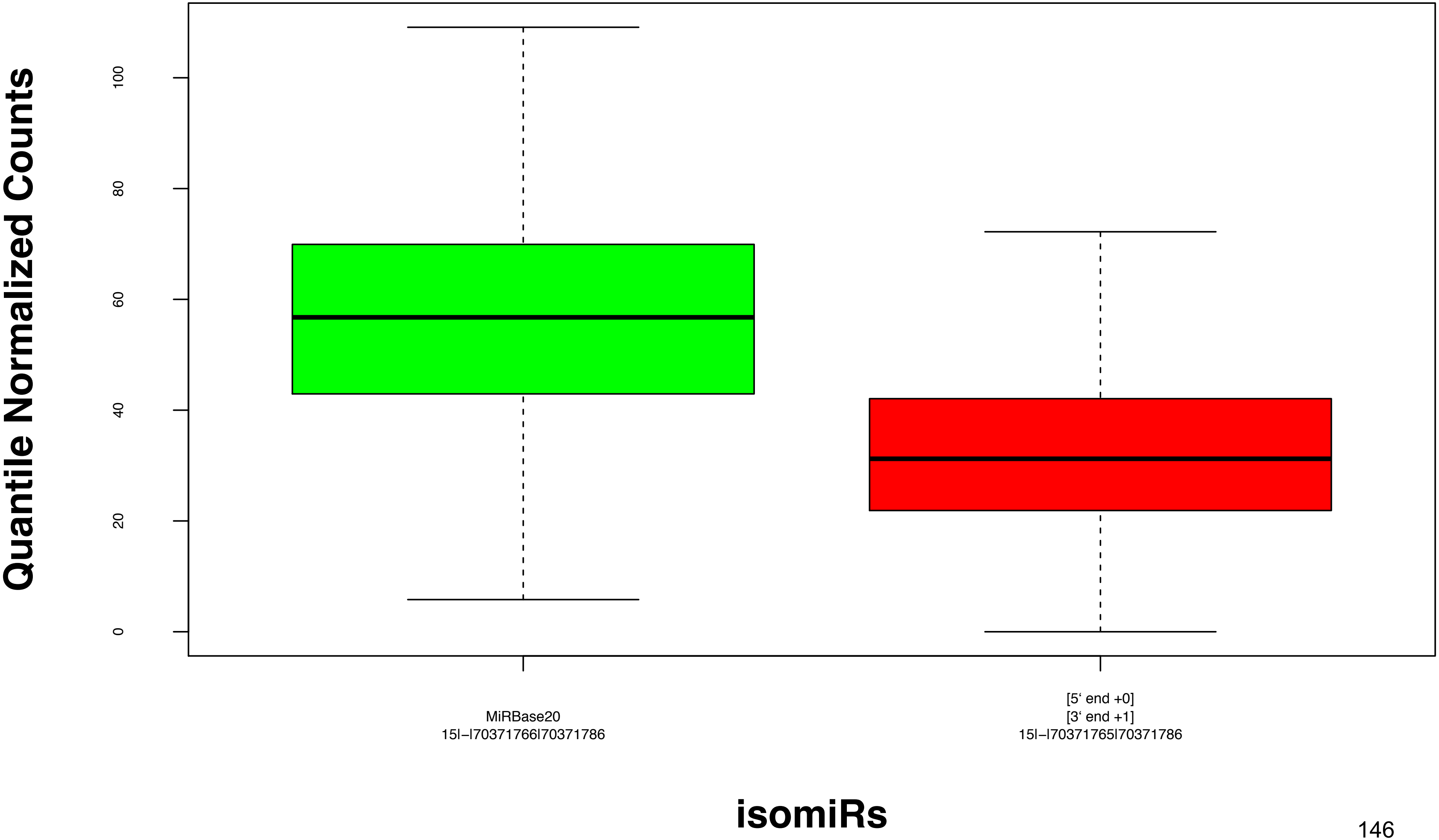

Supp. Figure 1
(pages 1 to 194)

MIMAT0000705=hsa−miR−362−5p=MI0000762

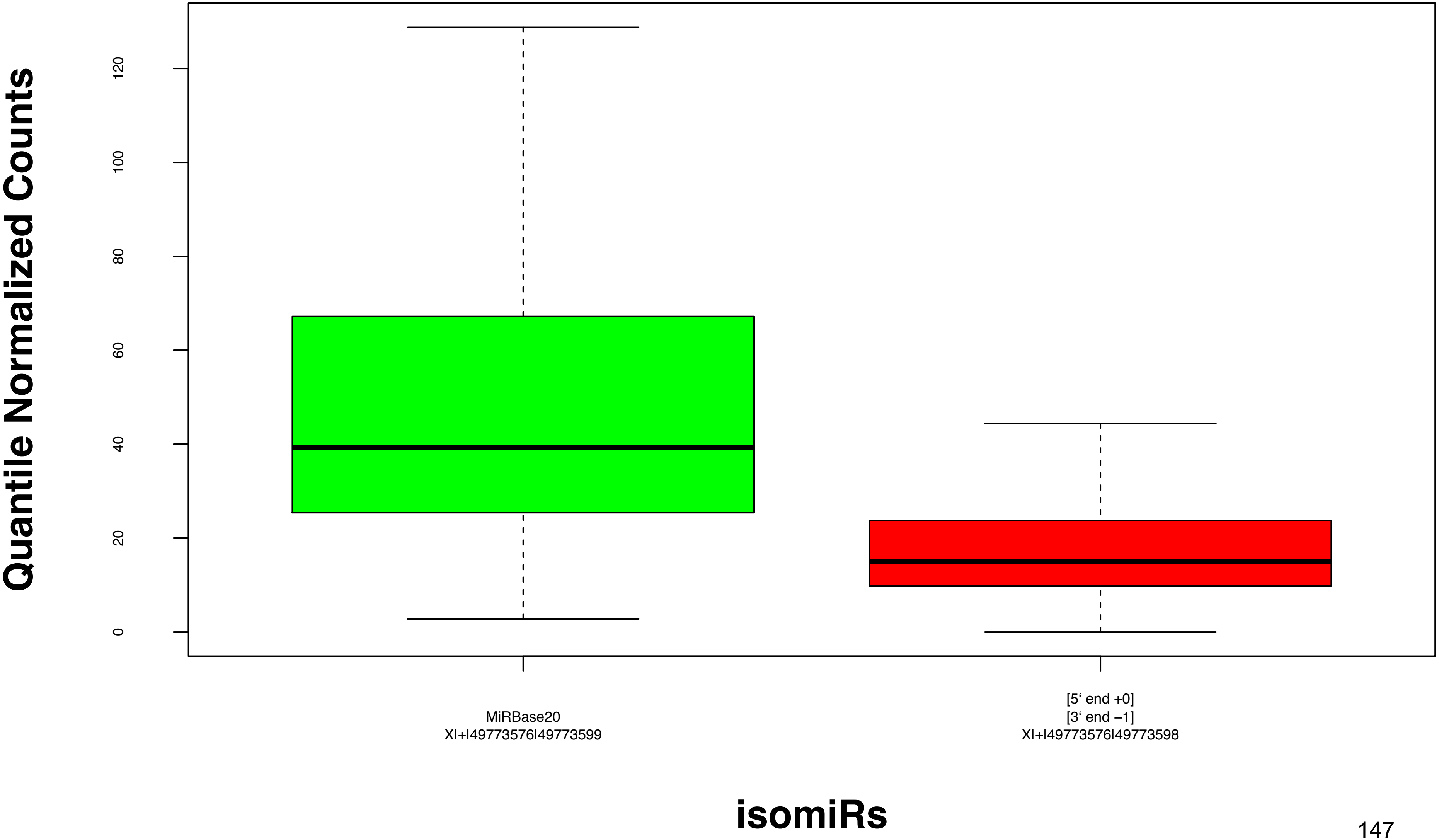

Supp. Figure 1
(pages 1 to 194)

MIMAT0003241=hsa−miR−576−5p=MI0003583

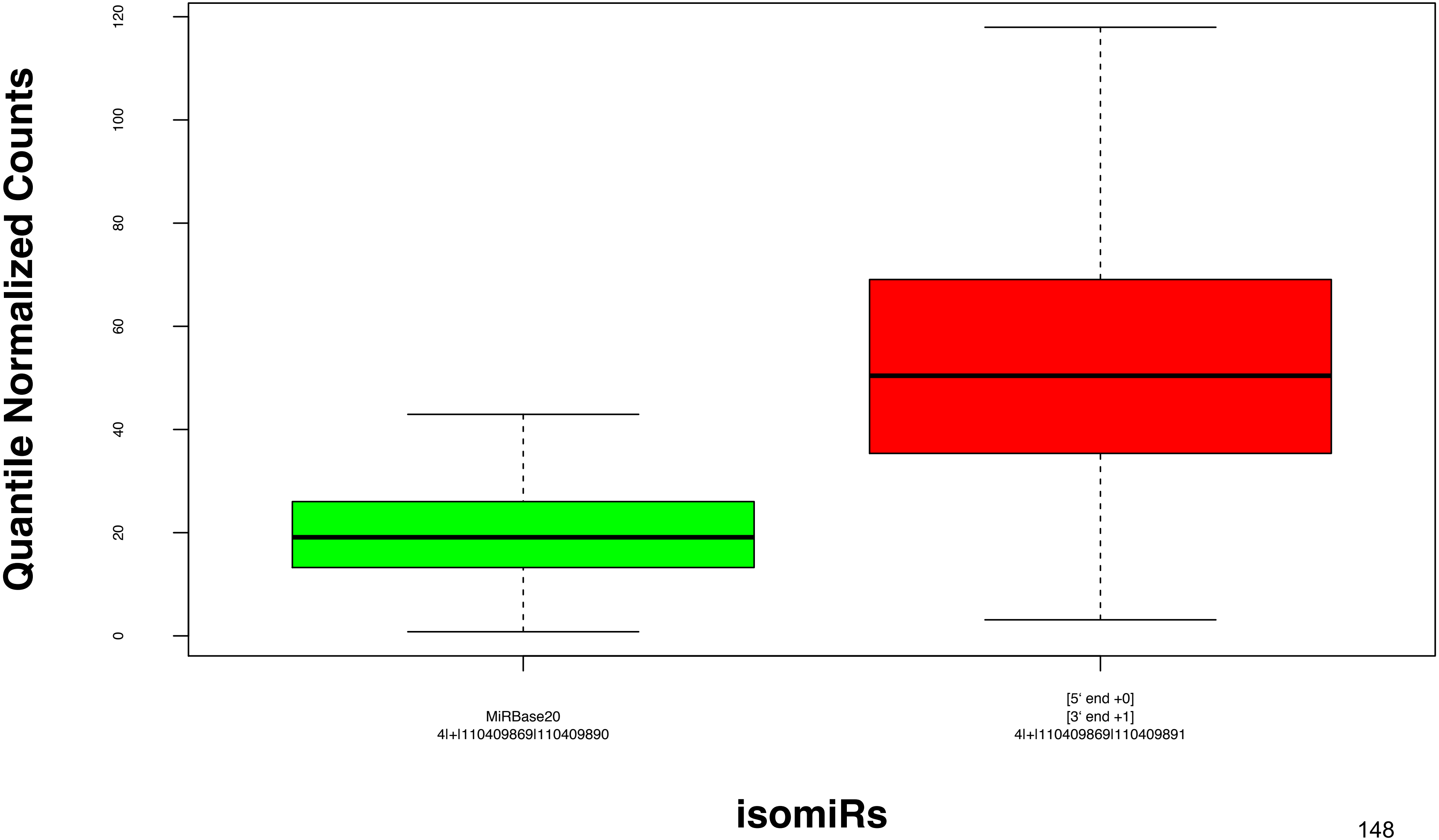

Supp. Figure 1
(pages 1 to 194)

MIMAT0003239=hsa−miR−574−3p=MI0003581

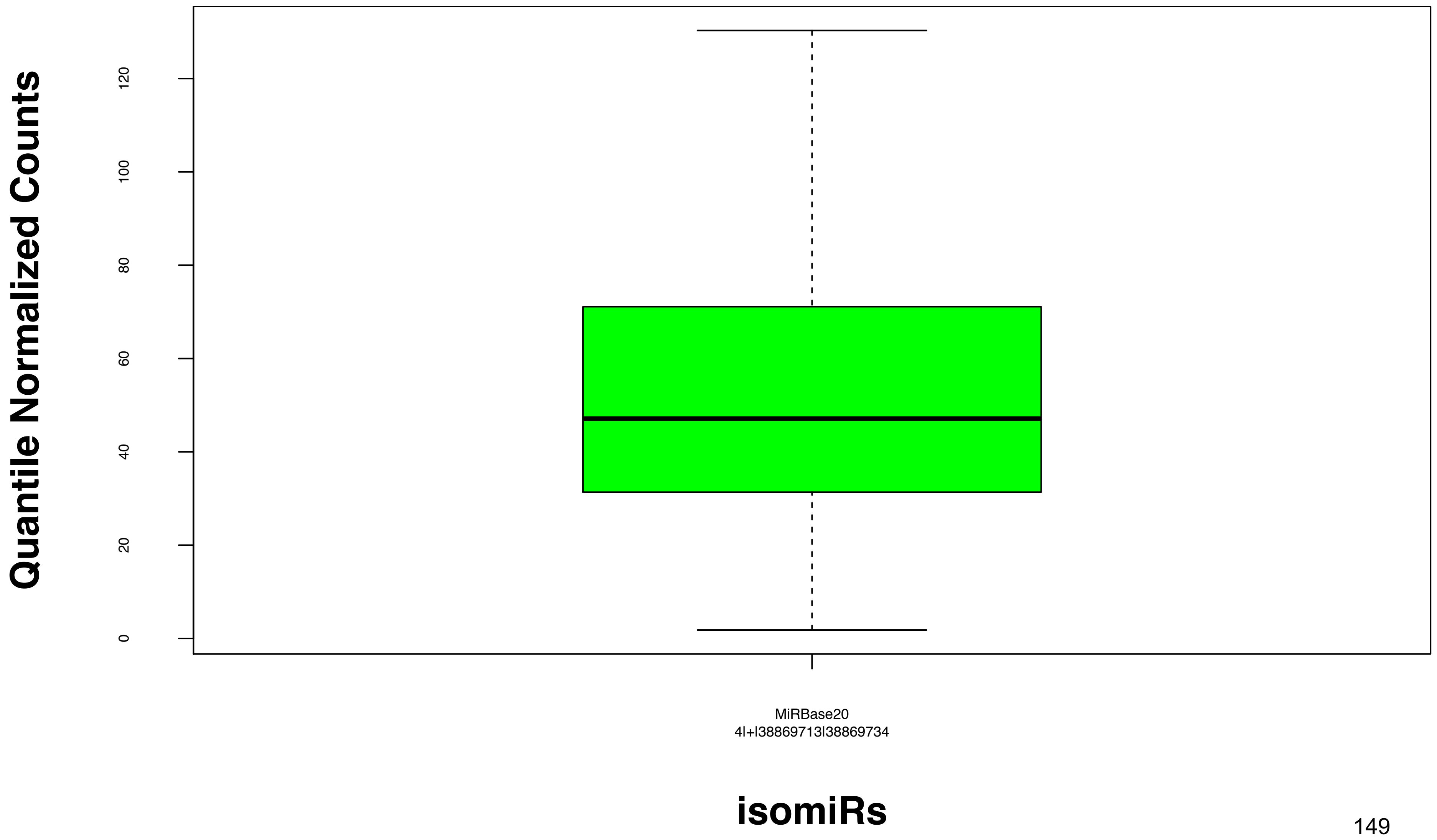

Supp. Figure 1
(pages 1 to 194)

MIMAT0000099_1=hsa−miR−101−3p=MI0000739

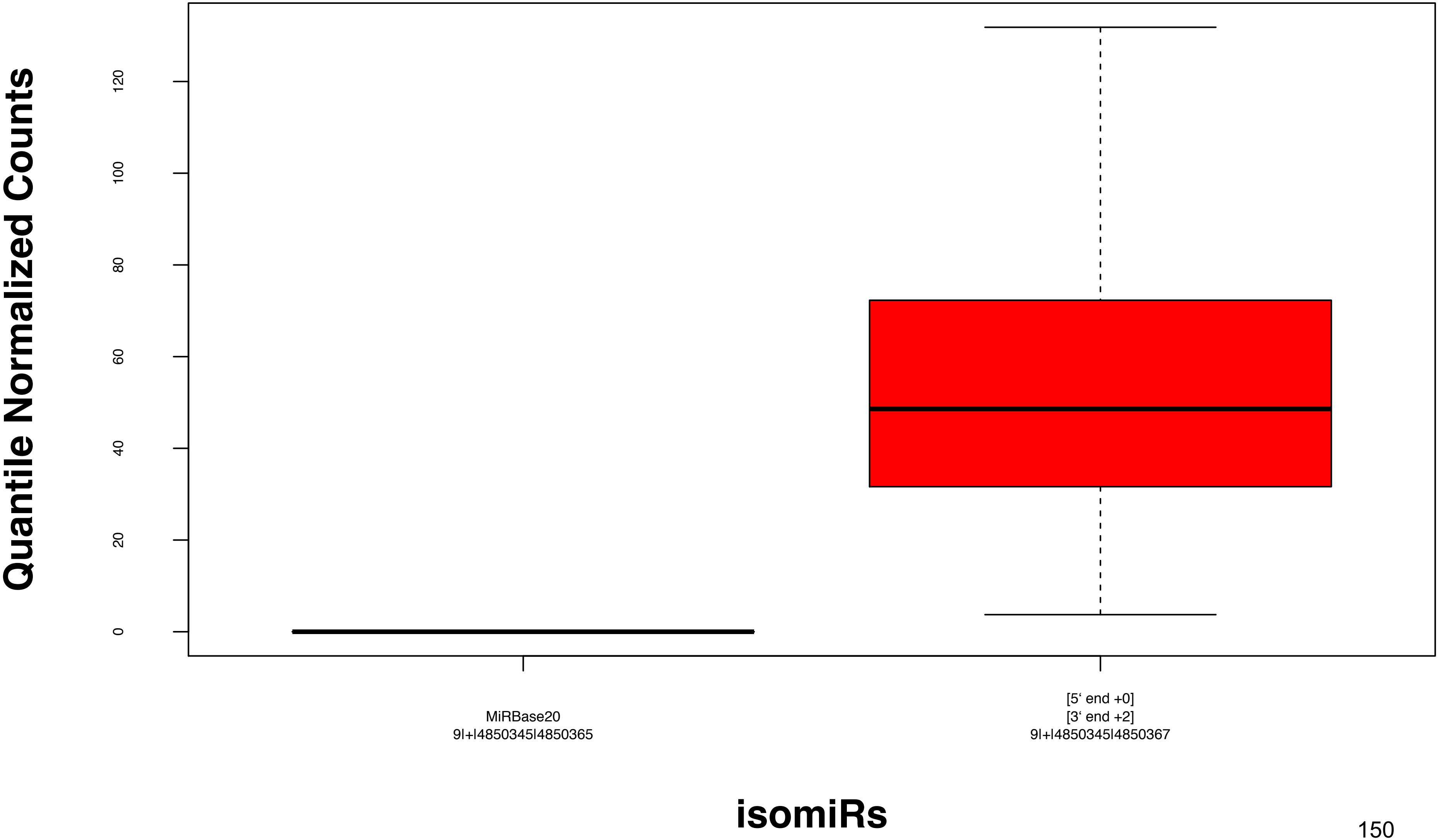

Supp. Figure 1
(pages 1 to 194)

MIMAT0004767=hsa–miR–193b–5p=MI0003137

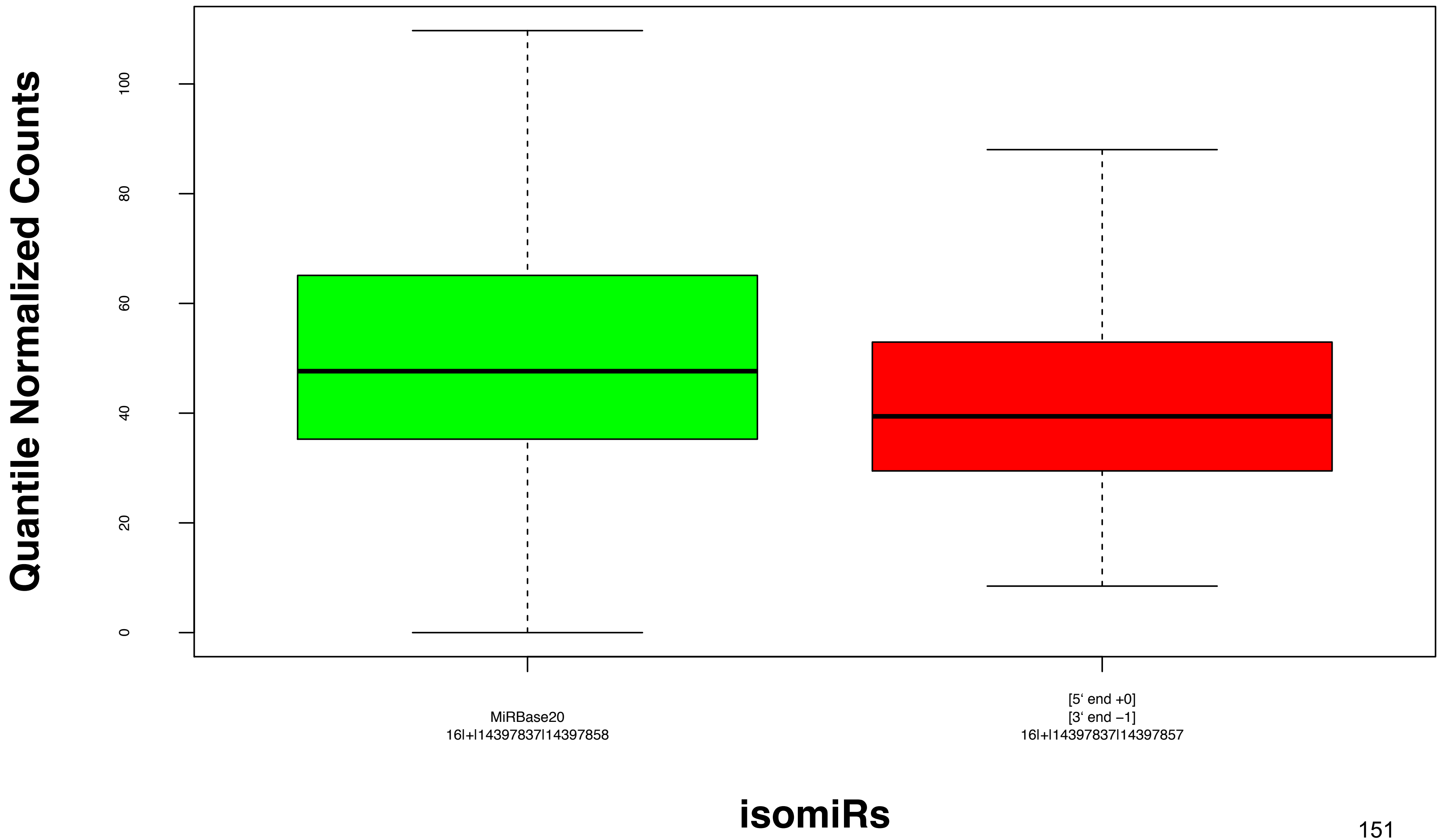

Supp. Figure 1
(pages 1 to 194)

MIMAT0004985=hsa–miR–942–5p=MI0005767

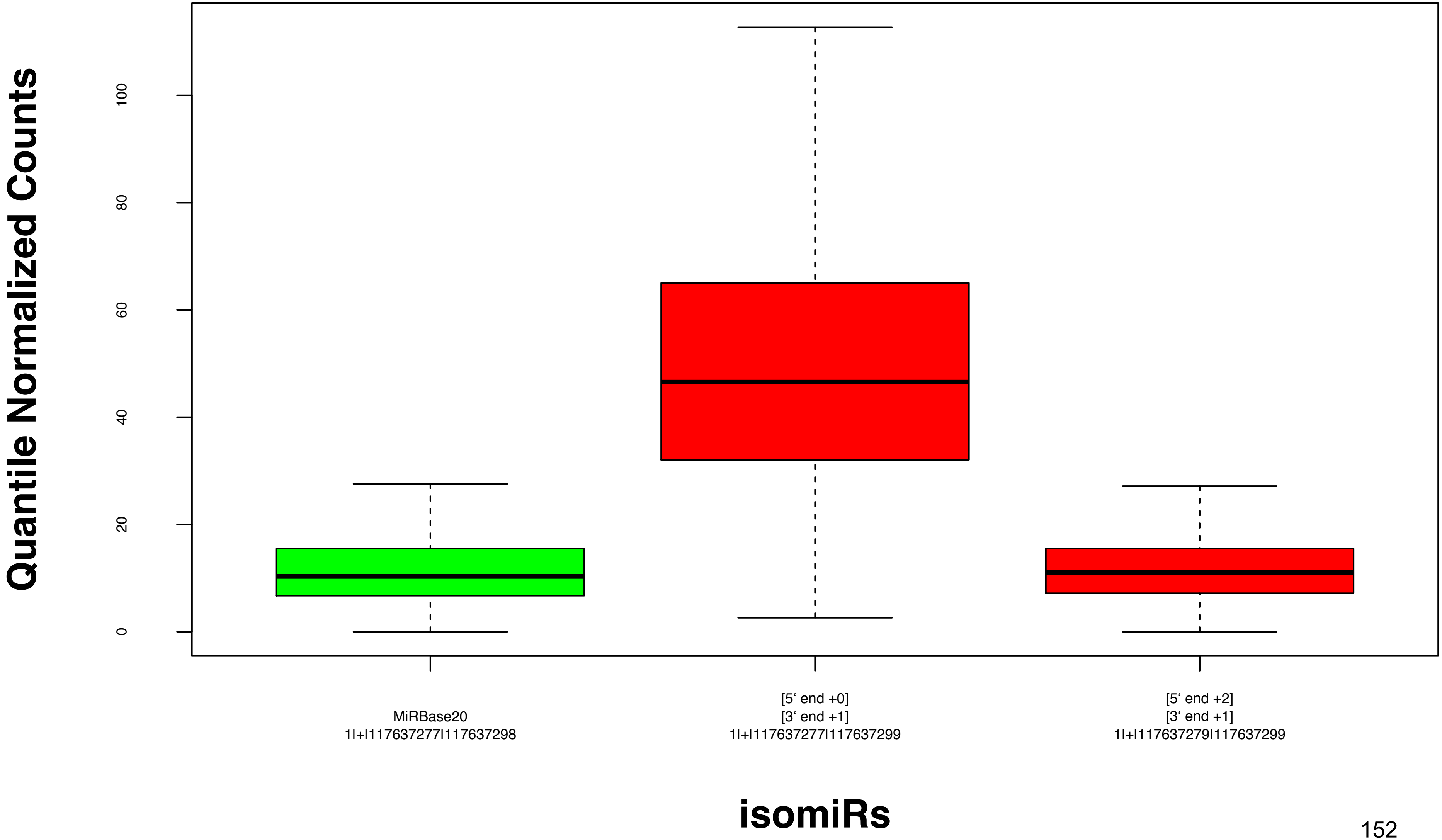

Supp. Figure 1
(pages 1 to 194)

MIMAT0004955=hsa–miR–374b–5p=MI0005566

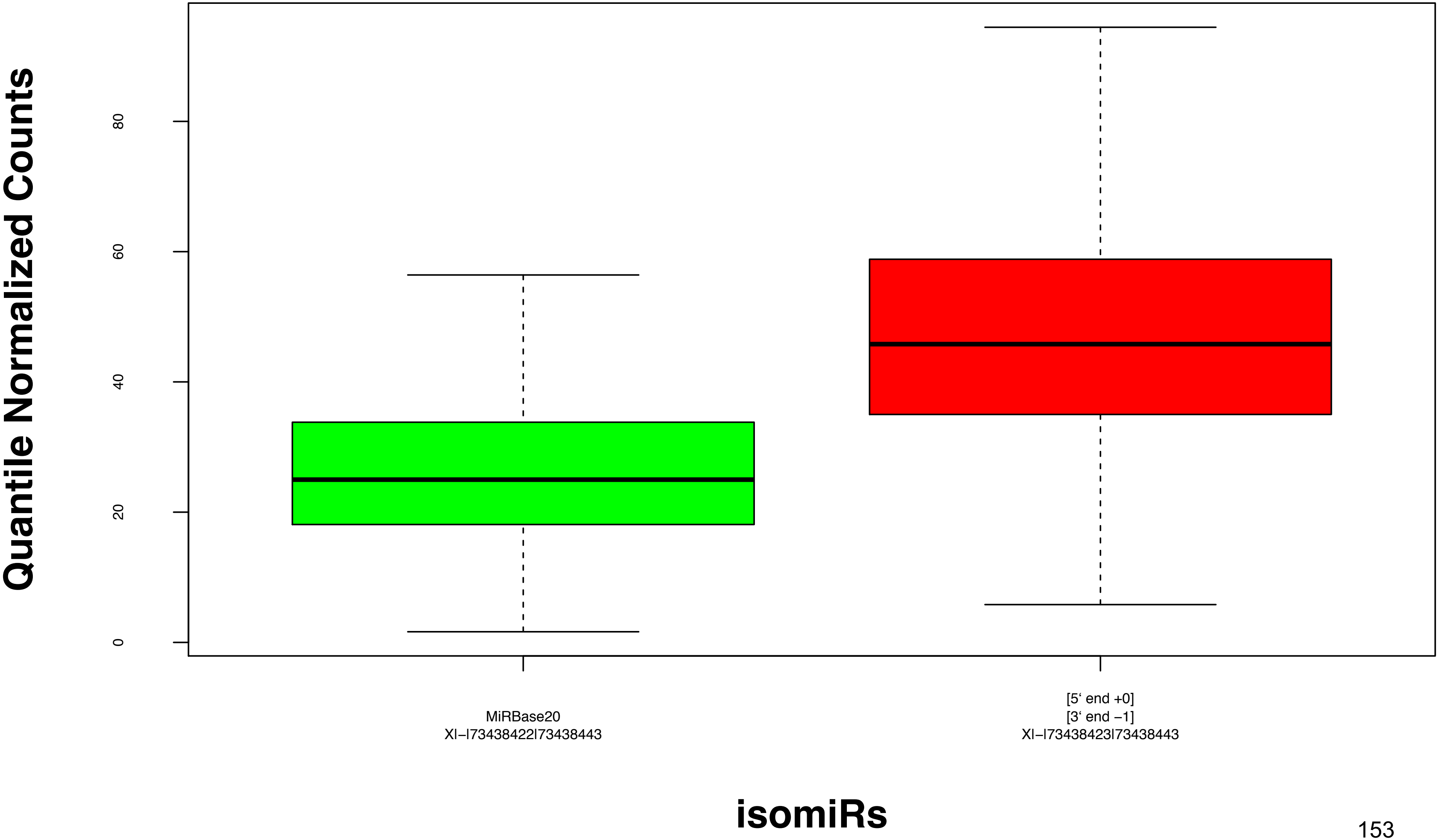

Supp. Figure 1
(pages 1 to 194)

MIMAT0000424=hsa−miR−128−3p=MI0000447

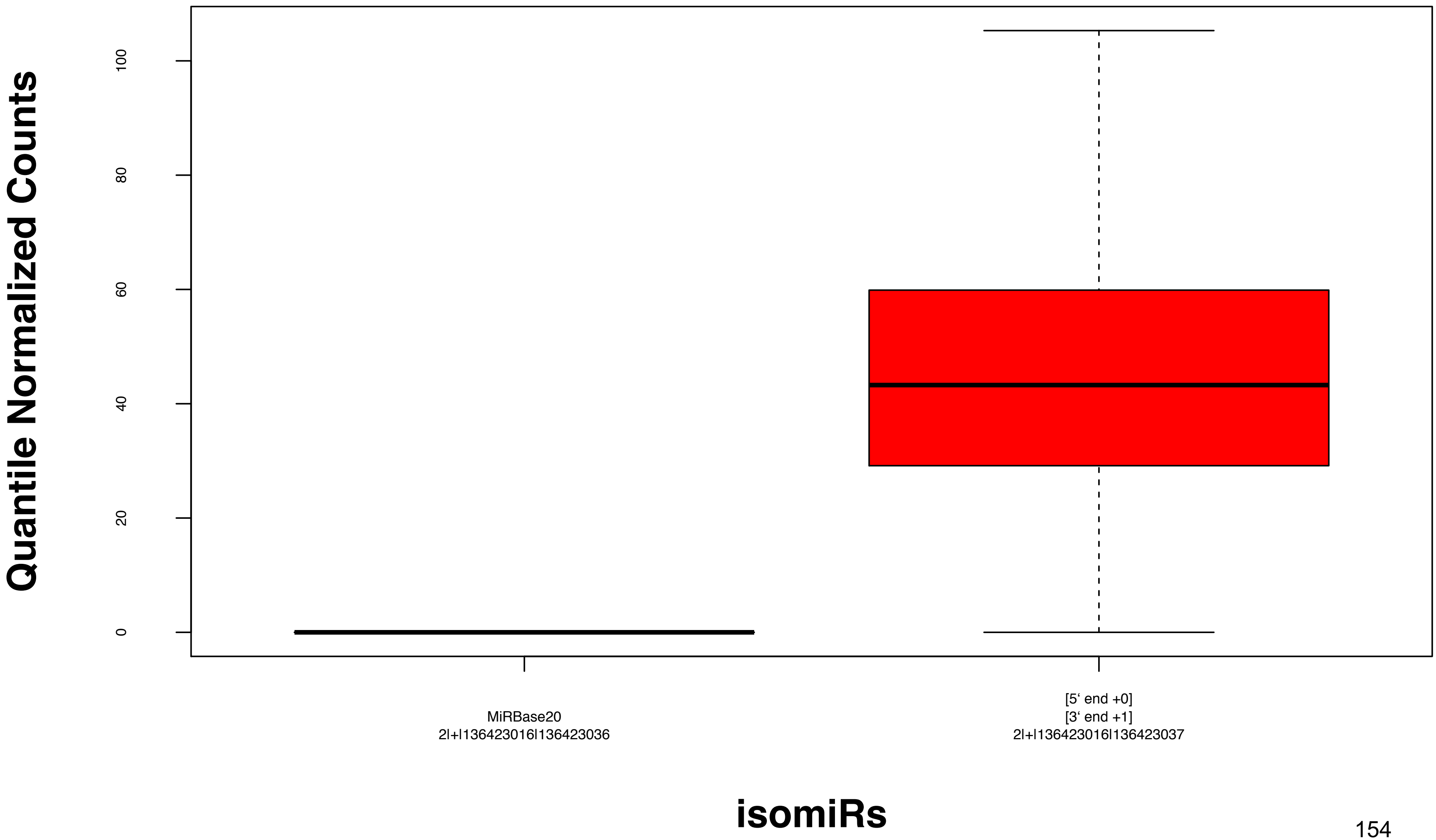

Supp. Figure 1
(pages 1 to 194)

MIMAT0006789=hsa−miR−1468−5p=MI0003782

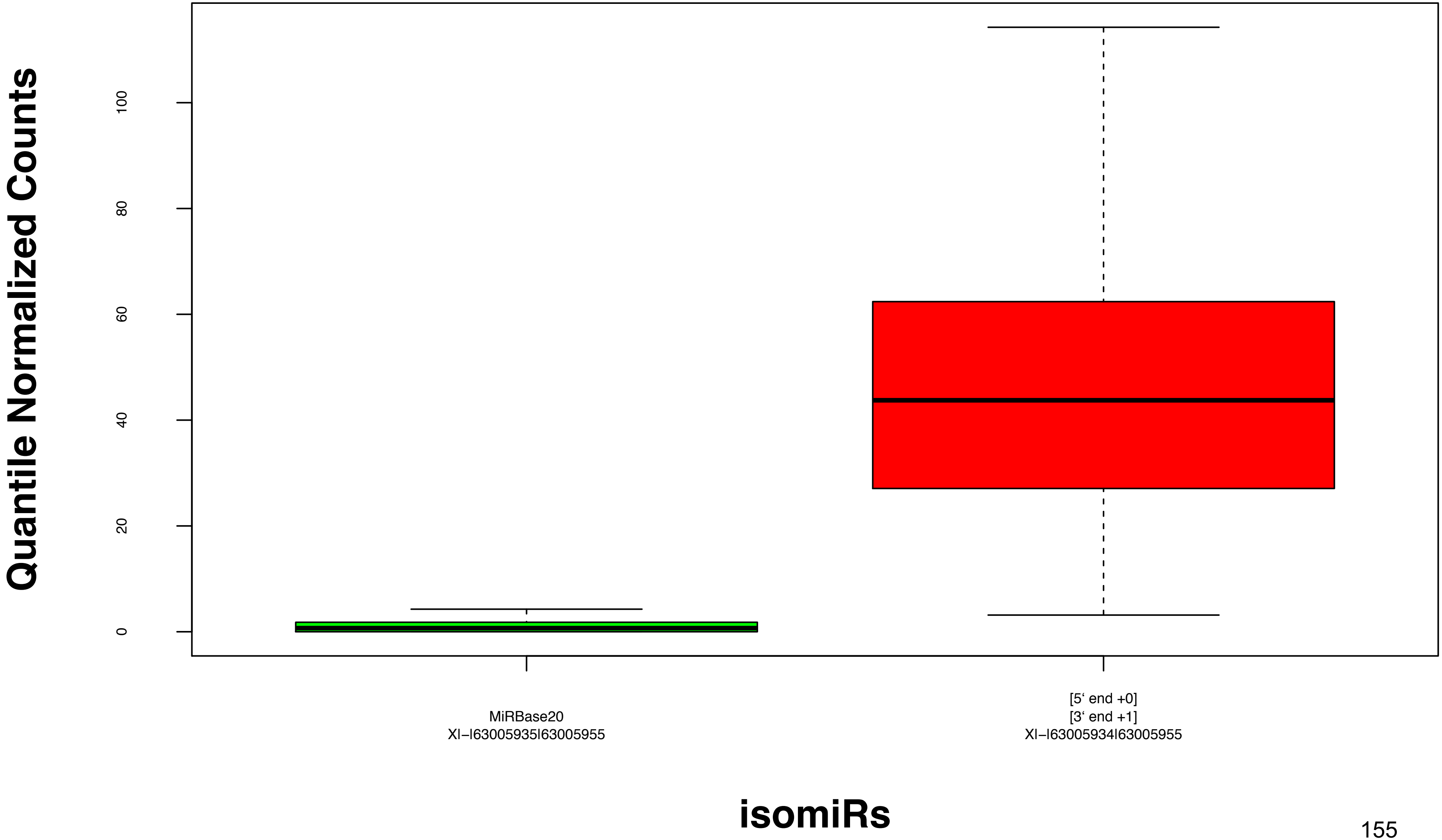

Supp. Figure 1
(pages 1 to 194)

MIMAT0000062_2=hsa−let−7a−5p=MI0000060

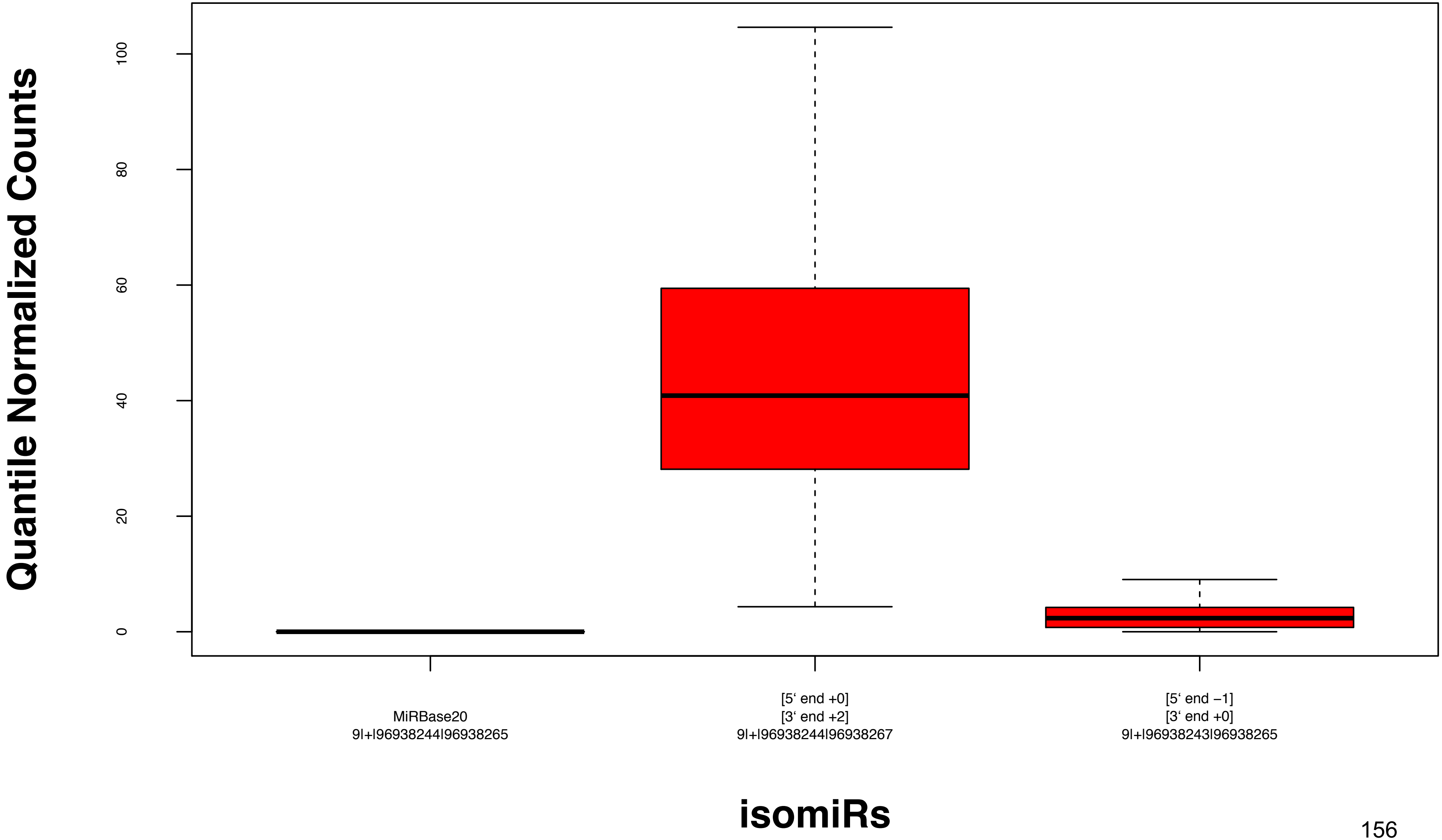

Supp. Figure 1
(pages 1 to 194)

MIMAT0004487=hsa–let–7f–2–3p=MI0000068

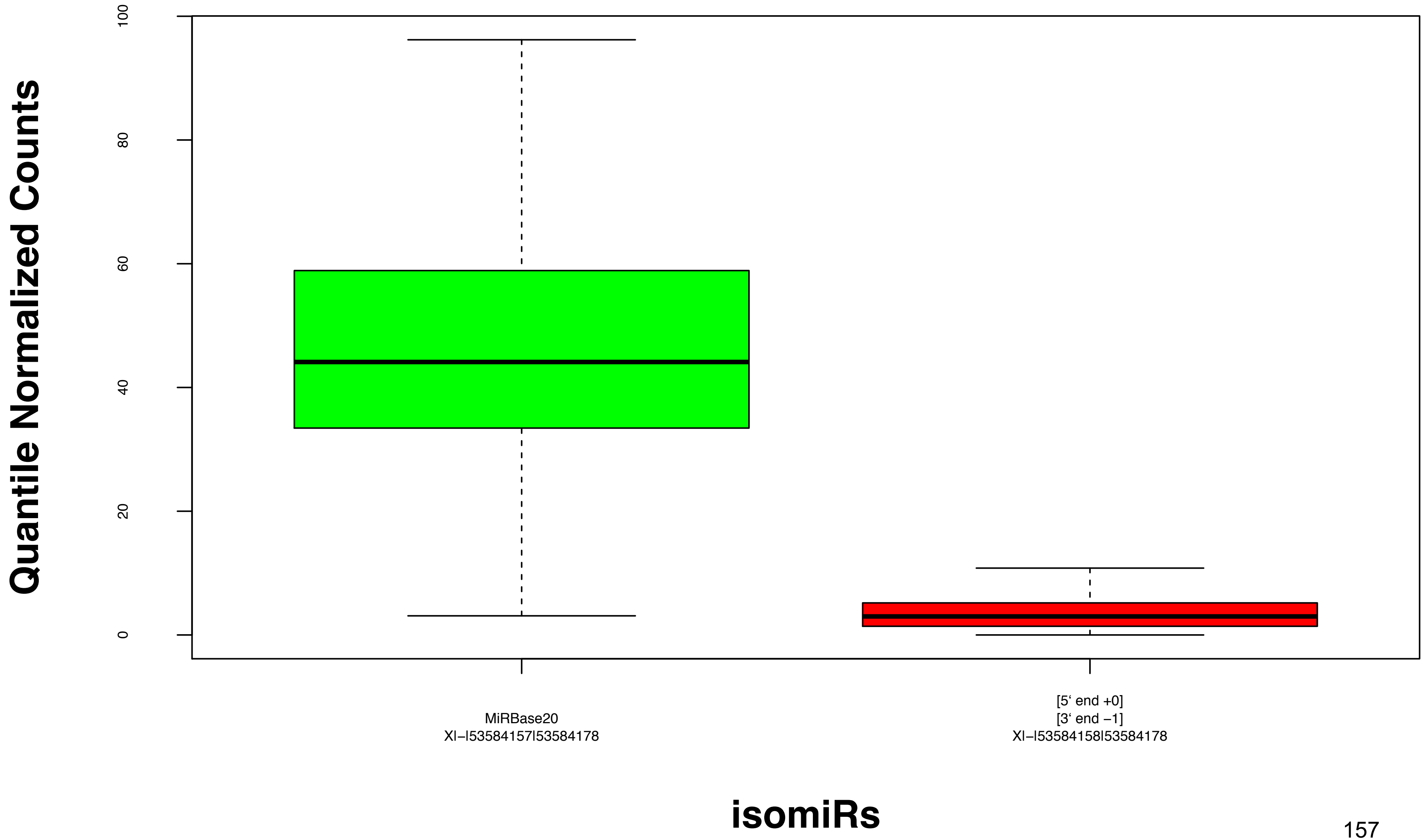

Supp. Figure 1
(pages 1 to 194)

MIMAT0000438=hsa−miR−152−3p=MI0000462

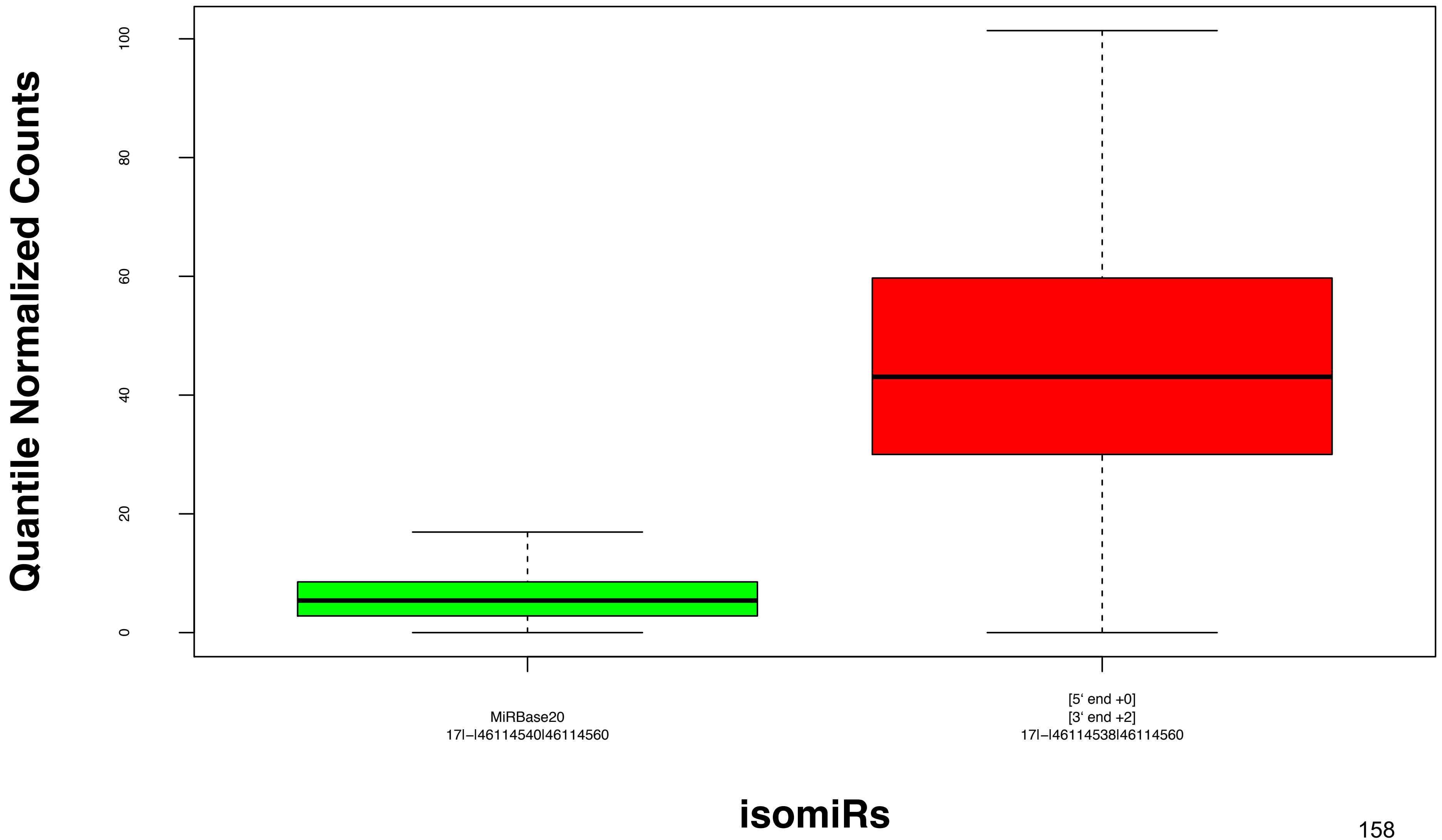

Supp. Figure 1
(pages 1 to 194)

MIMAT0000069=hsa−miR−16−5p=MI0000070

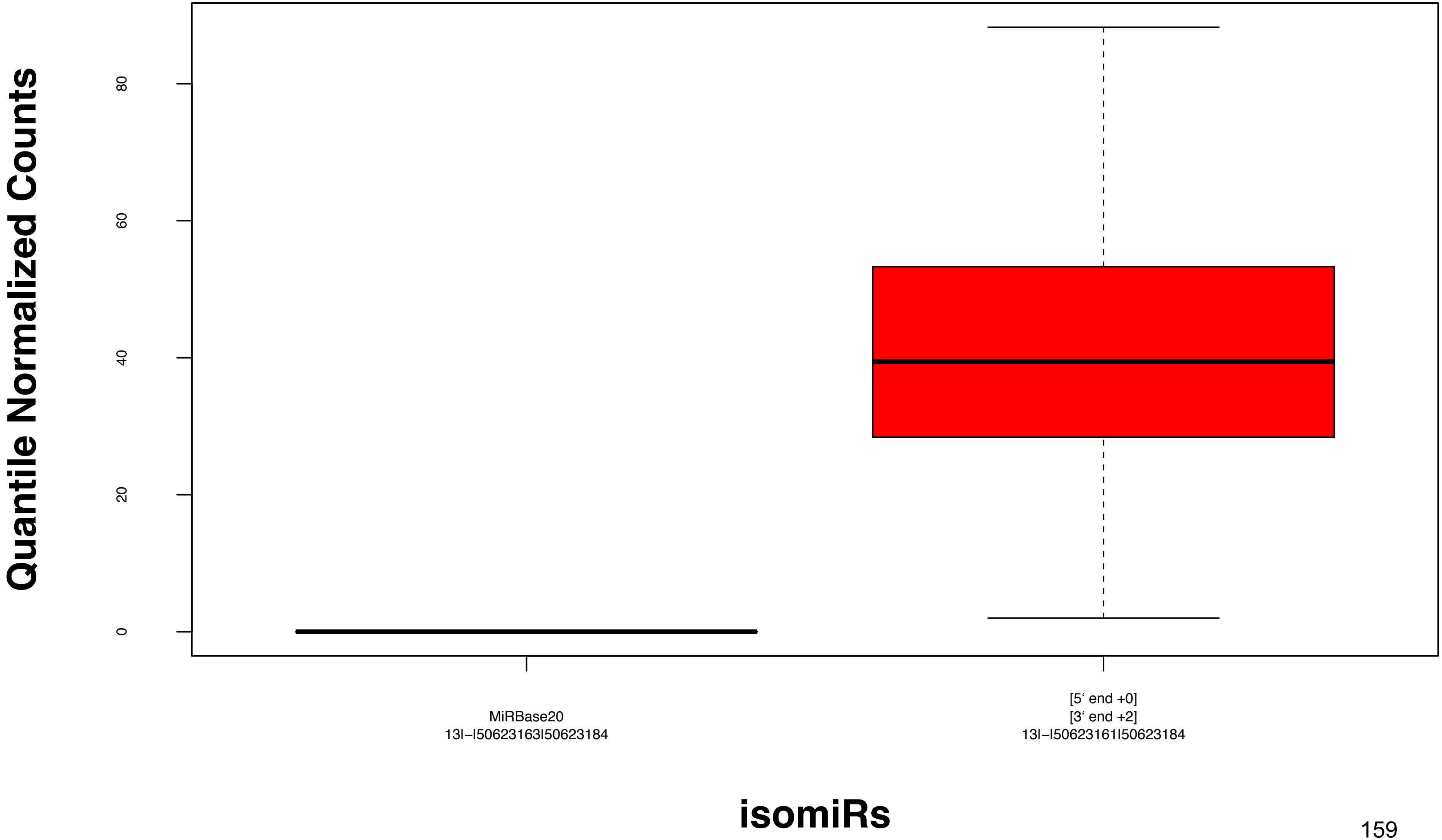

Supp. Figure 1
(pages 1 to 194)

MIMAT0000727=hsa−miR−374a−5p=MI0000782

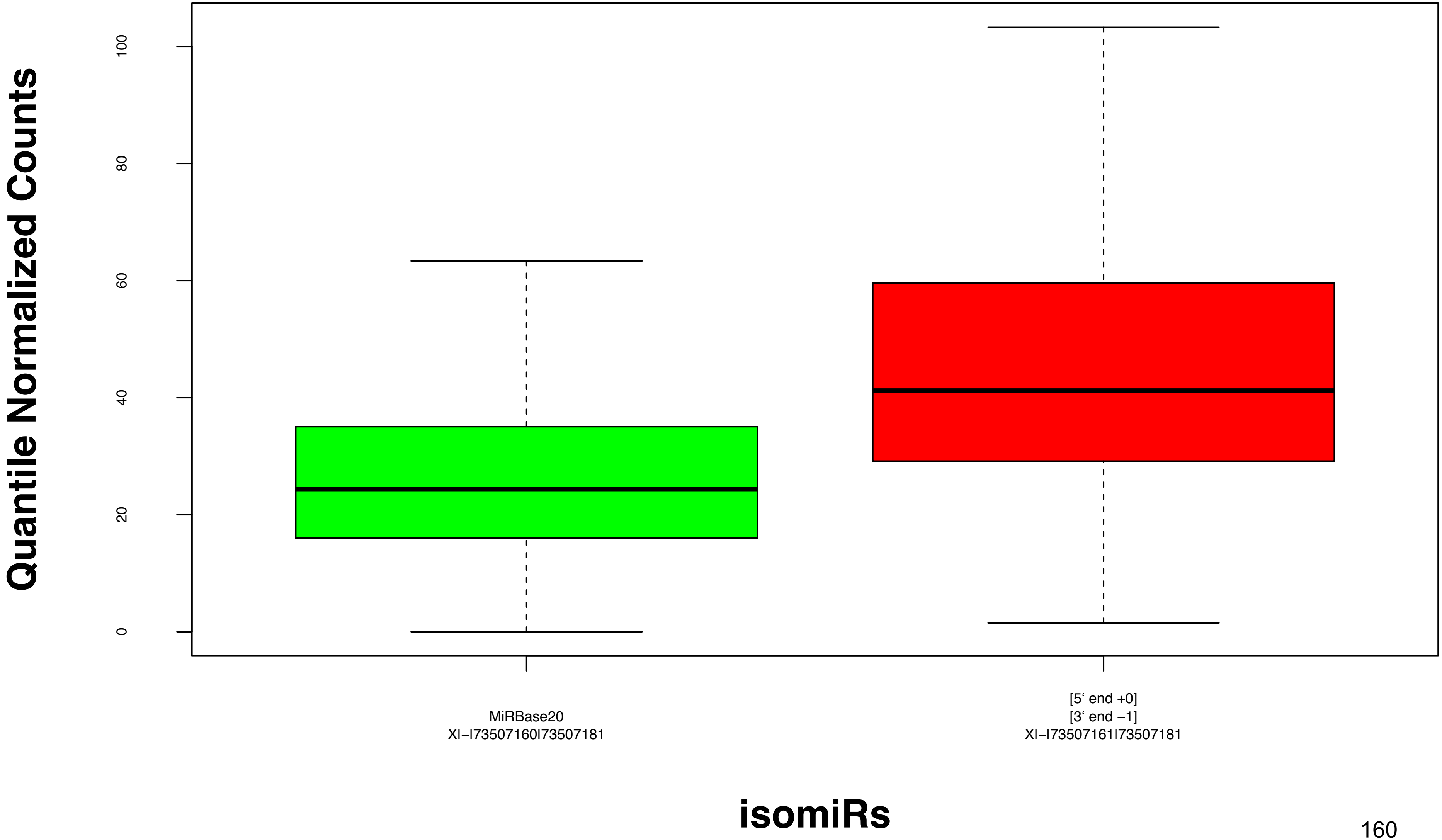

Supp. Figure 1
(pages 1 to 194)

MIMAT0019761=hsa−miR−4677−3p=MI0017308

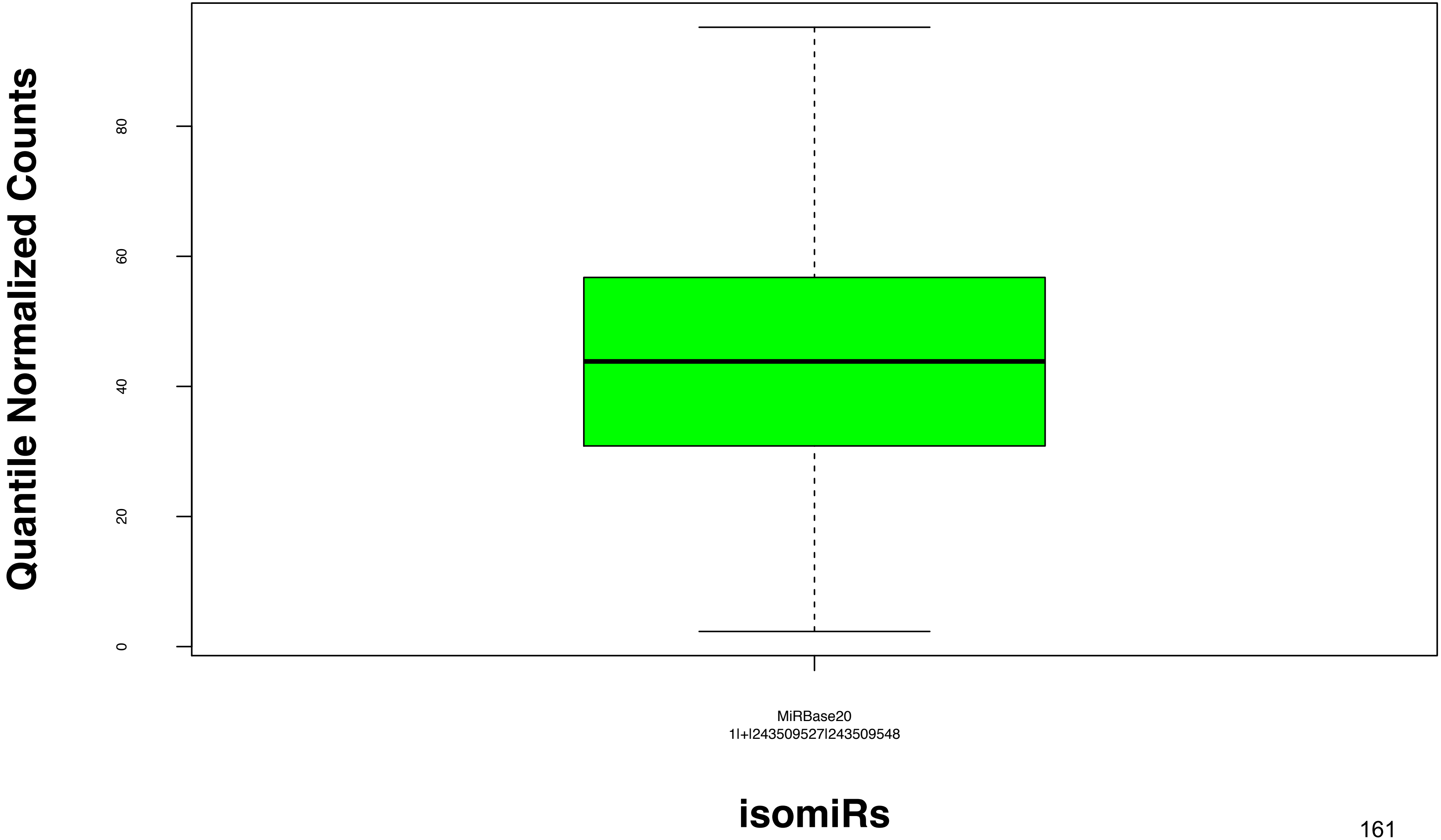

Supp. Figure 1
(pages 1 to 194)

MIMAT0000090=hsa−miR−32−5p=MI0000090

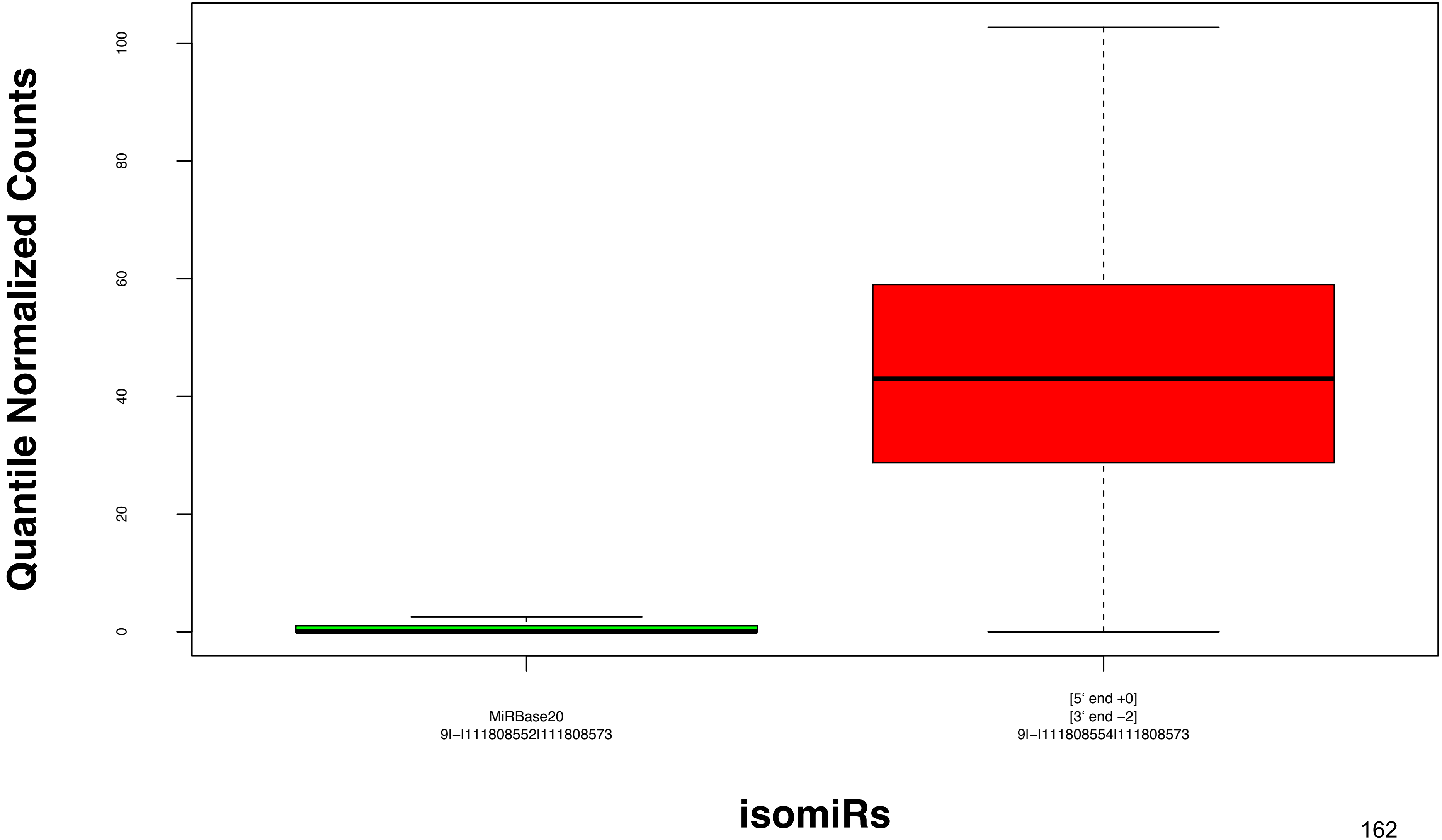

Supp. Figure 1
(pages 1 to 194)

MIMAT0000731=hsa–miR–378a–5p=MI0000786

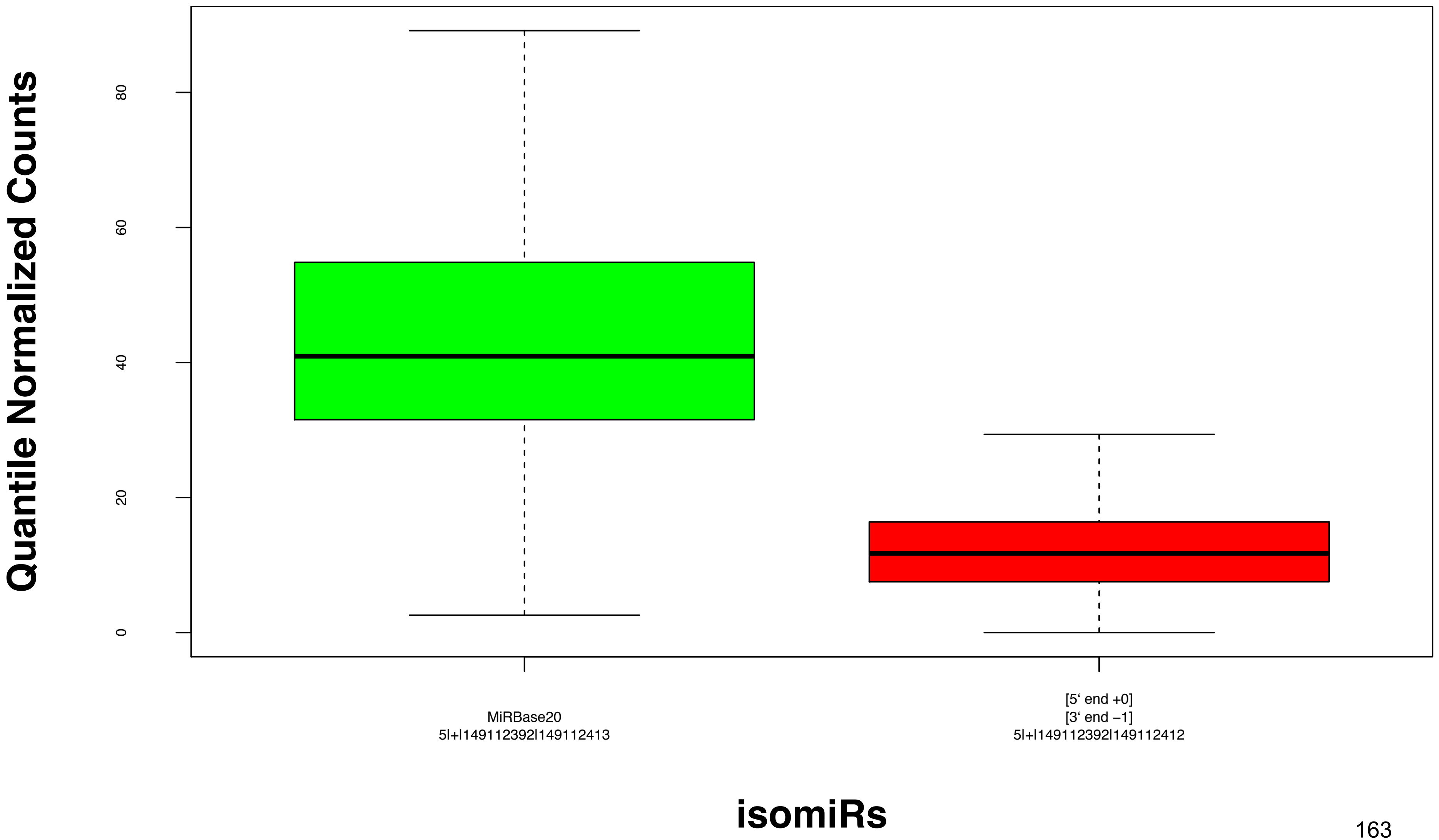

Supp. Figure 1
(pages 1 to 194)

MIMAT0000455=hsa−miR−185−5p=MI0000482

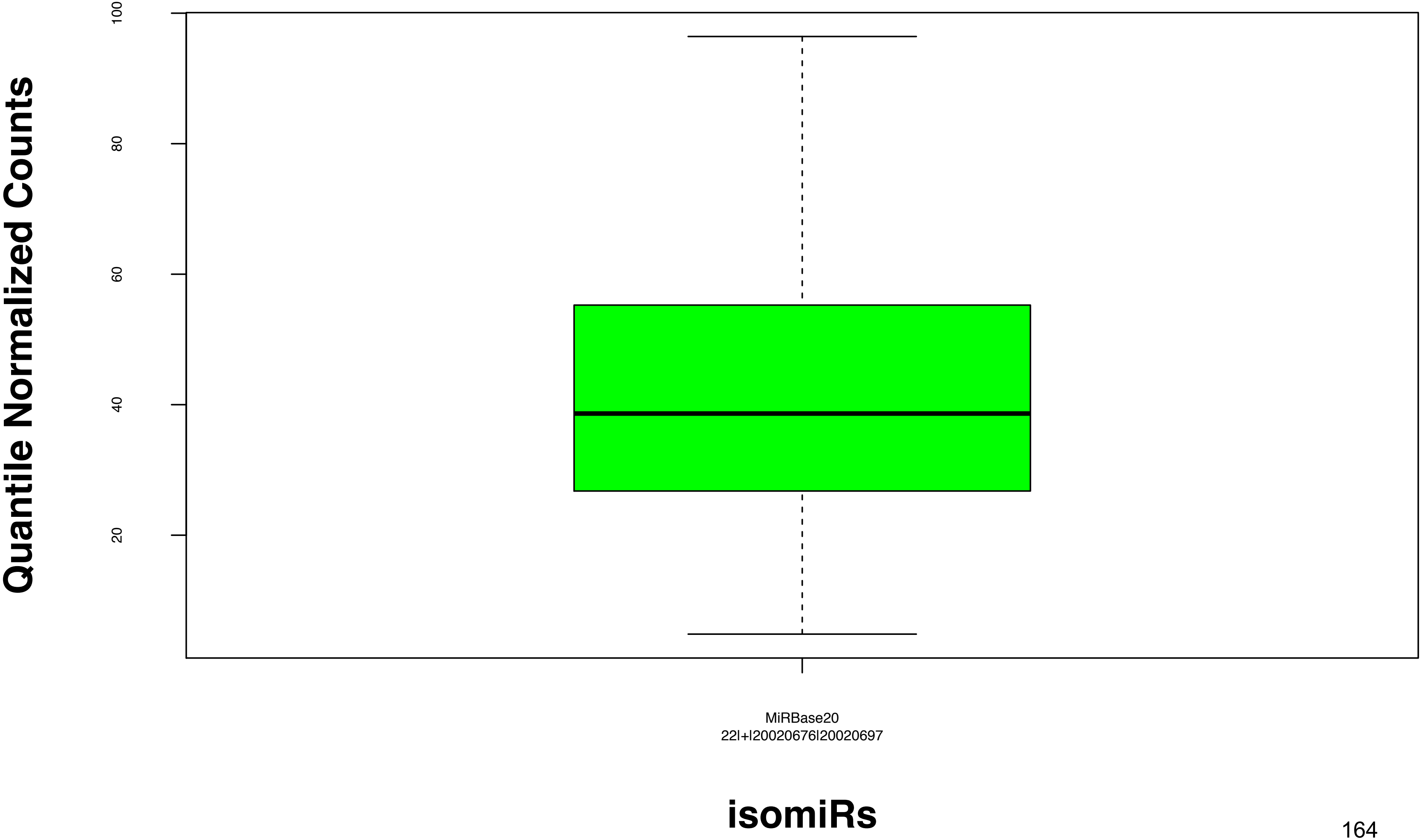

Supp. Figure 1
(pages 1 to 194)

MIMAT0004553=hsa−miR−7−1−3p=MI0000263

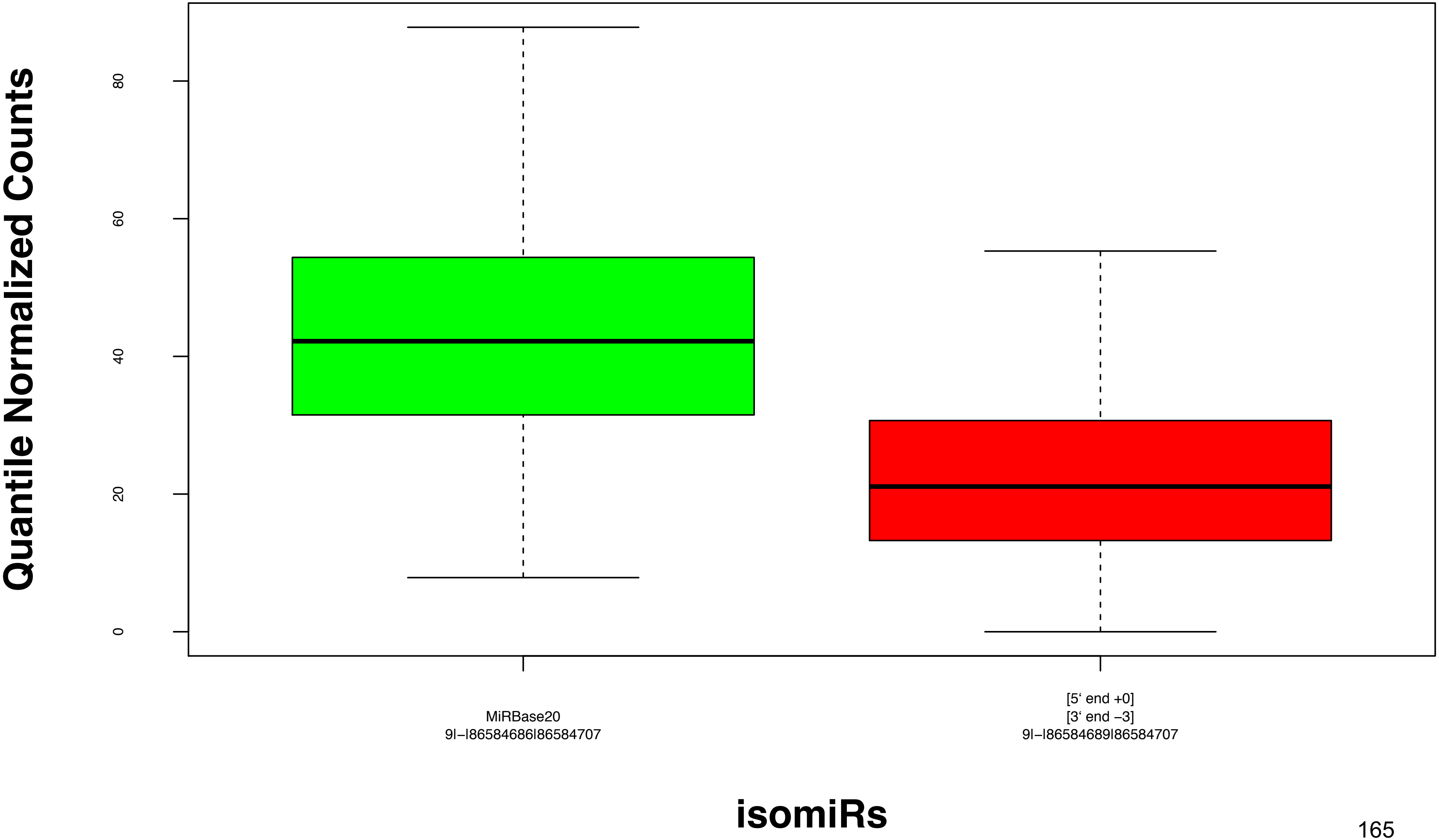

Supp. Figure 1
(pages 1 to 194)

MIMAT0004588=hsa−miR−27b−5p=MI0000440

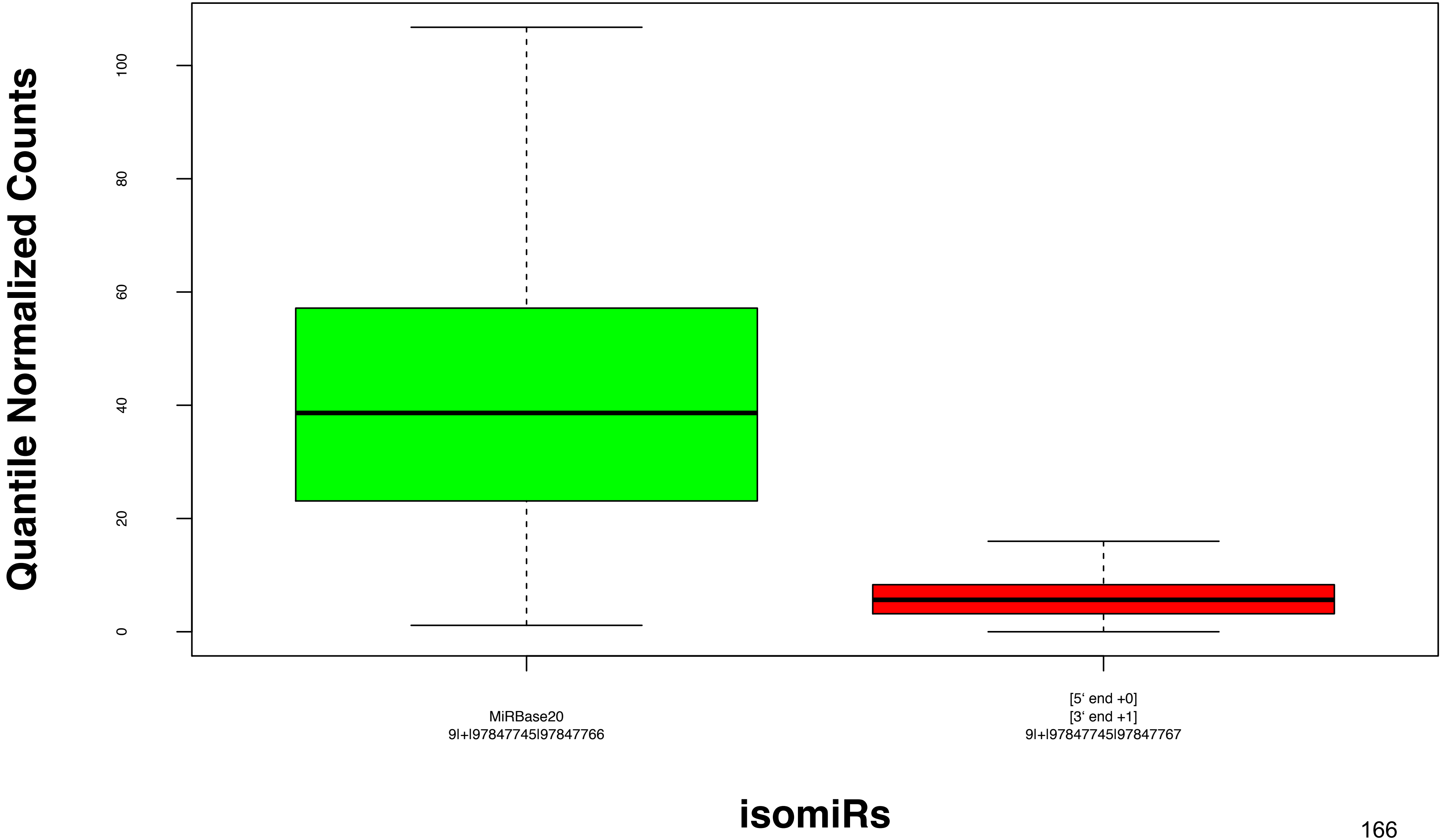

Supp. Figure 1
(pages 1 to 194)

MIMAT0030019=hsa−miR−7704=MI0025240

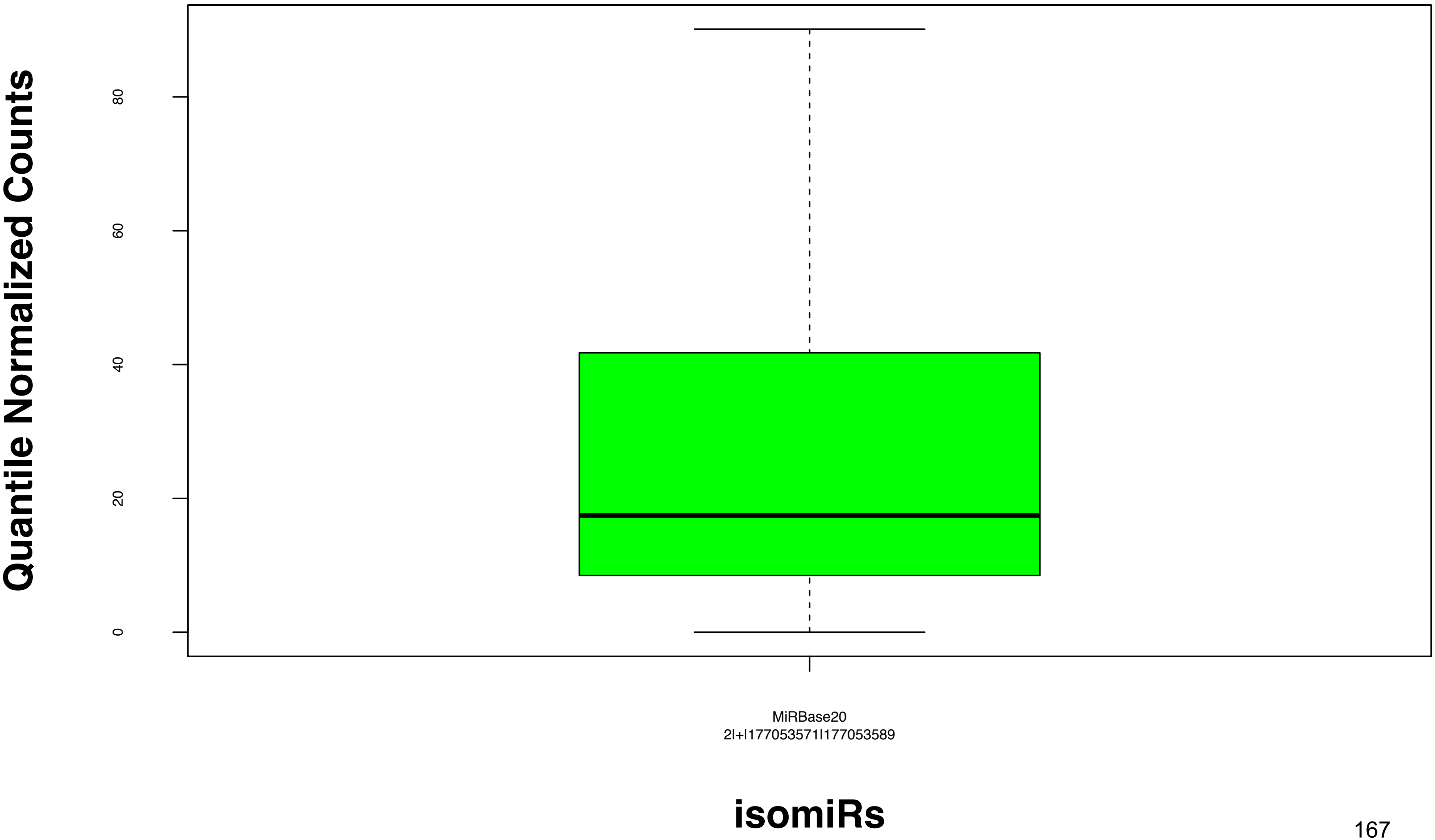

Supp. Figure 1
(pages 1 to 194)

MIMAT0004500=hsa−miR−26b−3p=MI0000084

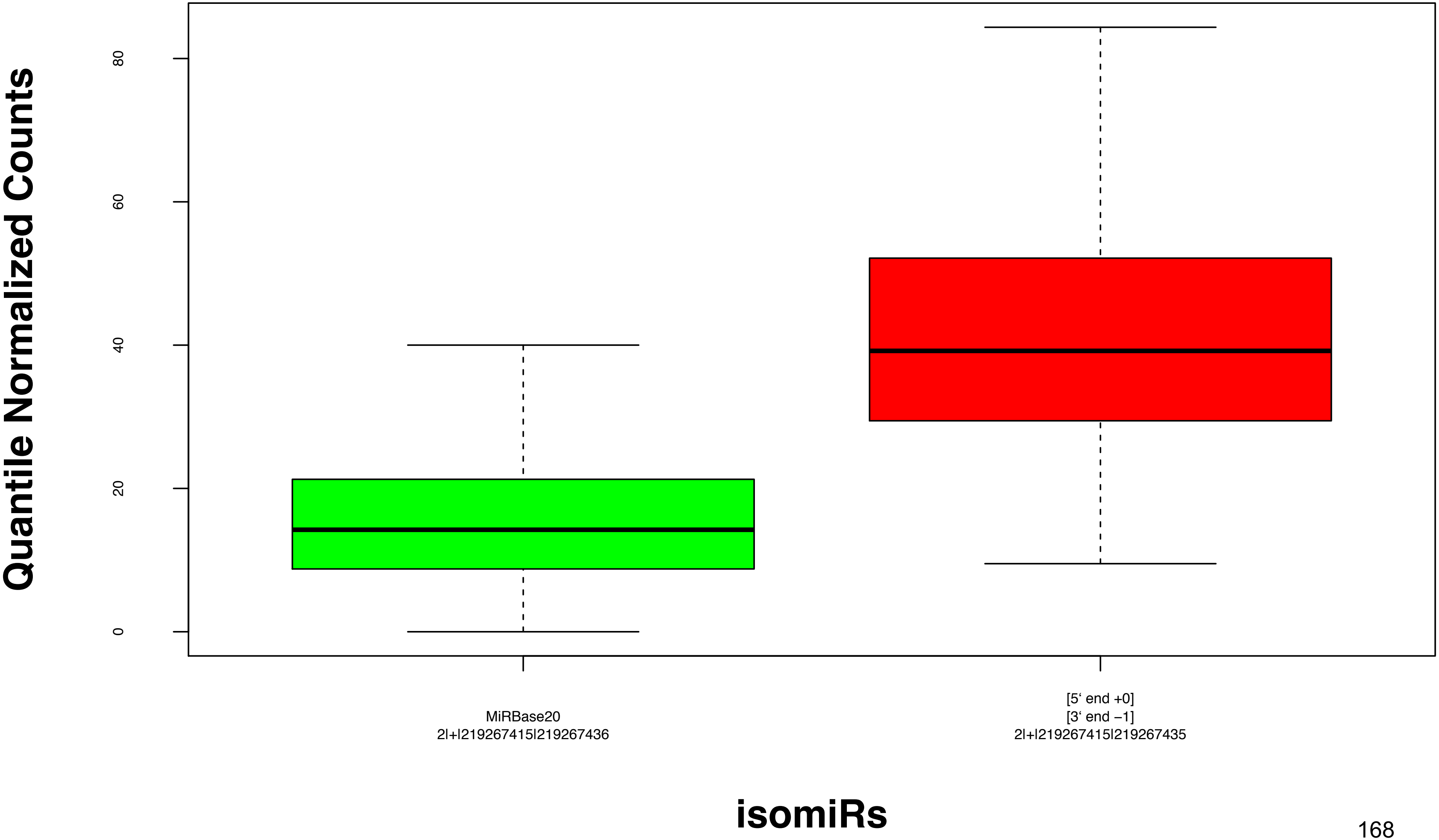

Supp. Figure 1
(pages 1 to 194)

MIMAT0004949=hsa−miR−877−5p=MI0005561

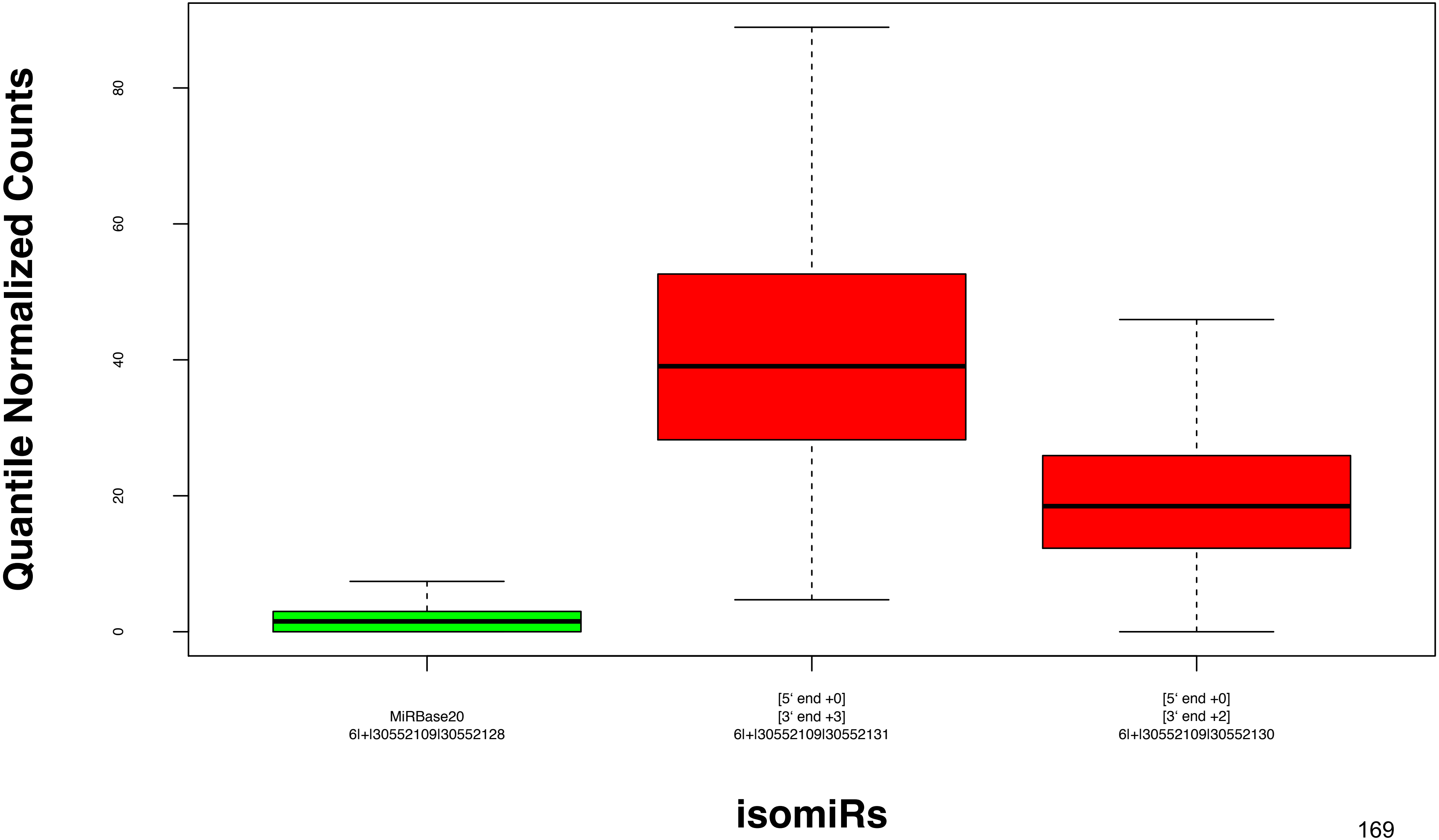

Supp. Figure 1
(pages 1 to 194)

MIMAT0000761=hsa−miR−324−5p=MI0000813

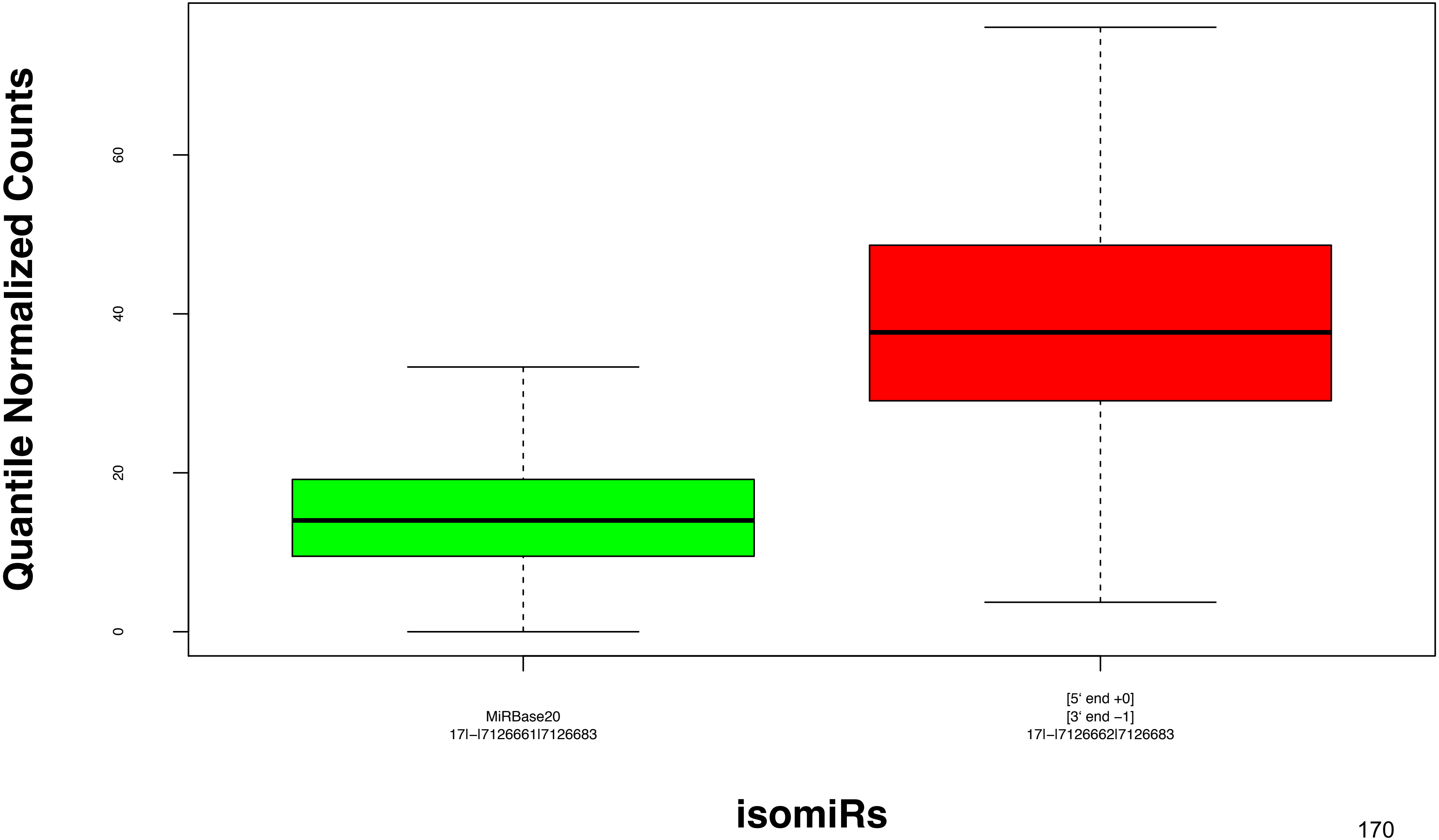

Supp. Figure 1
(pages 1 to 194)

MIMAT0004570=hsa−miR−223−5p=MI0000300

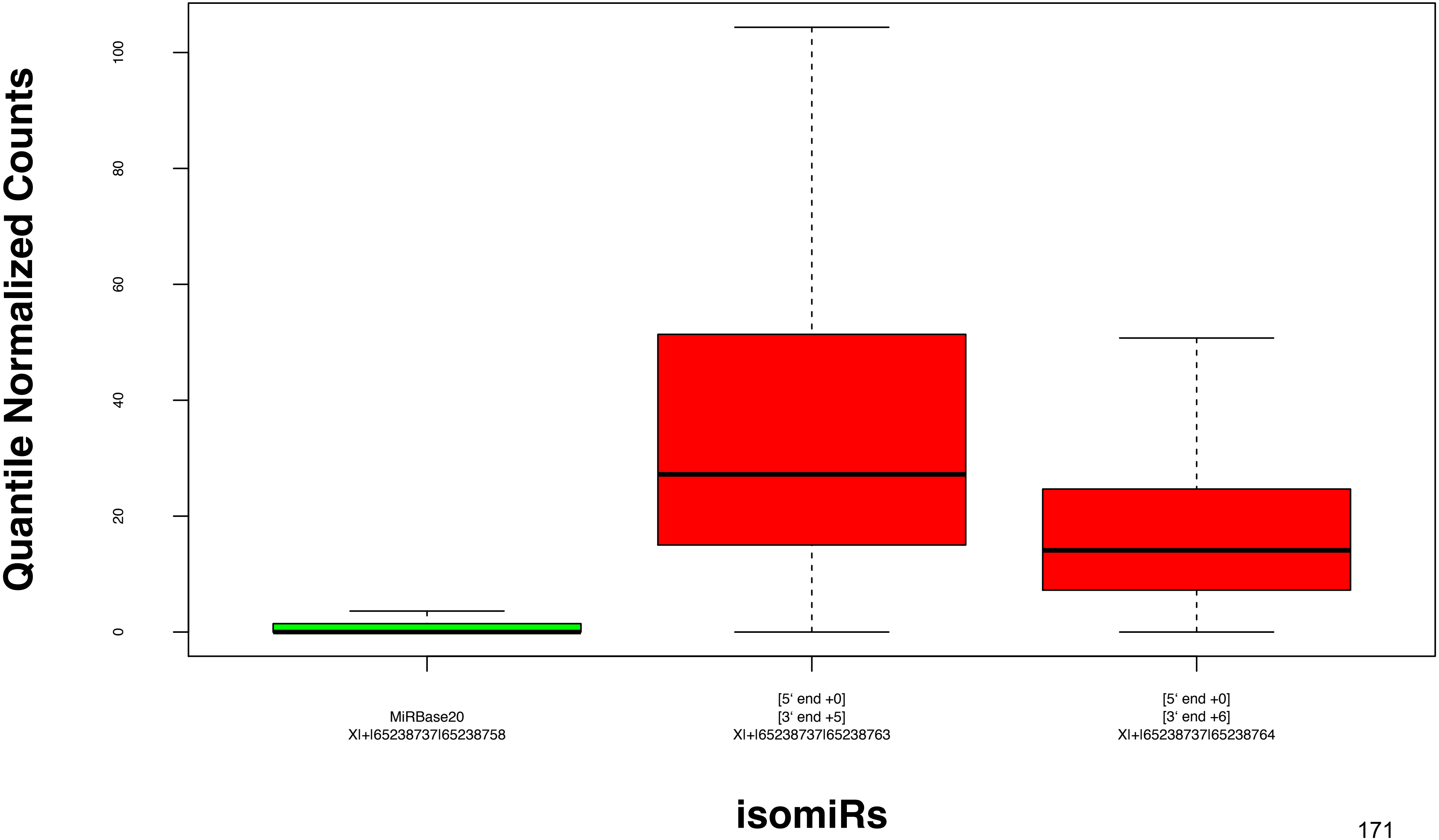

Supp. Figure 1
(pages 1 to 194)

MIMAT0004673=hsa−miR−29c−5p=MI0000735

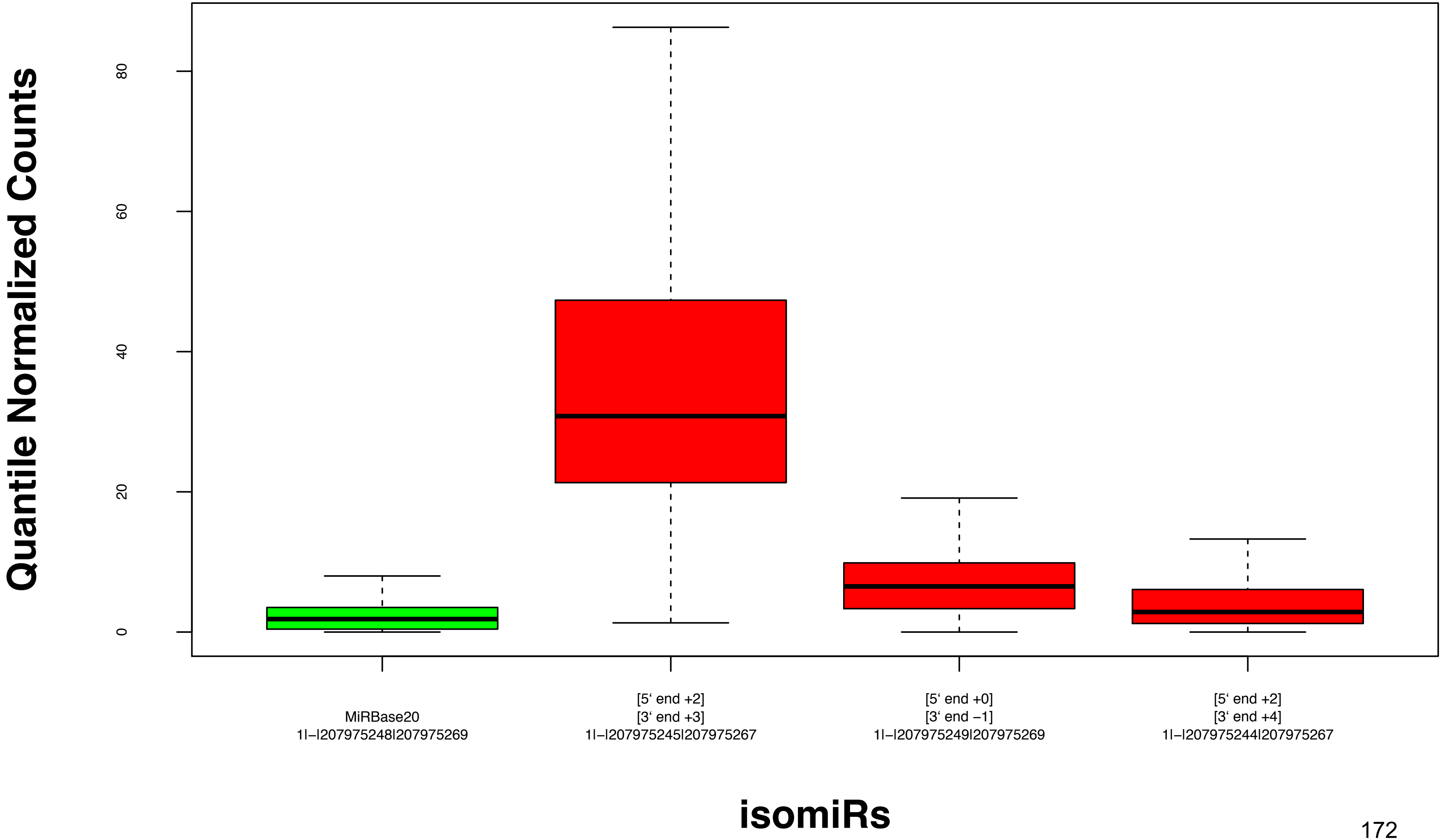

Supp. Figure 1
(pages 1 to 194)

MIMAT0000460_1=hsa−miR−194−5p=MI0000732

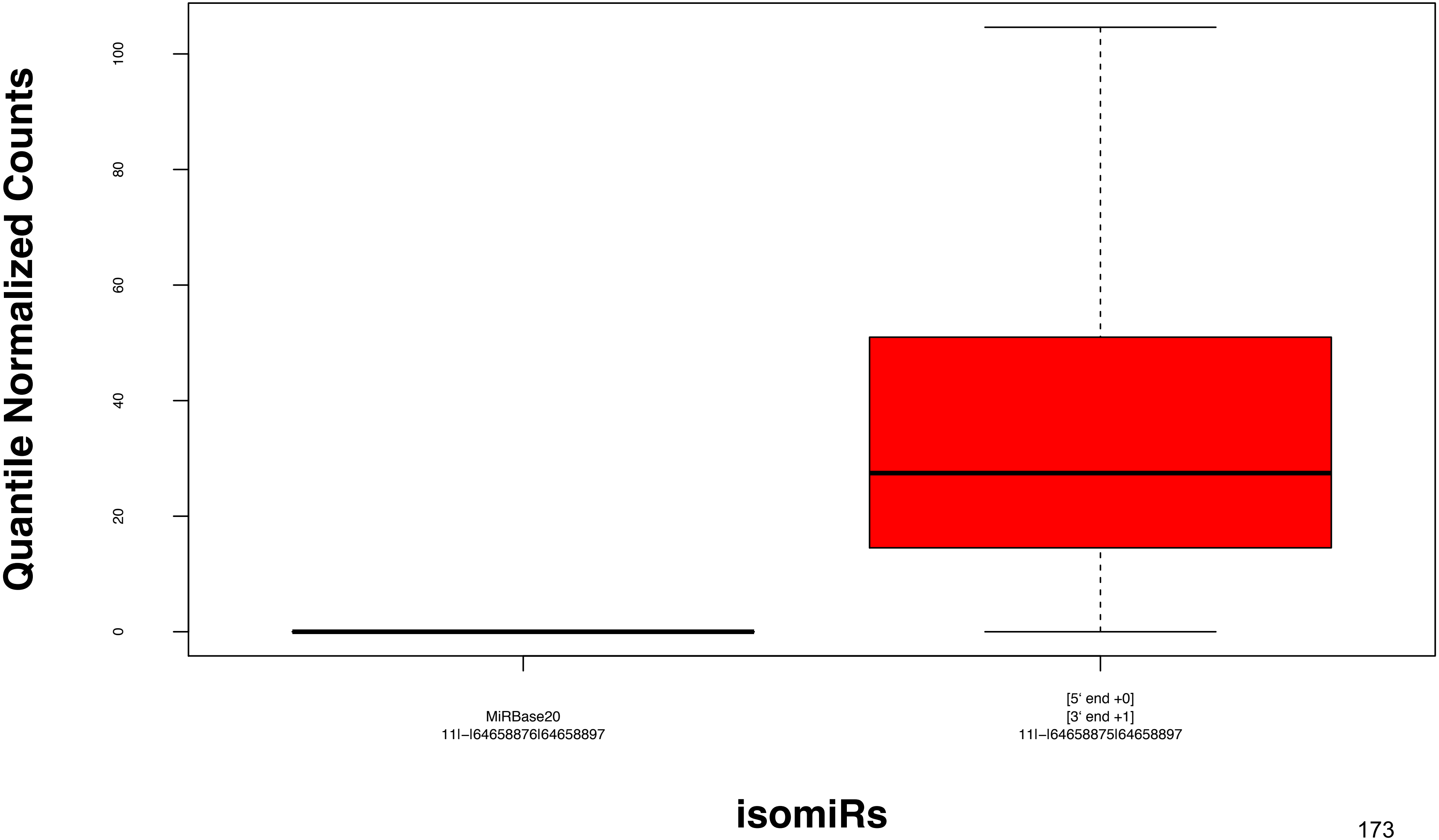

Supp. Figure 1
(pages 1 to 194)

MIMAT0003242=hsa−miR−577=MI0003584

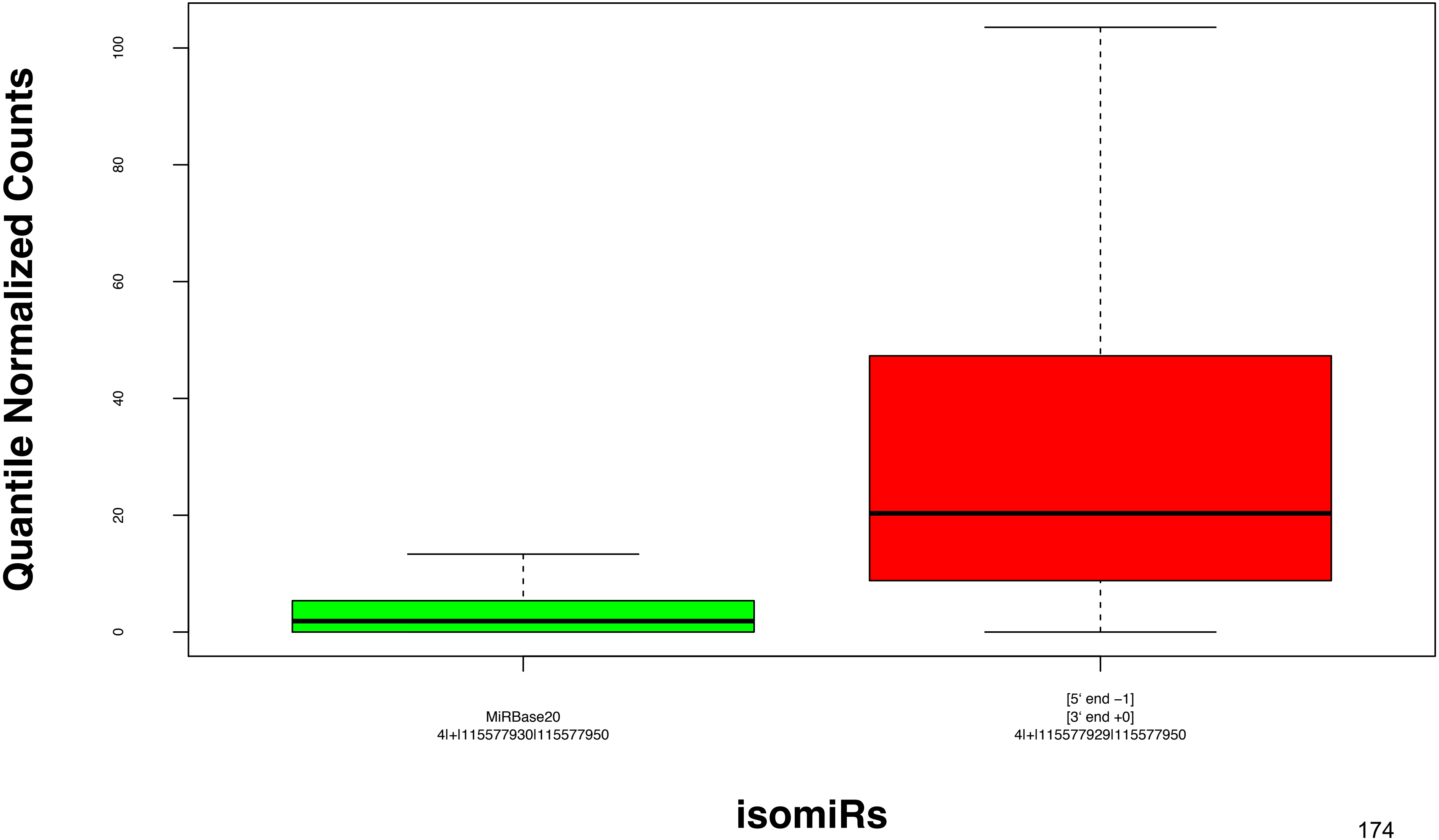

Supp. Figure 1
(pages 1 to 194)

MIMAT0003884=hsa−miR−454−5p=MI0003820

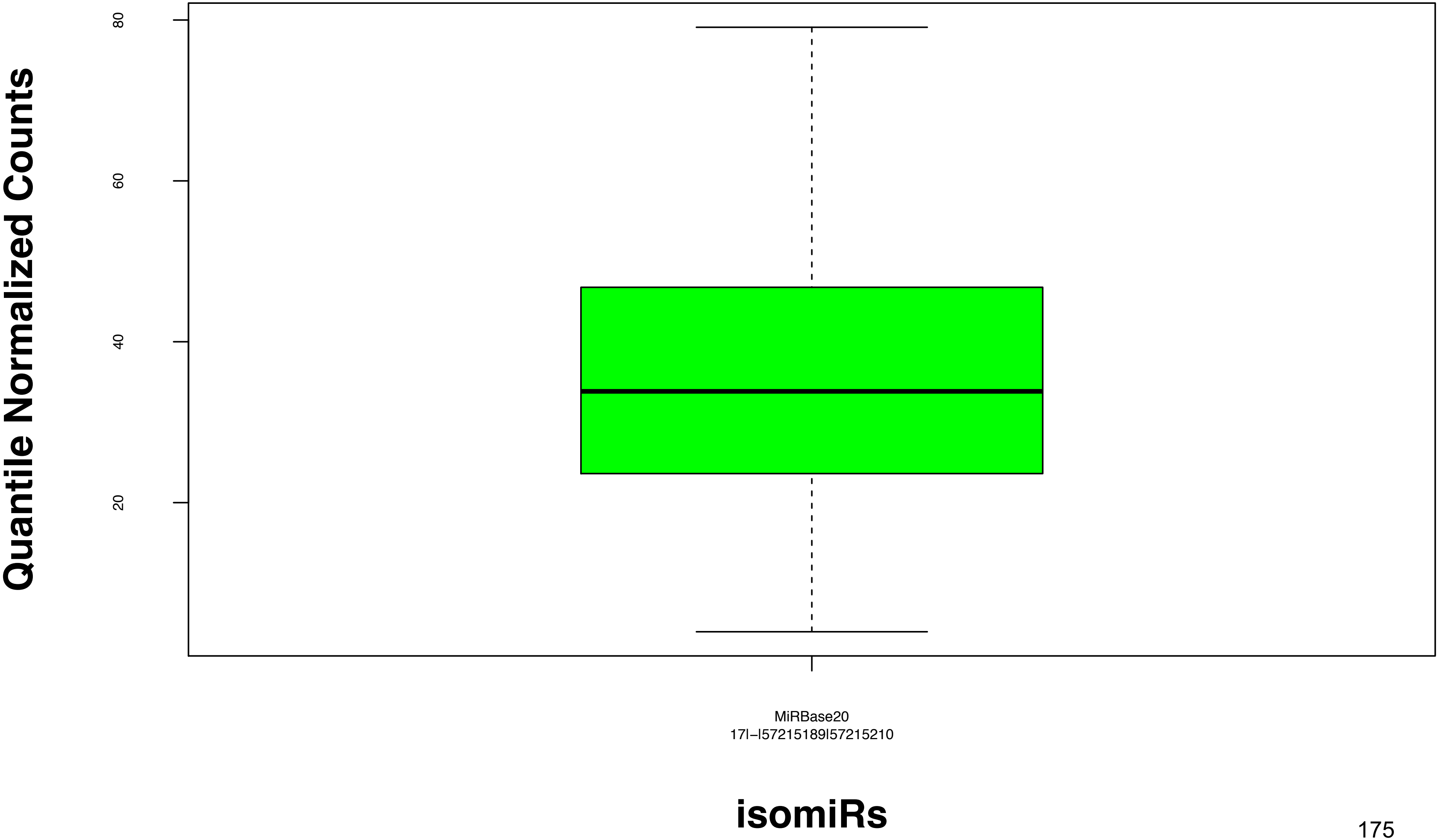

Supp. Figure 1
(pages 1 to 194)

MIMAT0003266=hsa−miR−598−3p=MI0003610

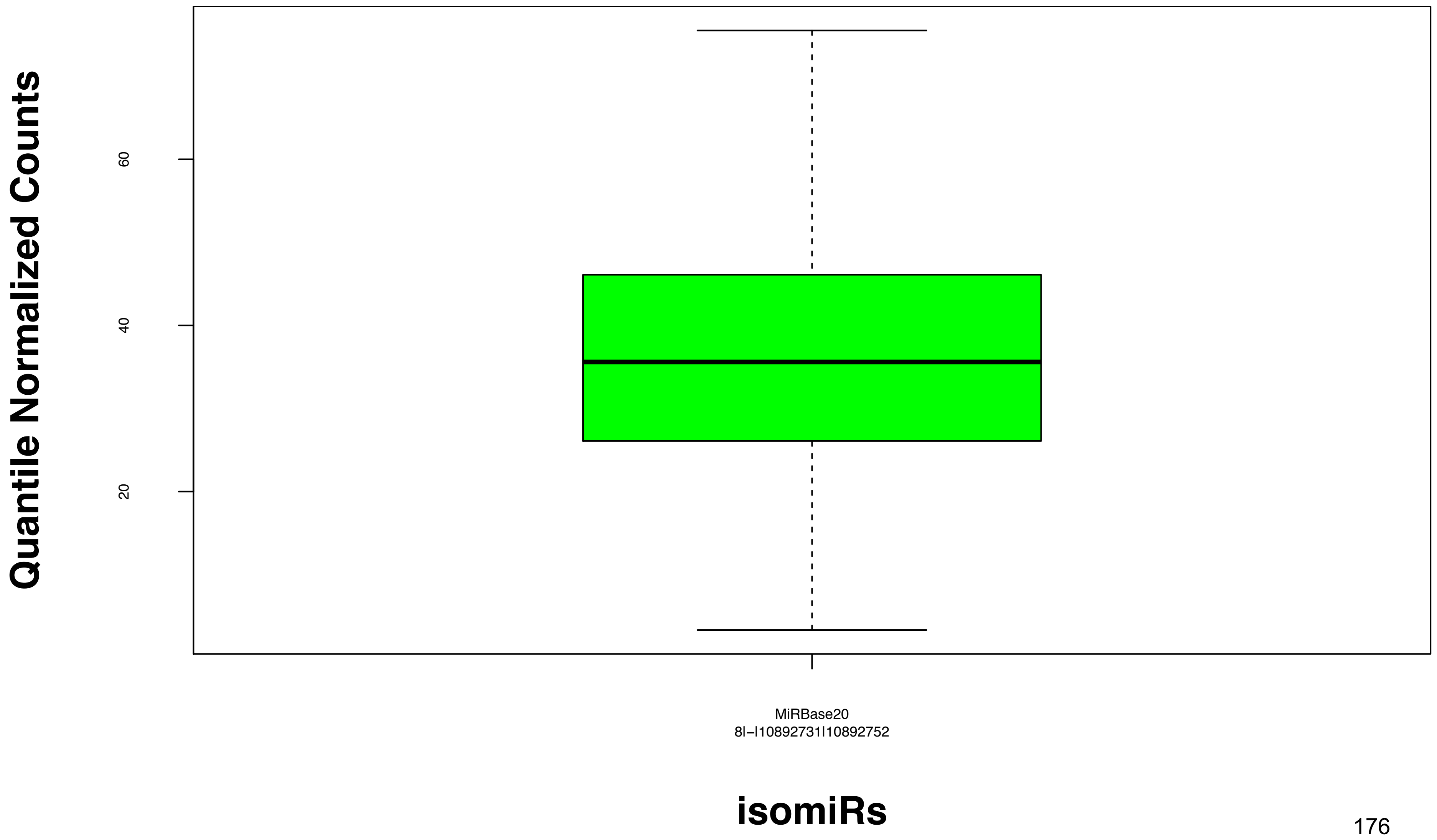

Supp. Figure 1
(pages 1 to 194)

MIMAT0000097=hsa−miR−99a−5p=MI0000101

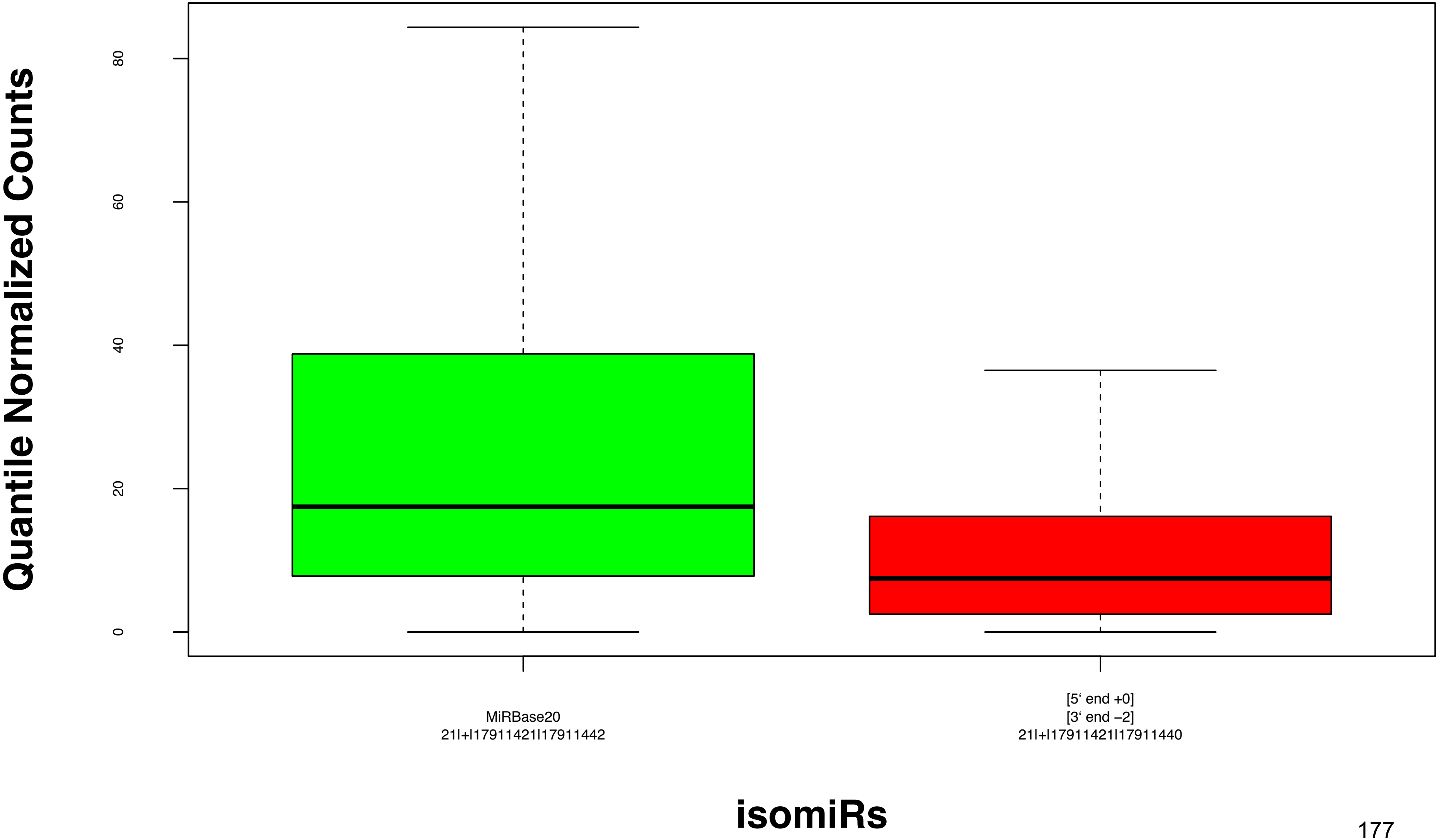

Supp. Figure 1
(pages 1 to 194)

MIMAT0017992=hsa−miR−3614−5p=MI0016004

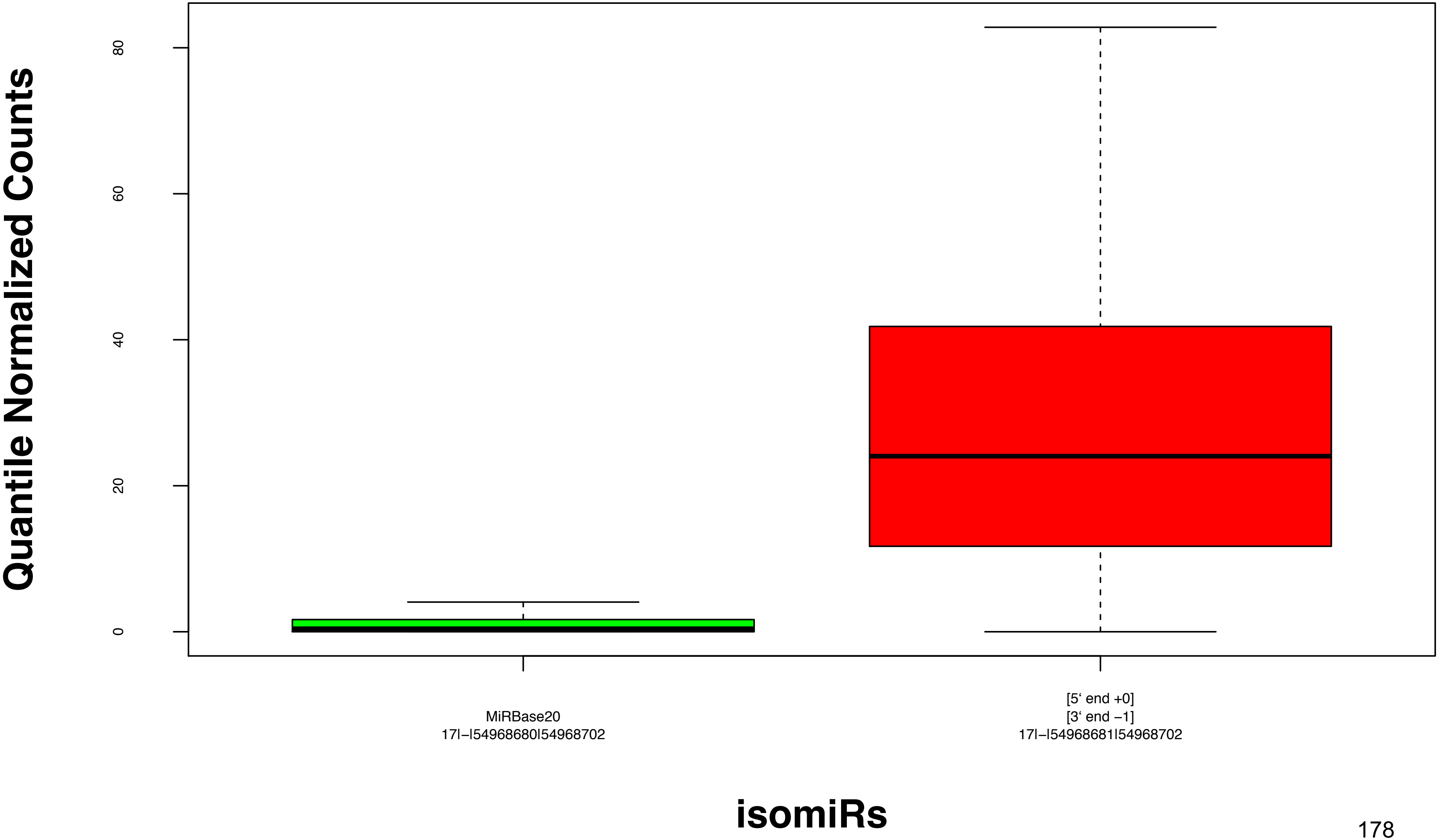

Supp. Figure 1
(pages 1 to 194)

MIMAT0004589=hsa−miR−30b−3p=MI0000441

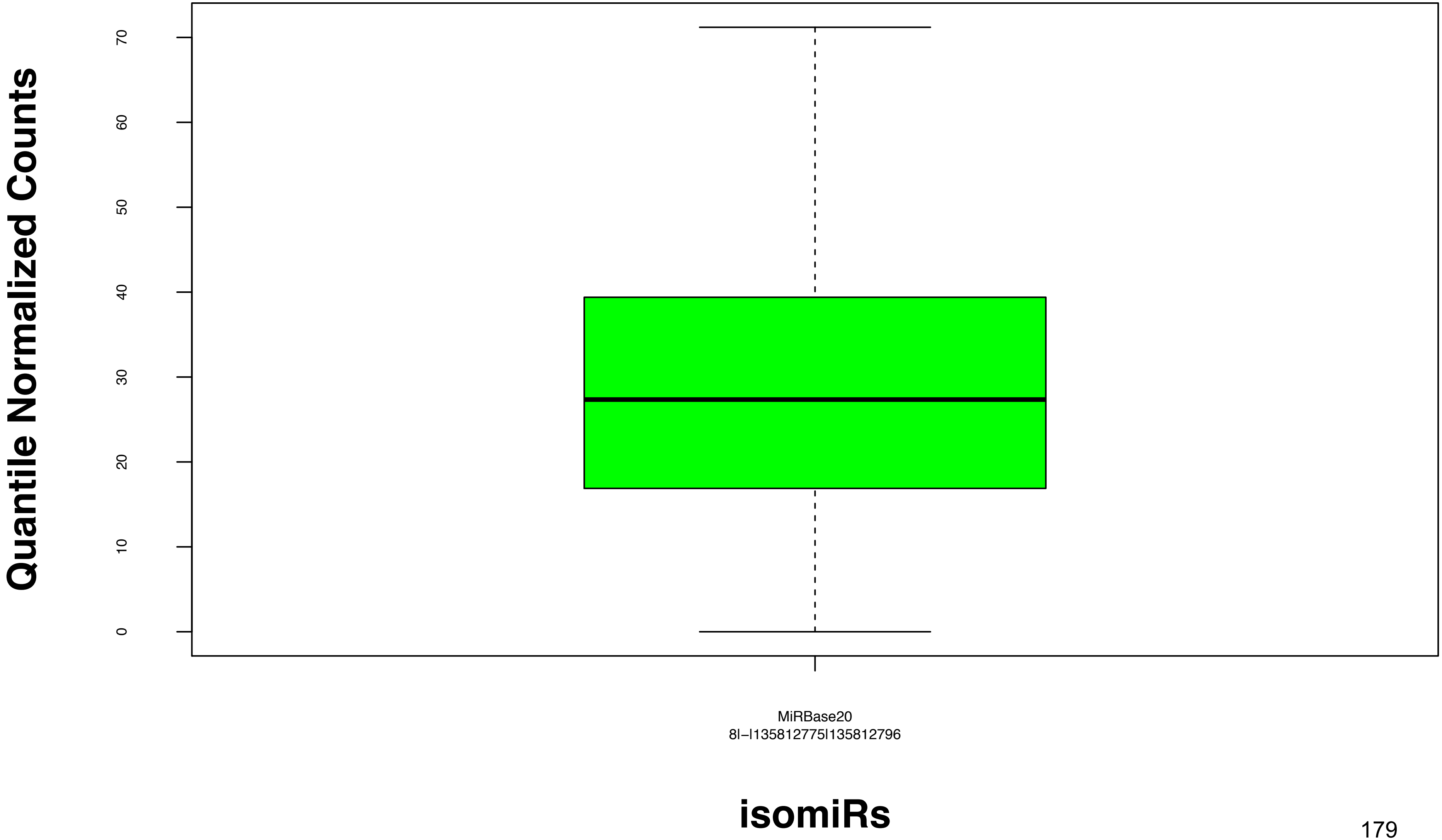

Supp. Figure 1
(pages 1 to 194)

MIMAT0009199=hsa–miR–365a–5p=MI0000767

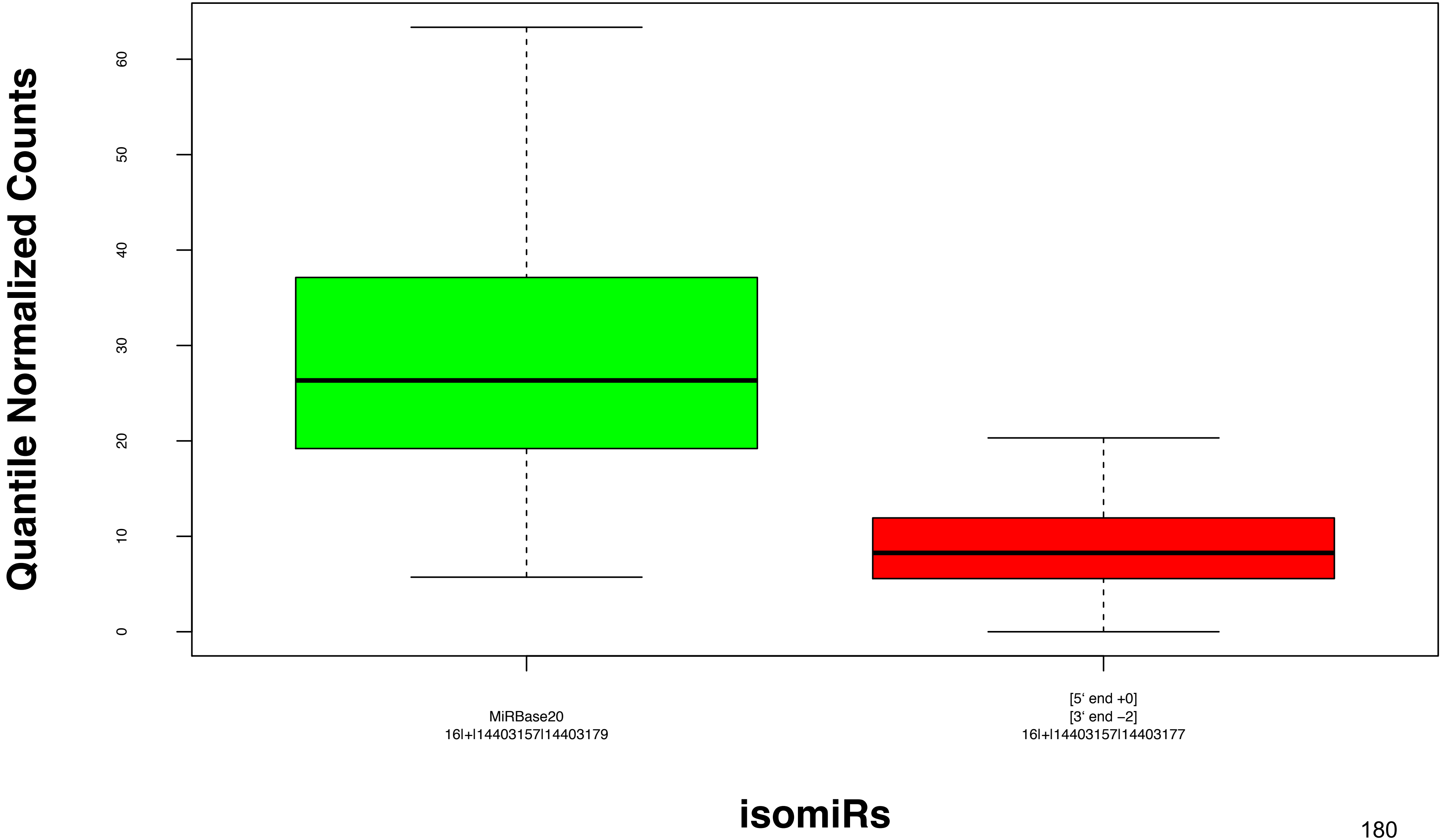

Supp. Figure 1
(pages 1 to 194)

MIMAT0000280=hsa−miR−223−3p=MI0000300

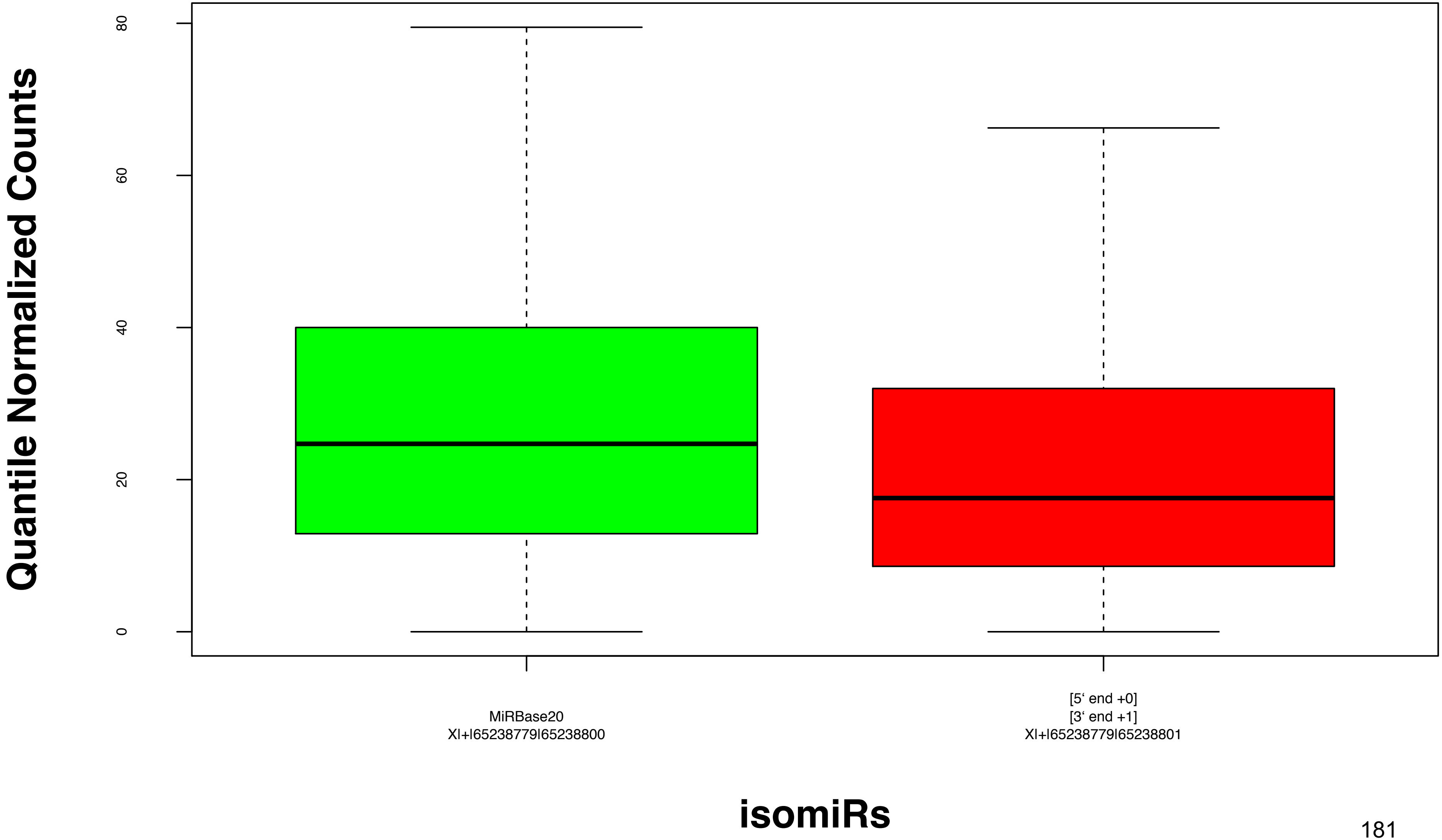

Supp. Figure 1
(pages 1 to 194)

MIMAT0005825=hsa−miR−1180−3p=MI0006273

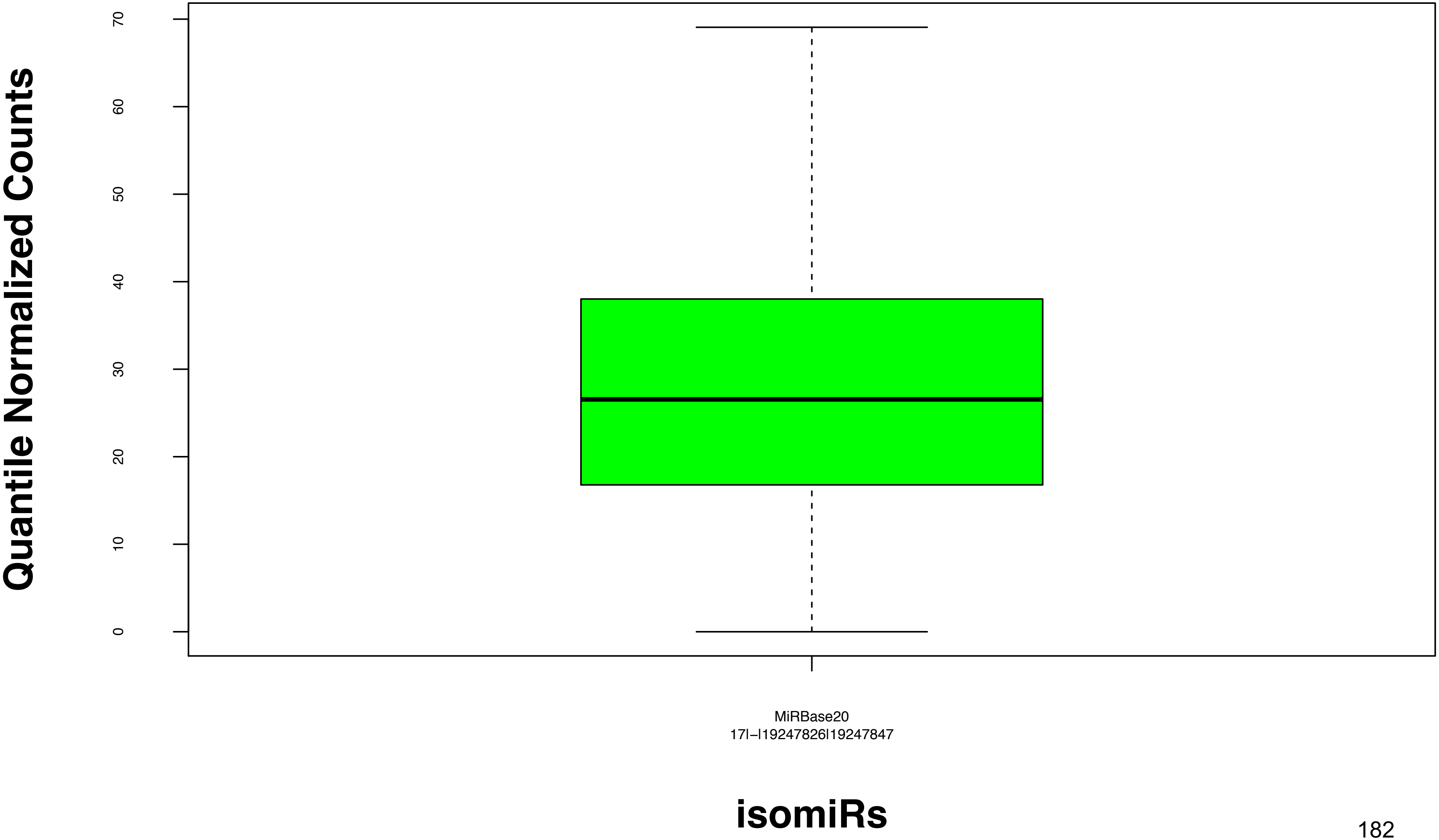

Supp. Figure 1
(pages 1 to 194)

MIMAT0004509=hsa–miR–93–3p=MI0000095

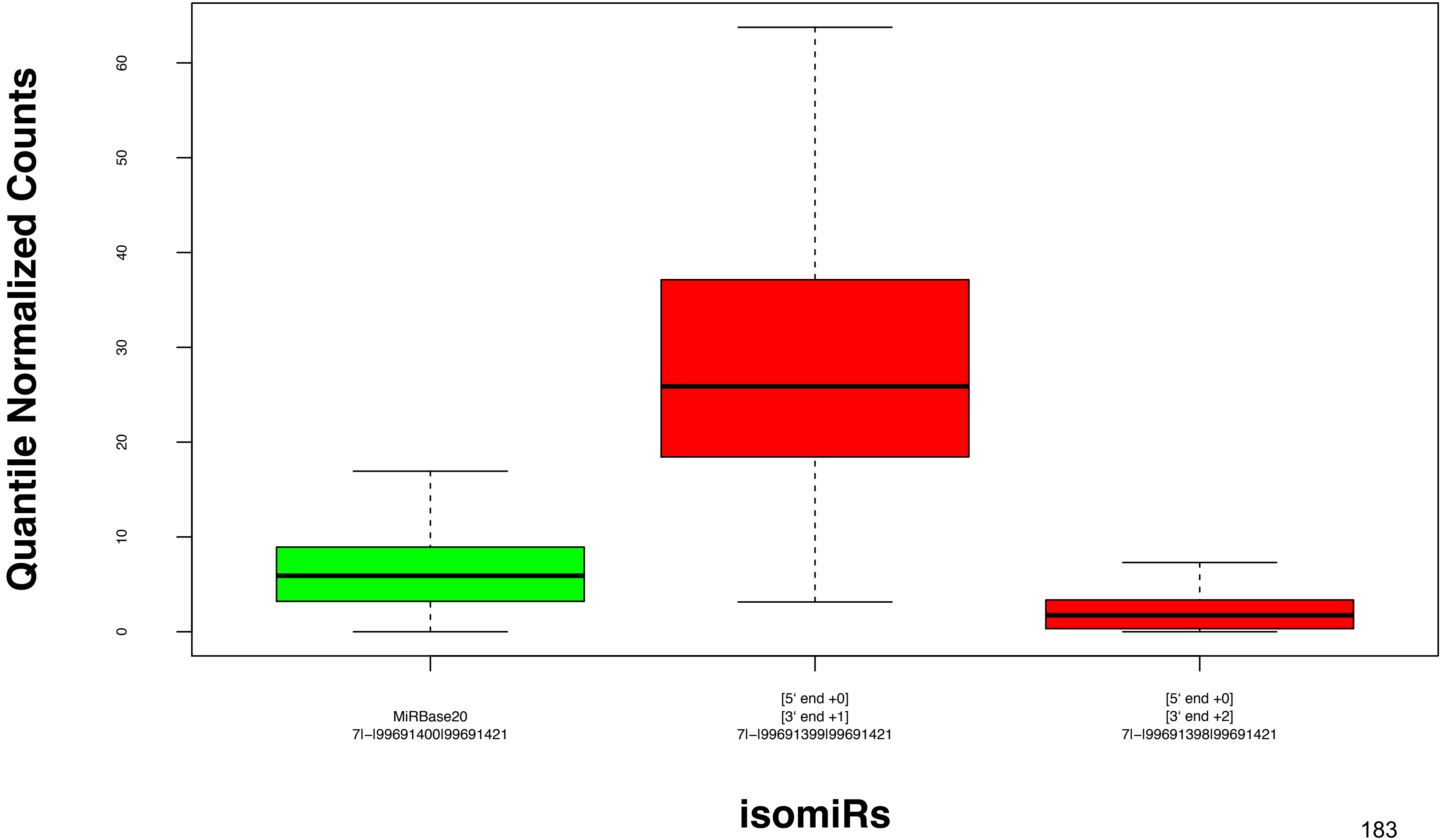

Supp. Figure 1
(pages 1 to 194)

MIMAT0019058=hsa−miR−4521=MI0016887

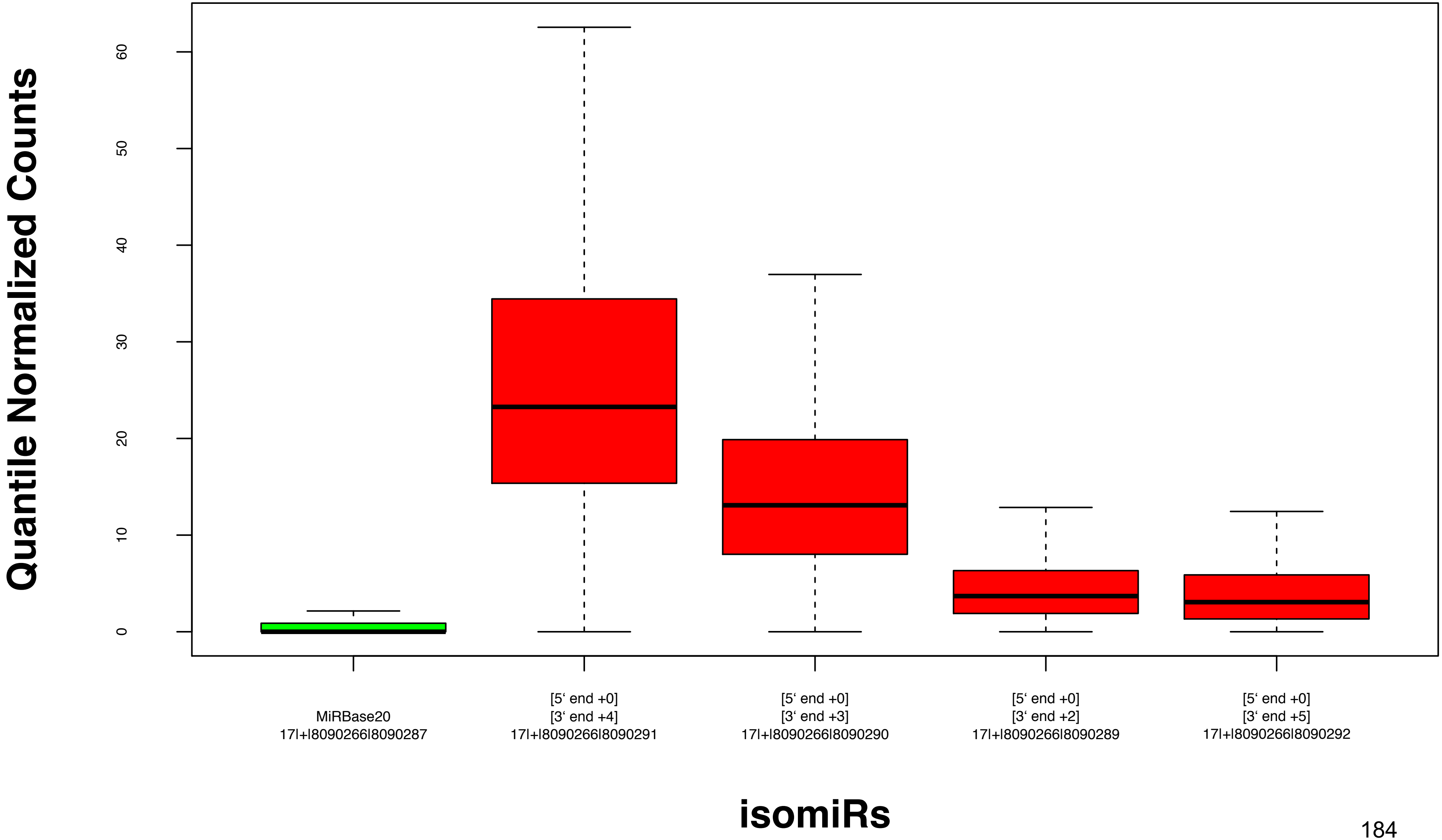

Supp. Figure 1
(pages 1 to 194)

MIMAT0005797=hsa–miR–1301–3p=MI0003815

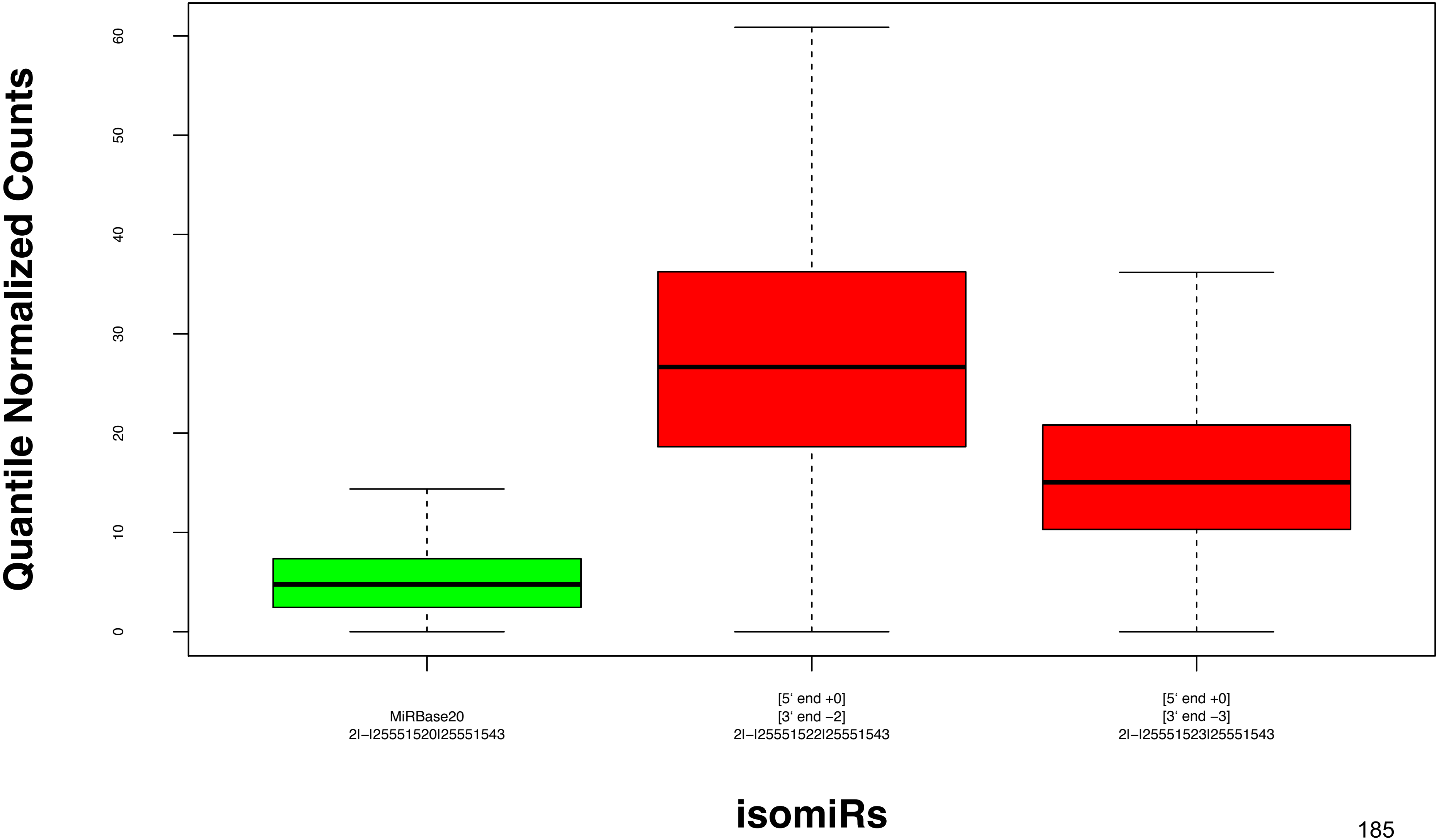

Supp. Figure 1
(pages 1 to 194)

MIMAT0004797=hsa−miR−582−3p=MI0003589

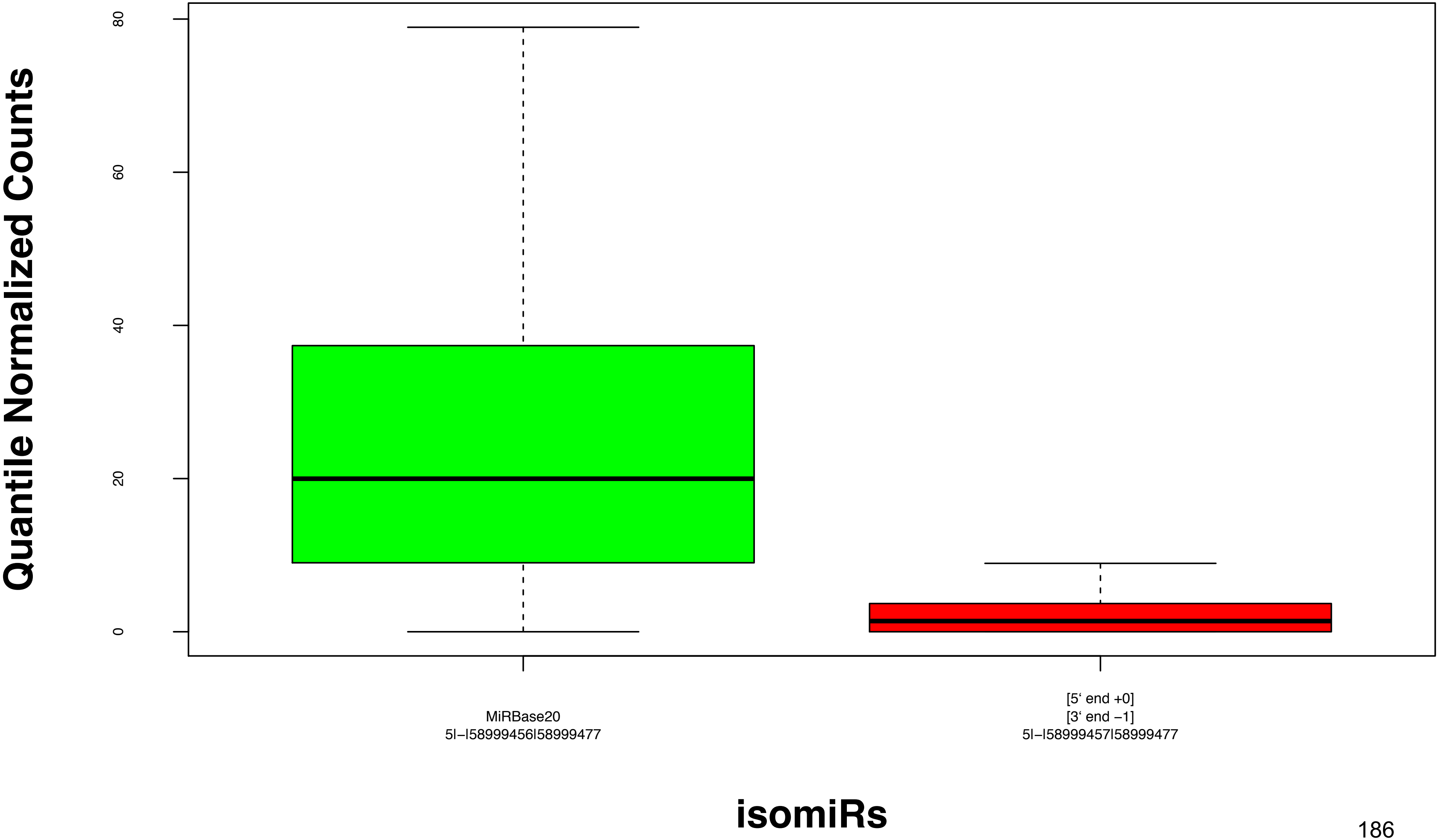

Supp. Figure 1
(pages 1 to 194)

MIMAT0004486=hsa−let−7f−1−3p=MI0000067

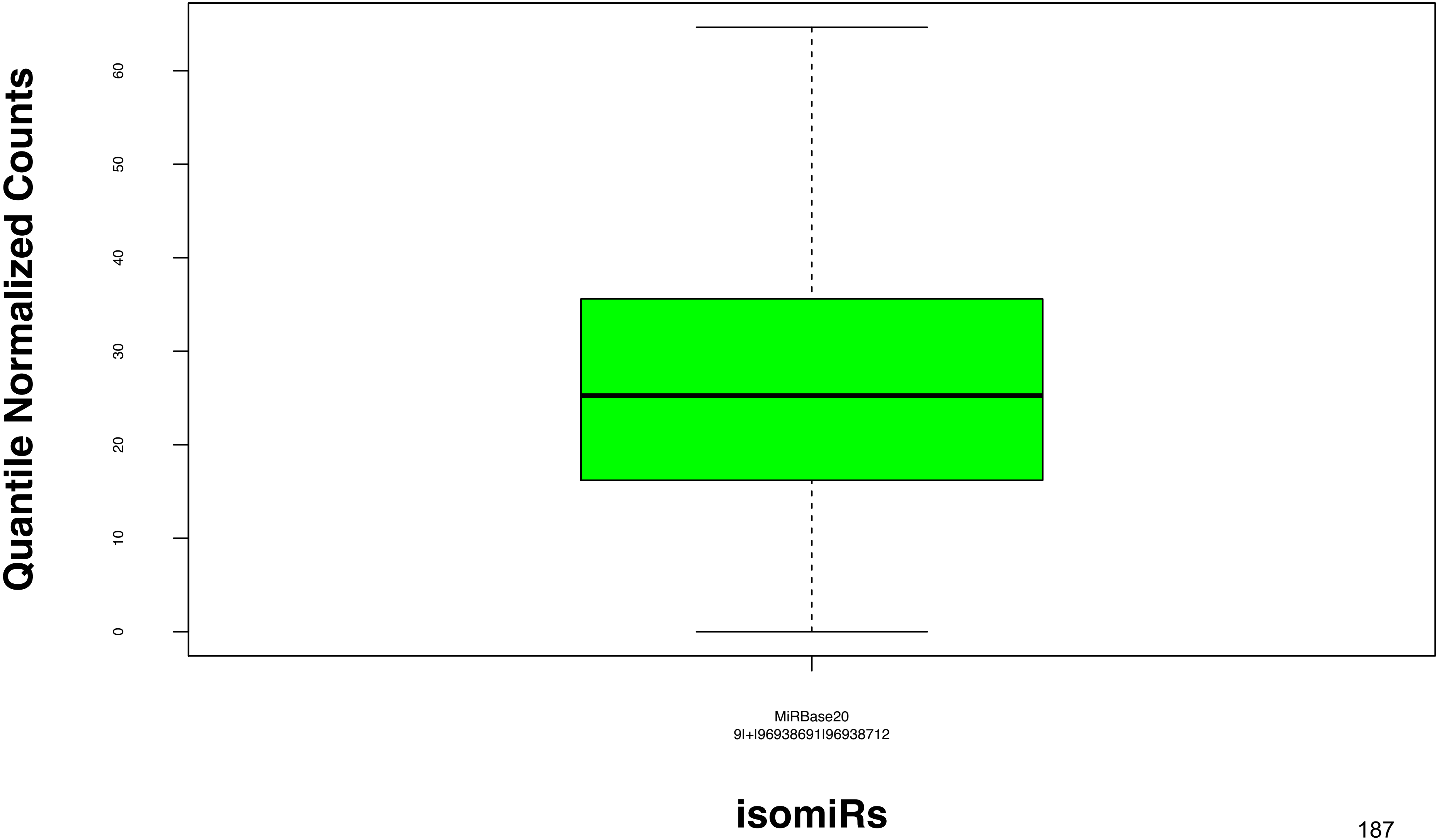

Supp. Figure 1
(pages 1 to 194)

MIMAT0004607=hsa−miR−138−1−3p=MI0000476

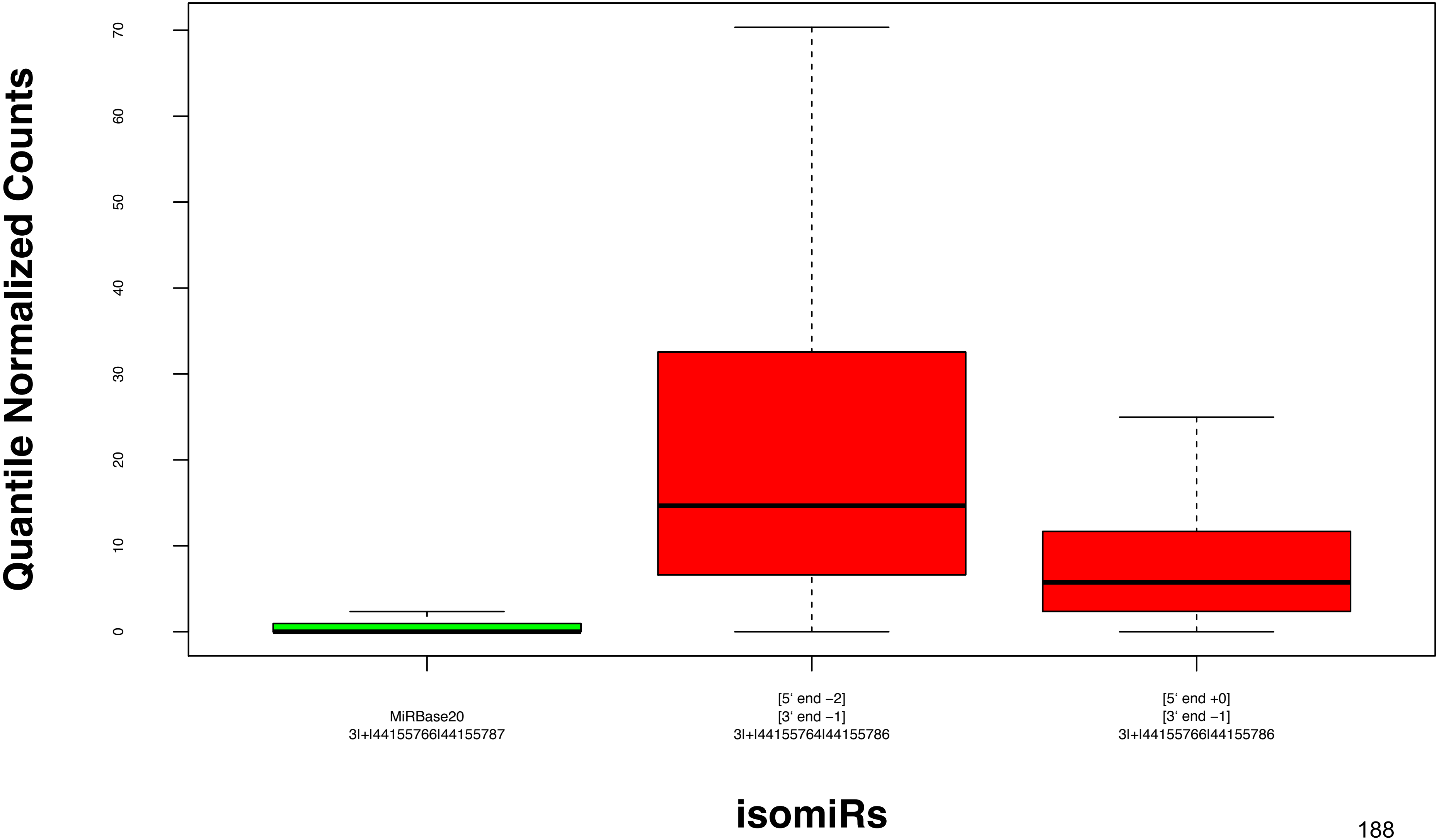

Supp. Figure 1
(pages 1 to 194)

MIMAT0000069_1=hsa–miR–16–5p=MI0000115

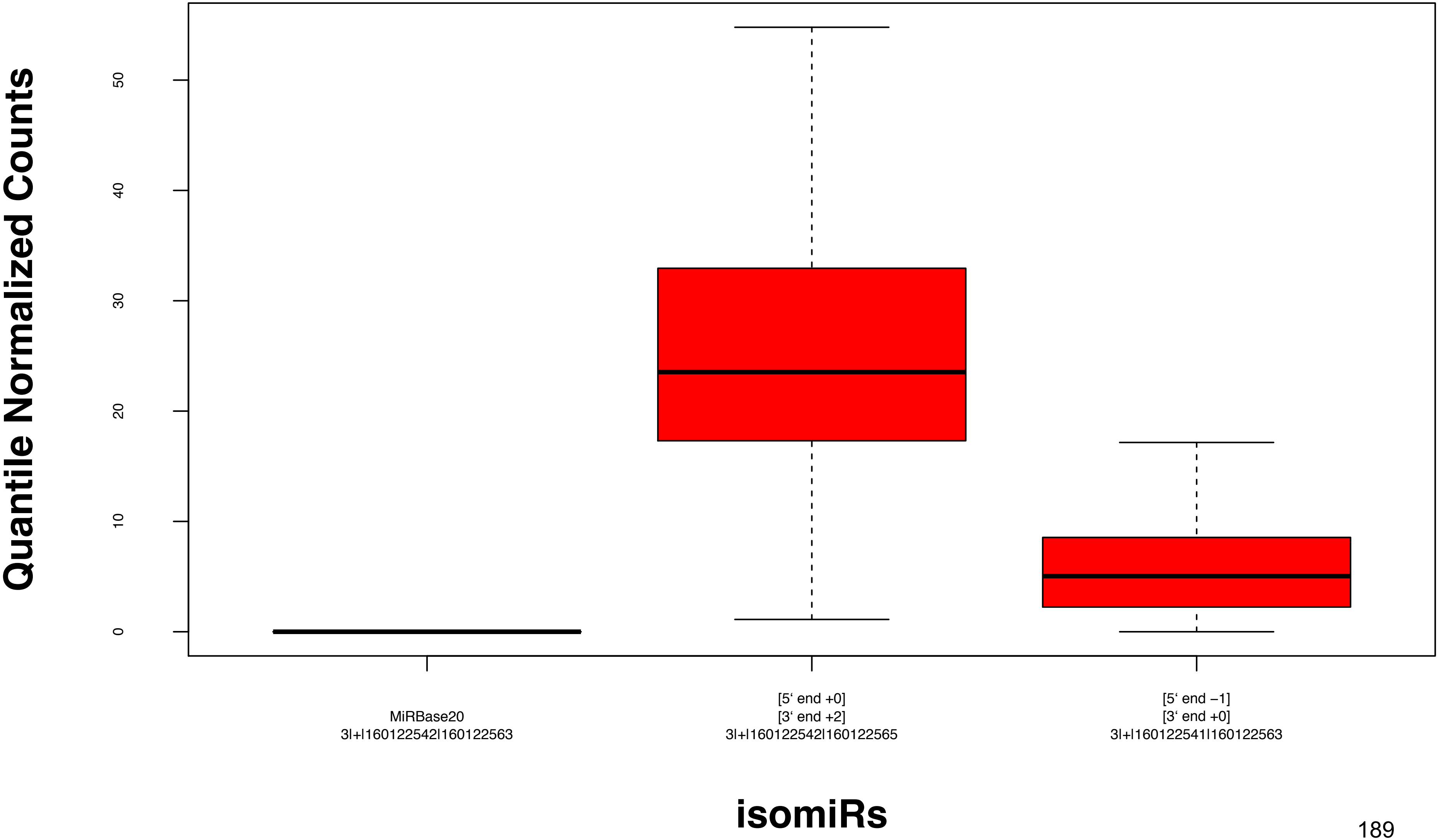

Supp. Figure 1
(pages 1 to 194)

MIMAT0000690=hsa−miR−296−5p=MI0000747

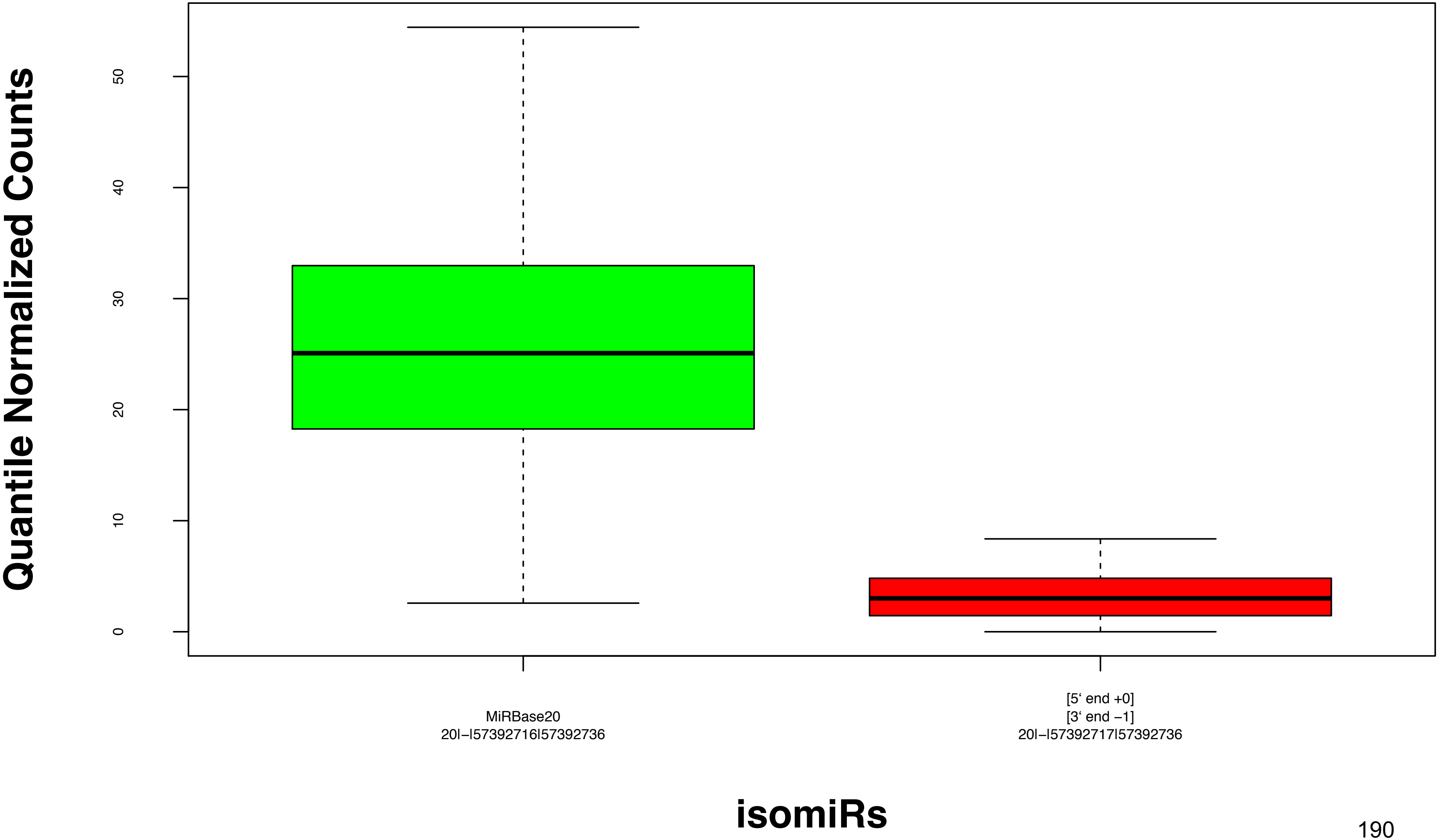

Supp. Figure 1
(pages 1 to 194)

MIMAT0019880=hsa−miR−4746−5p=MI0017385

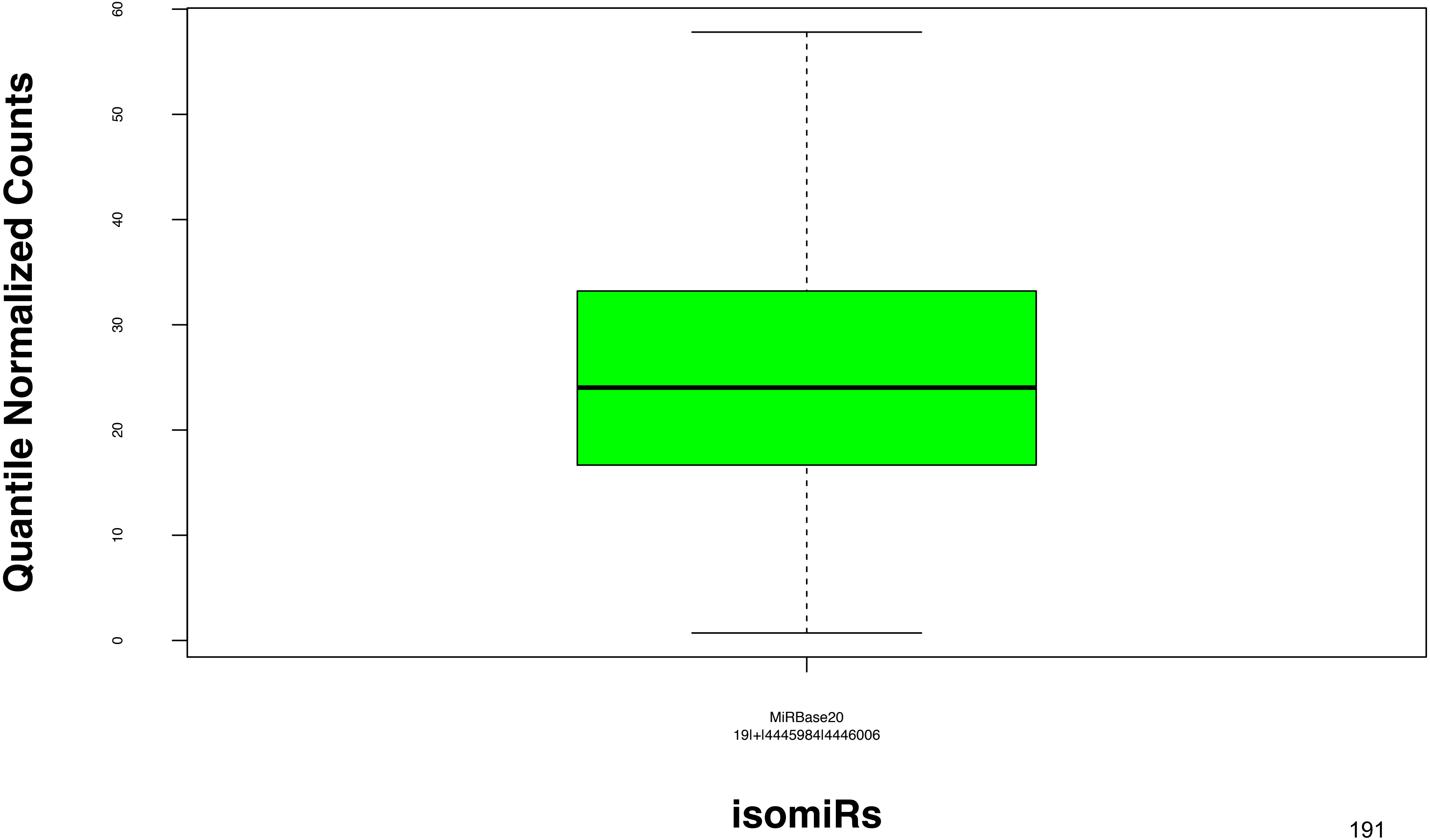

Supp. Figure 1
(pages 1 to 194)

MIMAT0004497=hsa−miR−24−2−5p=MI0000081

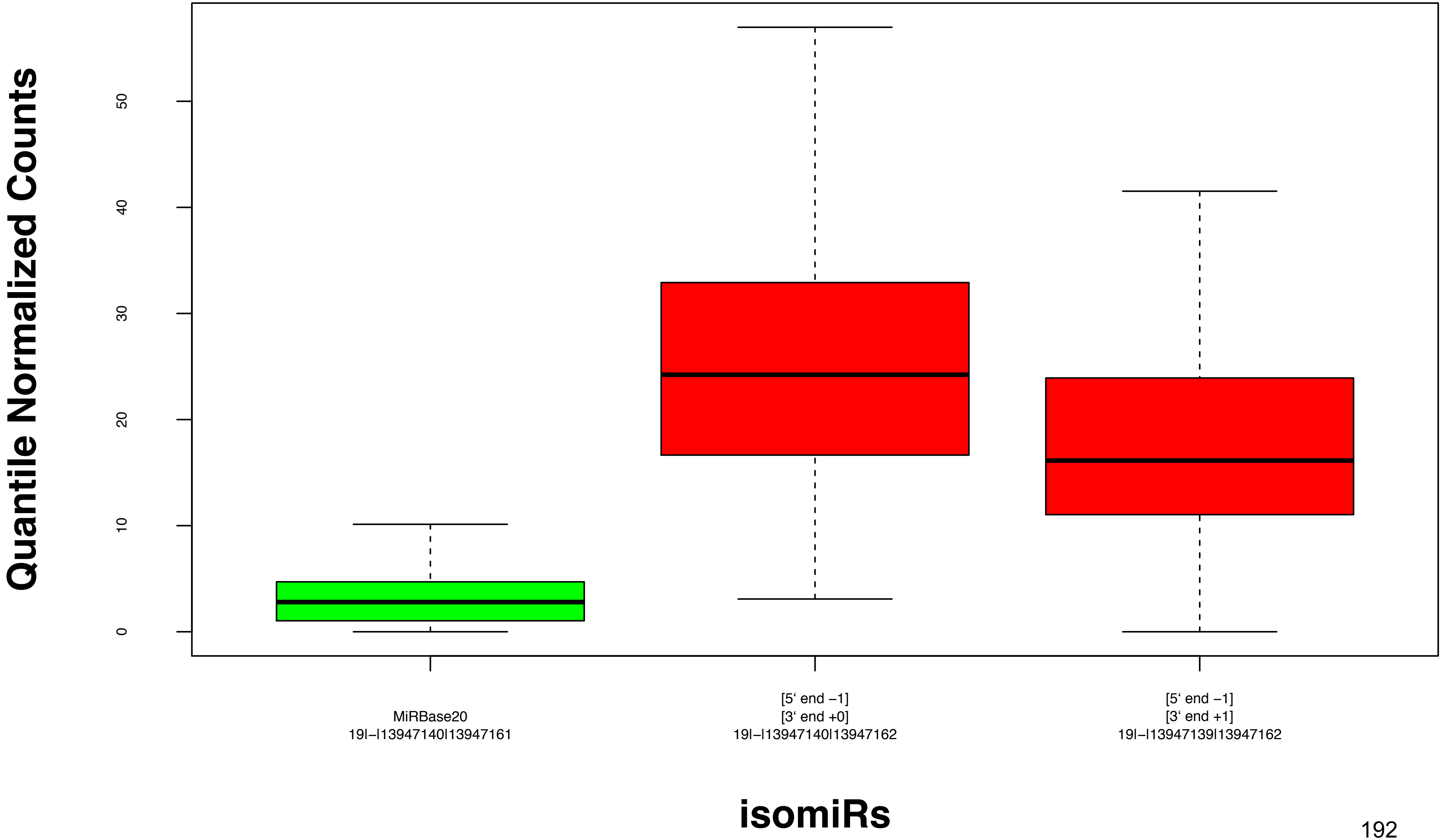

Supp. Figure 1
(pages 1 to 194)

MIMAT0004694=hsa−miR−342−5p=MI0000805

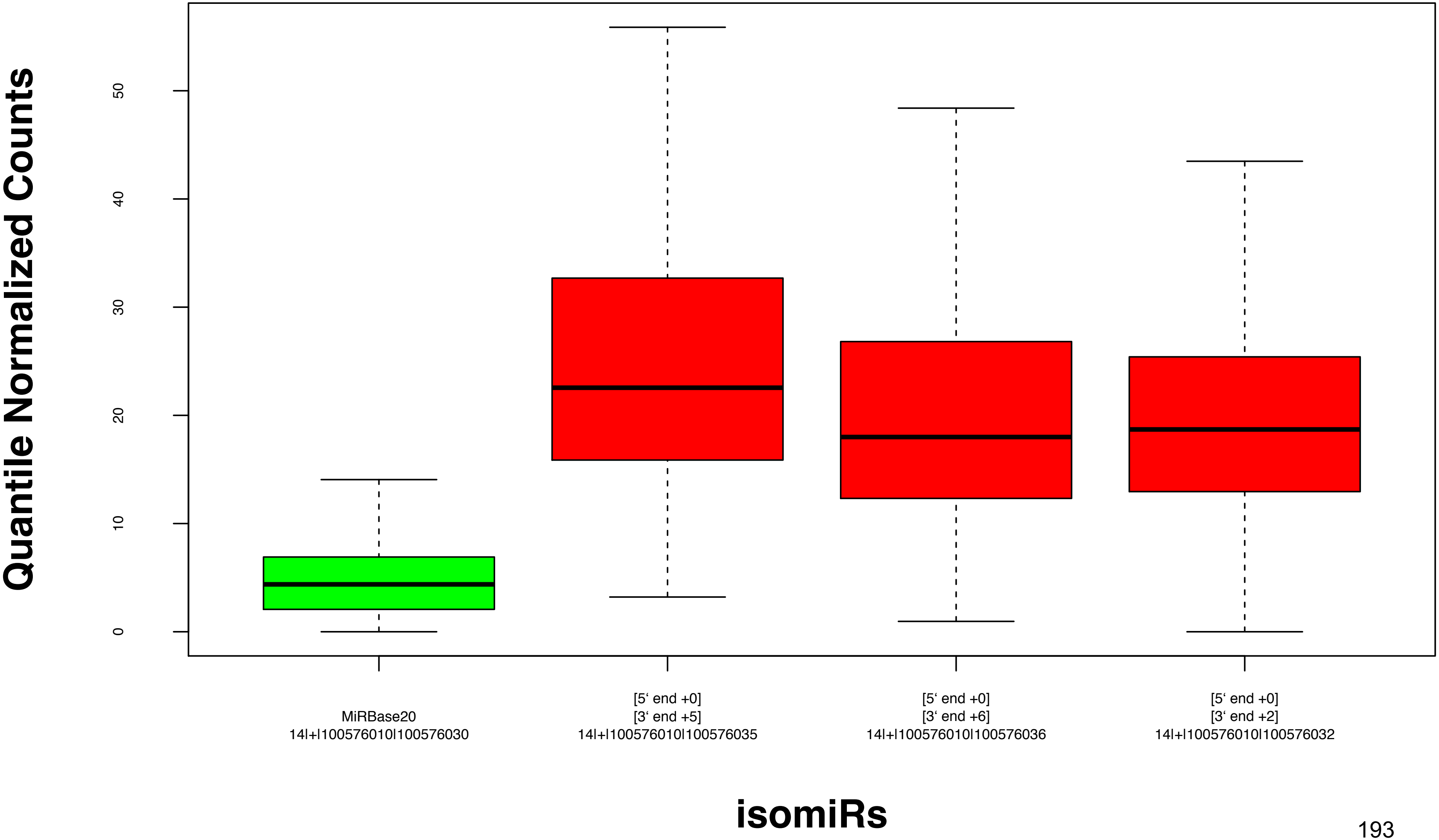

Supp. Figure 1
(pages 1 to 194)

MIMAT0000269=hsa–miR–212–3p=MI0000288

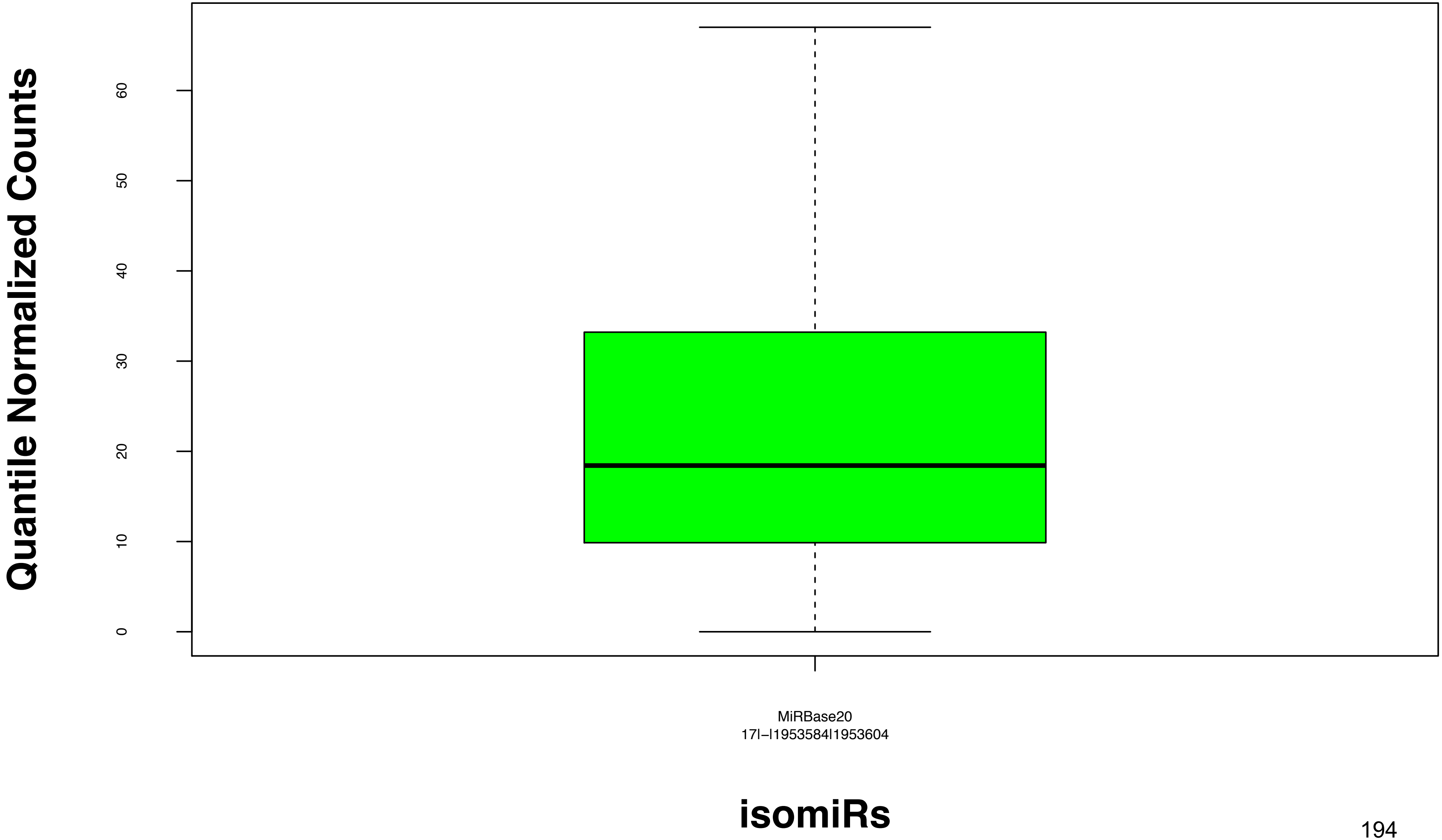

CEU vs All

Supp. Figure 2
(pages 195 to 199)

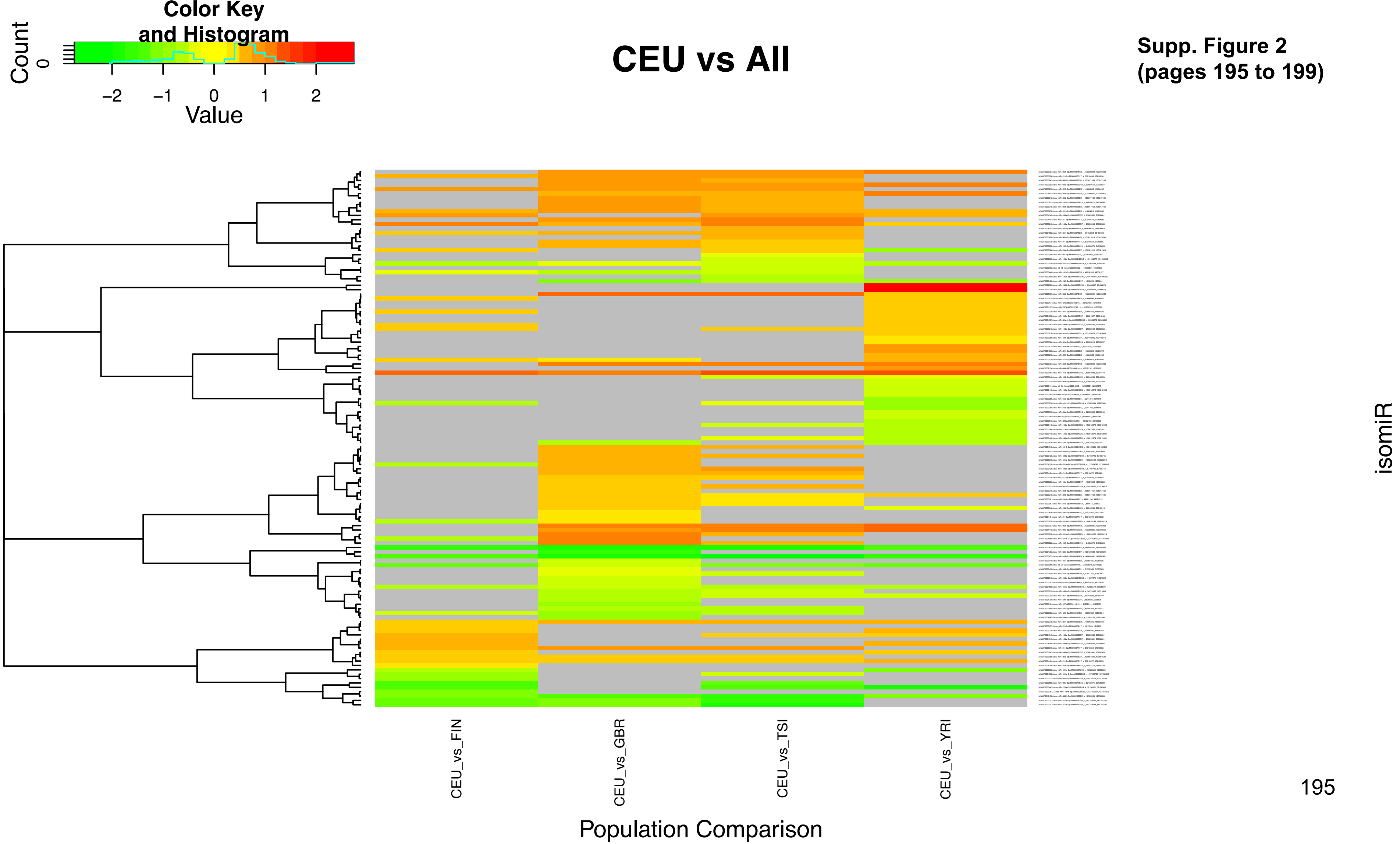

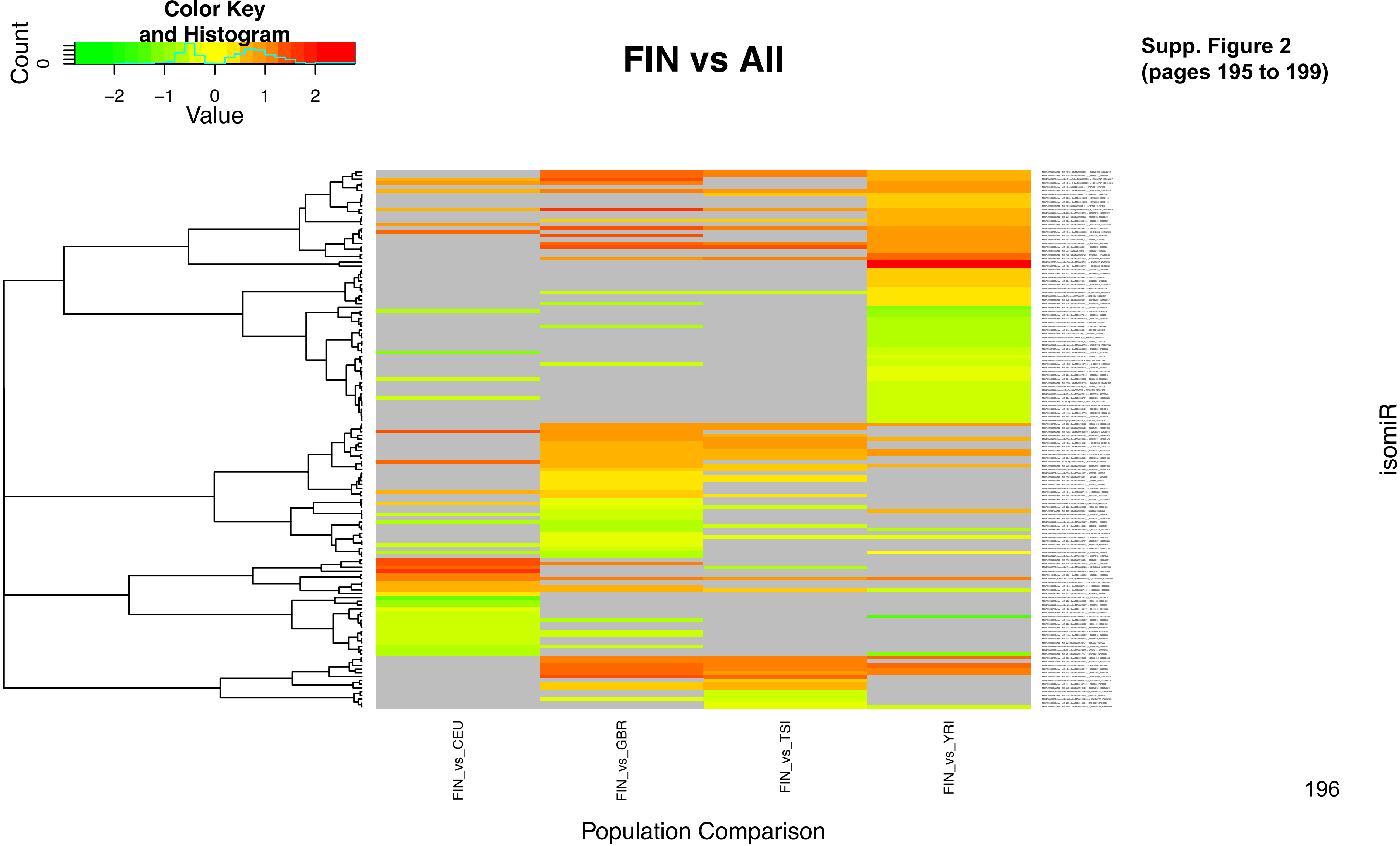

Supp. Figure 2
(pages 195 to 199)

Supp. Figure 2
(pages 195 to 199)

GBR vs All

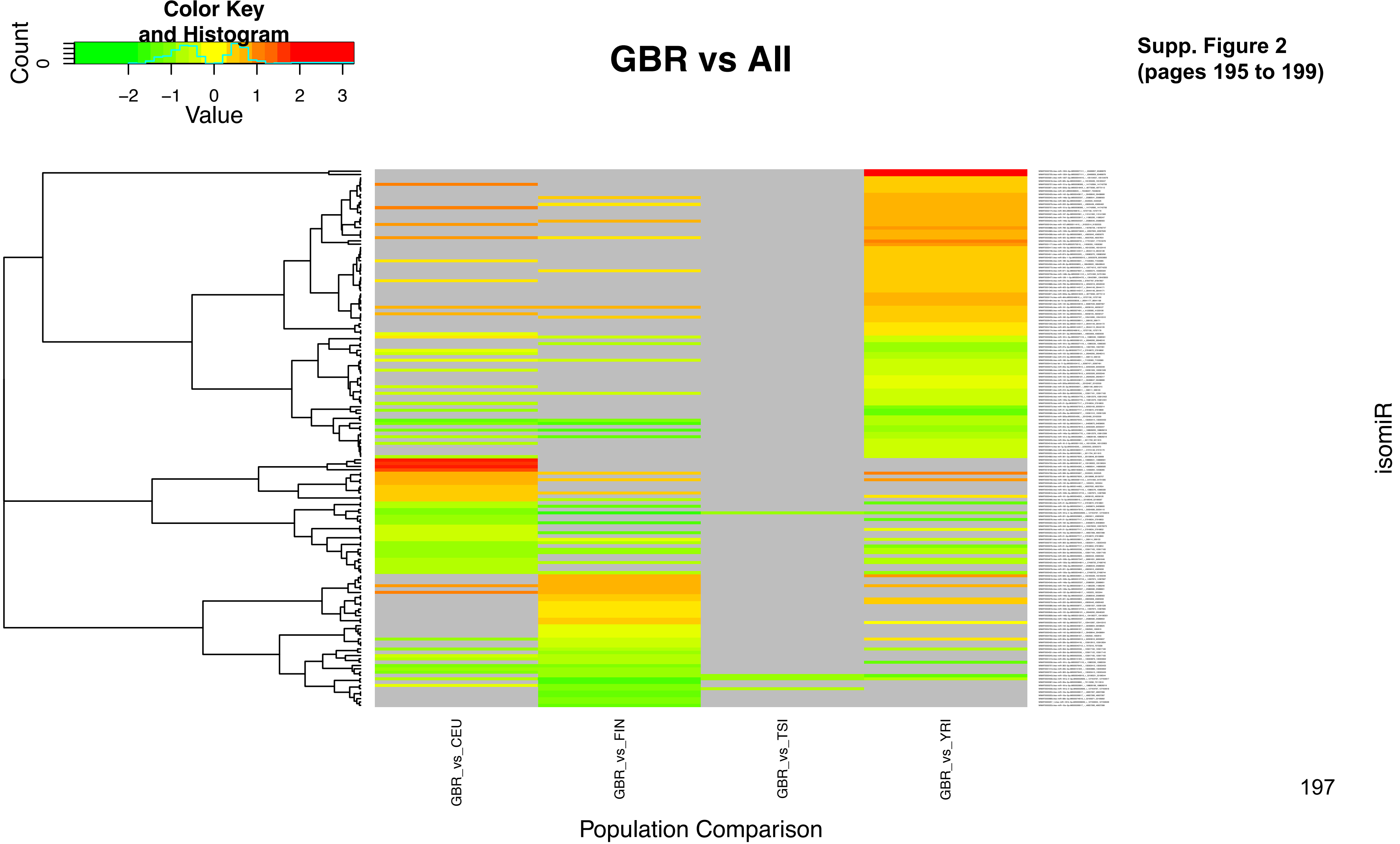

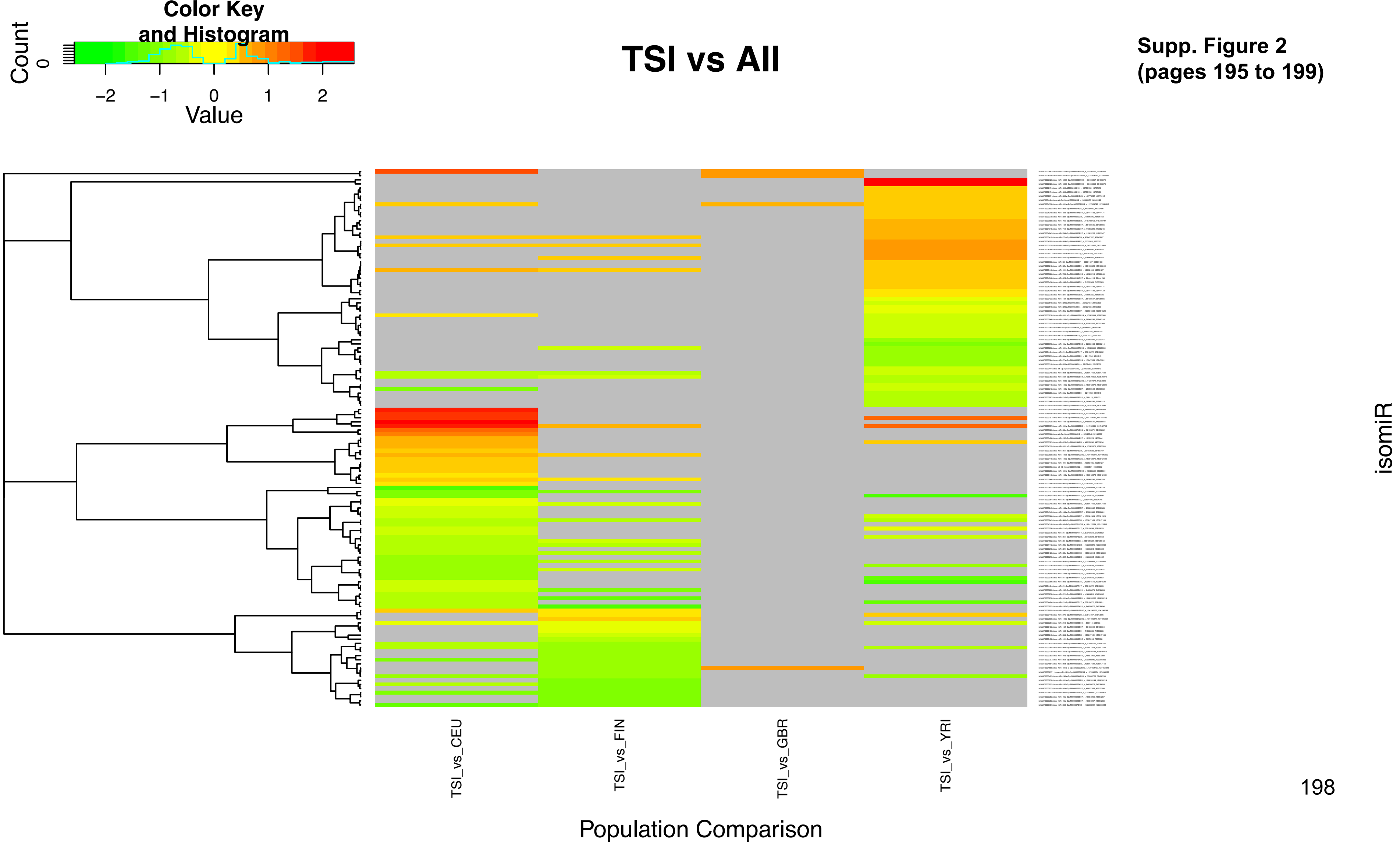

Color Key
and Histogram
Count
0
−2
−1
0
1
2
Value
TSI vs All
Supp. Figure 2
(pages 195 to 199)
isomiR
TSI_vs_CEU
TSI_vs_FIN
TSI_vs_GBR
TSI_vs_YRI
Population Comparison

Supp. Figure 2
(pages 195 to 199)

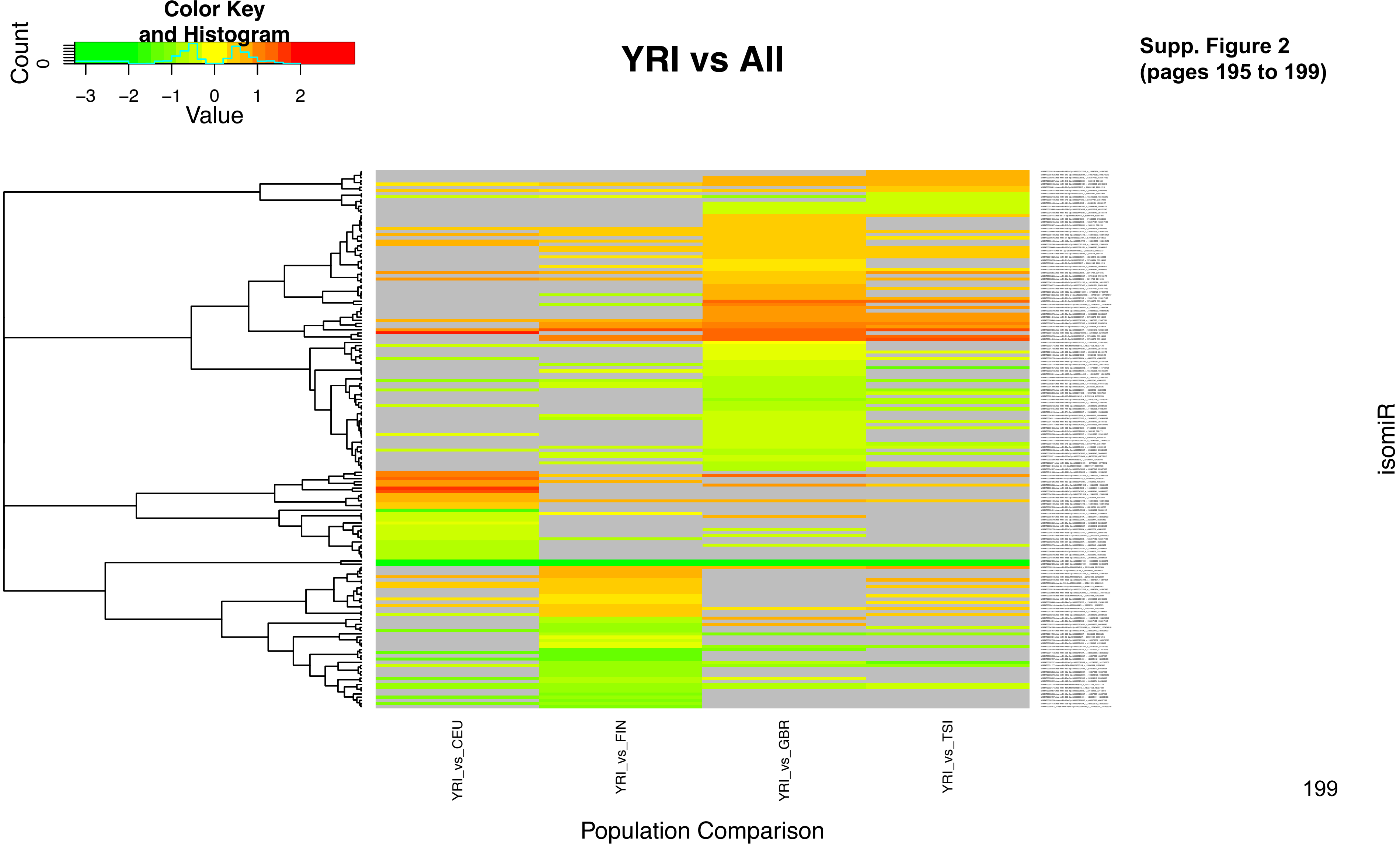

Supp. Figure 3
(pages 200 to 204)

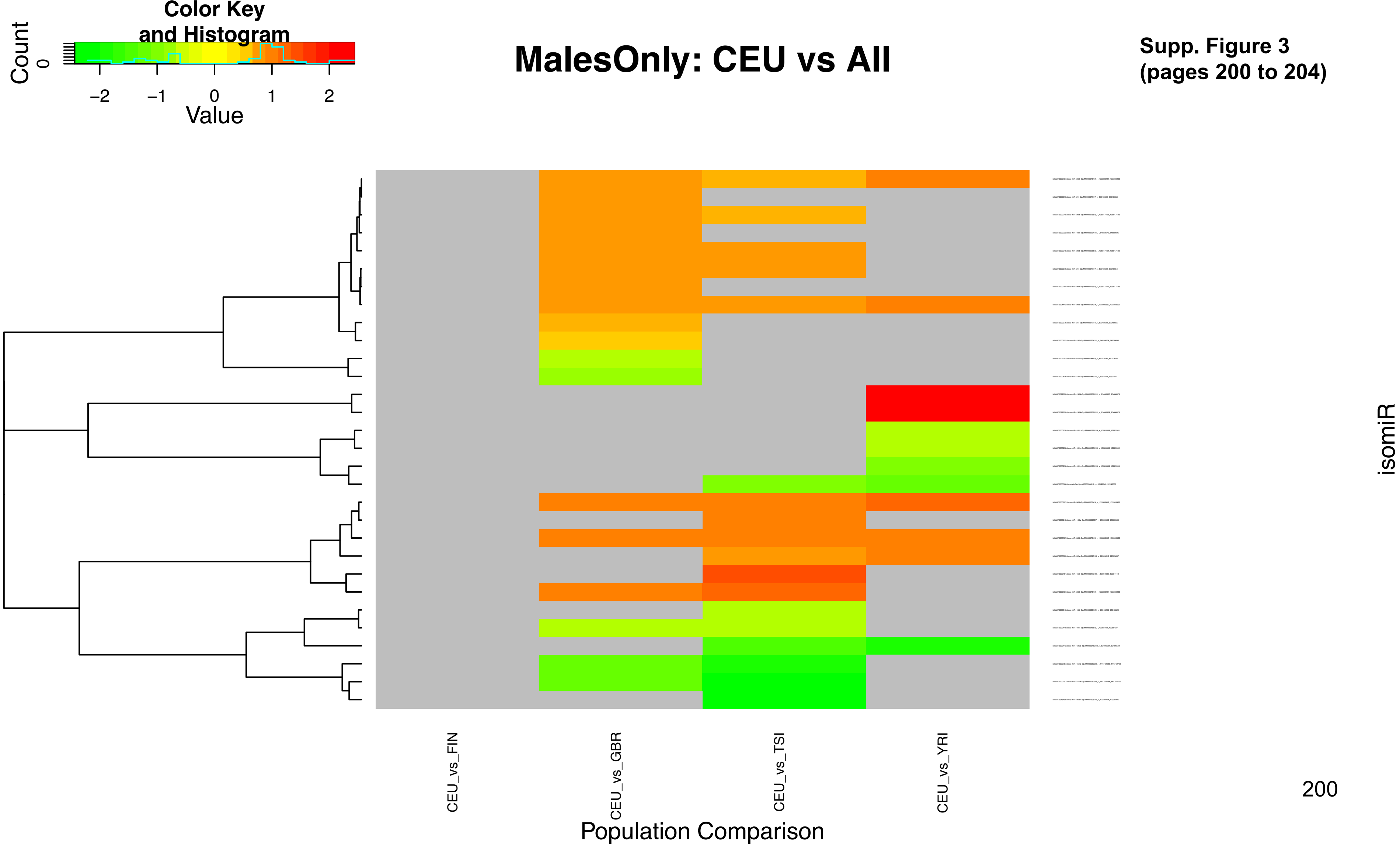

MalesOnly: FIN vs All

Supp. Figure 3
(pages 200 to 204)

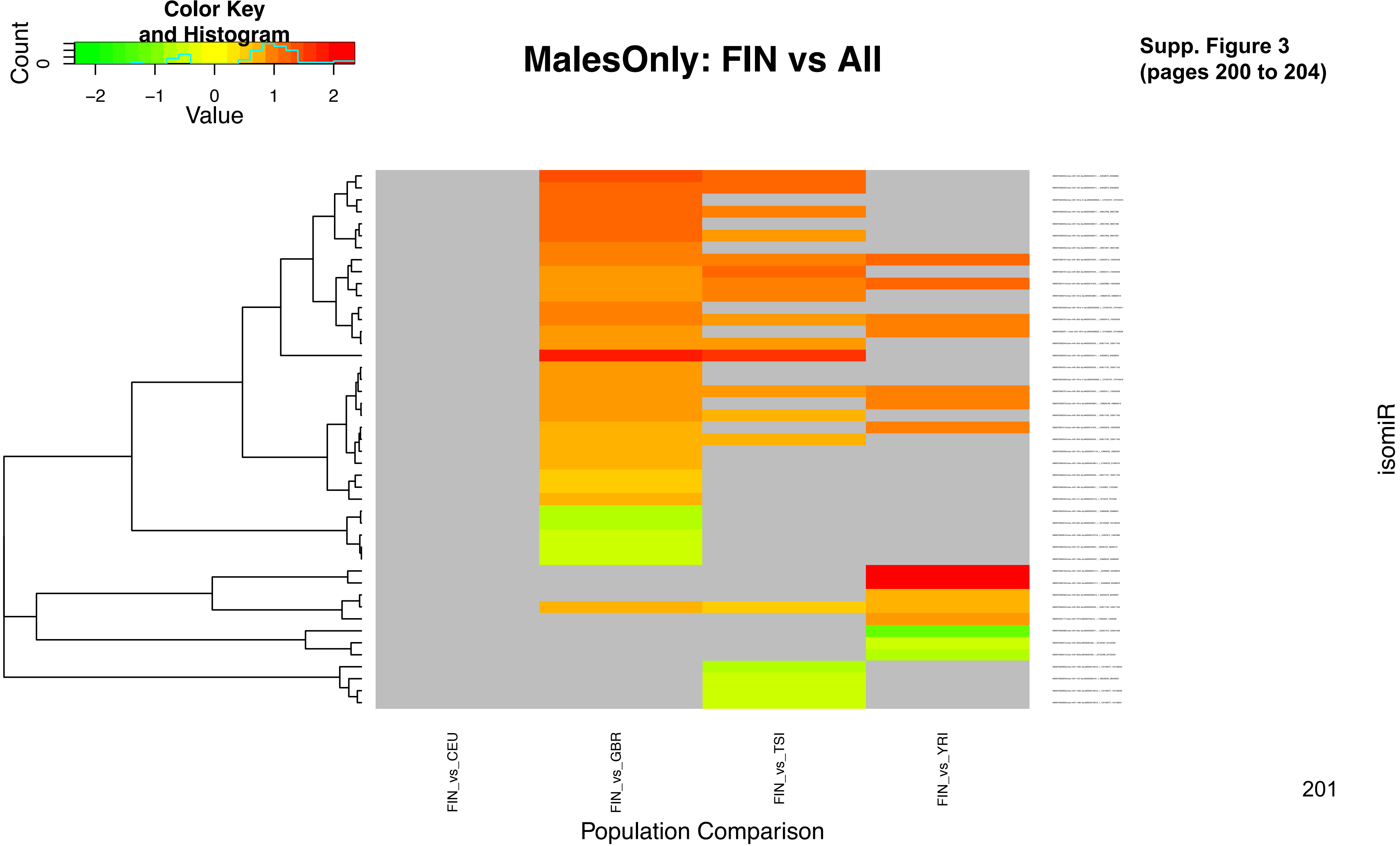

Supp. Figure 3
(pages 200 to 204)

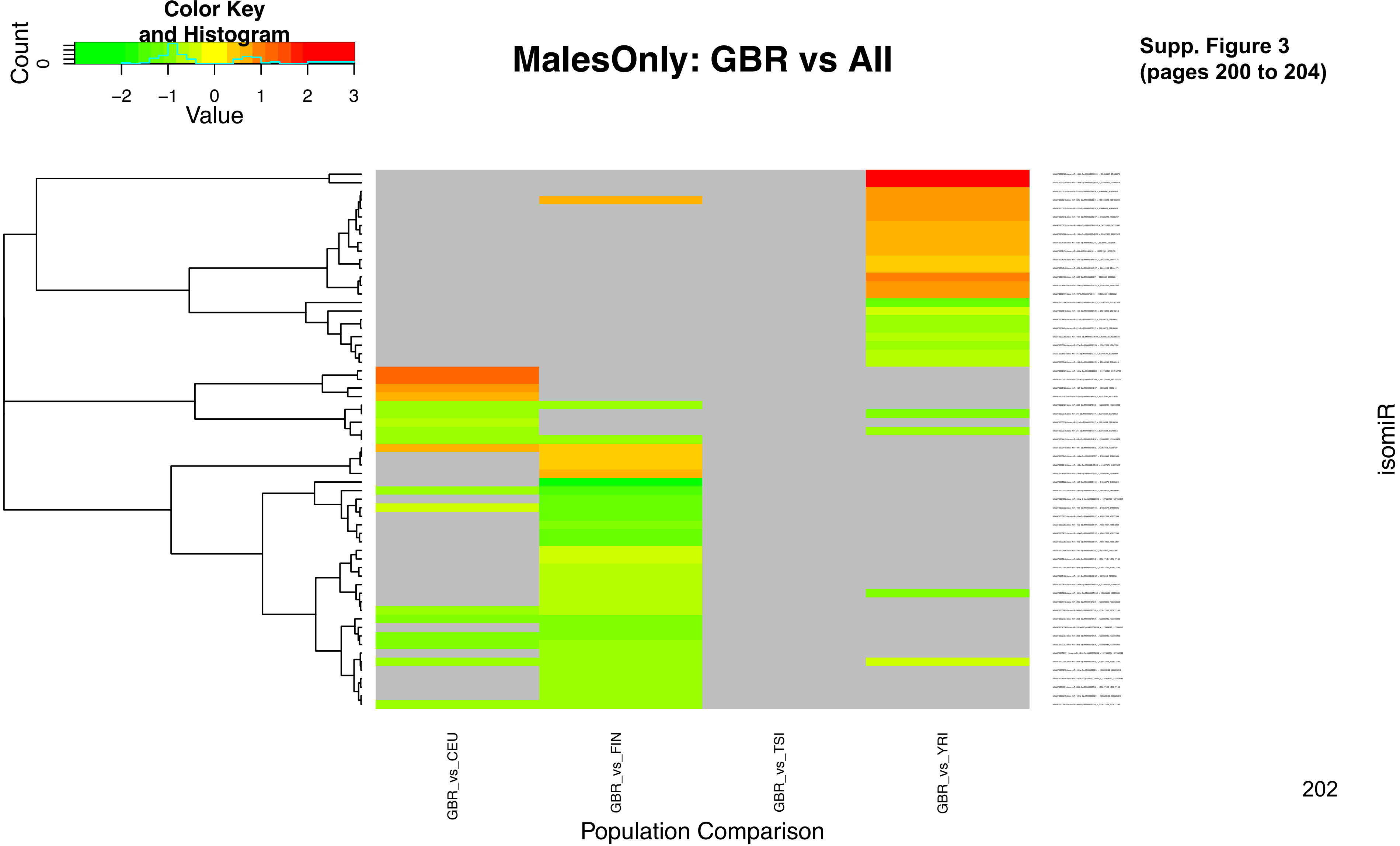

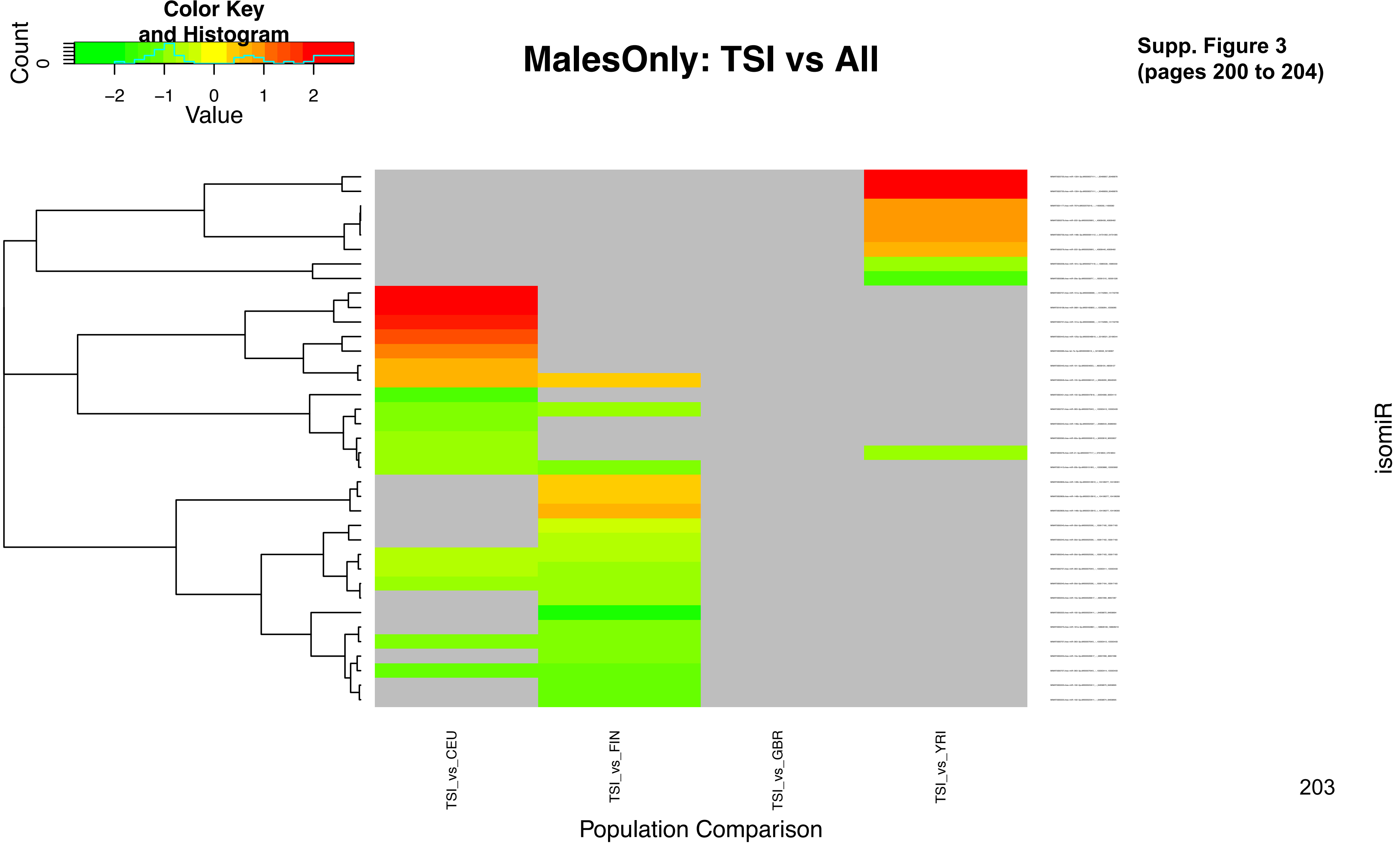
Color Key
and Histogram
Count
0
−2
−1
0
1
2
Value
MalesOnly: TSI vs All
Supp. Figure 3
(pages 200 to 204)
isomiR
TSI_vs_CEU
TSI_vs_FIN
TSI_vs_GBR
TSI_vs_YRI
Population Comparison

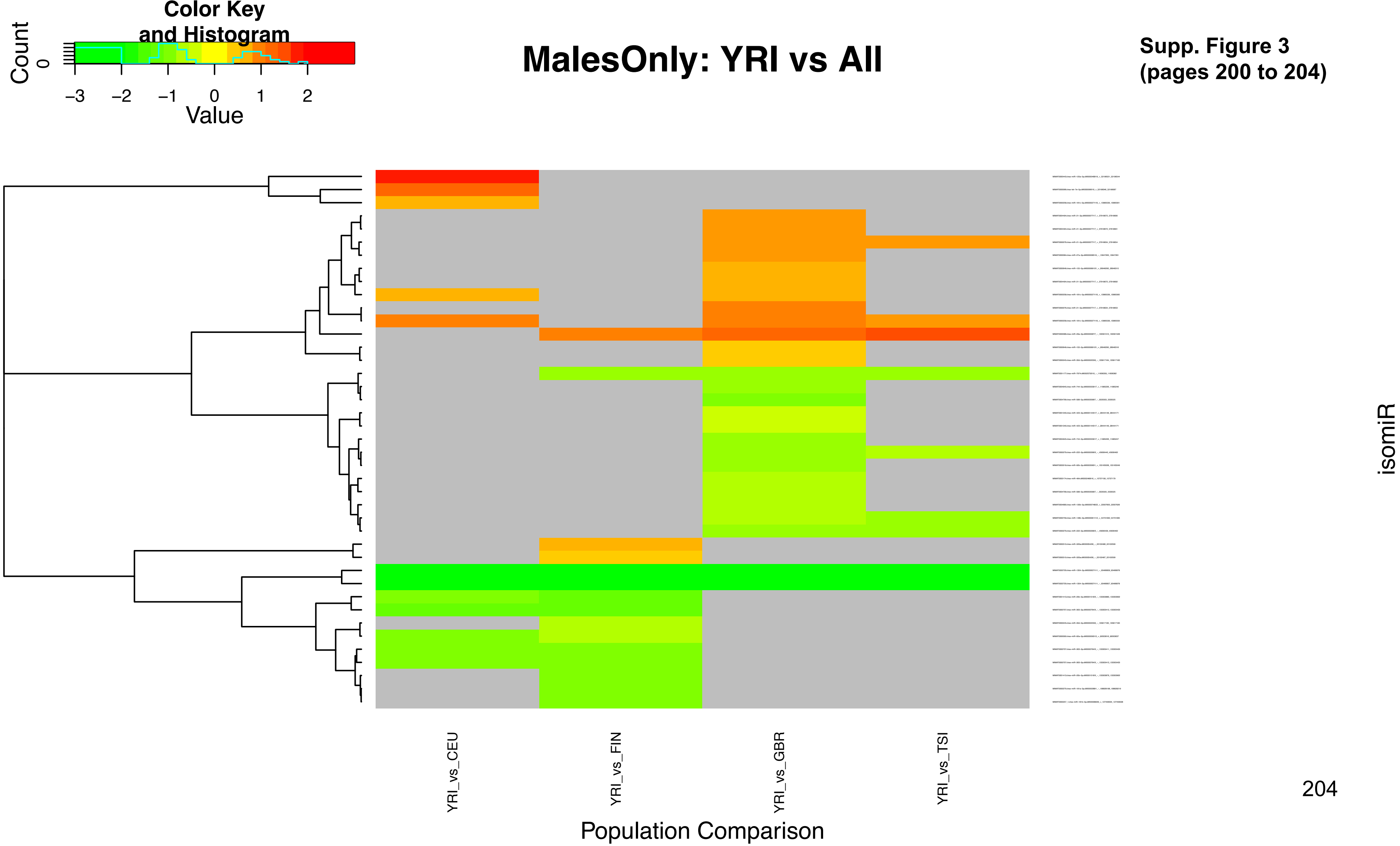

Supp. Figure 3
(pages 200 to 204)

Supp. Figure 4
(pages 205-209)

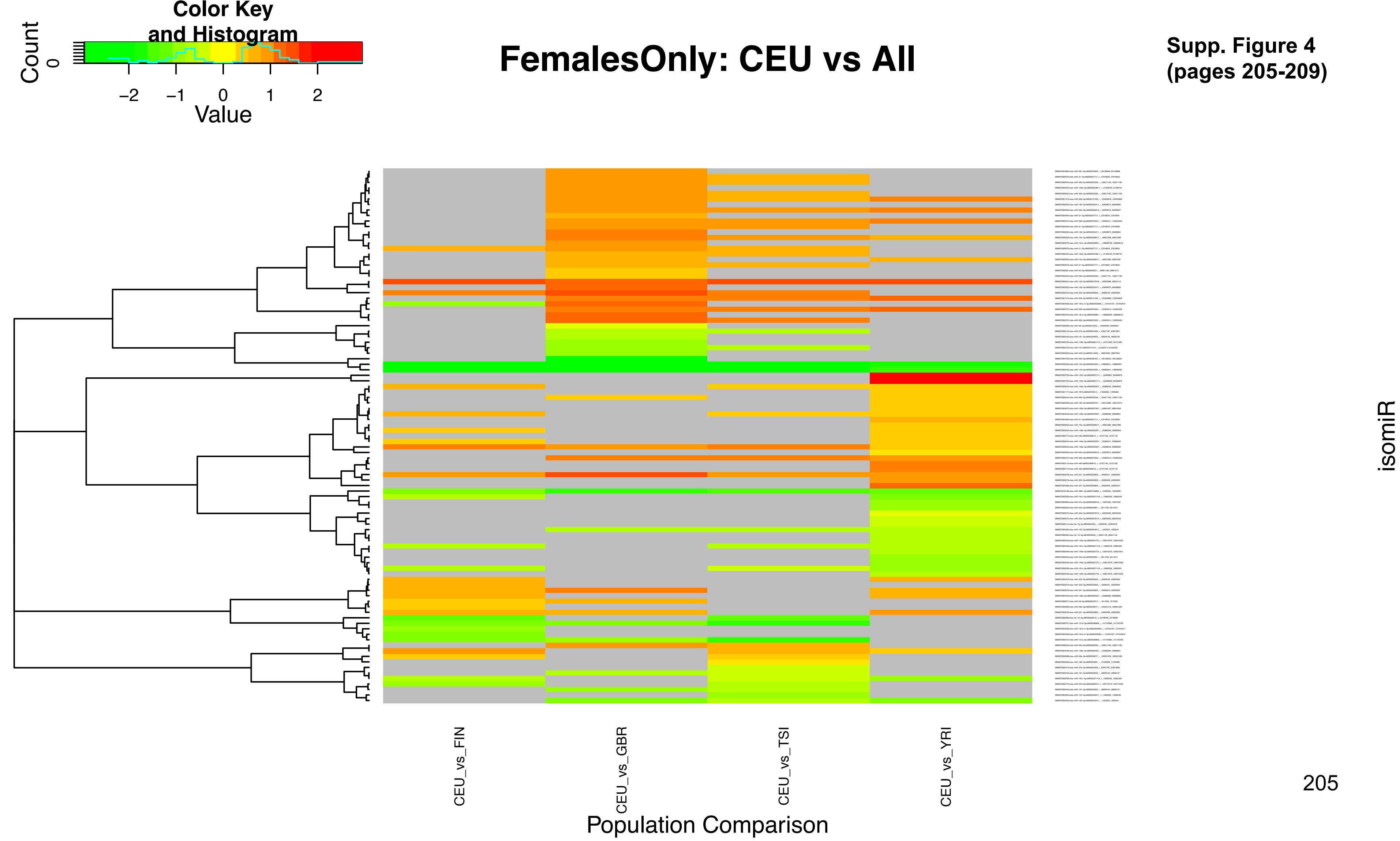

Supp. Figure 4
(pages 205-209)

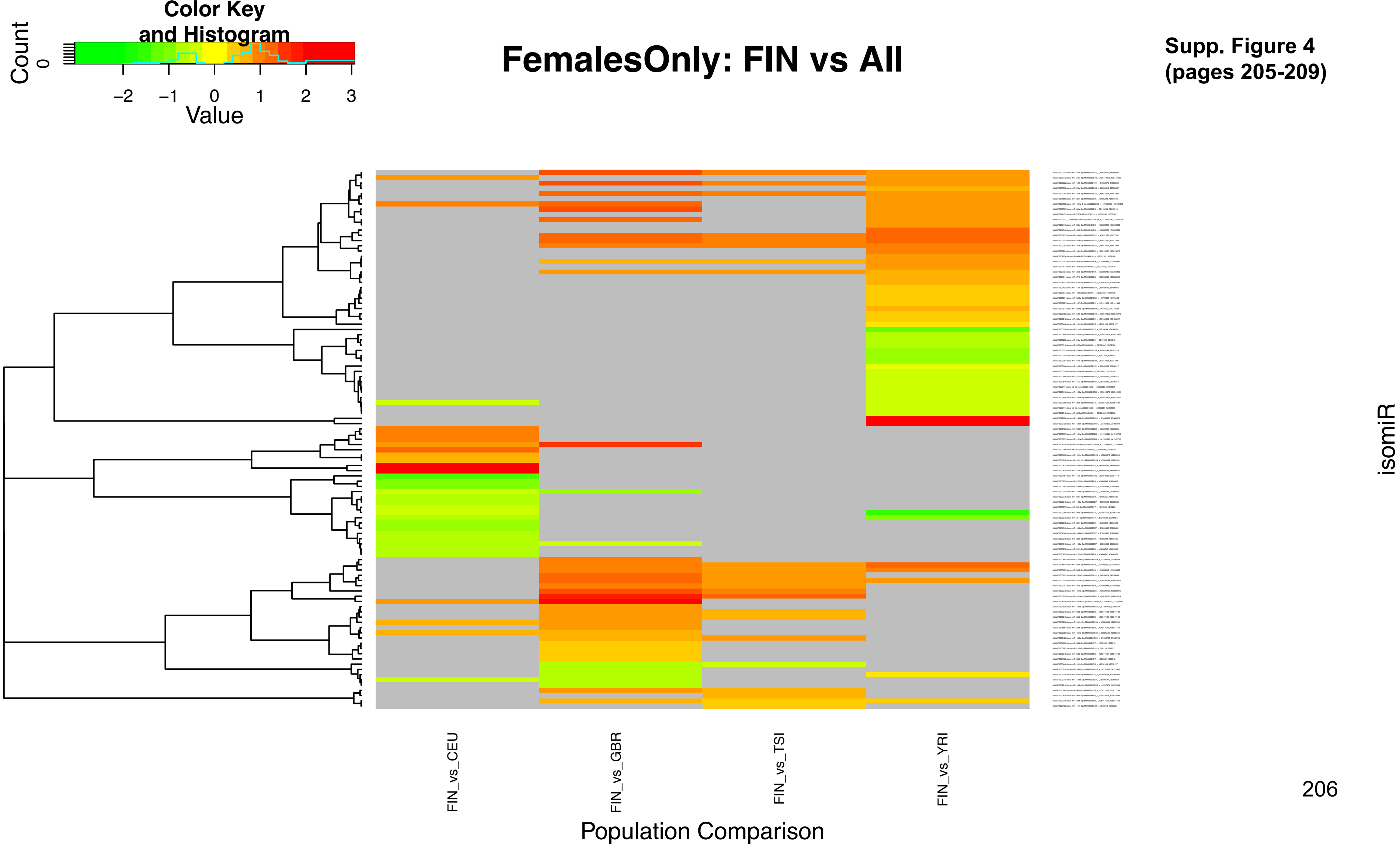

FemalesOnly: GBR vs All

Supp. Figure 4 (pages 205-209)

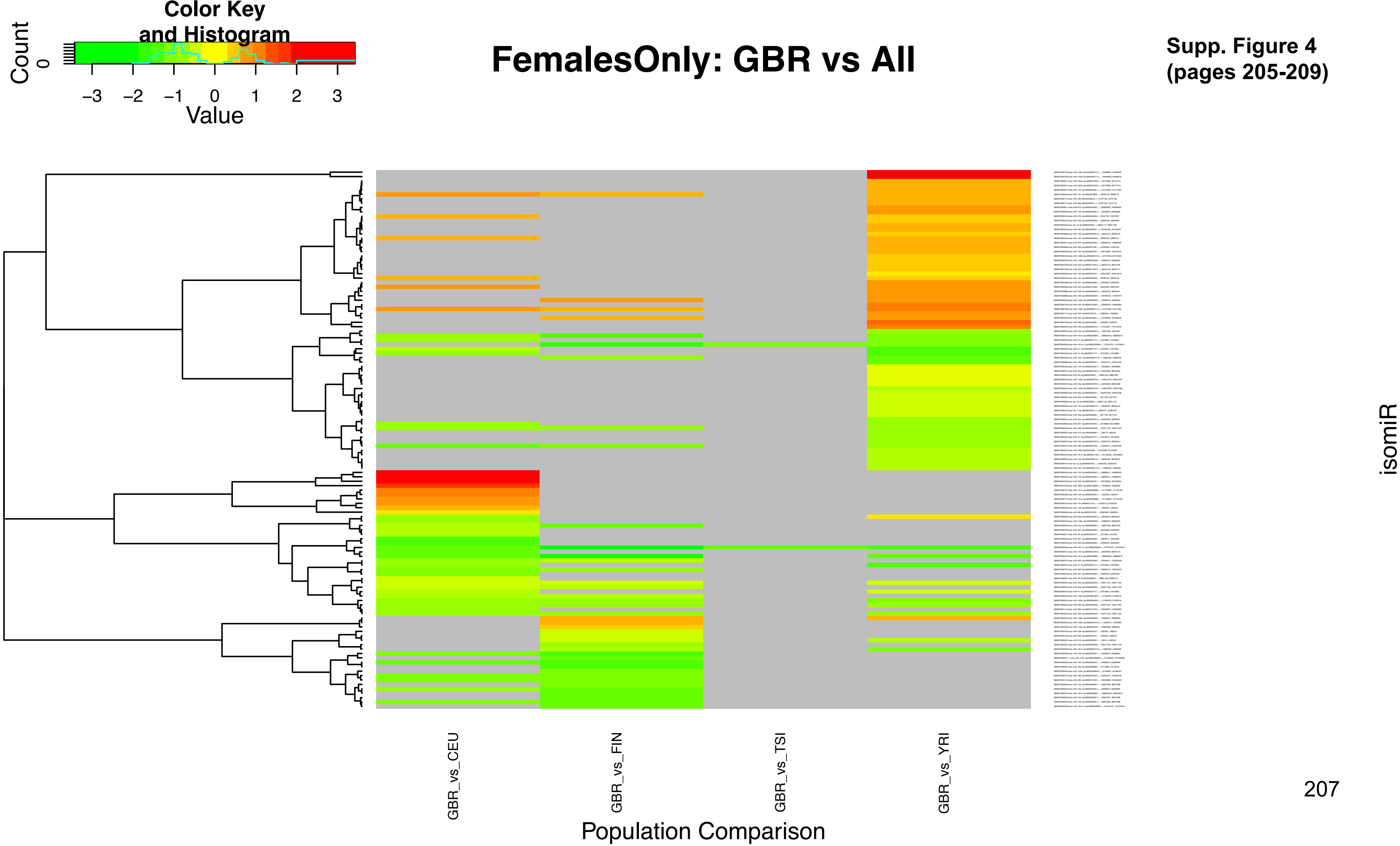

Supp. Figure 4
(pages 205-209)

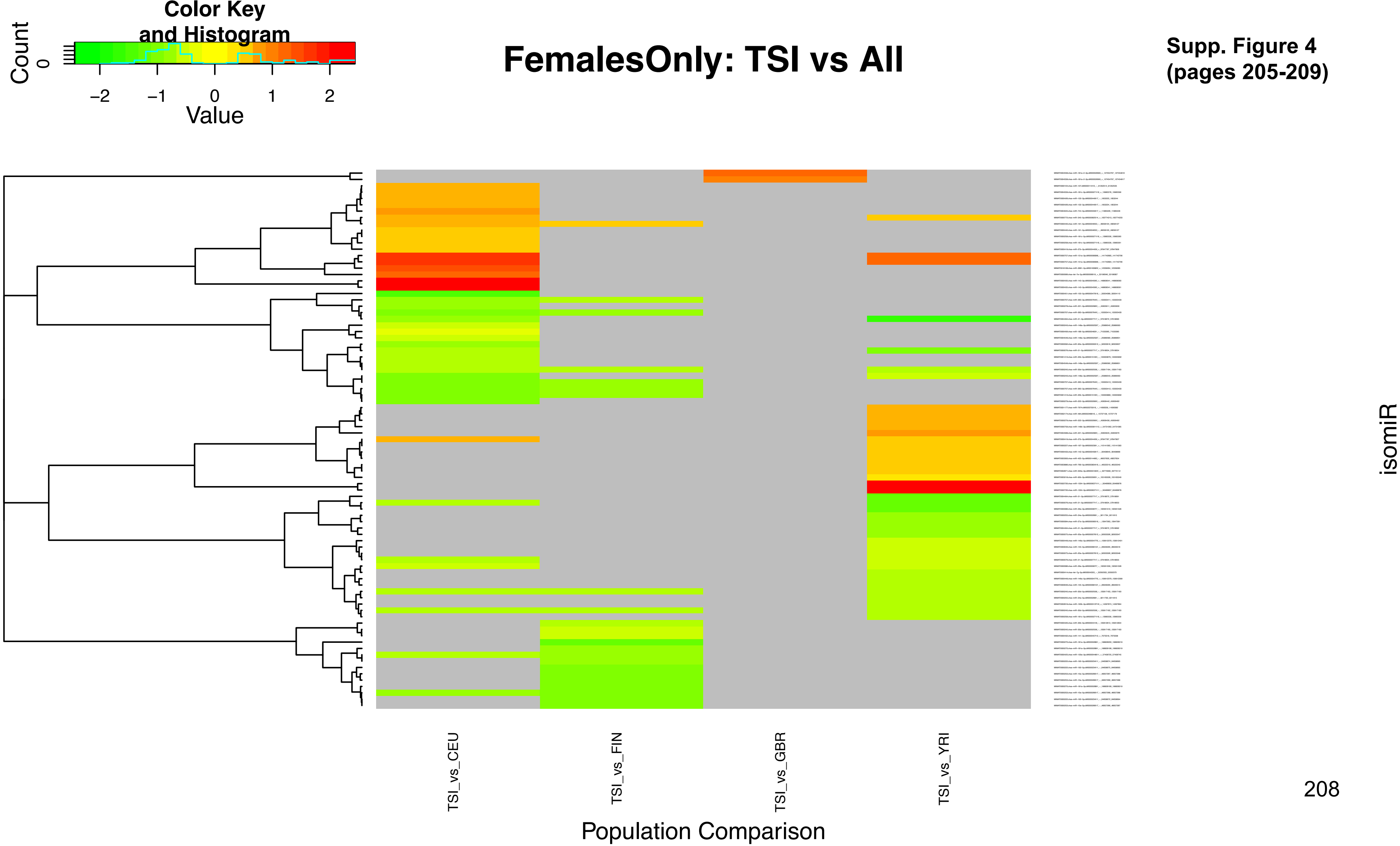

FemalesOnly: YRI vs All

Supp. Figure 4
(pages 205-209)

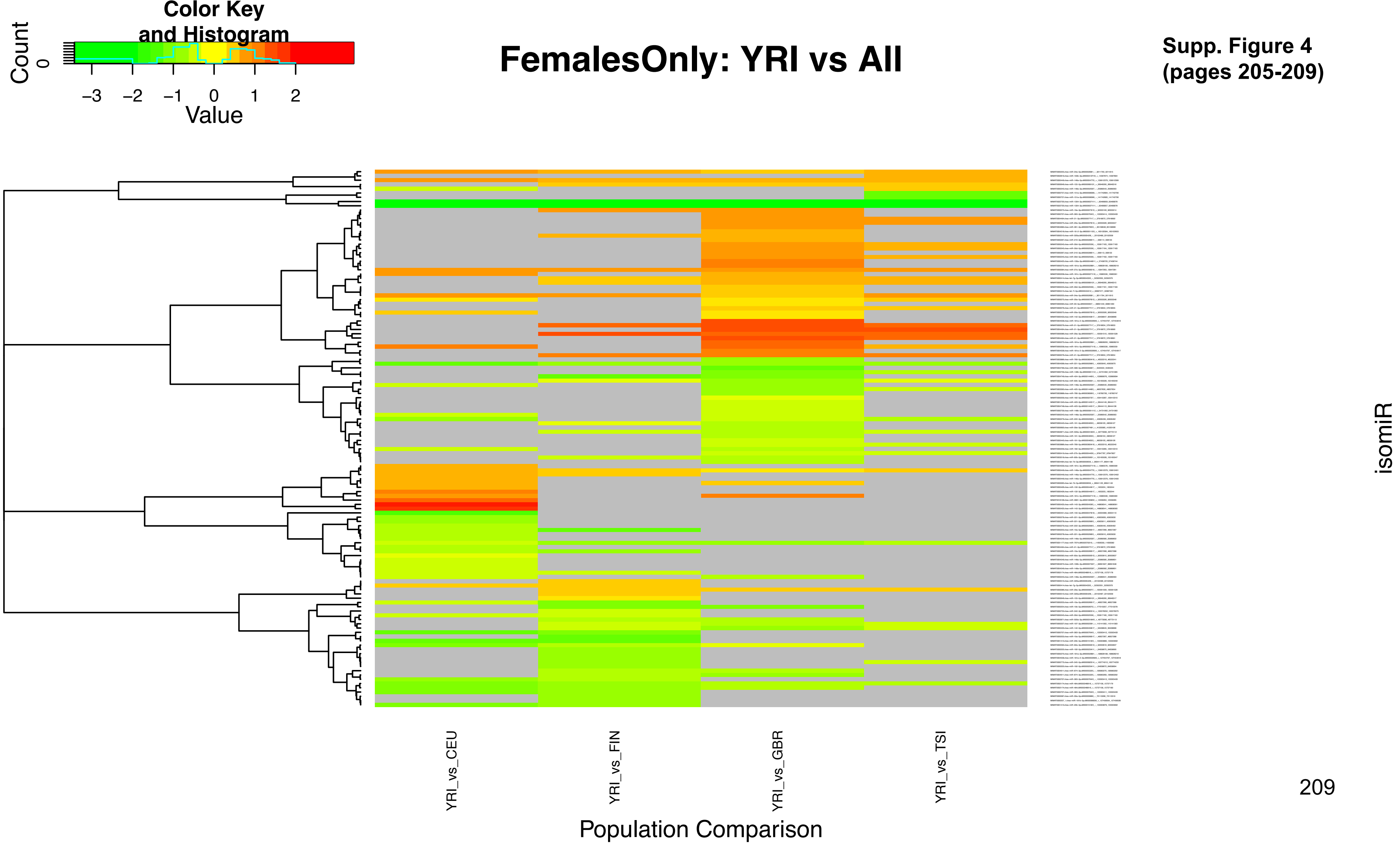

Supp. Figure 5
(page 210)

.

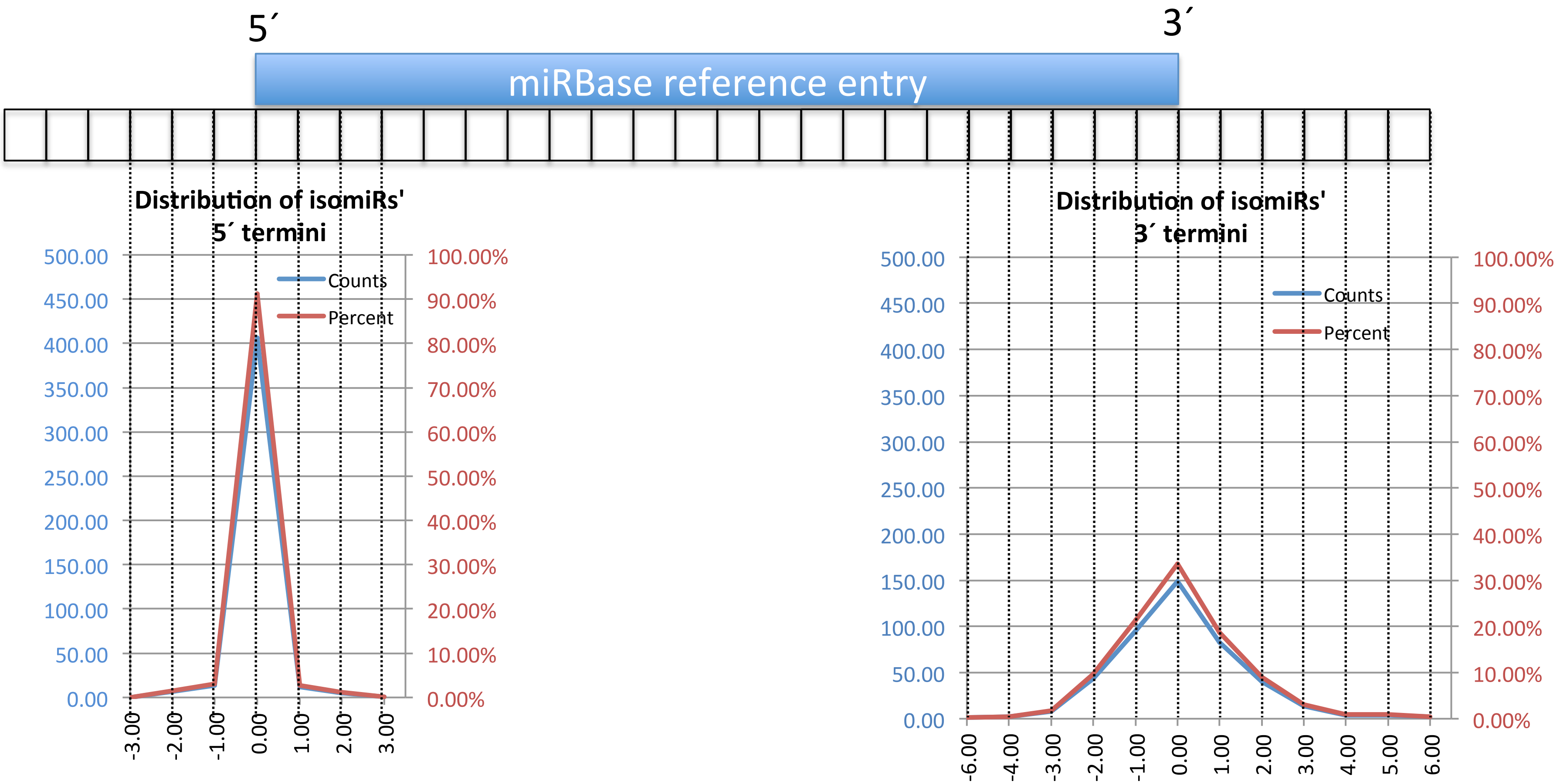

Supp. Table 1. Statistics for each population separately for the single most abundant isomiR produced from a given precursor arm. The most abundant isomiR's identity was determined by analyzing the technical replicates separately for each of the populations. At each locus, we also generated the "conservative" estimate by requiring that the most abundant isomiR have higher expression than the miRBase reference by at least 0.585 log2 units.

	**CEU**		**FIN**		**GBR**		**TSI**		**YRI**	
	Number	Percentage	Number	Percentage	Number	Percentage	Number	Percentage	Number	Percentage
MiRNA precursor arms considered	197		190		170		192		188	
MiRBase miRNA sequence not among the 95-th percentile	63	32%	61	32%	56	33%	66	34%	57	30%
Top isomiR not in agreement with miRBase	99	50%	93	49%	92	54%	97	51%	96	51%
Top isomiR (conservative) not in agreement with miRBase	86	44%	84	44%	77	45%	87	45%	83	44%
Top isomiR disagrees with miRBase at 5' end	13	7%	13	7%	10	6%	13	7%	10	5%
Top isomiR disagrees with miRBase at 5' end ***only***	3	2%	2	1%	2	1%	3	2%	2	1%
Top isomiR disagrees with miRBase at 3' end	96	49%	91	48%	90	53%	94	49%	94	50%
Top isomiR disagrees with miRBase at 3' end ***only***	86	44%	80	42%	82	48%	84	44%	86	46%
Top isomiR disagrees with miRBase at ***both ends***	10	5%	11	6%	8	5%	10	5%	8	4%

Supp. Table 2. Statistics for each population separately for the single most abundant isomiR produced from a given precursor arm – using only females. The most abundant isomiR's identity was determined by analyzing the entire collection of all unique datasets that passed quality control and belonged to females. At each locus, we also generated the "conservative" estimate by requiring that the most abundant isomiR have higher expression than the miRBase reference by at least 0.585 log2 units.

	CEU		FIN		GBR		TSI		YRI	
	Number	Percentage	Number	Percentage	Number	Percentage	Number	Percentage	Number	Percentage
MiRNA precursor arms considered	198		189		193		182		199	
MiRBase miRNA sequence not among the 95-th percentile	57	30%	52	28%	58	31%	56	30%	59	30%
Top isomiR not in agreement with miRBase	94	47%	91	48%	95	49%	87	48%	97	49%
Top isomiR (conservative) not in agreement with miRBase	84	42%	81	43%	87	45%	79	43%	87	44%
Top isomiR disagrees with miRBase at 5' end	13	7%	12	6%	12	6%	10	5%	13	7%
Top isomiR disagrees with miRBase at 5' end ***only***	2	1%	3	2%	3	2%	1	1%	2	1%
Top isomiR disagrees with miRBase at 3' end	92	46%	88	47%	92	48%	86	47%	95	48%
Top isomiR disagrees with miRBase at 3' end ***only***	81	41%	79	42%	83	43%	77	42%	84	42%
Top isomiR disagrees with miRBase at ***both ends***	11	6%	9	5%	9	5%	9	5%	11	6%

Supp. Table 3. Statistics for each population separately for the single most abundant isomiR produced from a given precursor arm – using only males. The most abundant isomiR's identity was determined by analyzing the entire collection of all unique datasets that passed quality control and belonged to males. At each locus, we also generated the "conservative" estimate by requiring that the most abundant isomiR have higher expression than the miRBase reference by at least 0.585 log2 units.

	**CEU**		**FIN**		**GBR**		**TSI**		**YRI**	
	Number	Percentage	Number	Percentage	Number	Percentage	Number	Percentage	Number	Percentage
MiRNA precursor arms considered	192		183		187		189		195	
MiRBase miRNA sequence not among the 95-th percentile	63	32%	58	31%	60	31%	54	30%	62	31%
Top isomiR not in agreement with miRBase	92	48%	87	48%	91	49%	92	49%	93	48%
Top isomiR (conservative) not in agreement with miRBase	81	42%	79	43%	82	44%	83	44%	82	42%
Top isomiR disagrees with miRBase at 5' end	13	7%	11	6%	10	5%	11	6%	11	6%
Top isomiR disagrees with miRBase at 5' end ***only***	2	1%	3	2%	3	2%	2	1%	3	2%
Top isomiR disagrees with miRBase at 3' end	90	47%	84	46%	88	47%	90	48%	90	46%
Top isomiR disagrees with miRBase at 3' end ***only***	79	41%	76	42%	81	43%	81	43%	82	42%
Top isomiR disagrees with miRBase at ***both ends***	11	6%	8	4%	7	4%	9	5%	8	4%
